# Unimolecular Reactions of 2-Methyloxetanyl and 2-Methyloxetanylperoxy Radicals

**DOI:** 10.1021/acs.jpca.3c03918

**Published:** 2023-08-03

**Authors:** Anna C. Doner, Nicholas S. Dewey, Brandon Rotavera

**Affiliations:** † University of Georgia, Department of Chemistry, Athens, Georgia 30602, United States; ‡ University of Georgia, College of Engineering, Athens, Georgia 30602, United States

## Abstract

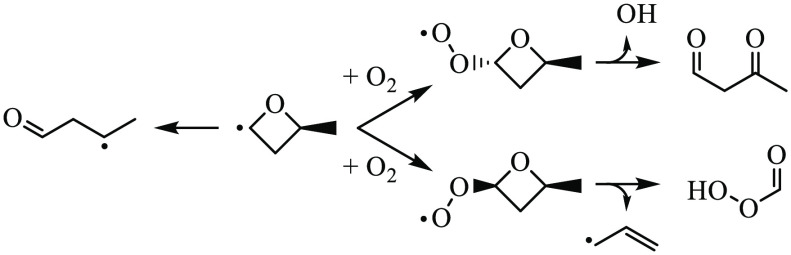

Alkyl-substituted cyclic ethers are intermediates formed in abundance during the low-temperature oxidation of hydrocarbons and biofuels via a chain-propagating step with ȮH. Subsequent reactions of cyclic ether radicals involve a competition between ring opening and reaction with O_2_, the latter of which enables pathways mediated by hydroperoxy-substituted carbon-centered radicals (Q̇OOH). Due to the resultant implications of competing unimolecular and bimolecular reactions on overall populations of ȮH, detailed insight into the chemical kinetics of cyclic ethers remains critical to high-fidelity numerical modeling of combustion. Cl-initiated oxidation experiments were conducted on 2-methyloxetane (an intermediate of *n*-butane oxidation) using multiplexed photoionization mass spectrometry (MPIMS), in tandem with calculations of stationary point energies on potential energy surfaces for unimolecular reactions of 2-methyloxetanyl and 2-methyloxetanylperoxy isomers. The potential energy surfaces were computed using the KinBot algorithm with stationary points calculated at the CCSD(T)-F12/cc-pVDZ-F12 level of theory. The experiments were conducted at 6 Torr and two temperatures (650 K and 800 K) under pseudo-first-order conditions to facilitate Ṙ + O_2_ reactions. Photoionization spectra were measured from 8.5 eV to 11.0 eV in 50-meV steps, and relative yields were quantified for species consistent with Ṙ → products and Q̇OOH → products. Species detected in the MPIMS experiments are linked to specific radicals of 2-methyloxetane. Species from Ṙ → products include methyl, ethene, formaldehyde, propene, ketene, 1,3-butadiene, and acrolein. Ion signals consistent with products from alkyl radical oxidation were detected, including for Q̇OOH-mediated species, which are also low-lying channels on their respective potential energy surfaces. In addition to species common to alkyl oxidation pathways, ring-opening reactions of Q̇OOH radicals derived from 2-methyloxetane produced ketohydroperoxide species (performic acid and 2-hydroperoxyacetaldehyde), which may impart additional chain-branching potential, and dicarbonyl species (3-oxobutanal and 2-methylpropanedial), which often serve as proxies for modeling reaction rates of ketohydroperoxides. The experimental and computational results underscore that reactions of cyclic ethers are inherently more complex than currently prescribed in chemical kinetic models utilized for combustion.

## 1 Introduction

**Figure 1:**
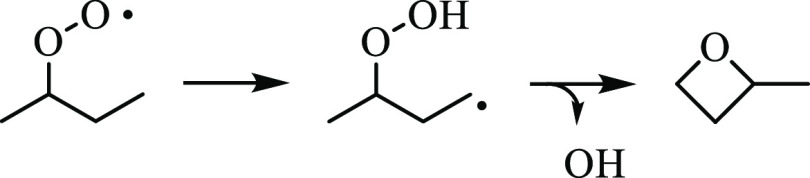
Formation pathway of 2-methyloxetane from 2-butyl + O_2_ in a chain-propagating step with ȮH.

The direct detection of intermediates formed from hydroperoxy-substituted carbon-centered radicals (Q̇OOH) remains paramount to chemical kinetics modeling of combustion^1−3^ because the precise balance between the unimolecular reaction and bimolecular reaction with O_2_ governs the rate of proliferation of reactive species. Q̇OOH radicals are produced via isomerization of organic peroxy radicals (ROȮ) at temperatures below 1000 K,^4^ the subsequent reactions of which depend in part on molecular structure.^5^ Unimolecular reactions of Q̇OOH in the forward direction lead to intermediates produced from three pathways: (1) C–O β-scission of β-Q̇OOH, yielding conjugate alkene + HOȮ; (2) concerted O–O bond scission and C–O bond formation, yielding cyclic ether + ȮH (Figure 1); and (3) concerted C–C and O–O β-scission, yielding carbonyls/alkenes + ȮH. Bimolecular reactions of Q̇OOH with O_2_ (second O_2_-addition) include a chain-branching pathway leading to a net increase in radicals that also produces ketohydroperoxides in the process.^6^

**Figure 2:**
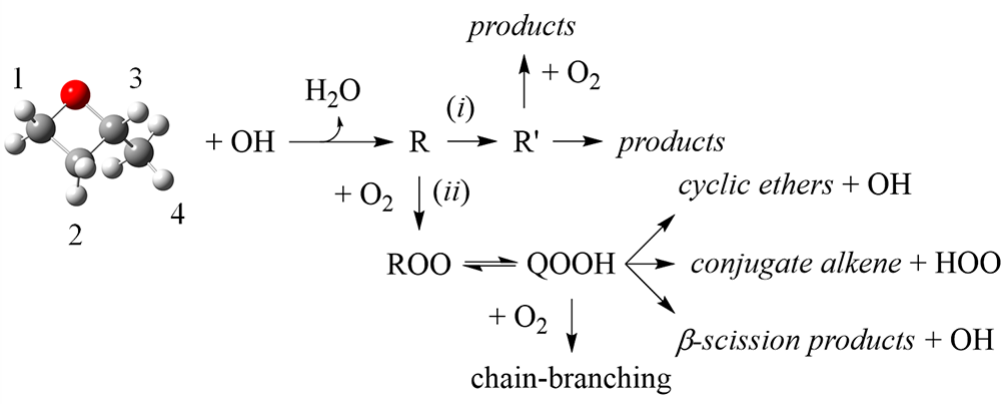
Abstraction of hydrogen from 2-methyloxetane yields four distinct radicals (Ṙ) that subsequently undergo competition between ring opening (*i*) and reactions with O_2_ (*ii*) during low-temperature oxidation. Ṙ′ represents a carbon-centered radical produced from ring opening. Numerical designations indicate distinct H-abstraction sites.

Q̇OOH-isomer-specific intermediates produced from unimolecular reaction (cyclic ethers) and bimolecular reaction (ketohydroperoxides) provide the most direct means for determining reaction rates governing the balance of reactions. However, quantitative, isomer-resolved speciation measurements are required, along with complementary theoretical rate calculations. While several experiments report the direct detection of ketohydroperoxides,^7−13^ with some notable exceptions involving theoretical calculations of photoionization cross sections conducted for ketohydroperoxides derived from dimethyl ether,^14^ ethene ozonolysis,^15^ and *n*-pentane,^16^ quantitative isomer resolution remains difficult. In contrast, cyclic ether isomers are amenable to experimental quantification that provides clear targets for theoretical rate calculations of Q̇OOH → products. However, because the quantification of cyclic ethers occurs most often under steady-state conditions that reflect a balance of formation and consumption reactions, accurate modeling of the chemical kinetics of Q̇OOH is tied directly to a complete description of consumption reactions as depicted in Figure 2.

Subsequent reactions of cyclic ethers, both in the context of biofuels^5,17^ and as oxidation intermediates, are complex^18−22^ because of competing reactions of ring-opening and reaction with O_2_. Common Q̇OOH-derived intermediates from alkane oxidation are substituted three-membered cyclic ethers (alkyloxiranes) and four-membered cyclic ethers (alkyloxetanes). Reaction pathways of alkyloxiranes involve Q̇OOH ring-opening reactions into resonance-stabilized ketohydroperoxide-type radicals^18−20^ and also exhibit stereochemical dependence.^19^ Similar competing pathways also exist for radicals derived from 2,4-dimethyloxetane.^21,22^ Doner et al.^21^ computed stationary points on potential energy surfaces for 2,4-dimethyloxetanyl radicals at the CCSD(T)-F12/cc-pVTZ-F12//ωB97XD/6-311++G(*d*,*p*) level of theory. Solutions to the master equation were then conducted in order to determine the branching fractions of products from ring opening. Rate coefficients were independent of stereochemistry, and major products from ring-opening of (carbon-centered) 2,4-dimethyloxetanyl isomers were methyl, allyl, propene, acetyl, acetaldehyde, 3-butenal, and 1-penten-3-yl-4-ol. Doner et al.^22^ also conducted a similar set of calculations on (oxygen-centered) 2,4-dimethyloxetanylperoxy radicals that revealed diastereomeric-specific reaction pathways due to the orientation of the peroxy group and the accessibility of certain hydrogen atoms in the ROȮ → Q̇OOH step. Conventional alkyl-type Q̇OOH decomposition pathways, such as cyclic ether formation and HOO-elimination, are important and compete with Q̇OOH ring-opening reactions, the balance of which also exhibited a stereochemical dependence. One major conclusion from Doner et al.^21,22^ is that rates of ring-opening of 2,4-dimethyloxetanyl isomers are of the same order of magnitude as those of the addition of O_2_, the net effect of which is a complex and competitive network of reactions that includes chain-branching channels and crossover reactions (i.e., pathways that lead to radicals otherwise produced from the oxidation reactions of other hydrocarbons).

Barrier heights on potential energy surfaces for alkyl + O_2_^23−28^ show for pathways involving the formation of alkyloxetanes that ROȮ → Q̇OOH isomerization, which proceeds via a six-membered transition state, is a low-energy pathway: ∼20 kcal/mol relative to the ROȮ well, depending on the type of carbon (primary/secondary/tertiary) involved. In contrast, Q̇OOH decomposition pathways leading to alkyloxetanes are among the highest barriers (∼30 kcal/mol relative to the ROȮ well) on alkylperoxy surfaces. However, in speciation measurements, oxetanes are quantitatively significant species.^29−32^ In the context of chemical kinetics modeling, consumption reactions of cyclic ethers are often truncated, including only disproportionation reactions such as ȮH + cyclic ether → H_2_O + products, or are treated via species lumping.^33^ Building on developments from reactions of alkyloxiranes and alkyloxetanes,^18−22^ recent mechanism development for *n*-pentane by Liu et al.^34^ accounts for some of the complexity of cyclic ether consumption reactions including the formation of bicyclic ethers and dicarbonyls.

Chemical kinetics insight on the combustion of 2-methyloxetane, an intermediate of *n*-butane, is limited,^35−37^ and products from oxidation reactions are unknown. Duke and Holbrook^35^ measured rate coefficients for H-abstraction for the reaction ĊH_3_ + 2-methyloxetane → products at temperatures below 500 K, wherein methyl radicals were produced via the photolysis of acetone. Zalotai et al.^36^ measured thermal decomposition rates of 2-methyloxetane up to 3 kPa and subsequently, utilized RRKM theory from 659 K to 757 K to model thermal decomposition at pressures above 1.4 kPa.^37^

**Figure 3:**
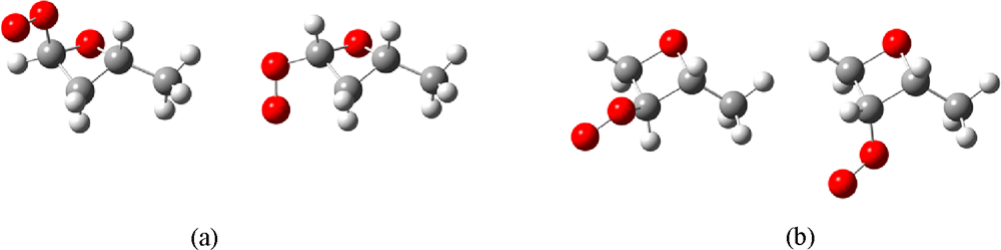
Molecular structure of *anti-* and *syn*-stereoisomers of (a) 2-methyloxetan-4-peroxy and (b) 2-methyloxetan-3-peroxy radicals.

Resulting from H-abstraction, 2-methyloxetane yields four distinct 2-methyloxetanyl radical isomers (Ṙ) that undergo a competition (Figure 2): unimolecular decomposition (*pathway i*) or reaction with O_2_ (*pathway ii*). The balance of reactions unfolding across the two pathways contributes to radical populations formed during *n*-butane oxidation and other systems involving butyl radicals. Stereochemical dependence is introduced into the reaction network of 2-methyloxetan-4-yl and 2-methyloxetan-3-yl depending on the side to which O_2_ adds (Figure 3). For example, abstraction of the tertiary hydrogen atom by the peroxy group is restricted in *syn*-2-methyloxetanyl-4-peroxy while abstraction from the methyl group remains a viable ROȮ → Q̇OOH isomerization pathway.

The present work utilizes time-resolved photoionization mass spectrometry (MPIMS) measurements of product formation from the Cl-initiated oxidation of 2-methyloxetane along with potential energy surface calculations to provide clarity on reaction mechanisms from 2-methyloxetanyl radicals and related peroxy radicals, as depicted in Figure 2. In addition to products from ring-opening reactions of initial Ṙ radicals, the experiments reveal that Ṙ + O_2_ reactions of 2-methyloxetanyl are significant and may involve a pathway to chain-branching as in 2,4-dimethyloxetane-derived radicals.^21,22^ Emphasis is placed herein on connecting species detected in the experiments to specific Ṙ and Q̇OOH radical isomers, supported by the potential energy surface results, in order to provide detailed targets for combustion modeling.

## 2 Experimental and Computational Approach

The sections below provide detail of the multiplexed photoionization mass spectrometry (MPIMS) experiments (Section 2.1), photoionization cross-section measurements (Section 2.2), and potential energy surface computations (Section 2.3).

### 2.1 Experimental Method

MPIMS experiments were conducted at the Chemical Dynamics Beamline of the Advanced Light Source^38^ in a slow-flow quartz reactor^39^ with a mass resolution of *m*/Δ*m* ≈ 1500 using pulsed-photolytic chlorine atom-initiated oxidation with constant initial reactant number densities, diluted in He (Table 1). Two temperatures, 650 K and 800 K, were utilized at a pressure of 6 Torr. The selected temperatures correspond to peak yields of 2-methyloxetane in *n*-butane oxidation.^29^ The experimental procedure is similar to that in Rotavera et al.^24^

**Table 1:** Initial Number Densities of Reactants (cm^–3^) and Pressure Utilized for Cl-Atom-Initiated Oxidation Experiments^a^

pressure	[RH]_0_	[O_2_]_0_	[Ċl]_0_	[Cl_2_]_0_
6 Torr	7.5 × 10^13^	1.1 × 10^16^	5.4 × 10^12^	7.4 × 10^13^

a The [He] balance is not listed. Pseudo-first-order conditions were used: [RH]_0_:[Ċl]_0_ = 14 and [O_2_]_0_:[ Ṙ]_0_ = 2060.

Photolysis of Cl_2_, using unfocused 351 nm light from an excimer laser operating at 2.5 W, generated Ċl atoms instantaneously and homogeneously along both the radial and longitudinal axes of the reactor. The photoionization cross-section from Sander et al.^40^ (0.18 Mb) enabled calculation of the initial chlorine atom concentration, [Ċl]_0_. The Ċl atoms react via RH + Ċl → Ṙ + HCl to form the initial Ṙ radicals, which subsequently undergo pseudo-first-order reaction with O_2_. The initial radical distribution for the 1:2:3:4 sites (cf. Figure 2) is approximately 0.53:0.11:0.26:0.10 using structure–activity relations^41^ (S1a). Because of the pseudo-first-order conditions employed, [RH]_0_:[Ċl]_0_ = 14, the initial depletion of 2-methyloxetane is due to Ċl atoms being consumed by RH to produce [Ṙ]_0_ ≅ [Ċl]_0_. To reduce the potential for side chemistry unrelated to Ṙ + O_2_, the pseudo-first-order conditions employed for RH + Ċl ensured that Ṙ + Ċl reactions remain minimal, and the >10^3^ excess of [O_2_]_0_ relative to [Ċl]_0_ forces Ṙ + O_2_ to be the dominant reaction. Initial depletion of [RH]_0_ by Ċl is near 2% (S1b). Thermal decomposition of closed-shell 2-methyloxetane yields the product pair propene + formaldehyde or ethene + acetaldehyde. Minor ion signals were observed at *m*/*z* 28 and *m/z* 30 in the pre-photolysis region only at 800 K. Utilizing rate parameters from Zalotai et al.^36^ for pathways leading to both species, the rate of thermal decomposition at 800 K is minor (∼10^–2^ s^–1^); at 650 K, the rate for either of the two pathways is ∼10^–6^ s^–1^. In addition, ion signal is not detected at *m*/*z* 29 (HĊO), which is important given that post-photolysis may yield formyl from Ċl + formaldehyde → HCl + HĊO in a case where significant formaldehyde is formed pre-photolysis via thermal decomposition. In addition, no ion signal presented at *m*/*z* 43 (indicative of vinoxy, H_2_ĊCH(=O), or acetyl, H_3_CĊ=O, derived from Ċl + acetaldehyde).

The photoionization experiments were conducted over a photon energy range of 8.5–11.0 eV using 50-meV intervals. Products from the Ċl-initiated oxidation reactions exit the quartz reactor through a 600 μm side orifice into a detector region maintained at ∼10^–8^ Torr forming a near-effusive molecular beam, which is then collimated by a 1.5 mm-diameter skimmer positioned approximately 20 mm downstream from the side orifice. Cations, consisting of both parent and fragment ions, are formed by orthogonally intersecting the collimated molecular beam with quasi-continuous photons produced from tunable synchrotron radiation and then detected using an orthogonal-acceleration time-of-flight mass spectrometer equipped with microchannel plates.

### 2.2 Reference Measurements

In order to quantify species concentrations and assign isomeric contributions to the peaks in the mass spectra, absolute photoionization cross sections σ(*E*) were measured at 600 K for methyl vinyl ketone and 1-oxiran-2-yl-ethanone in separate, nonreactive experiments (S2). The σ(*E*) are defined relative to propene in accordance with the procedure described in Welz et al.^42^ Relative yields were calculated as the concentration of a species (e.g., 1,3-butadiene) relative to propene, formed via 2-methyloxetanyl decomposition, using least-squares fitting of the corresponding absolute photoionization spectra time-integrated 20 ms postphotolysis. The equation for quantifying the relative yield is derived in S3.

### 2.3 Potential Energy Surfaces

To support the experimental results, potential energy surfaces for each Ṙ and ROȮ radical were constructed using KinBot.^43^ Geometry optimizations and IRC calculations were performed with Gaussian 16. The coupled-cluster stationary point energies were computed using ORCA.^44^ The reaction search was conducted at the L0 = AM1 level of theory via a series of constrained optimizations according to specified reaction families. After refining the transition-state geometry at L1 = B3LYP/6-31+G, intrinsic reaction coordinate (IRC) calculations were performed at the same level of theory. The conformer search is also carried out at the L1 level of theory. The exact method for the conformer search is described in Doner et al.^21,22^ For the lowest conformer of each stationary point, including transition states, the geometries, frequencies, and the zero-point energy correction were obtained at the L2 = ωB97X-D/6-311++G(*d*,*p*) level of theory. Stationary point energies were then computed at the L3 = CCSD(T)-F12/cc-pVDZ-F12 level of theory. For each 2-methyloxetanyl radical, ring-opening pathways via C–C and C–O β-scission that exceeded a 30 kcal/mol barrier height threshold were neglected. C–H β-scission reactions of the initial 2-methyloxetanyl radicals, which are commonly higher in energy,^25^ exceeded the threshold and were neglected. All barrier heights for ring opening via C–O bond scission were less than 30 kcal/mol. For the 2-methyloxetanyl peroxy (ROȮ) radical surfaces, only stationary points along pathways with barriers submerged below the corresponding Ṙ + O_2_ energy were calculated. To save computational resources, a maximum of three elementary steps from each ROȮ were considered. Geometry coordinates of all optimized structures are compiled in S4. Adiabatic ionization energies were also calculated for several species (S5) using the composite CBS-QB3 method within Gaussian 16.^45^

## 3 Results

Potential energy surfaces for 2-methyloxetanyl → products (Section 3.1) and 2-methyloxetanylperoxy → products (Section 3.2) are summarized, and select reactions are discussed in the context of favorable pathways that produce species observed in the experiments. The 10 surfaces are in S6 along with stationary point energies. Relative yields were calculated by using photoionization spectra for species detected in the MPIMS experiments (Section 3.3). Reaction mechanisms are described that connect the detected species to specific Ṙ and/or ROȮ radicals of 2-methyloxetane.

### 3.1 Potential Energy Surfaces for 2-Methyloxetanyl → Products

**Figure 4:**
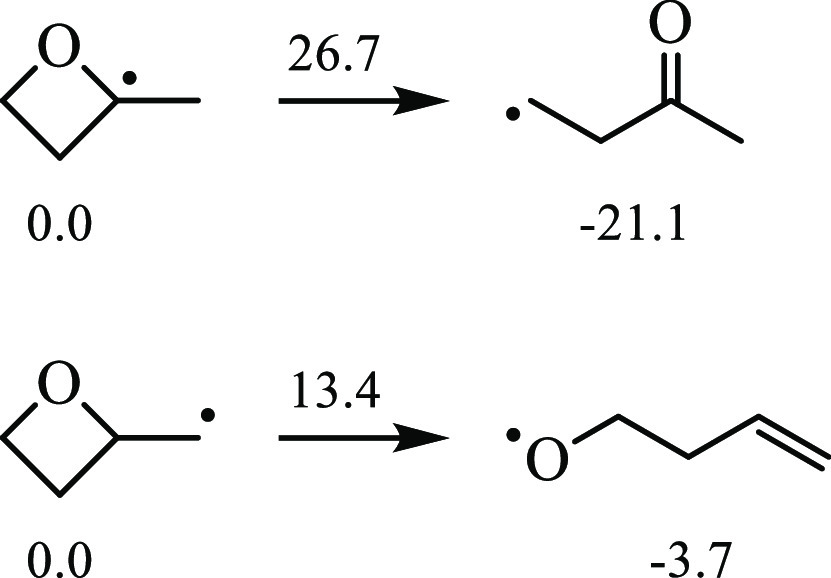
Stationary point energies and barrier heights, relative to radicals R3 and R4, for ring-opening reactions. The formation of unsaturated alkoxy radicals presents lower barriers than reactions forming carbonyl-substituted alkyl radicals. However, the latter type of reaction is significantly more exothermic. The geometries were optimized at ωB97X-D/6-311++G(*d*,*p*), and the stationary points were computed at CCSD(T)-F12/cc-pVDZ-F12; units: kcal/mol.

Results of potential energy surfaces are discussed below for reaction pathways of each 2-methyloxetanyl isomer, which typically undergo ring-opening, followed by β-scission into alkenes, carbonyls, and radicals. Ring-opening via C–O bond scission is consistently more energetically favorable than via C–C bond scission. However, for one case (R4), the barrier height for ring opening via C–C bond scission was only 15.2 kcal/mol. Species with the radical center separated from the ether group by one carbon (e.g., R2, R4) present the lowest barriers for ring opening. Radicals with the unpaired electron adjacent to the ether group undergo ring-opening reactions to form carbonyl-substituted alkyl radicals, which is exothermic. Figure 4 gives the barrier heights for the ring opening of R3 and R4 via C–O bond scission. The barrier height for ring-opening of the tertiary radical, R3, is approximately twice that of the primary radical, R4. However, ring-opening of R3 is 21.1 kcal/mol exothermic compared to only 3.7 kcal/mol for R4.

#### 3.1.1 2-methyloxetan-4-yl (R1)

**Figure 5:**
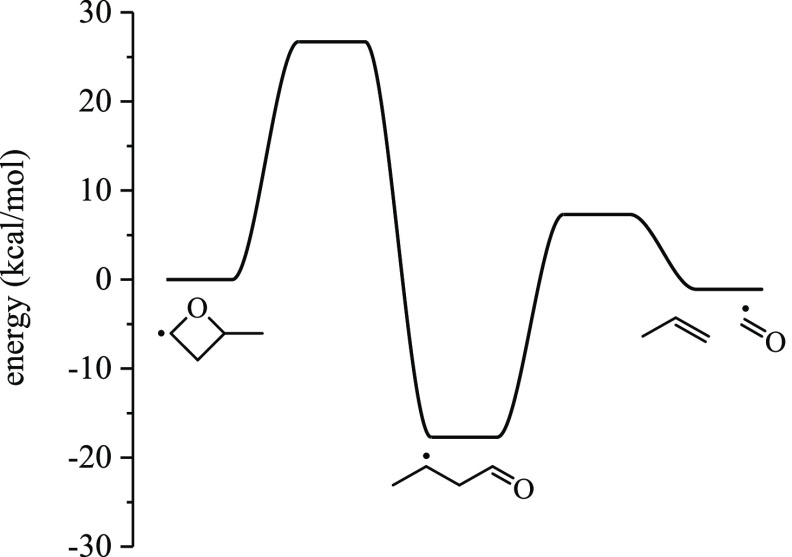
R1 undergoes exothermic ring opening to give butanal-3-yl, which leads to formyl and propene.

For R1, two pathways exist, yielding either propene + HĊO or acrolein + ĊH_3_. The lowest-energy pathway for R1 is given in Figure 5. R1 undergoes exothermic ring opening via C–O bond scission, giving butanal-3-yl which undergoes C–C β-scission to yield formyl and propene. Alternatively, an exothermic H-shift reaction from butanal-3-yl yielding 2-ethylvinoxy exists and involves a barrier height of 30.4 kcal/mol that is connected to a hydroxy-substituted resonance-stabilized radical (S6).

#### 3.1.2 2-methyloxetan-3-yl (R2)

**Figure 6:**
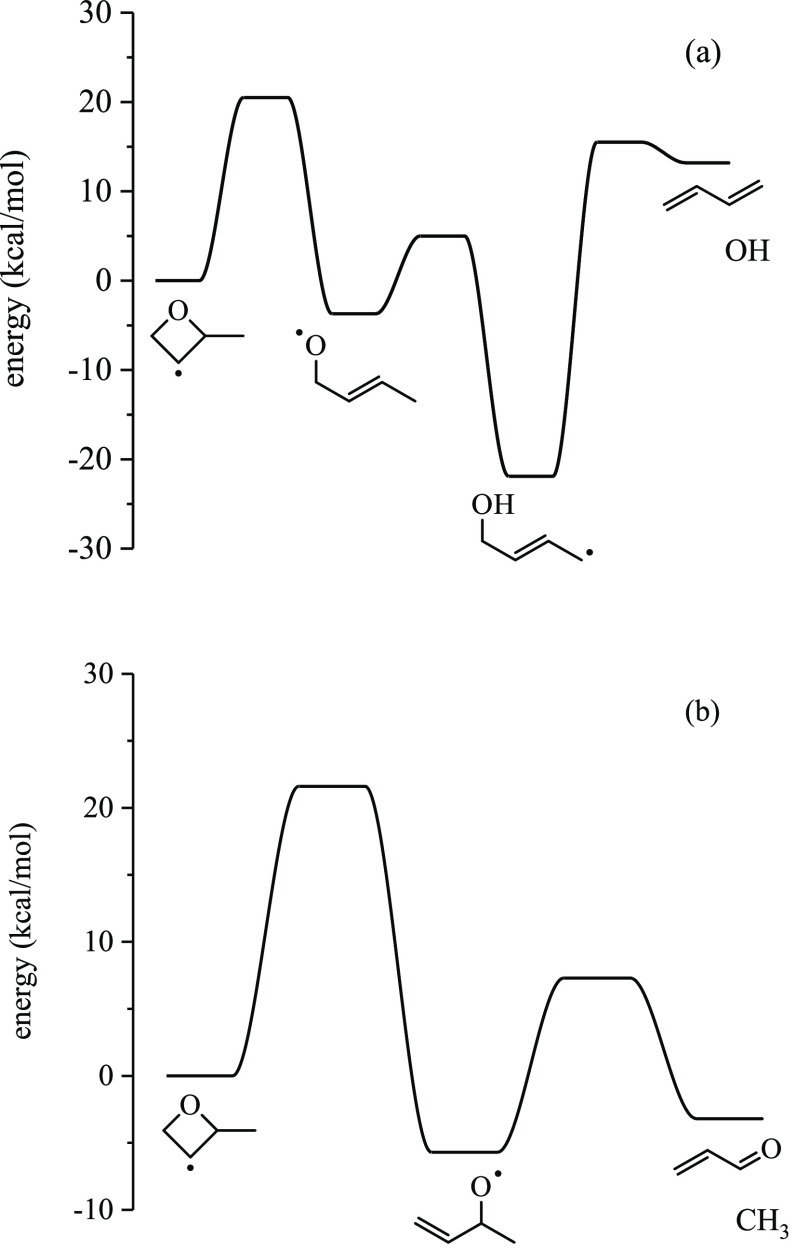
Low-energy pathways for the unimolecular decomposition of R2. (a) Pathway forming butadiene via ring opening, internal H-transfer, and β-scission. (b) Pathway forming acrolein via ring-opening and β-scission. The geometries were optimized at ωB97X-D/6-311++G(*d*,*p*), and the stationary points were computed at CCSD(T)-F12/cc-pVDZ-F12.

For R2, three pathways exist, yielding either acrolein + ĊH_3_, acetaldehyde + Ċ_2_H_3_, or butadiene + ȮH. The lowest-energy pathway for the unimolecular decomposition of R2 is given in Figure 6a. R2 undergoes ring-opening via scission of the C–O bond in the 3 position (20.5 kcal/mol barrier), giving but-2-en-3-oxy. Primary hydrogen is then transferred to the oxy group through a six-membered transition state with a barrier height of 8.7 kcal/mol, producing a resonance-stabilized but-2-en-1-ol-4-yl, which loses ȮH to yield butadiene. No other pathways from any of the Ṙ or ROȮ radicals to butadiene were identified. Another low-energy pathway yielding acrolein is given in Figure 6b. R2 undergoes ring opening via a 21.6 kcal/mol barrier followed by methyl loss over a 13.0 kcal/mol barrier that yields acrolein. Alternatively, a 1,2-H-shift over a 17.1 kcal/mol barrier forms 2-butanone-4-yl, which is also a ring-opening product of R3 and is 22.9 kcal/mol exothermic. A submerged pathway to methyl vinyl ketone + Ḣ also exists (S6).

#### 3.1.3 2-methyloxetan-2-yl (R3)

**Figure 7:**
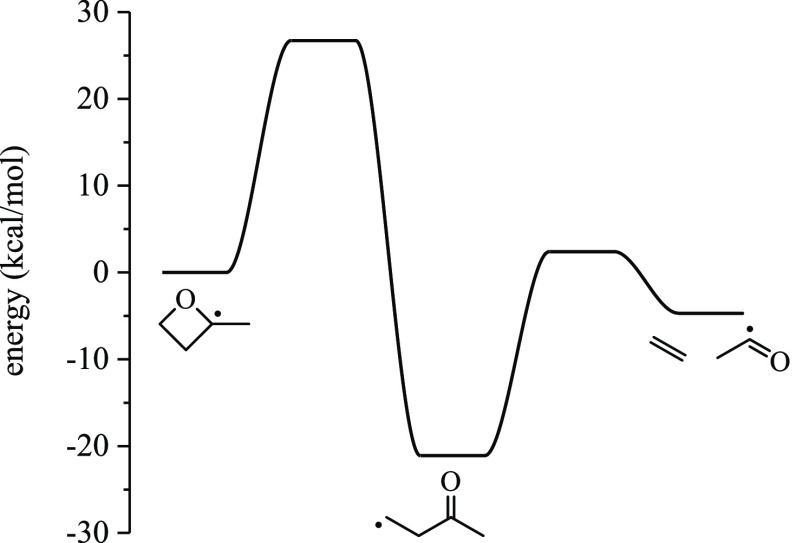
Ring opening of R3 via C–O bond scission forms 3-butanone-1-yl, which decomposes to acetyl + ethylene.

For R3, two pathways exist, yielding either ethene + H_3_CĊ=O or ketene + Ċ_2_H_5_. The latter pathway involves an intramolecular hydrogen shift that exceeds the energy criteria specified in KinBot. The lowest-energy pathway for R3 is ring-opening followed by β-scission yielding acetyl and ethylene (Figure 7). The 1,2-H-shift reaction forming but-1-en-3-oxy, which is also a ring-opening product of R2, encounters a barrier height of 40 kcal/mol.

#### 3.1.4 (2-ylomethyl)oxetane (R4)

**Figure 8:**
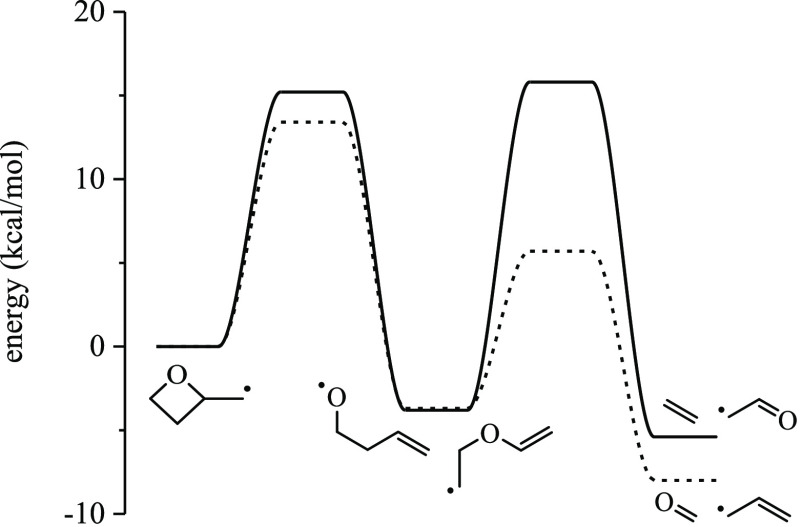
Low-energy pathways for ring opening of R4 via C–O and C–C bond scission. Each of the ring-opened intermediates decomposes via β-scission, giving ethylene and vinoxy or formaldehyde and allyl.

For R4, three pathways exist, leading to formaldehyde + allyl (Ċ_3_H_5_), ethene + H_2_ĊCH(=O), or CO + *n*-propyl. R4 may undergo two ring-opening pathways below the 30 kcal/mol threshold: one via C–O bond scission forming 1-butene-4-oxy and one via C–C bond scission forming 2-(vinyloxy)ethan-1-yl (Figure 8). Subsequent β-scission yields formaldehyde + allyl and ethylene + vinoxy, respectively. An alternative, lower-energy pathway exists for 2-(vinyloxy)ethan-1-yl in which the radical closes to form tetrahydrofuran-2-yl. Subsequently, tetrahydrofuran-2-yl undergoes ring opening, forming 1-butanal-4-yl that undergoes a 1,4-H shift to form 1-butanal-1-yl, which decomposes to form CO + *n*-propyl. The CO-elimination pathway may become significant at lower temperatures due to the involvement of hydrogen-shift reactions.

### 3.2 Potential Energy Surfaces for 2-Methyloxetanylperoxy → Products

The present section summarizes the potential energy surfaces for each 2-methyloxetanylperoxy radical. The ROȮ well depth varies depending on the position of the peroxy group. ROO3, which is a tertiary peroxy radical, forms the deepest well (35.9 kcal/mol) relative to the R3 + O_2_ entrance channel. ROO4 (primary peroxy radical) forms the shallowest well (25.3 kcal/mol) relative to the R4 + O_2_ entrance channel. ROȮ → Q̇OOH pathways that involve six- or seven-membered transition states are favored due to increased ring strain of the oxetane group imposed on other isomerization reactions.

**Figure 9:**
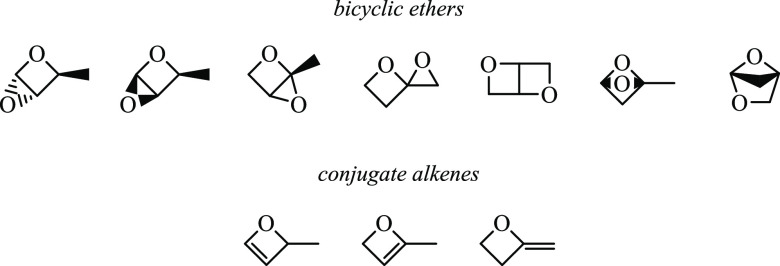
Intermediates from Ṙ + O_2_ reactions of 2-methyloxetane: bicyclic ethers (*m*/*z* 86) and conjugate alkenes (*m*/*z* 70) for which barrier heights were calculated.

Q̇OOH species undergo either bicyclic ether formation, HOO elimination, or ring-opening reactions. Constitutional isomers of bicyclic ethers and conjugate alkenes for which pathways were identified are given in Figure 9. Ring-opening reactions for Q̇OOH species follow the same patterns described for Ṙ discussed above. In cases where ring-opening of Q̇OOH results in an α-Q̇OOH, OH is lost in the same step, leaving behind a carbonyl group. Such reactions are highly exothermic: ∼50 kcal/mol relative to the Q̇OOH well depth and ∼80 kcal/mol relative to the Ṙ + O_2_ entrance channel.^22^ Other ring-opened Q̇OOH species undergo stepwise β-scission, yielding a variety of products, including cyclic ethers, organic radicals and acids, and HOȮ.

#### 3.2.1 2-methyloxetanyl-4-peroxy (ROO1)

Both *anti*- and *syn*-ROO1 involve pathways yielding 2-methyl-2*H*-oxete, 3-methyl-2,5-dioxabicyclo[2.1.0]pentane, 3-hydroperoxyacrylaldehyde and ĊH_3_, 3-methyloxirane-2-carbaldehyde, performic acid and 2-methylformyl, and 2-butenal. *anti*-ROO1 traverses a unique pathway to 3-oxobutanal while decomposition pathways of *syn*-ROO1 can lead to performic acid + allyl, vinyl ether + ȮH, 2,5-dioxabicyclo[2.1.1]hexane + ȮH, and 2-(vinyloxy)oxirane + ȮH, among a total of 17 pathways.

**Figure 10:**
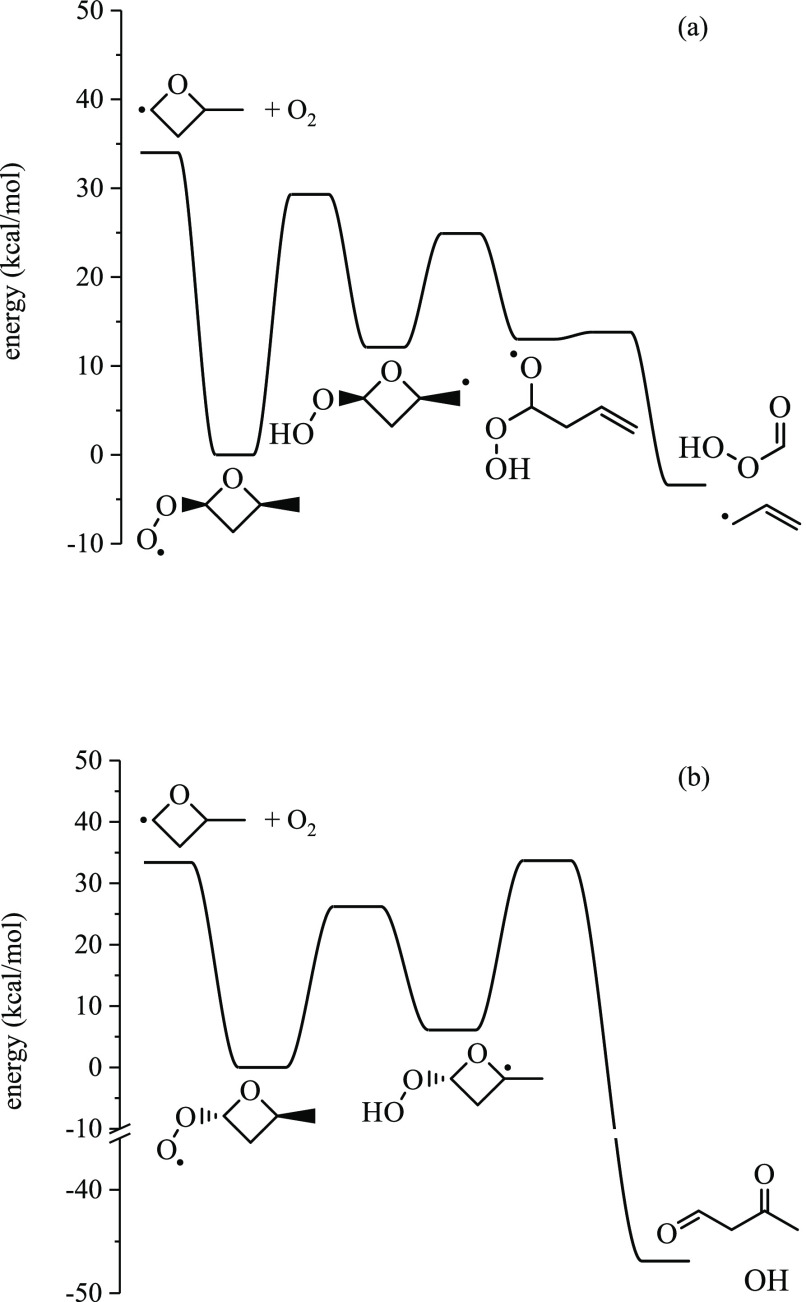
Lowest-energy pathways for (a) *syn*-ROO1 and (b) *anti*-ROO1.

The ROO1 radical, 2-methyloxetanyl-4-peroxy, may form two diastereomers depending on the side of the ring to which O_2_ adds, which differ in the relative positions of the peroxy group and the methyl group on the oxetane ring. The lowest-energy pathway for each diastereomer is given in Figure 10. For *syn*-ROO1 (Figure 10a), the peroxy group abstracts H from the methyl group, which is on the same side of the oxetane ring. The Q̇OOH produced, QOOH14, undergoes ring opening via C–O bond scission, yielding 1-hydroperoxy-1-oxybutan-3-ene, which undergoes β-scission over a 1−2 kcal/mol barrier to performic acid + allyl. Performic acid is a ketohydroperoxide and may contribute to chain branching. However, the most abundant organic peroxide in the atmosphere,^46^ performic acid decomposition mechanisms, and reaction rates under combustion-relevant conditions (higher temperatures and pressures) are unknown. An analogous pathway from 2,4-dimethyloxetanylperoxy radicals produces peracetic acid with appreciable branching fractions between 300 K and 1000 K at 1 atm,^22^ providing an indication that the channel is of potential importance in the oxidation of 2-methyloxetane and, more broadly, to *n*-butane combustion.

**Figure 11:**
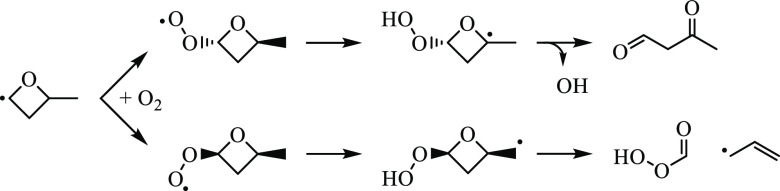
When O_2_ is added to R1, two diastereomers may form: *anti*-ROO1 and *syn*-ROO1. The stereochemistry of ROO1 determines which QOOH pathways are possible. Only *anti*-ROO1 can form QOOH13, and only *syn*-ROO1 can form QOOH14.

For *anti*-ROO1, the peroxy group abstracts hydrogen from the tertiary carbon, giving QOOH13, which can undergo ring opening via C–O bond scission with a barrier of 27.6 kcal/mol, giving 3-oxobutanal formed with ȮH in a concerted step from an α-Q̇OOH. The reaction is 53 kcal/mol exothermic relative to QOOH13 and ∼80 kcal/mol exothermic relative to the Ṙ + O_2_ entrance channel. Figure 11 shows stereoisomer-specific pathways of *syn*- and *anti*-ROO1 undergoing ring-opening reactions producing 3-oxobutanal and performic acid + allyl, respectively. Both *anti*- and *syn-*ROO1 encounter barrier heights to *anti*-QOOH12 (33.4 kcal/mol) and *syn*-QOOH12 (33.9 kcal/mol) approximately equal in energy to the R1 + O_2_ entrance channel. The lowest-energy pathway for both *anti*- and *syn-*QOOH12 is the formation of the bicyclic ether, 3-methyl-2,5-dioxabicyclo[2.1.0]pentane. Alternatively, two ring-opening pathways for each diastereomer exist. For *syn*-QOOH12, the ring-opening barrier heights are 20.8 and 21.8 kcal/mol. For *anti*-QOOH12, the ring-opening barrier heights are 20.0 and 21.7 kcal/mol. Neither pathway is submerged.

#### 3.2.2 2-methyloxetanyl-3-peroxy (ROO2)

Both *anti*-ROO2 and *syn*-ROO2 involve pathways to 2-methyl-2*H*-oxete, 3-methyl-2,5-dioxabicyclo[2.1.0]pentane, 2-butenal, 3-methyloxirane-2-carbaldehyde, and 2-methylmalonaldehyde. The surface for the *anti*-diastereomer contains unique pathways to 4-methyl-2*H*-oxete, 1-methyl-2,5-dioxabicyclo[2.1.0]pentane, 1-(oxiran-2-yl)ethan-1-one, and methyl vinyl ketone. The surface for the *syn*-diastereomer involves unique pathways to acrolein + formaldehyde, 2-(vinyloxy)acetaldehyde, and 2,5-dioxabicyclo[2.2.0]hexane. In total, 18 pathways were identified.

**Figure 12:**
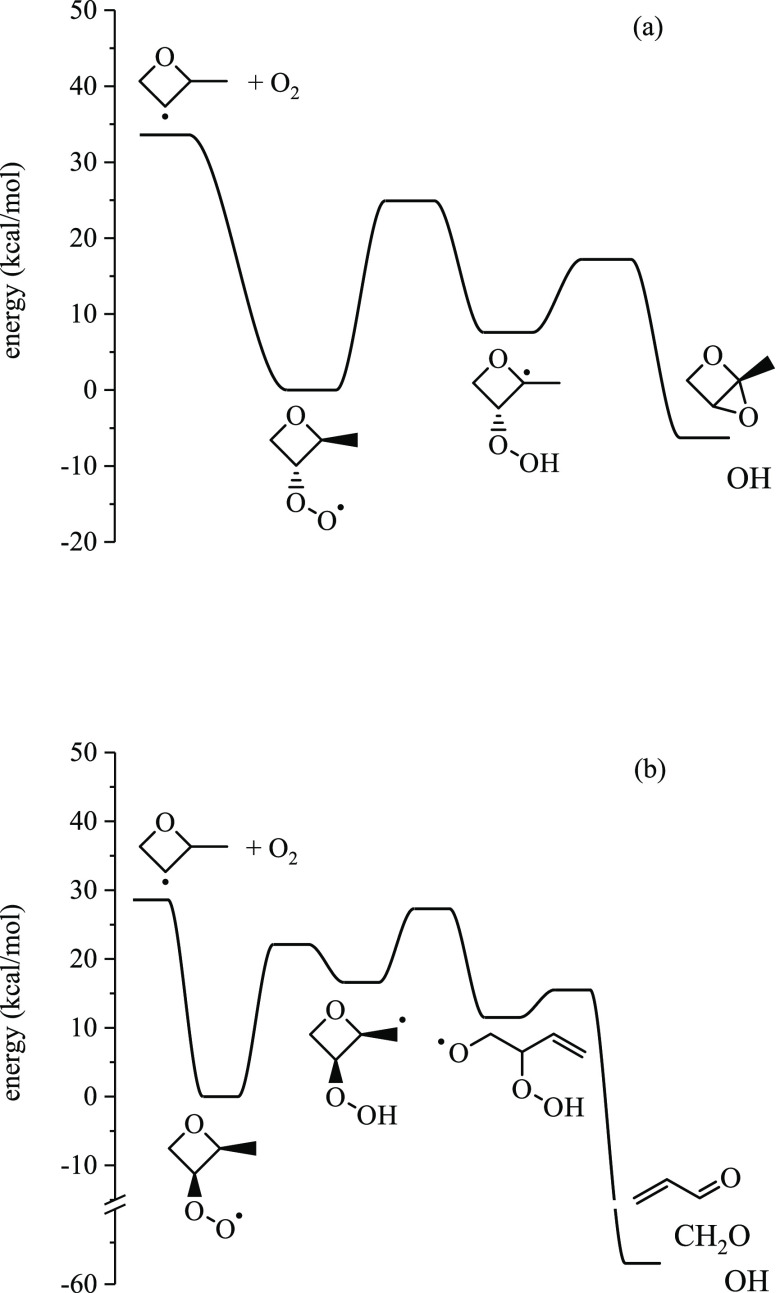
Lowest-energy pathways for *anti*-ROO2 (a) and *syn*-ROO2 (b), forming the bicyclic ether 1-methyl-2,5-dioxabicyclo[2.1.0]pentane and acrolein, respectively.

The lowest-energy pathway for each ROO2 diastereomer is given in Figure 12. For *anti*-ROO2, the lowest-energy pathway is intramolecular H-abstraction from the tertiary carbon, forming QOOH23, over a 24.9 kcal/mol barrier. The lowest-energy pathway for QOOH23 is bicyclic ether formation over a 9.6 kcal/mol barrier. For *syn*-ROO2, the lowest-energy pathway forms *syn*-QOOH24 over a 22.1 kcal/mol barrier, which involves a 10.7 kcal/mol barrier for ring opening, yielding 2-hydroperoxy-1-oxy-but-3-ene. Subsequent β-scission of 2-hydroperoxy-1-oxy-but-3-ene forms acrolein + formaldehyde + ȮH over a 4.0 kcal/mol barrier.

The barrier heights from *anti*-ROO2 and *syn*-ROO2 to QOOH21 are 26.8 and 26.6 kcal/mol barrier, respectively, which are both submerged below R2 + O_2_. The lowest barrier for *anti*- and *syn*-QOOH21 is bicyclic ether formation (11.6 and 17.6 kcal/mol, respectively), yielding 3-methyl-2,5-dioxabicyclo[2.1.0]pentane. HOO elimination from *anti*-QOOH21 encounters a barrier height of 20.3 kcal/mol, and HOO elimination from *syn*-QOOH21 encounters a barrier height of 18.7 kcal/mol at the L2 level of theory, both of which are submerged below R2 + O_2_. The barrier heights for the ring opening of *anti*-QOOH21 and *syn*-QOOH21 are 25.3 and 25.2 kcal/mol (L2), respectively. Neither pathway is submerged below R2 + O_2_.

The lowest-energy pathway for the isomerization of *anti*-ROO2 is the formation of QOOH23 via internal H abstraction from the tertiary carbon, followed by decomposition into bicyclic ether, 1-methyl-2,5-dioxabicyclo[2.1.0]pentane. For *syn*-ROO2, the lowest barrier to QOOH is internal H abstraction from the methyl group, forming QOOH24. QOOH24 undergoes ring opening via C–O bond scission, giving 2-hydroperoxy-1-oxy-3-butene, which decomposes into acrolein + formaldehyde + ȮH. The MPIMS experiments detected acrolein (*vide infra*), which is a product of both Ṙ and ROȮ decomposition. Bahrini et al.^47^ reported discrepancies in the predictions of measured acrolein concentrations in jet-stirred reactor experiments on *n*-butane oxidation. The addition of previously excluded pathways forming acrolein to chemical kinetic models may ameliorate the disagreement by reducing the mechanism truncation error.

#### 3.2.3 2-methyloxetanyl-2-peroxy (ROO3)

**Figure 13:**
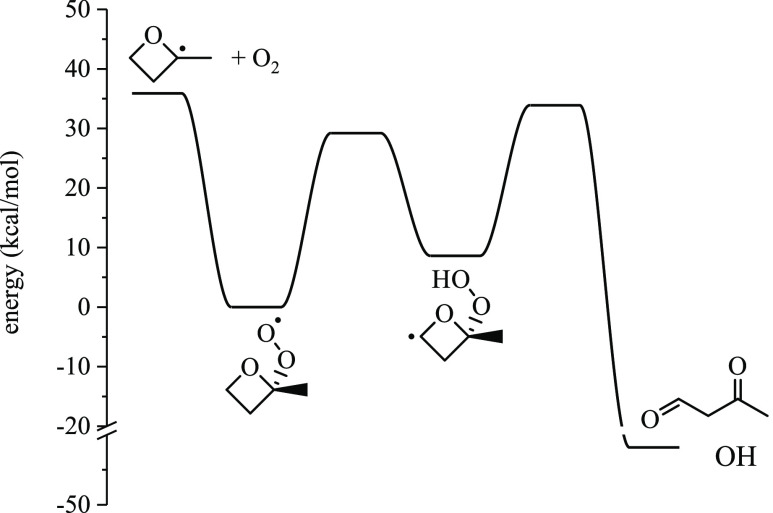
Lowest-energy pathway for ROO3 produces 3-oxobutanal via ring opening and OH loss from QOOH31, which is 50.4 kcal/mol exothermic relative to the corresponding Q̇OOH.

For ROO3, four pathways exist, yielding either 4-methyl-2*H*-oxete + ȮH, 2-methyleneoxetane + ȮH, 1-methyl-2,5-dioxabicyclo[2.1.0]pentane + ȮH, or 3-oxobutanal + ȮH. The lowest-energy pathway for ROO3, 2-methyloxetanyl-3-peroxy, is given in Figure 13. The peroxy group abstracts H from the secondary carbon adjacent to the ether group (the 1 position, cf. Figure 2) over a 29.2 kcal/mol barrier, giving QOOH31. QOOH31 undergoes ring opening via C–O bond scission concerted with OH loss over a 25.3 kcal/mol barrier, similar to QOOH13 (cf. Section 3.2.1). The product is also 3-oxobutanal, which is 50.4 kcal/mol exothermic relative to Q̇OOH. The barrier height for the formation of QOOH32 (34.8 kcal/mol) is also submerged below the R3 + O_2_ entrance channel. QOOH32 can undergo bicyclic ether formation over a 15.2 kcal/mol barrier or HOO elimination over a 20.7 kcal/mol barrier. Neither ring-opening reaction for QOOH32 is submerged below the entrance channel.

#### 3.2.4 2-(methylperoxy)oxetane (ROO4)

**Figure 14:**
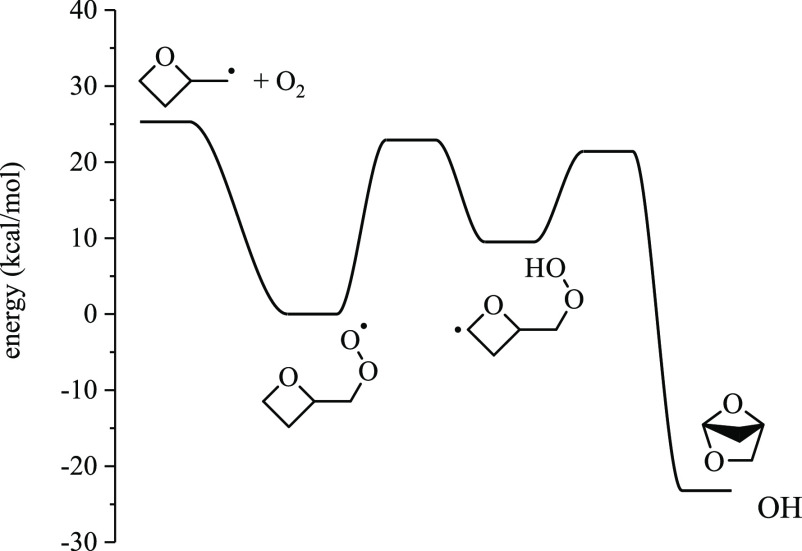
Lowest-energy pathway for ROO4 is internal H-abstraction via a seven-membered transition state followed by bicyclic ether formation, yielding 2,5-dioxabicyclo[2.1.1]hexane.

For ROO4, four pathways exist, yielding either 2-methyleneoxetane + HOȮ, 1,4-dioxaspiro[2.3]hexane + ȮH, 2,5-dioxabicyclo[2.1.1]hexane + ȮH, or 2,5-dioxabicyclo[2.2.0]hexane + ȮH. The lowest-energy pathway for 2-(methylperoxy)oxetane (ROO4) is internal H-abstraction from the 1 position via the seven-membered transition state, forming QOOH41 via a 22.9 kcal/mol barrier. The lowest-energy barrier for QOOH41 is bicyclic ether formation, yielding 2,5-dioxabicyclo[2.1.1]hexane + ȮH over a barrier of 11.9 kcal/mol (Figure 14). The ring-opening pathway forming 4-hydroperoxybutanal-3-yl has a barrier of 25.8 kcal/mol, which is 10 kcal/mol above the R4 + O_2_ entrance channel. Similarly, the barrier height for the ring opening of QOOH43 is 24.3 kcal/mol, which is 7.9 kcal/mol above the entrance channel.

### 3.3 Product Species from MPIMS Experiments

**Figure 15:**
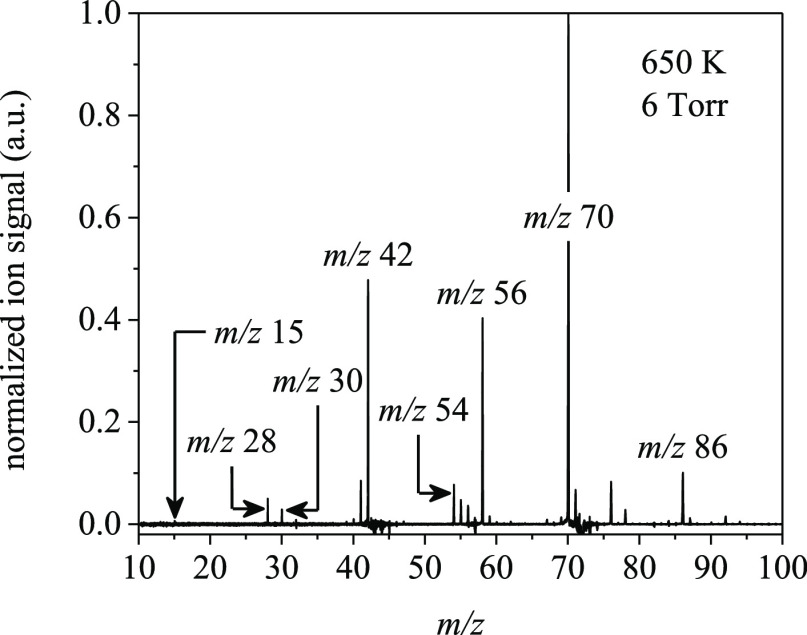
Difference mass spectra from the Cl-initiated oxidation of 2-methyloxetane at 650 K and 6 Torr, integrated 30-ms post-photolysis over the photon energy range of 8.5–11.0 eV.

The difference mass spectra in Figure 15, measured from the Cl-initiated oxidation of 2-methyloxetane at 650 K and 6 Torr, highlight the mass peaks considered herein. The negative signal at *m*/*z* 72 is produced from 2-methyloxetane [C_4_H_8_O]^+^ and related fragment ions and results from background subtraction 20-ms pre-photolysis. Fragment ions from 2-methyloxetane are produced at *m*/*z* 43, *m/z* 44, *m/z* 54, and *m/z* 57. Calibration of the mass spectra was performed using the exact mass for ethene, propene, and 1-butene to obtain coefficients that convert time-of-flight to mass. Similar mass peaks as in Figure 15, although in different proportions, were detected at 800 K (S7).

The sections below describe unique products ascribed either to Ṙ (Section 3.3.1) or to Ṙ + O_2_ reactions (Section 3.3.2). Intermediates formed via the reactions in Figure 2 were quantified directly by using photoionization spectra fitting (Table 2). The formation of RCl at *m*/*z* 106 via chain chlorination precluded the determination of branching fractions (i.e., product concentrations relative to initial radical concentration) because an unknown portion of the initial 2-methyloxetanyl is consumed by Cl_2_ rather than by O_2_. Instead, to insulate species quantification from chlorine-driven reactions, relative yields in Table 2 are defined relative to propene at 650 K. For example, the nominal methylperoxy yield was 3% with respect to that of propene at 650 K and undetected at 800 K; 1,3-butadiene yields at 650 and 800 K are 11 and 30%, respectively. The formation of methylperoxy (*m*/*z* 47) arises from the conversion of methyl due to the high concentration of O_2_ used in the experiments, and relative yields were quantified using σ(10.65 eV) = 3.5 Mb (S8). Other species were identified using either photoionization spectral analysis or adiabatic ionization energies (S9). For all relevant cases, the contribution to the ion signal from the corresponding ^13^C isotope peaks was subtracted prior to fitting to ensure accuracy in quantifying the spectra.

**Table 2:**
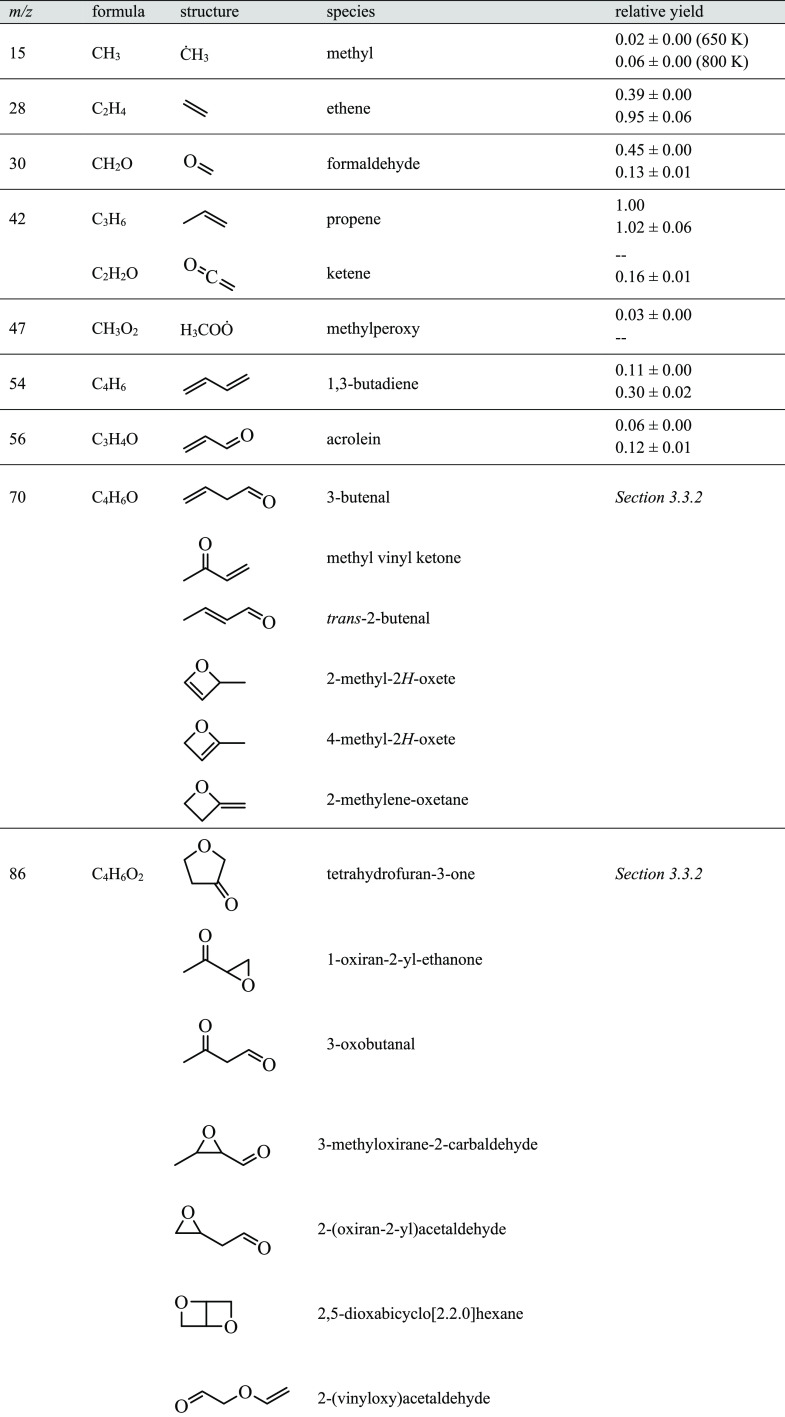
Nominal Masses, Molecular Formulas, and Relative Yields at 6 Torr Relative to Propene Yield at 650 K^a^

a Listed uncertainties include 1σ statistical errors in the fit of the photoionization spectra.

**Figure 16:**
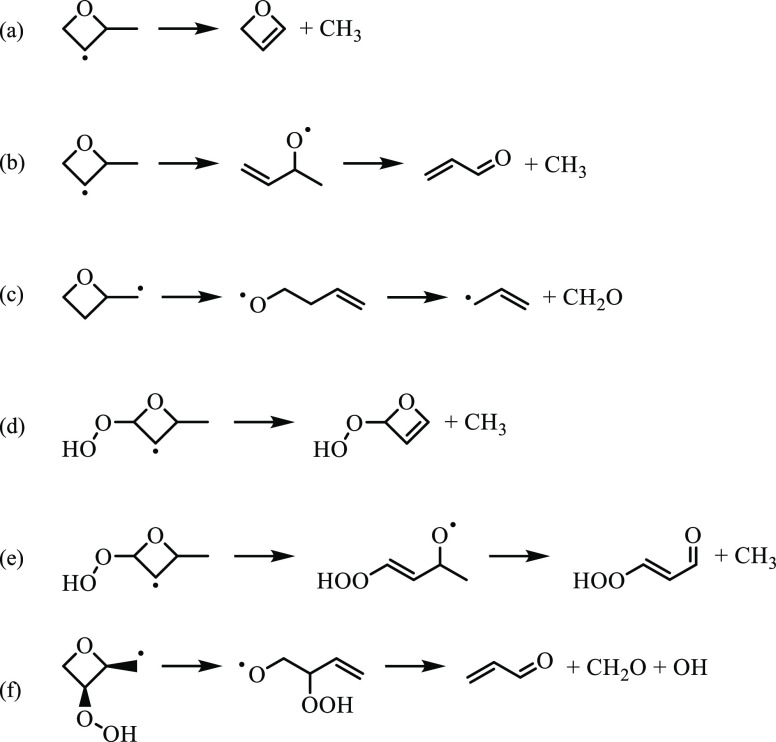
Reaction mechanisms leading to ĊH_3_, formaldehyde, and acrolein from 2-methyloxetanyl and 2-methyloxetanylperoxy radicals.

Of the twenty species in Table 2, three are products of unimolecular reactions of both 2-methyloxetanyl and 2-methyloxetanylperoxy radicals: ĊH_3_, formaldehyde, and acrolein. Relative yields of ĊH_3_ and acrolein increased by factors of 3 and 2 from 650 K to 800 K, respectively, while that of formaldehyde decreased by a factor of ∼3.5 ascribed to an increase in the rate of reaction of R4 with O_2_. Reaction mechanisms for each species are shown in Figure 16. β-Scission of R2 yields oxetene + ĊH_3_, which is neglected on the R2 surface (Figure 6) because the barrier is 35.5 kcal/mol at the L1 level of theory. In addition, oxetene (*m*/*z* 56) was not detected in the experiments because the adiabatic ionization energy (9.01 eV) is below the onset energy (∼10 eV) of the ion signal measured at *m*/*z* 56. R2 can also undergo ring-opening and subsequent β-scission to form acrolein + ĊH_3_. R4 can undergo a similar reaction sequence to yield formaldehyde + propenyl, which is also energetically accessible (Figure 8). Moreover, ĊH_3_ can form as a result of β-scission from *syn*-/*anti*-QOOH12 (S6.11). However, the surface in Figure 8 neglects the pathway because the barrier of the preceding ROȮ → Q̇OOH isomerization step exceeds the Ṙ + O_2_ entrance channel (S6.5 and S6.6). In addition, the ion signal at the nominal mass (*m*/*z* 88) of the coproduct, 3-hydroperoxyacrylaldehyde, was not detected in the experiments. Another pathway producing ĊH_3_, in addition to 3-hydroperoxy-2-propenal, is ring-opening of QOOH12. In Figure 16f, formaldehyde and acrolein form coincident with ȮH via ring-opening of *syn*-QOOH24 and subsequent β-scission and are the least energetically accessible (by ∼3 kcal/mol). The pathway is also in competition with cyclic ether formation and concerted β-scission and ȮH elimination.

#### 3.3.1 Products from the Unimolecular Reaction of 2-Methyloxetanyl Radicals

**Figure 17:**
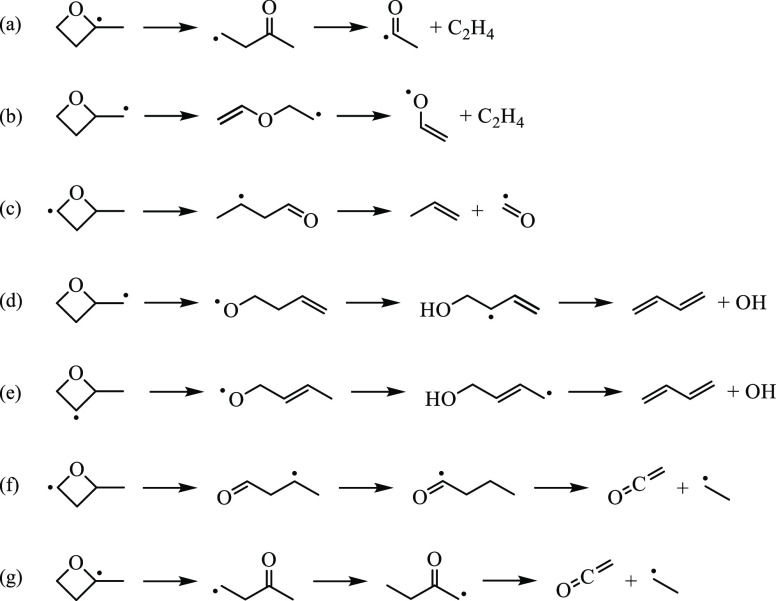
Reaction mechanisms leading to ethene, propene, 1,3-butadiene, and ketene from 2-methyloxetanyl radicals.

Several species arise exclusively from the unimolecular decomposition of 2-methyloxetanyl radicals: ethene, propene, ketene, and 1,3-butadiene. Relative yields for ethene and 1,3-butadiene increased with temperature, while propene remained nearly constant within experimental uncertainty and ketene was detected only at 800 K. Reaction mechanisms leading to all four species are shown in Figure 17. The production of ethene occurs as a result of ring-opening and subsequent β-scission of R3 and R4. The barrier to the former is energetically accessible (cf. Figure 7), and the products are submerged below the entrance channel. On the R4 surface (cf. Figure 8), however, the pathway leading to formaldehyde + allyl is energetically favored to that forming vinoxy + ethene. The ion signal at *m*/*z* 43 (vinoxy) was not detected under the conditions of the experiments potentially due to rapid consumption by O_2_ yielding formaldehyde. Another pathway to formaldehyde involves oxidation followed by β-hydrogen abstraction and the subsequent loss of CO, which in Weidman et al.^48^ was reported as the lowest-energy pathway for vinoxy + O_2_. Ring-opening and subsequent β-scission of R1 yields propene. The corresponding surface (cf., Figure 5) shows that the pathway is energetically accessible. The ion signal at the mass-to-charge ratio of the coproduct, formyl (*m*/*z* 29), was not detected in the experiments likely due to facile oxidation into CO.^49^ Another source of propene is propyl + O_2_,^50^ yet no direct pathway to propyl from 2-methyloxetanyl radicals exists. However, the reaction sequence 2-methyloxetany-4-yl → butanal-3-yl → butanal-1-yl → *n*-propyl + CO provides an indirect pathway.

**Figure 18:**
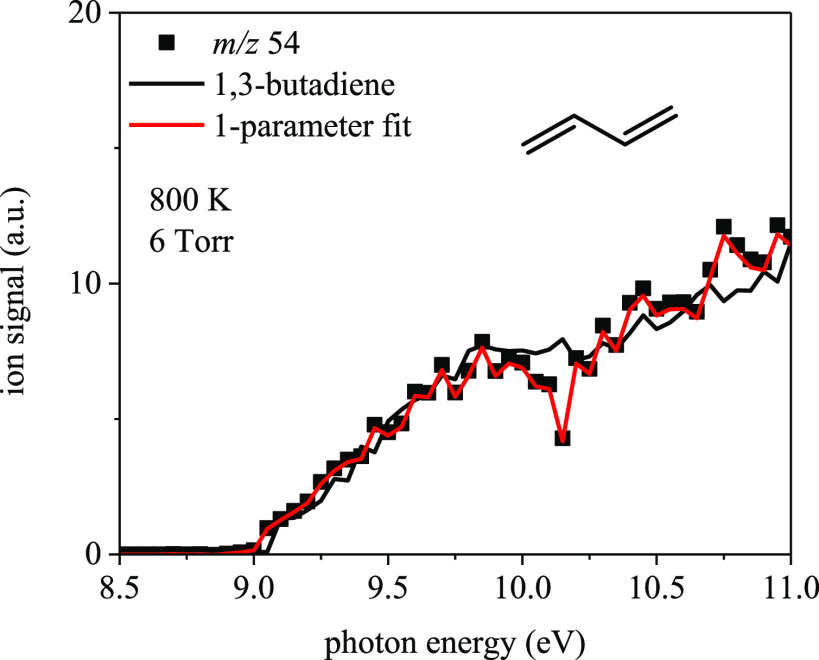
Photoionization spectrum of the *m*/*z* 54 ion signal from Cl-initiated oxidation of 2-methyloxetane at 6 Torr and 800 K overlaid with the spectrum for 1,3-butadiene.

Ion signal at *m/z* 54 was also detected in the experiments and confirmed as 1,3-butadiene (Figure 18) which forms via two potential pathways: ring-opening of R2 and/or R4 (Figure 17). Following ring-opening of R2, an H-shift occurs via a six-membered transition state where the oxy group abstracts a hydrogen from the primary carbon with a barrier of 5 kcal/mol. Subsequently, concerted β-scission and OH elimination results in 1,3-butadiene. Meanwhile, the 1,3-H-shift follows ring-opening of R4 via a four-membered transition state, after which OH-elimination occurs to form 1,3-butadiene.

Similar to 1,3-butadiene, ketene may form via ring-opening of R1 and R3, followed by a 1,4-H-shift and 1,5-H-shift, respectively (Figure 17). In both cases, β-scission results in ketene + ethyl, the later of which provides an additional source of ethene.^51^ For both R1 and R3, barriers for the hydrogen shifts exceed ∼30 kcal/mol. Moreover, the barrier on the R1 surface for butanal-1-yl → ketene + ethyl is 40.4 kcal/mol, and the reaction is in competition with CO elimination, which can readily occur from butanal-1-yl. While rate coefficients for butanal-1-yl → CO + *n*-propyl are not reported, chemical kinetics mechanisms of butanal^52^ use the analogous rate for propanal. The range of rate coefficients in the literature is on the order of 10^6^–10^9^ s^–1^ at 650 K and 10^7^–10^10^ s^–1^ at 800 K.^53^

#### 3.3.2 Products from the Unimolecular Reaction of the 2-Methyloxetanylperoxy Radicals

**Figure 19:**
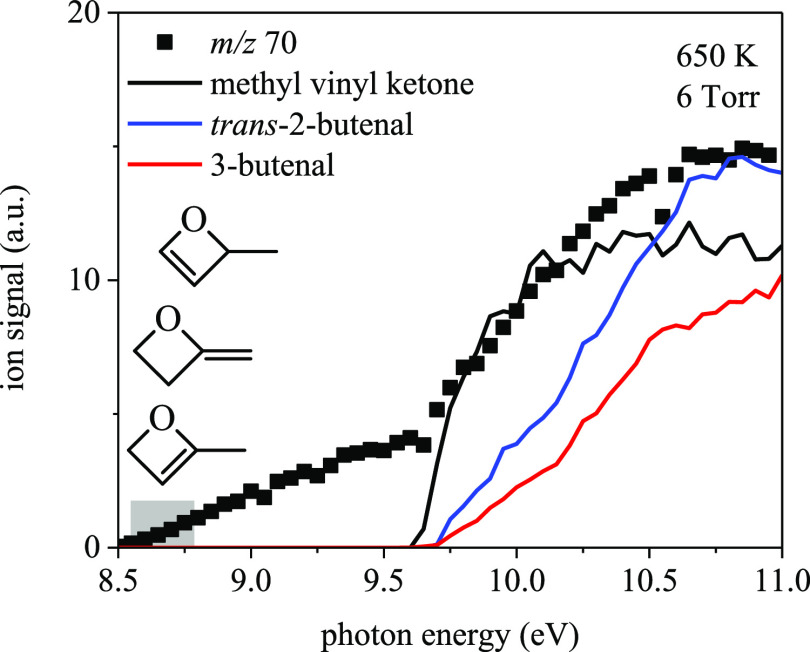
*m*/*z* 70 ion signal measured at 6 Torr and 650 K integrated for 30 ms postphotolysis compared against the photoionization spectrum of methyl vinyl ketone, *trans*-2-butenal,^54^ and 3-butenal.^10^ Ion signals below ∼9.5 eV may arise from 2-methyl-2*H*-oxete (8.77 eV), 4-methyl-2*H*-oxete (8.55 eV), and 2-methylene-oxetane (8.57 eV) formed via loss of the OH from 2-methyloxetanylperoxy radicals.

**Figure 20:**
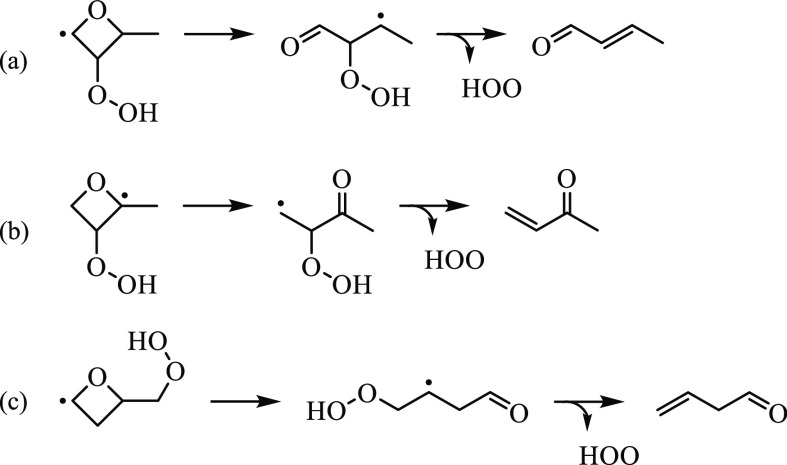
Reaction mechanisms for 3-butenal, methyl vinyl ketone, and *trans*-2-butenal via HOO-elimination from ketohydroperoxide-type radicals.

The mass peaks at *m*/*z* 70 and *m/z* 86 in Figure 15 arise from Ṙ + O_2_ and unimolecular reactions of 2-methyloxetanylperoxy radicals. Figure 19 shows the measured ion signal at *m*/*z* 70, where a gradual rise in ion signal begins at 8.5 eV and is followed by a sharper rise at ∼9.6 eV. Based on the potential energy surfaces in Sections 3.1 and 3.2, several species likely contribute to the sharp increase in signal: methyl vinyl ketone, 2-butenal, and 3-butenal. Reaction mechanisms of each species via OH-elimination of ketohydroperoxide-type radicals are shown in Figure 20. While 3-butenal and methyl vinyl ketone are produced from ring-opening of QOOH41 and QOOH23, respectively, the barriers along the pathways for both reactions exceed 30 kcal/mol and were neglected by KinBot. The barrier for the ring-opening of QOOH21, leading to 2-hydroperoxy-butanal-3-yl, in contrast, is energetically accessible. KinBot identified the subsequent unimolecular reaction of 2-hydroperoxy-butanal-3-yl and calculated a barrier height ∼2 kcal/mol higher than that for the formation of 2-methylpropanedial (S6). HOȮ elimination from ketohydroperoxide-type radicals derived from 2-methyloxetane yields vinyl ether, yet no contribution is attributed to the *m*/*z* 70 ion signal because the shape of the reference photoionization cross-section does not align under the conditions of the experiment.

The photoionization spectra for the acyclic species in Figure 19 do not explain the rise in ion signal at *m*/*z* 70 from 8.5 eV to 9.6 eV, which may result from HOO-elimination species 2-methyl-2*H*-oxete, 4-methyl-2*H*-oxete, or 2-methylene-oxetane (adiabatic ionization energies of 8.77 eV, 8.55 eV, and 8.57 eV, respectively). Barrier heights to direct HOO elimination from 2-methyloxetanylperoxy range from 30 to 38 kcal/mol (S6). Barriers to sequential, Q̇OOH-mediated reactions proceed via *syn*-/*anti*-QOOH12 and *anti*-QOOH21 for 2-methyl-2*H*-oxete and QOOH23 for 4-methyl-2*H*-oxete and are 25–27 kcal/mol (S6).

**Figure 21:**
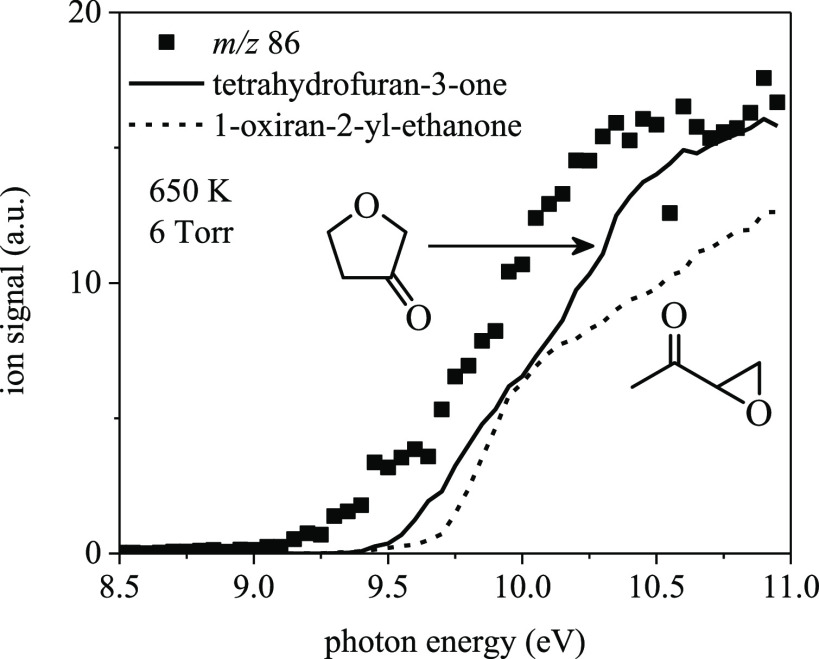
*m*/*z* 86 ion signal measured at 6 Torr and 650 K integrated for 30-ms post-photolysis compared against the photoionization spectrum of tetrahydrofuran-3-one and 1-oxiran-2-ylethanone. Several other species may also contribute to the *m*/*z* 86 ion signal above ∼9.0 eV: 2-(vinyloxy)acetaldehyde (9.14 eV), 2,5-dioxabicyclo[2.2.0]hexane (9.34 eV), 3-oxobutanal (9.54 eV), 2-oxiran-2-yl-acetaldehyde (9.88 eV), 2-methylpropanedial (9.91 eV), and 3-methyloxirane-2-carbaldehyde (9.96 eV).

Figure 21 shows species that may contribute to the ion signal at *m*/*z* 86. The onset energy for the experimental signal is ∼9.2 eV, while those of tetrahydrofuran-3-one and 1-oxiran-2-yl-ethanone are ∼9.5 eV and 9.7 eV, respectively. However, neither species is exclusively responsible for the total ion signal above ∼9.5 eV. To aid in species assignment, adiabatic ionization energies were calculated for several other species that may contribute to the ion signal at *m*/*z* 86: 2-(vinyloxy)acetaldehyde (9.14 eV), 2,5-dioxabicyclo[2.2.0]hexane (9.34 eV), 3-oxobutanal (9.54 eV), 2-oxiran-2-yl-acetaldehyde (9.88 eV), 2-methylpropanedial (9.91 eV), and 3-methyloxirane-2-carbaldehyde (9.96 eV).

**Figure 22:**
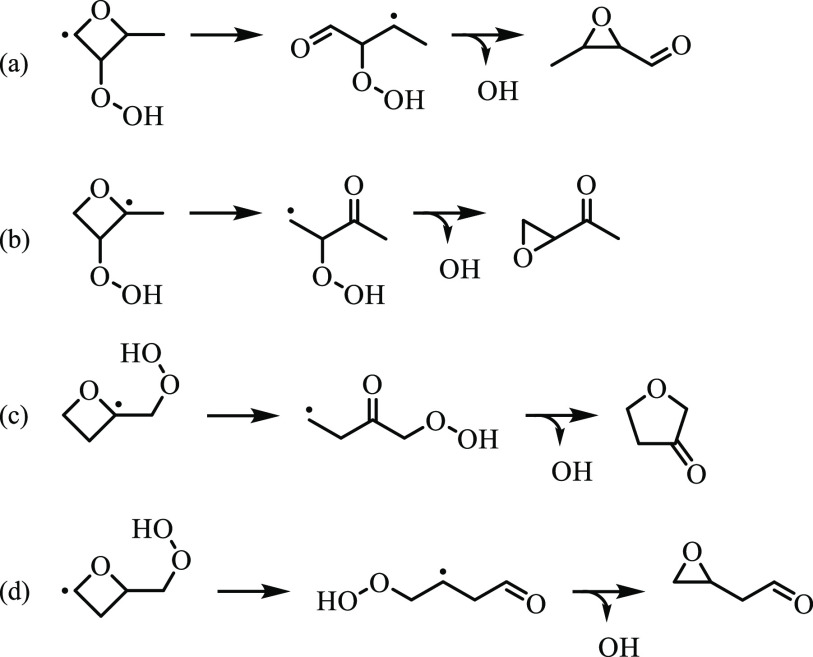
Reaction mechanisms for 3-methyloxirane-2-carbaldehyde, 1-oxiran-2-yl-ethanone, tetrahydrofuran-3-one, and 2-oxiran-2-yl-acetaldehyde via ketohydroperoxide-type radicals.

Reaction mechanisms via ketohydroperoxide-type radical-producing species in Figure 21 are shown in Figure 22. For example, 3-methyloxiran-2-carbaldehdye forms from 2-hydroperoxybutanal-3-yl, which is ∼3 kcal/mol above the lowest-energy barrier and leads to 2-methylpropanedial. The formation pathway is derived from 2-hydroperoxy-3-butanal-3-yl, unfolding via a 1,2-formyl shift and the concerted loss of ȮH, which involves a barrier height of 3.5 kcal/mol. In addition, 1-oxiran-2-yl-ethanone forms via 3-hydroperoxy-2-butanone-4-yl with a barrier of 14.7 kcal/mol. The ring-expansion product, tetrahydrofuran-3-one, forms via 1-hydroperoxy-2-butanone-4-yl with a barrier of 4 kcal/mol. The proceeding step, involving ring opening of QOOH43, was excluded from the ROO4 potential energy surface because the barrier is not submerged. As a result, the barrier to tetrahydrofuran-3-one was calculated only at L2. The barrier to 2-oxiran-2-yl-acetaldehyde, formed via 4-hydroperoxy-butanal-3-yl, is ∼9 kcal/mol. The barrier to QOOH41 exceeded the specified energy threshold for the ROO4 surface because the ROO4 well depth is only ∼25 kcal/mol. As a result, the barrier height for 2-oxiran-2-yl-acetaldehyde formation was calculated only at L1.

**Figure 23:**
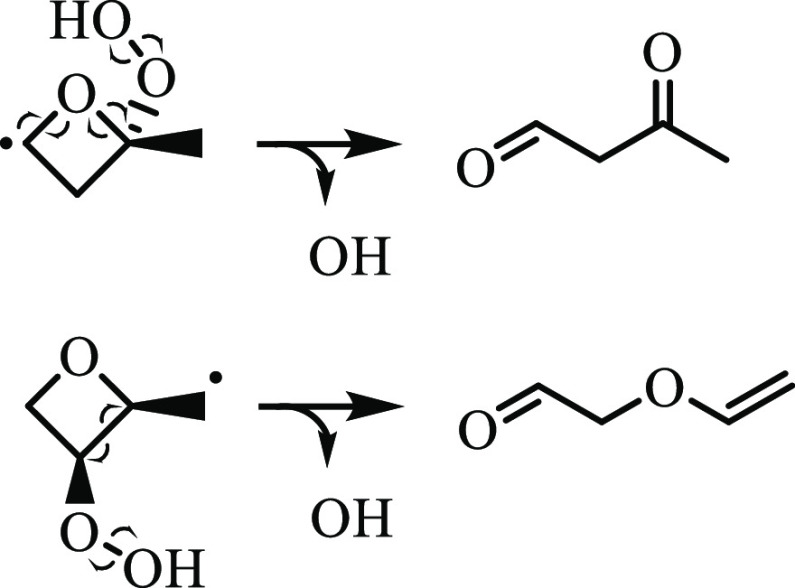
Reaction mechanisms for 3-oxobutanal and 2-(vinyloxy)acetaldehyde via concerted β-scission and OH-elimination, which is the lowest-energy pathway of the two reactions producing the former.

Other species that may contribute to the *m*/*z* 86 ion signal do not form via ketohydroperoxide-type radicals. More specifically, 3-oxobutanal and 2-(vinyloxy)acetaldehyde are produced by concerted ring-opening and OH-elimination, as shown in Figure 23. For example, 3-oxobutanal forms from the *anti*-diastereomers of QOOH31 and QOOH13. KinBot neglected 3-oxobutanal pathways on the corresponding *syn*-surfaces due to barriers exceeding the specified energy threshold. The barrier from *anti*-QOOH31 is submerged below the R3 + O_2_ entrance channel by ∼2 kcal/mol. *anti*-QOOH13 is ∼2 kcal/mol above the R1 + O_2_ entrance channel, and 3-oxobutanal is submerged by ∼80 kcal/mol. 2-(vinyloxy)acetaldehyde is produced from *syn*-QOOH24, and similar to 3-oxobutanal, KinBot neglected the relevant pathway on the *anti*-QOOH24 surface. In contrast, on the *syn*-QOOH24 surface, the barrier to 2-(vinyloxy)acetaldehyde is 14.7 kcal/mol, which is the lowest-energy barrier.

Another species that may contribute to the *m*/*z* 86 ion signal is 2,5-dioxabicyclo[2.2.0]hexane, which is one of the seven cyclic ether isomers that form from 2-methyloxetane. More specifically, 2,5-dioxabicyclo[2.2.0]hexane is produced by QOOH42 and *syn*-QOOH24 via a four-membered transition state. The barrier to the former is 12.3 kcal/mol, and that of the latter is 16.5 kcal/mol.

## 4 Discussion

The detection of species from unimolecular decomposition of 2-methyloxetanyl (Ṙ) and from Ṙ + O_2_ reactions (Figure 2) underscores the complex network of reactions from the oxidation of 2-methyloxetane. S10 provides a table connecting each product detected in the MPIMS experiments to specific Ṙ or Q̇OOH radicals. Species formed directly from unimolecular reaction pathways of Ṙ were ethene, propene, ketene, 1,3-butadiene, methyl, formaldehyde, and acrolein. The latter three are also connected to Q̇OOH-mediated pathways. Ion signals at *m*/*z* 70 and *m/z* 86, the spectra for which exhibited a weak temperature dependence (S11), indicate that typical alkylperoxy-type reaction pathways are relevant to reactions of 2-methyloxetane. The *m*/*z* 70 ion signal may contain contributions from all three conjugate alkene isomers formed via HOO-elimination from 2-methyloxetane, which is, in contrast to alkyloxiranes,^18,19^ likely due to differences in ring strain. The Q̇OOH-mediated formation of bicyclic ethers may contribute to the ion signal at *m*/*z* 86 based on adiabatic ionization energy calculations (S12) and favorable pathways on ROȮ potential energy surfaces. Such pathways contribute directly to the time evolution of ȮH populations.

The formation of ĊH_3_ may occur via ring-opening of R1, R2, and R3 (Figure 2). The most direct pathway is from R2, where methyl forms coincident with acrolein via C–C β-scission of the ring-opening product, but-1-ene-3-oxy. Additional pathways connected to *anti*- and *syn*-conformers of QOOH12 also exist, although the preceding isomerization step is ∼3 kcal/mol above both Ṙ + O_2_ entrance channels. Pathways to ethene arise exclusively from Ṙ radicals, most directly from R3 and R4 via the decomposition of ring-opening products but-2-one-4-yl and H_2_ĊCH_2_OCH=CH_2_, respectively. An indirect pathway also exists on the R1 surface from C–C β-scission of the ring-opening product, butanal-2-yl. However, the isomerization reaction butanal-2-yl → butanal-3-yl is submerged, and either of the carbonyl radicals may react with O_2_ to yield 3-butenal (*m*/*z* 70) coincident with HOȮ. In addition, tunneling^55−57^ may facilitate intramolecular H-transfer in carbonyl radicals. The ring-opening reaction of R3 that yields ethene + acetyl also provides a pathway to methyl vinyl ketone (*m*/*z* 70), upon reaction of but-2-one-4-yl with O_2_, which may contribute to HOȮ formation in *n*-butane oxidation

Reaction pathways from R2 and R4, via the decomposition of but-2-en-4-oxy and but-1-en-4-oxy, respectively, and from *syn*-QOOH24 produce formaldehyde. The unimolecular decomposition of R1, R2, and R3 yields propene. The most direct pathway is from R1, wherein C–C β-scission of butanal-3-yl also yields formyl. Reactions of ring-opening products but-2-one-4-yl and butanal-1-yl, derived from R3 and R4, respectively, with O_2_ may yield ketene. The more facile decomposition channel for the latter radical, however, is *n*-propyl + CO. Several pathways on the surfaces for R1, R2, and R3 produce 1,3-butadiene. The unimolecular reaction of R2 is the most direct and occurs via isomerization of but-2-en-4-oxy into an OH-substituted, resonance-stabilized radical that subsequently decomposes into 1,3-butadiene + ȮH. Reaction from R4 may also contribute and involves a similar reaction sequence in which an intramolecular H shift of but-1-en-oxy forms a resonance-stabilized radical (HO–CH_2_–ĊH–CH=CH_2_) that then undergoes C–O β-scission into 1,3-butadiene + ȮH. Reactions of R1, R2, and R3 yield acrolein, with R2 being the most direct pathway. Another pathway to acrolein is the ring opening of *syn*-QOOH24.

Ring-opening reactions of certain Q̇OOH isomers may contribute to chain-branching. The potential energy surface for *syn*-ROO1 contains an energetically favorable pathway to the formation of performic acid (*m*/*z* 62). However, the ion signal detected at *m*/*z* 62 was obscured by fragment ions of products from chain chlorination (RCl), which precluded the experimental confirmation of performic acid and, by extension, the existence of a chain-branching channel from 2-methyloxetane oxidation (S13). Similar issues arose for the ion signal at *m*/*z* 76, the nominal mass of hydroperoxy acetaldehyde, which is connected to a Q̇OOH ring-opening pathway on the ROO4 potential energy surface: but-1-en-3-oxy-4-hydroperoxy → hydroperoxy acetaldehyde + vinyl.

Consumption mechanisms prescribed for 2-methyloxetane in *n*-butane mechanisms^33,58,59^ include only a fraction of the species observed herein (S14), such as propene + formyl and ethene + acetyl, which are assigned as products of H-abstraction reactions. In some instances, the number of species is minimized as a means of maintaining the overall size of a chemical mechanism. However, the exclusion of detailed chemical mechanisms for 2-methyloxetane is likely to impact species profiles and ignition delay time predictions, as with alkyloxiranes,^20^ because of the impact on radical populations as shown herein.

## 5 Conclusions

Species produced from unimolecular reactions of 2-methyloxetanyl and 2-methyloxetanylperoxy were quantified using photoionization spectral measurements produced from Cl-initiated oxidation of 2-methyloxetane in MPIMS experiments. Potential energy surfaces were also computed for all isomers of Ṙ and ROȮ radicals and, in the latter case, accounted for stereochemistry. Species produced from unimolecular reactions of 2-methyloxetanyl radicals in the experiments included methyl, ethene, formaldehyde, propene, ketene, 1,3-butadiene, and acrolein, each of which is produced along pathways on the surfaces. Reactions of 2-methyloxetanyl with O_2_ yielded products similar to alkyl oxidation, i.e., conjugate alkene + HOȮ and chain-propagation species. All three of the conjugate alkene isomers formed via HOO elimination may contribute to the 2-methyloxetane reaction network based on submerged pathways on the potential energy surfaces and ionization energy calculations. In addition, numerous ȮH-forming channels were identified on the surfaces for 2-methyloxetanylperoxy → products.

Potential energy surfaces for ROȮ isomers included low-lying formation pathways to a ketohydroperoxide species (peracetic acid) and to 3-oxobutanal, the latter of which is a dicarbonyl associated with the unimolecular decomposition of 3-hydroperoxybutanal^60^ formed via *n*-butane oxidation.^8^ Including pathways to 3-oxobutanal from 2-methyloxetane in addition to pathways from 3-hydroperoxybutanal is necessary in order to accurately model the decomposition rates of 3-hydroperoxybutanal. In addition to 3-oxobutanal, as is evident in the photoionization spectra, several Q̇OOH-mediated species were produced, including tetrahydrofuran-3-one and 1-oxiran-2-yl-ethanone, for which viable reaction pathways were identified.

The results affirm that alkyloxetanes, as with alkyloxiranes, undergo Q̇OOH-mediated reactions that directly impact populations of ȮH and other radicals. Both cyclic ether intermediates are abundant in the low-temperature combustion of hydrocarbons and biofuels. Understanding the balance of reactions of cyclic ether radicals as a function of temperature and oxygen concentration enables accurate combustion modeling and requires isomer-resolved speciation experiments. Rate parameters for elementary reactions such as the production of isomers of cyclic ether radicals via H-abstraction, ring-opening of (carbon-centered) 2-methyloxetanyl radicals, and reactions with O_2_ are required for detailed chemical kinetics mechanisms to improve the fidelity of combustion modeling given the importance to Q̇OOH and related chain-branching as well as the formation of other important species from cyclic ether oxidation.

## Supporting Information Available

SAR calculations of the initial radical distribution for 2-methyloxetane + Ċl; ^13^C time profiles of 2-methyloxetane at 650 and 800 K; absolute photoionization cross-sections σ(*E*); equation for relative yield from the least-squares fitting of photoionization spectra; geometry coordinates for optimized structures; adiabatic ionization energies calculated at the CBS-QB3 level of theory at 0 K; potential energy surfaces; difference mass spectrum at 800 K; methylperoxy radical (CH_3_OȮ) absolute photoionization spectrum; photoionization spectra and fits at 650 K and 800 K; grouping of radical-specific products from MPIMS experiments; temperature dependence of ion signals at *m*/*z* 70 and *m/z* 86; cyclic ether isomer structures; ion signal at *m*/*z* 62 and *m/z* 76; and prescribed consumption reactions of 2-methyloxetane (PDF)
