# Supplementary material for: Unimolecular Reactions of 2-Methyloxetanyl and 2-Methyloxetanylperoxy Radicals
Source: J Phys Chem A. 2023 Aug 3;127(32):6816–29. doi: 10.1021/acs.jpca.3c03918 (PMC10440797; doi:10.1021/acs.jpca.3c03918)
Supplement: Supplementary file 1 — jp3c03918_si_001.pdf [file jp3c03918_si_001.pdf]

Unimolecular Reactions of 2-methyloxetanyl and 2-methyloxetanylperoxy Radicals

Anna C. Doner[a, †], Nicholas S. Dewey[a, †], and Brandon Rotavera[a, b, *]

[a]*University of Georgia, Department of Chemistry, Athens, Georgia 30602, USA*
[b]*University of Georgia, College of Engineering, Athens, Georgia 30602, USA*

*Corresponding author, rotavera@uga.edu

Table of Contents

S1a – SAR calculations of initial radical distribution for 2-methyloxetane + Ċl

S1b – ^{13}C time profiles of 2-methyloxetane at 650 K and 800 K

S2 – absolute photoionization cross-sections $\sigma(E)$

S3 – equation for relative yield from least-squares fitting of photoionization spectra

S4 – geometry coordinates for optimized structures

S5 – adiabatic ionization energies calculated at the CBS-QB3 level of theory at 0 K

S6 – potential energy surfaces

S7 – difference mass spectrum at 800 K

S8 – methylperoxy radical (CH_3OO) absolute photoionization spectrum

S9 – photoionization spectra and fits at 650 K and 800 K

S10 – grouping of radicals-specific products from MPIMS experiments

S11 – temperature dependence of ion signal at *m/z* 70 and *m/z* 86

S12 – cyclic ether isomer structures

S13 – ion signal at *m/z* 62 and *m/z* 76

S14 – prescribed consumption reactions of 2-methyloxetane

S1a. SAR calculations of initial radical distribution for 2-methyloxetane + Ċl

	per C-H bond					
	k_1	k_2	k_3	k_4	k_{total}	Δ (reference)
site-specific	8.85E-11	1.87E-11	8.85E-11	1.09E-11	3.36E-10	1.40
site-specific fraction	53%	11%	26%	10%	100%	

Knox, J.H. and Nelson, R.L.
Trans. Faraday Soc., vol. 55, 1959
reference reaction: cyclobutane + Cl
$2.4 \cdot 10^{-10}$ cm^3 $molec^{-1}$ s^{-1}

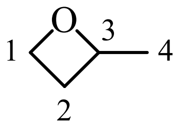

$k_1 = k_{cyclobutane}F(-O-)F(-CH_2-)$

$k_2 = k_{cyclobutane}F(-CH_2-)F(-CH-)$

$k_3 = k_{cyclobutane}F(-O-)F(-CH_2-)F(-CH_3)$

$k_4 = k_{\textit{iso}\text{-butane}}$

Aschmann and Atkinson, *Int. J. Chem. Kin.*, vol. 45, 2013

$F(-CH_3-)$	1.00	
$F(-CH_2-)$	0.79	
F(>CH-)	0.79	
F(-O-)	3.74	scaled substituent factor, F, for reaction with OH by 0.61
$F(-CH_2-)$	0.79	Cl
$F(-CH_2-)$	1.29	OH
ratio	0.61	

S1b. time profiles of ^{13}C 2-methyloxetane at 650 K and 800 K

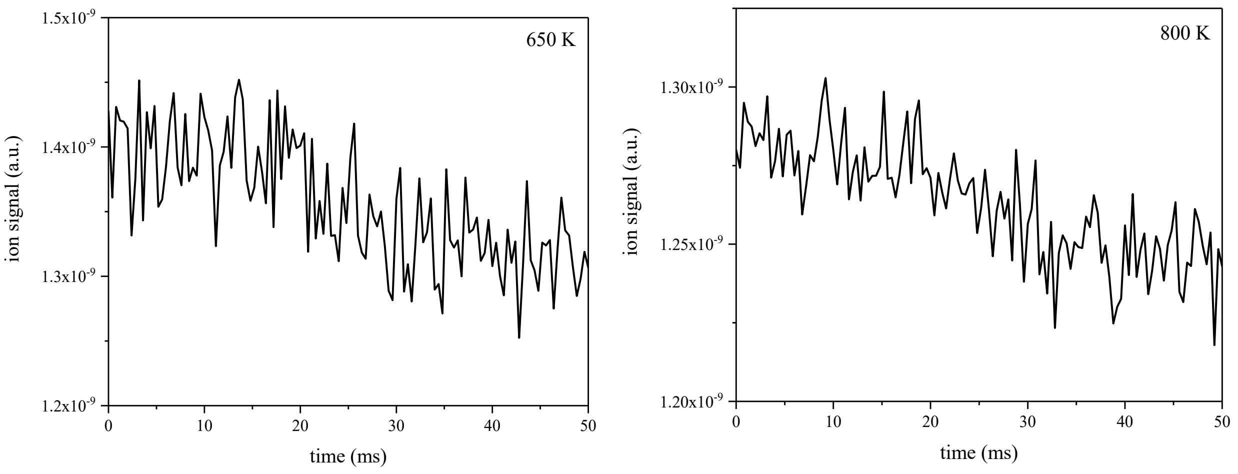

S2. absolute photoionization cross-sections, $\sigma(E)$, of methyl vinyl ketone and 1-oxiran-2-yl-ethanone

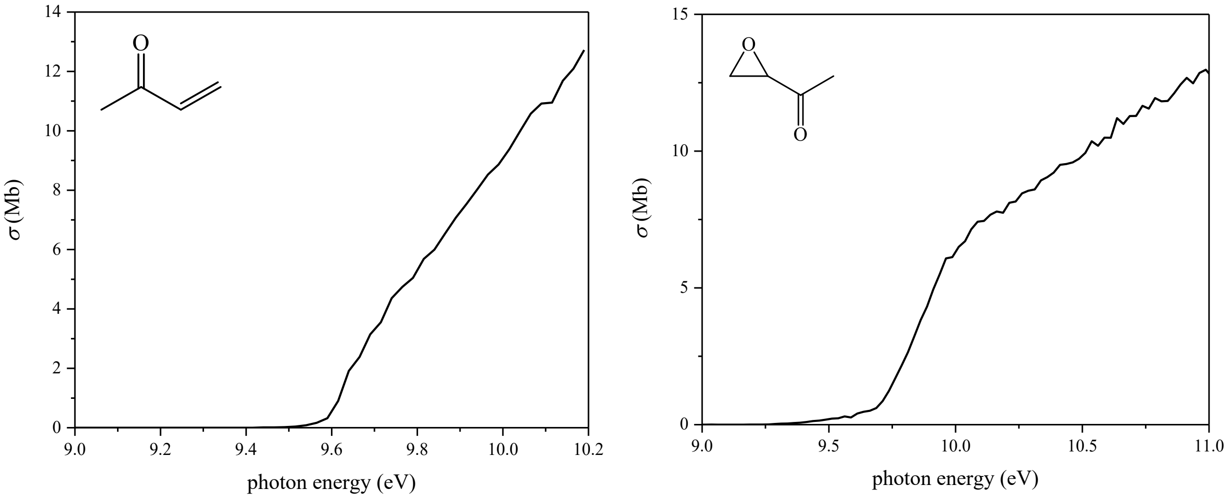

S3. derivation of relative yield equation from least-squares fitting of photoionization spectra

Generic ion signal for species i:

$$S_i(\mathrm{E})=\Lambda\sigma_i(E)N_i m_i^{\beta(P,T)} \qquad (1)$$

Λ	instrument response factor
$\sigma_i(E)$	absolute photoionization cross-section of species i (Mb)
N_i	concentration of species i
$m_i^{\beta(P,T)}$	mass discrimination factor of species i

Concentration ratio of species x relative to species y, derived from the application of Eqn. 1 to ion signals of both species (Λ cancels):

$$N_x/N_y = (S_x/\sigma_x)(\sigma_y/S_y)(m_y/m_x)^{\beta(P,T)} \qquad (2)$$

where the generic term (S_i/σ_i) is the derived fitting coefficient A for species i in Eqn. 4, determined using the absolute photoionization cross-section of species i, $\sigma_i(E)$, measured in a separate experiment.

$$S_i(E) = A_i\sigma_i(E) \qquad (3)$$

$$A_i = (S_i(E)/\sigma_i\,(E)) \qquad (4)$$

Determination of species x concentration using [propene]$_{650\ \mathrm{K}}$, where A_x is determined separately from A_1; $\beta(P,T)$ is 0.59 for 10 Torr and 600 K, determined from previous analysis of mass discrimination in the molecular beam.

$$N_x = \left((A_x/A_y)(m_y/m_x)^{\beta(P,T)}\right)\cdot N_y \qquad (5)$$

From the above, the relative yield for a given species i is defined in Eqn. 6, using N_x and [propene]$_{650\ \mathrm{K}}$.

$$branching\ fraction\ of\ species\ i = \left({}^{N_x}/_{[propene]_{650\ K}}\right)\cdot 100 \qquad (6)$$

S4. geometry coordinates for all optimized structures

S4.1.1 R1

MOR1rop.inp

C	0.011915	-0.020356	-0.237729
C	1.418575	0.067447	0.29879
C	2.03151	1.418069	0.277145
H	2.017602	-0.666913	-0.268494
H	1.392797	-0.357984	1.317826
C	3.414116	1.621909	0.782396
H	3.50763	1.330239	1.838851
H	3.727451	2.663369	0.696539
H	4.141953	1.00658	0.233514
O	-0.623463	0.904378	-0.666507
H	-0.427995	-1.040078	-0.213475
H	1.4462	2.24234	-0.110098

MOR1.inp

C	-6.521994	2.55005	-0.023448
C	-5.573472	1.435471	-0.350337
C	-4.740823	2.578591	-0.972779
H	-5.932792	0.688855	-1.065753
H	-5.106527	0.923785	0.496175
C	-3.44398	2.947873	-0.298741
H	-3.586137	3.027217	0.782054
H	-3.074077	3.904976	-0.673501
H	-2.688911	2.182024	-0.498604
O	-5.805753	3.530168	-0.677574
H	-7.604359	2.610863	-0.004549
H	-4.624525	2.507557	-2.057264

MOR1RO.inp

C	-6.60941	2.577541	-0.173758
C	-5.599202	1.496628	-0.38461
C	-4.620183	2.489945	-1.012459
H	-5.925169	0.669727	-1.025712
H	-5.180691	1.062477	0.533962
C	-3.363151	2.879696	-0.327255
H	-3.531196	2.990147	0.747679
H	-2.982041	3.823978	-0.722735
H	-2.584554	2.118662	-0.469817
O	-5.981287	3.675063	-0.401386
H	-7.683323	2.531118	-0.012881
H	-4.643152	2.572447	-2.095347

S4.1.2 R2

711952161721200720062_intra_H_migration_suprafacial_2_6.inp

C	1.64472902	-1.13561275	0.60297324
C	1.10589048	-0.05973878	0.00006846
C	1.56187958	1.30025445	0.17187145
H	0.2769216	-0.20714914	-0.68980585
C	2.77349021	1.68448373	0.93008878
H	2.62266261	1.63151923	-0.42313107
H	3.48007808	0.9360696	1.25944003
H	2.8823976	2.70406186	1.27103448
O	1.26365096	-2.42623247	0.40979748
H	0.84727401	2.09562445	-0.00903444
H	0.5500556	-2.45932528	-0.23103298
H	2.44899472	-1.07286294	1.32500199

712193423882780860062.inp

C	1.50013986	0.00924178	-0.01220047
C	0.0322833	0.0166706	-0.00389386
C	0.698516	-0.32564333	-1.27150485
H	-0.63856658	0.84045137	0.20428605
C	0.64526642	0.61124859	-2.45726142
H	0.71173826	1.65133388	-2.13446841
H	-0.28888409	0.47649086	-3.0090767

H	1.47708469	0.41760989	-3.13984179
O	2.20622916	1.21758281	-0.00031692
H	0.74944744	-1.38279904	-1.52967048
H	2.16706537	1.58459561	0.8840795
H	2.03374702	-0.82519542	0.44117919

711952161721200720062_r12_insertion_R_11_9_1.inp

C	1.19750505	-0.02946561	0.16794087
C	1.97393889	-1.25623728	0.19316557
C	1.40094777	-2.44957887	-0.00983375
H	3.03888371	-1.16055973	0.38755729
C	2.10793676	-3.76233584	0.00612287
H	3.17593438	-3.64456205	0.19683192
H	1.97796357	-4.27882381	-0.9501897
H	1.6853048	-4.41528535	0.77649637
O	1.68222937	1.09174644	0.34252562
H	0.32797529	-2.47887775	-0.1998563
H	2.28609679	1.89703864	-0.86331057
H	0.11942315	-0.13316994	-0.04778273

711952311741200570042_R_Addition_MultipleBond_2_1_11.inp

C	2.22809449	0.99463385	-0.51463537
C	1.45125803	-0.04299651	-0.13029923
C	-0.00265988	0.00563721	-0.04820181
H	1.93377413	-0.98100517	0.13766921
C	-0.74938968	1.0958663	0.1481148
H	3.92243306	0.17297134	-0.22533465
H	-1.83110416	1.03507565	0.15624428
H	-0.30714542	2.0709344	0.326107
O	3.57262539	0.9664779	-0.637242
H	-0.50442981	-0.95372723	-0.15144179
H	2.01189613	1.9877873	1.26563181
H	1.81196734	1.89922214	-0.94137452

711912311911110570002_intra_H_migration_3_6.inp

C	0.16583936	0.07979436	-0.22919548
C	1.59224407	-0.08330475	0.25033126
C	2.28707587	1.2469717	0.19214454
H	1.57929219	-0.50767759	1.25572688
C	2.88557983	1.72710274	-1.08134122
H	3.55035272	1.24138326	-0.06640282
H	3.05730994	2.7844125	-1.22989852
H	2.91105842	1.06369024	-1.93667982
O	-0.80640246	-0.26512286	0.3884937
H	2.04370182	1.9955582	0.93768686
H	2.0955717	-0.79415986	-0.41882357
H	0.06236739	0.59133972	-1.21006485

711952311741200570042.inp

C	1.45665615	0.04171729	0.04498793
C	-0.03593282	-0.01806232	0.05029073
C	-0.83525922	-0.66577002	-0.88169507
H	-0.51498426	0.50069623	0.8759119
C	-0.41605229	-1.375573	-1.98902859
H	1.55575284	1.92878724	-0.41632172
H	-1.13730276	-1.84267536	-2.647719
H	0.63048626	-1.49290546	-2.24419401
O	1.9359352	1.36291291	0.25779674
H	-1.90845393	-0.60750795	-0.71455442
H	1.87347831	-0.37740451	-0.87822354
H	1.85963585	-0.5454519	0.87636199

531371300930560080002.inp

C	0.057367	-1.946603	-0.529951
C	0.387005	-0.709397	-0.744942
C	0.729366	0.574825	-0.96813
C	1.698158	1.278446	-0.259549
H	1.914196	2.310601	-0.501964
H	2.25248	0.810177	0.544281
H	0.197167	1.086193	-1.768078
H	-0.731715	-2.212242	0.171312
H	0.558276	-2.766636	-1.04144

712193323793121330062_r13_insertion_ROR_3_2_1_9.inp

C	1.89184618	1.38034288	0.35879991
C	1.48936101	0.21964288	-0.30619919

C	0.00874832	0.34424593	-0.15694978
H	1.94497107	-0.0952831	-1.23869091
C	-0.86624532	-0.24839131	-1.23903555
H	-0.48278214	0.01329485	-2.22798869
H	-1.88343747	0.13973829	-1.15134925
H	-0.90443096	-1.33729708	-1.14478377
O	0.14275041	1.7810751	-0.24585421
H	-0.35835181	0.06358735	0.84074515
H	2.75087012	1.96080632	0.0276177
H	1.58280354	1.56177994	1.38222558

71191235172120063 0002_intra_H_migration_9_6.inp

C	-0.8321697	1.28645797	0.01541956
C	-0.09280785	-0.03218453	0.02034776
C	1.23161131	-0.10453984	0.00244619
H	-0.71053547	-0.92447058	0.03919141
C	2.07854089	1.11476104	-0.02360008
H	1.18277134	2.00045376	-0.0207904
H	2.65489232	1.26713489	-0.9378465
H	2.68065629	1.28797682	0.87009731
O	0.000379	2.40514571	-0.00765135
H	1.7205109	-1.07746186	0.00660823
H	-1.4681935	1.32108137	0.91307706
H	-1.49153053	1.30133326	-0.86581318

41113108060006000 0002.inp

C	1.08770286	-0.35353224	1.05719848
C	1.63908149	0.2450702	0.03398306
H	1.06219342	-1.34630943	1.48646657
C	2.53762384	-0.41703446	-0.9852005
H	2.67861974	-1.47697081	-0.76708539
H	3.51783359	0.06843416	-0.99899981
H	2.10898613	-0.32231483	-1.98718551
H	1.44992292	1.30897042	-0.11736689

71195216172120072 0062_Intra_R_Add_ExoTetCyclic_F_1_3_5.inp

C	0.21352098	0.0257965	-0.29227783
C	1.45528919	-0.19958943	-1.01020533
C	2.00142022	0.96319394	-0.48327709
H	1.974856	-1.154182	-1.0271567
C	3.25459478	1.04908616	0.33812606
H	3.89521346	0.17911019	0.17287171
H	3.0009868	1.07935177	1.40540297
H	3.81750488	1.95991472	0.11692964
O	-0.03651846	-0.49295739	0.94658097
H	1.50011045	1.90849894	-0.68251397
H	0.63740285	-1.14704857	1.14913834
H	-0.60055872	0.65288823	-0.63361902

71191246223109012 0002_Intra_R_Add_Endocyclic_F_9_1.inp

C	0.15338648	1.55691265	0.70040257
C	1.403621	1.28482528	0.13974788
C	1.27531168	-0.18972888	-0.05864596
H	1.84671718	1.92622289	-0.61429284
C	2.03041653	-0.81992594	-1.20773266
H	1.91236157	-0.22461493	-2.11614979
H	1.64533771	-1.82336183	-1.40224462
H	3.09404928	-0.9006352	-0.96643161
O	-0.13423444	-0.00912467	-0.32462014
H	1.41050591	-0.77151958	0.86449409
H	-0.38191437	2.47960446	0.4844699
H	-0.17277358	1.03571473	1.59358915

70178214176094038 0001.inp

C	-0.01714824	-0.0344462	-0.16254537
C	1.474548	-0.00418979	0.10258607
C	1.99103937	1.40662155	0.02838179
H	1.63822743	-0.4051074	1.1097548
C	2.35565605	2.13277833	1.07819231
H	2.33003685	1.72855036	2.08618152
H	2.70185599	3.15387791	0.96523886
O	-0.53972774	-0.63959669	-1.05785037
H	2.03254713	1.84496571	-0.96620168
H	1.96843023	-0.6540111	-0.62341408
H	-0.62330345	0.57215463	0.54400774

701782031571050570001.inp

C	1.10933339	0.00470782	-0.00000683
C	1.75723604	1.32332075	0.00000411
C	1.02935176	2.44193007	0.00007561
H	2.84358612	1.33948172	-0.00004867
C	1.57073315	3.83173149	0.00009355
H	2.66185755	3.84135109	0.00004048
H	1.21540715	4.37943874	0.87852239
H	1.21532126	4.3794948	-0.87826561
O	1.699418	-1.04748225	-0.00007048
H	-0.05699281	2.34656666	0.00012576
H	-0.00232266	0.0308078	0.00004895

711952311741200570042_r12_insertion_R_11_1_2.inp

C	1.26128961	0.08144925	0.14431614
C	-0.02253445	0.30102533	-0.21824843
C	-0.8501427	-0.66172229	-0.93319199
H	-0.47729752	1.25663115	0.03573166
C	-0.41709988	-1.63214803	-1.74281731
H	1.62526167	1.80432928	0.87148329
H	-1.1120626	-2.32551743	-2.20121033
H	0.63372938	-1.75024638	-1.98749402
O	2.03891139	0.93853341	0.84021402
H	-1.92174654	-0.55275742	-0.78281448
H	1.98177357	0.34404762	-1.75668153
H	1.72387808	-0.89485951	0.06432797

170170000000000000002.inp

H	0.78377938	0.68035453	1.86788243
O	1.00071762	1.61199847	1.70220657

711952311741200570042_intra_H_migration_5_11.inp

C	1.35817705	0.01275797	0.00047162
C	1.40617792	-0.77142883	1.27258749
C	0.19281736	-1.10548538	1.71702493
H	2.34084406	-1.02726138	1.76270307
C	-0.93075701	-0.62113218	0.86599491
H	1.74625924	1.68771155	0.85494806
H	-1.57797354	0.14328767	1.29738107
H	-1.48927804	-1.3767986	0.31380589
O	1.94216507	1.26967359	0.01360946
H	0.03023558	-1.69346585	2.61672935
H	0.01930695	0.00048984	-0.00856313
H	1.67905172	-0.5051436	-0.90407777

711912311911110570002_intra_H_migration_3_12.inp

C	1.64872201	1.13214647	0.45516879
C	1.52499915	-0.04537192	-0.4763485
C	0.01886328	0.06482454	-0.26984017
H	1.90390301	0.12130889	-1.48463113
C	-0.75780198	-1.09452301	0.2766165
H	-0.26383598	-1.52300976	1.15346392
H	-0.83175467	-1.88964534	-0.47718115
H	-1.77104785	-0.80313336	0.55968865
O	2.10064591	2.22758609	0.31044575
H	-0.49476722	0.73796443	-0.95112314
H	1.93345709	-0.9653699	-0.05477328
H	0.42685087	0.90948155	0.90660969

711912311911110570002_r12_insertion_R_1_2_4.inp

C	1.10958649	0	0
C	1.68446832	2.11725499	0
C	0.866989	2.55786341	1.00814655
H	2.7196837	1.85582268	0.19405017
C	-0.50841552	3.12736006	0.80561472
H	-0.78089102	3.16165131	-0.25530911
H	-0.56900405	4.1521302	1.20072432
H	-1.27463485	2.54606816	1.33881379
O	1.81279227	-0.78116704	0.61509674
H	1.20793572	2.46298077	2.03923141
H	1.42942756	2.30191038	-1.04071438
H	0	0	0

711912311911110570002_intra_H_migration_3_4.inp

C	-0.02411574	0.01981504	-0.18414181
C	1.4278453	0.04137927	-0.19719325

C	2.18519929	1.21564819	0.26310521
H	1.8487	0.07790513	0.97000154
C	3.63572009	1.38117038	-0.04322947
H	4.13885027	2.00225762	0.70016516
H	3.76482515	1.86271289	-1.02200919
H	4.15118645	0.41742792	-0.09121498
O	-0.71273121	0.91417016	0.27971085
H	1.5932535	2.03444105	0.65416804
H	1.9610701	-0.73634791	-0.73545377
H	-0.48446527	-0.8922612	-0.60780636

711952161721200720062_intra_H_migration_1_11.inp

C	1.24730508	0.05988175	0.01453943
C	1.99699877	-1.17486749	0.1074258
C	1.43408163	-2.38734033	-0.01525179
H	3.06850425	-1.07540457	0.26640581
C	2.17516299	-3.68362306	0.03496356
H	3.24320295	-3.52918431	0.20184738
H	2.05010512	-4.2399492	-0.9000205
H	1.78951128	-4.32188523	0.83644946
O	1.80057241	1.26033912	0.39857268
H	0.35675988	-2.4478309	-0.16558032
H	1.65725455	0.91689592	-0.79510964
H	0.15468062	0.00285713	0.01542562

711912462231090120002.inp

C	1.64144742	-1.02532736	0.30904555
C	1.18245513	-0.00581093	-0.40361415
C	1.77615517	1.37847097	-0.35293381
H	0.3394256	-0.12728103	-1.08050677
C	0.74609655	2.40573214	0.19222455
H	-0.14607707	2.41278593	-0.4361559
H	1.18151192	3.40529639	0.21323209
H	0.47333424	2.10299917	1.20466237
O	2.10865708	1.84658313	-1.59339138
H	2.64792246	1.40180574	0.32143504
H	1.18486629	-2.00717732	0.25693195
H	2.49480716	-0.91281434	0.97230407

712193323793121330062_r12_insertion_R_1_9_3.inp

C	-6.84303857	2.51183474	-0.12503705
C	-5.62734302	1.6633756	-0.26969213
C	-4.75124665	2.60923947	-0.82991028
H	-5.29674011	0.99735604	0.52038749
C	-3.29284522	2.69638176	-0.53243981
H	-3.02450521	2.13327339	0.36210413
H	-3.02013359	3.74389628	-0.38424107
H	-2.71169577	2.32763698	-1.38408213
O	-6.01393481	3.65032565	0.14387102
H	-5.06599589	3.10826091	-1.7427373
H	-7.50153073	2.25166353	0.70895498
H	-7.43598841	2.64718583	-1.03967889

711952311741200570042_intra_H_migration_9_4.inp

C	1.888968	-0.537427	-0.420416
C	1.548743	0.741886	-0.380118
C	2.257444	1.914276	-0.879465
H	0.493615	0.986054	0.117506
C	3.567999	2.107442	-0.725912
H	0.030102	1.433766	1.782272
H	4.057256	2.984667	-1.133813
H	4.183836	1.395425	-0.185923
O	-0.499305	1.269026	0.989697
H	1.654433	2.666706	-1.380943
H	1.274699	-1.298681	0.046443
H	2.780331	-0.86145	-0.951945

541501561120600080001.inp

C	1.71732396	-1.15741985	0.07659919
C	1.10480529	0.00071781	-0.16789842
C	1.77598295	1.29721852	-0.1562665
H	0.04001839	0.0084595	-0.39258647
C	1.16346428	2.45535618	-0.40076411
H	1.70030019	3.39648886	-0.38188732
H	0.10218956	2.49566691	-0.62798042

H	2.84076985	1.28947683	0.06842156
H	2.77859868	-1.19773058	0.3038155
H	1.18048805	-2.09855253	0.0577224
711912462231090120002_R_Addition_MultipleBond_9_3_5.inp			
C	1.5244475	-0.93185984	0.38315754
C	1.29168626	-0.05010007	-0.582943
C	2.01117926	1.25283732	-0.66402333
H	0.59668782	-0.25277668	-1.39300514
C	0.62050254	2.45673591	0.39246126
H	-0.25548318	2.32559342	-0.23133316
H	1.12189016	3.41425392	0.32394021
H	0.61097464	1.96659721	1.35921788
O	2.09952607	1.88397249	-1.72264555
H	2.74753295	1.42336668	0.14715197
H	1.02777961	-1.89458587	0.41223995
H	2.23387834	-0.71877199	1.17901495
711952311741200570042_r12_insertion_R_11_1_9.inp			
C	1.27890173	0.07250702	0.1277431
C	-0.0239957	0.26631333	-0.17692275
C	-0.84693657	-0.68758919	-0.90889379
H	-0.49896251	1.19258173	0.1407604
C	-0.41398981	-1.60224108	-1.7810786
H	1.6148693	1.76904741	0.92670419
H	-1.10327781	-2.29535671	-2.24833525
H	0.63013236	-1.67281356	-2.06894478
O	2.05451103	0.92029541	0.83715929
H	-1.91505672	-0.62216526	-0.71480554
H	1.91885663	0.45086558	-1.78240019
H	1.76890741	-0.88268151	-0.01737314
531371410970450040002.inp			
C	1.69438552	-1.14202675	-0.06790994
C	1.09755471	0.00800847	0.14853111
C	1.77155881	1.31396348	0.13420296
H	0.02634794	0.02093727	0.35584791
C	1.14257992	2.46698061	0.35740884
H	1.67282722	3.41178817	0.33885738
H	0.07739667	2.49983879	0.56553605
H	2.83870194	1.30941566	-0.07177828
H	2.69040779	-1.49954369	-0.29115149
711912311911110570002_h2_elim_4_10.inp			
C	1.11571399	-0.02689341	-0.03543711
C	1.81675425	1.27967474	0.033916
C	3.13273883	1.47806148	0.27330185
H	1.55593992	1.33300399	-1.99761037
C	4.2144177	0.45756399	0.35987996
H	3.83127962	-0.55930546	0.35430885
H	4.81042498	0.62408142	1.26276553
H	4.89734921	0.58578837	-0.48874408
O	1.62127126	-1.11801198	0.06498568
H	3.45351982	2.51305133	0.37766623
H	1.15700353	2.14299022	0.03629754
H	0.02016603	0.06511949	-0.182534
712193323793121330062_intra_R_migration_2_1.inp			
C	-0.24852137	1.48857115	0.00098477
C	0.00920808	0.14775995	0.29650117
C	1.49795995	0.1455619	0.1793757
H	-0.5012948	-0.37152772	1.10025553
C	2.27593101	-0.82867243	1.03575112
H	1.89822284	-0.82402894	2.06086791
H	3.33111876	-0.5473813	1.05723827
H	2.19810922	-1.84067219	0.6284882
O	1.51768155	1.5003833	0.68359137
H	1.85780875	0.12749054	-0.85943019
H	-1.04791371	2.03943266	0.49297405
H	0.1042457	1.92358611	-0.92752895
711912461931090420002.inp			
C	0.12071359	0.03025851	-0.22379991
C	1.5780957	-0.06921487	0.23896329
C	2.22636831	1.27995088	0.33086169
H	2.12714649	-0.70924606	-0.45759634

C	3.2274155	1.69591255	-0.43587125
H	1.59002811	-0.56289449	1.21657883
H	3.64910558	2.68915659	-0.32961078
H	3.66595993	1.05140353	-1.19259821
O	-0.56931875	-1.13798769	-0.24822085
H	1.81863997	1.95942274	1.07874661
H	0.04512631	0.51310198	-1.21634716
H	-0.45749313	0.71312722	0.43228935

71191231178124057002.inp

C	1.20911396	-1.34070663	0.52198463
C	1.51448902	-0.1866736	-0.26318434
C	2.04399847	1.07444723	0.3120995
H	1.35813931	-0.24930402	-1.33805293
C	3.37089548	1.50046009	-0.33248165
H	3.26442233	1.63271746	-1.41282399
H	4.14645629	0.75135549	-0.15710253
H	3.71245105	2.4473157	0.09123647
O	1.35684817	-1.39413283	1.7404664
H	2.15776434	0.95582757	1.39141784
H	1.30188473	1.86812279	0.14898458
H	0.82054469	-2.21811386	-0.02597824

711912462231090120002_intra_H_migration_9_11.inp

C	1.86099575	1.13502004	-0.47203372
C	1.58238197	-0.00079301	0.14118125
C	0.10207367	-0.08681768	0.38949055
H	2.29007506	-0.77337136	0.42695229
C	-0.56829281	-1.28077811	-0.27387352
H	-0.365508	-1.27689052	-1.34733792
H	-1.64886125	-1.23920315	-0.12160418
H	-0.19674299	-2.21643024	0.15498596
O	-0.3985613	1.13581117	-0.14768322
H	-0.09681454	-0.0991639	1.47008669
H	0.66142128	1.61561702	-0.55570093
H	2.74617532	1.63374947	-0.8412311

71195227191111053 0042.inp

C	-0.001061	0.082991	0.065893
C	1.444388	-0.039756	-0.305203
C	2.100171	1.311283	-0.415912
H	1.988865	-0.643732	0.43999
C	2.980337	1.792999	0.456198
H	0.354163	1.457376	1.334758
H	3.402545	2.785886	0.347625
H	3.322761	1.199032	1.299605
O	-0.325752	0.792419	1.181206
H	1.776037	1.931936	-1.248925
H	1.511917	-0.569803	-1.25997
H	-0.701352	-0.718935	-0.131334

300540360000000000001.inp

C	-2.27896838	0.05506765	-0.31633448
O	-3.40087662	0.1795552	-0.71653168
H	-2.05862978	-0.20897855	0.73644276
H	-1.40209022	0.1974267	-0.9777176

711912351721200630002_intra_R_migration_9_2.inp

C	1.08044409	0.0454665	-0.01243447
C	0.97462601	2.30343427	0.07420354
C	0.59834187	3.0393135	-0.93981091
H	1.29519605	2.50659079	1.08702177
C	0.17484211	2.59675332	-2.30903181
H	0.2064089	1.51292332	-2.40615791
H	0.82942252	3.04348578	-3.06285564
H	-0.84119839	2.94557915	-2.51457245
O	0.72202367	-0.31531536	-1.11991737
H	0.59307951	4.12096533	-0.76197201
H	0.38147352	0.05274489	0.84368977
H	2.15127149	0.16006244	0.23610288

711952161721200720062_intra_H_migration_1_6.inp

C	1.35815202	0.01279628	-0.0003466
C	1.40645867	-0.77163267	-1.27230175
C	0.19320786	-1.10583184	-1.71693123
H	2.34124058	-1.02751622	-1.76216994

C -0.93056954 -0.62136526 -0.86623397
H 0.01928061 0.00046586 0.00840813
H -1.48917826 -1.37695041 -0.31402254
H -1.57772446 0.1429465 -1.297904
O 1.9420836 1.26973714 -0.01360209
H 0.03084443 -1.69399545 -2.61655541
H 1.74633381 1.68760534 -0.85506138
H 1.67885938 -0.50491701 0.90436988
711952311741200570042_beta_delta_5_3_2_1_11.inp
C 2.01427399 1.71749246 2.37445254
C 2.52536745 1.66696451 1.12380663
C 1.82625592 1.11076285 -0.02721602
H 3.5240574 2.06518565 0.95491963
C 0.87220115 0.1760369 0.00091218
H 3.54724778 2.42876556 3.25403228
H 0.5674463 -0.30625785 0.924268
H 0.38316023 -0.1472612 -0.91023314
O 2.6226454 2.26097012 3.4505994
H 2.13591673 1.50560963 -0.99211674
H 2.30855415 -0.28192308 2.71802141
H 0.97678023 1.48584352 2.58300398
712193323793121330062_intra_R_migration_2_9.inp
C -6.88657329 2.35547471 -0.04448879
C -5.70087796 1.66433254 -0.30533535
C -4.85426903 2.79254718 -0.79575712
H -5.31321991 0.91263324 0.37362417
C -3.36250325 2.71564884 -0.55807146
H -3.15361139 2.39958567 0.46663623
H -2.90801933 3.69646477 -0.71438302
H -2.90171131 2.00841031 -1.25344477
O -5.53987365 3.67639384 0.12020229
H -5.07074262 3.08699401 -1.83275495
H -7.52772969 2.09500762 0.79551173
H -7.36586654 2.93693742 -0.82424003
711912462231090120002_r12_insertion_R_2_3_10.inp
C 2.00632266 -0.82312882 0.1948617
C 1.11251637 0.02733644 -0.22920605
C 1.65565609 2.11110684 -0.04617629
H 0.12255458 -0.02371361 -0.66195318
C 1.28959025 2.58127166 -1.43751877
H 0.26747421 2.30814099 -1.70832748
H 1.99271463 2.18345454 -2.17055669
H 1.36163459 3.67407884 -1.44900219
O 2.82095115 2.03491972 0.3318192
H 0.84180335 2.15940879 0.70644443
H 1.82124501 -1.8956841 0.15203846
H 2.95433975 -0.4808355 0.59940709
711912351721200630002_r12_insertion_R_11_1_9.inp
C 1.83178168 -0.00091595 -0.00268811
C 1.99324453 1.47111668 -0.0088803
C 2.01553592 2.17301534 1.12449083
H 2.12687301 1.93771546 -0.98100903
C 2.21579007 3.64829719 1.22681843
H 2.34764859 4.10680353 0.24531993
H 3.09415604 3.8750944 1.83897554
H 1.35781981 4.11723097 1.71899403
O 2.02722227 -0.70933356 -0.97999796
H 1.87698078 1.6373354 2.06435749
H 0.06555057 0.05946255 0.012156
H 1.73223562 -0.45530012 1.00743151
712193323793121330062_r13_insertion_ROR_1_2_3_9.inp
C -0.06312529 0.05961839 -0.06768805
C 1.41829248 0.04528422 0.08737607
C 1.67220376 1.42535118 0.00298109
H 1.90956476 -0.5666133 0.83660357
C 2.71454475 2.14654555 0.78809049
H 3.0761888 1.55276149 1.62829482
H 2.29247264 3.07855531 1.17129384
H 3.55627602 2.41402702 0.14084184
O -0.12214455 1.31078081 0.63016504

H	1.31480077	1.94984354	-0.87936846
H	-0.59739048	-0.74557344	0.4452259
H	-0.42905567	0.15139329	-1.09926608

71219332379312133006 2_r12_insertion_R_3_9_1.inp

C	-6.88178837	2.28610459	-0.06115037
C	-5.66426106	1.63958619	-0.2872801
C	-4.86034105	2.78844791	-0.80106944
H	-5.25369141	0.92541486	0.41819888
C	-3.36986342	2.78309565	-0.54379044
H	-3.16093621	2.50671327	0.49231436
H	-2.95583871	3.77761109	-0.72411188
H	-2.8701653	2.07627767	-1.21219814
O	-5.59490883	3.66806325	0.08024062
H	-5.0758056	3.042932	-1.84880496
H	-7.52218654	2.02264937	0.77849643
H	-7.37521147	2.82353433	-0.86334599

71191235172120063000 2_R_Addition_MultipleBond_2_1_11.inp

C	1.665657	0.008996	0.096792
C	1.785876	1.480005	-0.024415
C	1.250858	2.298438	0.88202
H	2.361097	1.849103	-0.869098
C	1.352977	3.787235	0.873368
H	1.931424	4.147231	0.020865
H	1.826582	4.145047	1.792806
H	0.356382	4.238417	0.834828
O	2.316983	-0.782526	-0.569886
H	0.687925	1.856956	1.705252
H	0.107296	-0.065652	-0.733494
H	1.102105	-0.341259	0.989242

71191235172120063000 2_R_Addition_MultipleBond_9_1_11.inp

C	1.73662734	0.0064394	-0.05079614
C	1.94986865	1.47187309	-0.04568276
C	2.19841243	2.13445507	1.08431595
H	1.92721399	1.96611305	-1.01306225
C	2.46912396	3.59825097	1.18945475
H	2.44241944	4.08487065	0.21300872
H	3.44987958	3.77589186	1.64140228
H	1.73009717	4.07788051	1.8391046
O	1.72940275	-0.67493142	-1.06611771
H	2.20789225	1.57241269	2.01894261
H	0.00433452	0.12334113	0.27941326
H	1.80013221	-0.47793745	0.94824396

71191231191111057000 2_R_Addition_MultipleBond_3_5_6.inp

C	0.22042206	0.08081943	-0.31632082
C	1.5998299	-0.06041544	0.30192068
C	2.31820794	1.25695167	0.27166454
H	1.48425677	-0.43881741	1.32001892
C	3.28433827	1.5681355	-0.60125324
H	2.24820654	2.25325998	-2.29185424
H	3.69647727	0.82442905	-1.27650112
H	3.77670373	2.53320864	-0.57370445
O	-0.80695547	-0.17516572	0.24817227
H	1.96793596	2.01192742	0.97103058
H	2.15048292	-0.79830439	-0.2932874
H	0.22014715	0.46028179	-1.36005915

71195231174120057004 2_r12_insertion_R_2_1_9.inp

C	1.61023165	-0.46750498	0.14319839
C	0.27318849	-0.27263242	0.17840553
C	-0.63004083	-0.45489338	-0.96752796
H	-0.19814827	-0.062225	1.13220105
C	-0.43858037	-1.33065139	-1.95165673
H	1.06332109	1.90078051	-0.82334159
H	-1.14260163	-1.41448492	-2.77140441
H	0.41337433	-2.00339693	-1.96082564
O	0.69565562	1.90068275	0.07134705
H	-1.517368	0.17125162	-0.96739621
H	2.12476031	-0.68129985	-0.78852358
H	2.21016697	-0.37686286	1.03913703

71191231191111057000 2_Intra_R_Add_ExoTetCyclic_F_3_1_12.inp

C	1.35615889	1.05465456	-0.29347483

C	1.83184751	-0.25031615	0.406368
C	0.38970137	-0.1020895	0.53481113
H	2.20292057	-0.98983152	-0.29469483
C	-0.57987073	-0.88460772	-0.29360386
H	-0.16526885	-1.09638941	-1.27990005
H	-1.5193582	-0.34391349	-0.42326076
H	-0.79945068	-1.83680334	0.20178299
O	1.21306342	1.1063048	-1.54037636
H	0.01361949	0.35569313	1.44399967
H	2.45321659	-0.07804332	1.27934318
H	1.4475942	1.96584451	0.30985885

71195216171721200720062_intra_H_migration_3_11.inp

C	1.37306592	0.26234573	-0.06673743
C	0.1212668	-0.22422898	-0.42506003
C	-0.71970295	0.84849733	-0.874551
H	-0.20257462	-1.24088271	-0.23307445
C	-2.2145475	0.7813733	-0.70272105
H	-2.4841387	0.44875057	0.30248947
H	-2.67251338	0.08857696	-1.41896232
H	-2.66922136	1.76156797	-0.86534219
O	1.47344565	1.54212399	-0.01089609
H	-0.40283343	1.32170687	-1.81169567
H	0.21262764	1.69456277	-0.26121049
H	2.25193419	-0.33773507	0.15840281

29041017000000000002.inp

C	0.68846779	1.24709333	1.37487167
O	-0.10955803	0.75878362	2.07872877
H	1.73206624	0.85893405	1.22946256

71191231191111057 0002_R_Addition_MultipleBond_3_2_1.inp

C	1.138803	-0.066702	-0.081055
C	1.615433	2.027056	0.036193
C	1.214222	2.16162	1.326012
H	2.659704	1.853659	-0.203326
C	2.063886	1.815869	2.504111
H	1.659811	0.946029	3.035569
H	2.094271	2.641567	3.222038
H	3.086192	1.578505	2.203358
O	1.928637	-0.836642	0.344942
H	0.190119	2.467395	1.528602
H	0.986198	2.371961	-0.77679
H	0.03753	-0.114766	0.083002

71195231174120057004 2_r12_insertion_R_9_1_11.inp

C	1.24159088	0.23869745	-0.68639745
C	-0.07776196	0.22081974	-0.58743858
C	-0.94876031	-0.74555655	-1.27893409
H	-0.56996354	0.98646946	0.00877006
C	-0.66163406	-2.03319356	-1.45560805
H	2.32325278	1.24126343	1.57023429
H	-1.34028902	-2.69167946	-1.98516472
H	0.25024681	-2.47054754	-1.06148486
O	2.40791962	1.826214	0.80581007
H	-1.89512694	-0.35381724	-1.64425842
H	1.85788816	1.11596813	-0.08839234
H	1.87659691	-0.42587471	-1.26352297

71191246223109012000 2_r13_insertion_ROR_6_5_3_9.inp

C	1.76619635	-1.12445578	0.0088353
C	1.0838052	0.01276091	-0.03386028
C	1.71751811	1.36397944	-0.05347775
H	-0.00413481	0.00600605	-0.0628073
C	1.2009683	2.37478461	0.93162885
H	0.84702322	2.96798949	-0.22110893
H	1.91932438	3.01570893	1.43326076
H	0.28377411	2.13675395	1.46381694
O	1.22498309	2.19360166	-1.12747397
H	2.80834029	1.28930634	-0.0861016
H	1.26701103	-2.08631335	0.03190863
H	2.85239514	-1.13145405	0.0218484

711912351721200630002.inp

C	1.30873739	0.037382	-0.05683834
C	1.94381344	1.4001326	-0.03564075

C	2.38316512	2.09183759	-1.08429782
H	2.03077897	1.83081648	0.95858661
C	2.36156203	1.6873675	-2.5280739
H	1.84438576	0.74336046	-2.70511082
H	3.38249592	1.58400438	-2.90944395
H	1.87462372	2.45916584	-3.13144859
O	0.29339371	-0.13536144	0.83363007
H	2.81815823	3.06933935	-0.8870462
H	2.05463744	-0.72923005	0.25148185
H	1.00014498	-0.29256363	-1.06149903

71191229197113051002.inp

C	-0.00374964	-0.00970024	0.01456929
C	1.51423252	-0.00008743	-0.01019799
C	2.10663225	1.40683467	-0.00090866
H	1.80946868	-0.55229561	0.88975134
C	1.76078883	2.19690291	-1.25969042
H	0.67791735	2.31780019	-1.36183176
H	2.12646564	1.68756624	-2.1565337
H	2.20637252	3.1937819	-1.23448727
O	-0.73820648	-0.81331651	-0.43178963
H	3.1927456	1.32314996	0.10001838
H	1.84693907	-0.5799874	-0.88018542
H	1.74680024	1.93906743	0.88489827

71195216172120072006 2_intra_H_migration_suprafacial_2_11.inp

C	1.19650042	0.2231381	0.07691842
C	-0.23686358	0.09752453	-0.08100631
C	-0.88246955	-1.07794812	-0.02195399
H	-0.78610367	1.0237549	-0.2350448
C	-2.36200491	-1.24822981	-0.14050485
H	-2.86465534	-0.29191394	-0.29933023
H	-2.77468508	-1.70760276	0.76393186
H	-2.61201765	-1.9120223	-0.9743883
O	1.8492963	1.39171279	-0.24405793
H	-0.29812308	-1.98552321	0.12545241
H	1.60751204	1.03631128	0.93005832
H	1.79920365	-0.6899954	0.073712

69193300284174064 0042.inp

C	0.015051	0.00374	0.006632
C	1.482524	-0.013446	0.029693
C	2.257643	1.252261	0.019991
H	1.993803	-0.882675	-0.375483
C	3.429654	1.400308	-0.541146
H	4.158084	2.184035	-0.686111
O	0.719912	-0.27004	1.204849
H	1.782807	2.097705	0.528907
H	-0.519868	-0.8378	-0.426151
H	-0.504622	0.958986	-0.020187

71195242219113012 0002.inp

C	-0.04563326	0.24656452	0.04504659
C	1.41429215	0.0117658	-0.08779686
C	1.92782256	-0.78039814	1.0943188
H	1.61004233	-0.57171608	-1.00146133
C	2.45905731	-1.99437987	1.00135108
H	3.00531408	1.1319871	-0.12279834
H	2.8030964	-2.53326913	1.87699663
H	2.56101395	-2.49473471	0.04216264
O	2.05829155	1.27735478	-0.16201149
H	1.82832723	-0.28641938	2.05862548
H	-0.39717063	1.15929857	0.50993347
H	-0.74088809	-0.55319486	-0.17444078

71195231174120057 0042_r13_insertion_ROR_4_2_1_9.inp

C	2.44545695	1.18352746	0.24219886
C	1.65134397	0.12195118	0.11259845
C	2.09735356	-1.27754714	0.17345767
H	0.58572392	0.28698153	-0.02790222
C	3.2609191	-1.73300453	-0.28816811
H	-1.61054753	2.99610345	-0.2274686
H	3.5337325	-2.77805203	-0.19514538
H	3.96827803	-1.07700936	-0.78616081
O	-0.72056537	2.61297808	-0.16705315

H	1.39685841	-1.98290083	0.61618387
H	2.04450231	2.18775612	0.1698078
H	3.50813314	1.07743808	0.43946261
71191231191111057000 2.inp			
C	0.00699946	0.0100547	0.01048275
C	1.51322132	0.0181471	0.08458424
C	2.15826567	1.34225616	-0.09146915
H	1.78318997	-0.447529	1.04896992
C	3.63803062	1.46823652	-0.03799959
H	4.0421476	1.10551835	0.91826936
H	3.96146152	2.50234441	-0.16538088
H	4.12548724	0.86690513	-0.81966717
O	-0.68105256	0.97609132	-0.17937107
H	1.52599098	2.20279543	-0.26939909
H	1.86759362	-0.71620183	-0.66066669
H	-0.4559975	-0.99029977	0.14774934
711912462231090120002_R_Addition_MultipleBond_9_3_10.inp			
C	1.10399974	-0.77049622	0.41571969
C	1.47243655	-0.06276253	-0.64847572
C	1.41263333	1.43271596	-0.75299097
H	1.80480797	-0.54933926	-1.56066813
C	0.93977092	2.23668805	0.44680676
H	-0.14493261	2.11589058	0.53205336
H	1.15965355	3.28930385	0.27528149
H	1.39507224	1.91098902	1.38321382
O	1.48170868	1.95445016	-1.86389983
H	3.11747871	1.47505125	-0.35626489
H	1.128086	-1.85415376	0.40023591
H	0.75988687	-0.30307459	1.33222208
712193323793121330062_r12_insertion_R_5_3_9.inp			
C	-6.80678584	2.44247557	0.0509633
C	-5.57451076	1.64252562	-0.19449045
C	-4.80042441	2.60982445	-0.8588522
H	-5.13892873	1.01170788	0.57325621
C	-3.32539186	2.76682463	-0.70835475
H	-2.94583518	2.23756447	0.16636672
H	-3.08742125	3.82842901	-0.60828903
H	-2.81389782	2.4032765	-1.6056689
O	-6.00698592	3.62169781	0.21147538
H	-5.22464845	3.07082106	-1.74704277
H	-7.36834395	2.1766727	0.95143626
H	-7.49181671	2.52861337	-0.80330873
711952221681140760062.inp			
C	1.54532756	-0.0453852	-0.03551498
C	0.03951818	-0.0062084	-0.02405354
C	-0.72849231	-0.51595192	-0.95490538
H	-0.41581165	0.5143651	0.82656398
C	-2.15199008	-0.66875038	-1.28139921
H	-2.43214665	-1.72426235	-1.33498578
H	-2.38723538	-0.21200893	-2.24651218
H	-2.77722702	-0.18870068	-0.51486626
O	2.11138771	1.25572697	-0.0395257
H	1.77230819	1.72206743	-0.80551239
H	1.89487651	-0.63555446	-0.89072763
H	1.91645996	-0.51906873	0.87630264
712193323793121330062_r12_insertion_R_2_3_9.inp			
C	-6.82672663	2.35251233	-0.07548455
C	-5.60834572	1.6700871	-0.59377813
C	-4.77642952	2.78872175	-0.77412464
H	-5.22727552	0.75920705	-0.14412631
C	-3.30573372	2.80121139	-0.52933777
H	-3.03992351	3.71585026	0.00580541
H	-2.76528977	2.81423692	-1.48158358
H	-2.98295642	1.94243496	0.06026188
O	-6.00100136	3.32206056	0.58333332
H	-5.14577068	3.59460716	-1.40299002
H	-7.43661664	1.76433571	0.61657299
H	-7.46893651	2.8151638	-0.83705761
711912311911110570002_r12_insertion_R_4_2_1.inp			
C	1.1383294	-0.00434817	-0.00769575

C	1.85000069	1.29169791	0.0231717
C	1.47439805	2.2535443	0.89169383
H	3.43659746	0.46391047	0.88246099
C	2.02969819	3.63423552	0.93477315
H	2.54908171	3.80280962	1.88471016
H	1.22743731	4.37645573	0.87917272
H	2.7322396	3.81469436	0.11961666
O	1.23822586	-0.81302322	-0.89381055
H	0.73782766	1.99722852	1.65269001
H	2.51206973	1.50492229	-0.81139474
H	0.46767841	-0.18632736	0.85936738
2706304100400000000002.inp			
C	0.12464882	-2.48147552	0.93523775
C	-1.06341546	-2.1273543	0.52939488
H	-1.65188462	-2.17714276	-0.37617652
H	0.83226294	-2.98230543	0.26990957
H	0.46617532	-2.29575599	1.95132631
711912351721200630002_intra_H_migration_suprafacial_3_11.inp			
C	1.66662833	1.59696137	-0.01579403
C	1.02355345	0.25355712	0.12811199
C	-0.24973825	-0.04165502	-0.27552596
H	1.66343588	-0.50184585	0.57851362
C	-0.89062604	-1.37891946	-0.11412272
H	-0.20627772	-2.10370693	0.33137061
H	-1.22662912	-1.77167934	-1.07923214
H	-1.77953309	-1.31093598	0.52384386
O	2.88992808	1.79688635	0.14199353
H	-0.84301592	0.74653019	-0.73522972
H	1.06157949	1.36616867	1.09337592
H	1.01765531	2.35150209	-0.49794227
711952311741200570042_intra_H_migration_5_6.inp			
C	1.67129194	0.41032904	0.08691665
C	0.18236996	0.16068535	0.00792161
C	-0.69970687	1.14690549	-0.08668409
H	-0.12822158	-0.87904021	0.03307668
C	-0.27714023	2.56988548	-0.12121548
H	0.97176098	2.42641342	-0.04034468
H	-0.57144043	3.16866765	0.74254477
H	-0.46347384	3.09286916	-1.06089506
O	2.02441152	1.75911231	0.05121055
H	-1.76297684	0.91859871	-0.14099628
H	2.14775453	-0.11605476	-0.75416693
H	2.04104085	-0.04114764	1.02017826
711912311911110570002_r12_insertion_R_4_2_3.inp			
C	1.13832893	-0.00432576	-0.00768659
C	1.85003961	1.29169868	0.02318126
C	1.47448546	2.25354519	0.89172422
H	3.4366313	0.46385293	0.88242391
C	2.02982781	3.63421928	0.93480851
H	2.73235617	3.8146675	0.11963843
H	2.54923782	3.80276584	1.88473592
H	1.22758784	4.37646413	0.87923562
O	1.23818121	-0.81299248	-0.89381398
H	0.73792472	1.99724171	1.65273389
H	2.51209605	1.50491395	-0.8113975
H	0.46769216	-0.18629602	0.8593894
711912462231090120002_intra_H_migration_9_12.inp			
C	1.85377034	1.13822065	-0.48450788
C	1.58110554	0.00437697	0.13499167
C	0.10200174	-0.08528732	0.3891304
H	2.29235084	-0.76457023	0.42173272
C	-0.56689927	-1.28443898	-0.26630492
H	-0.3680434	-1.28481542	-1.34051107
H	-1.64703411	-1.24565733	-0.11027024
H	-0.19074996	-2.21690976	0.16546576
O	-0.40456289	1.13324173	-0.15179031
H	-0.09290493	-0.09331697	1.47049172
H	2.73596011	1.63808637	-0.85928239
H	0.65234921	1.61454187	-0.56598271
711912351721200630002_r12_insertion_R_1_2_4.inp			

C	1.07884707	0.11424265	-0.05705377
C	1.05694452	2.37047377	0.12893724
C	0.81451493	2.98075854	-1.00061214
H	1.25677429	2.63141544	1.15943408
C	0.74122239	4.47596271	-1.1707278
H	0.91611195	5.00218991	-0.2309676
H	-0.24272712	4.76169189	-1.55301386
H	1.48583814	4.80697312	-1.90004642
O	0.84916482	-0.10530785	-1.23215452
H	0.65250692	2.36999177	-1.88832836
H	0.27546331	0.09007906	0.70221044
H	2.11274028	0.13850075	0.33407109
711952161721200720062.inp			
C	0.73294651	0.67915283	0.06058485
C	0.31045766	-0.61677964	0.26694199
C	-0.9844	-1.07515821	0.07699564
H	1.05525608	-1.33664288	0.60647831
C	-2.11014113	-0.20485772	-0.38509721
H	2.55459193	0.38759797	0.54776107
H	-2.2845253	0.63062928	0.30494519
H	-3.0412604	-0.76736493	-0.46479935
O	2.00277994	1.1146747	0.25037839
H	-1.18801267	-2.12107666	0.27482797
H	-1.90285861	0.23588666	-1.36866877
H	0.08219398	1.4754406 -0.27542208	
441041030570000000001.inp			
C	0.56674238	1.29130353	-0.19700478
C	-0.68267289	1.75395669	0.49411383
H	-1.26361701	2.41137244	-0.15217724
H	-0.41366501	2.27508001	1.41848453
H	-1.28076997	0.88357697	0.78242363
O	0.88637013	1.60204093	-1.31302755
H	1.21522136	0.61457042	0.40002658
711912462231090120002_r12_insertion_R_3_5_6.inp			
C	1.79390485	-1.11560309	-0.03378783
C	1.08612523	0.00696402	0.02882426
C	1.72445496	1.3539488	0.019263
H	-0.00018941	0.00331857	0.04329831
C	1.87551973	1.65573304	2.1134528
H	0.82943503	1.5972155	2.38902129
H	2.32160251	2.64257798	2.12200995
H	2.50487017	0.8212083	2.40054269
O	1.10138349	2.36829768	-0.31192504
H	2.83151438	1.33834955	-0.03979462
H	1.32107312	-2.09007428	-0.06841462
H	2.88055948	-1.093528 -0.06166004	
711912462231090120002_r12_insertion_R_5_3_2.inp			
C	2.00682449	-0.82321381	0.19464111
C	1.11292877	0.02710132	-0.22953962
C	1.65551603	2.1109747	-0.04604441
H	0.12309686	-0.02412016	-0.66256356
C	1.28974878	2.58125638	-1.43742593
H	1.9931855	2.18371617	-2.17031436
H	1.36153306	3.67408258	-1.44872796
H	0.26777865	2.30791945	-1.70857732
O	2.82071764	2.035011	0.33228455
H	0.84142874	2.1589697	0.70634241
H	1.82201756	-1.89580712	0.15160104
H	2.95464776	-0.48075413	0.59949972
712153574202750690002.inp			
C	0.47845213	0.84937952	-0.00426449
C	1.00556373	-0.46648577	0.68794958
C	-0.43126317	-0.27024646	0.79113418
H	1.37576453	-1.19340299	-0.02528164
C	-1.41025309	-1.06902642	-0.0181451
H	-0.99522093	-1.31727993	-0.9955162
H	-2.33972139	-0.51685205	-0.17049681
H	-1.64602752	-2.00039055	0.50644312
O	0.37839334	0.89417974	-1.26549347
H	-0.8025604	0.16370166	1.71461397

H 1.63537038 -0.27880978 1.55037295
H 0.6241094 1.76250204 0.58317392
54150158112060006001.inp
C -1.591265 -0.229392 -0.288285
C -0.648076 0.783231 -0.638693
C 0.625297 0.606357 -0.199942
H -0.879509 1.695154 -1.199032
C 1.754464 1.55042 -0.381148
H 1.471229 2.434501 -0.953474
H 2.583781 1.041891 -0.884326
H 2.136907 1.862274 0.596798
H 0.815952 -0.316052 0.34611
H -2.59243 0.104357 -0.627031
18034002000000000001.inp
H 2.24930093 1.81325024 2.38607303
O 2.14892304 2.25728506 1.54362051
H 1.75685803 1.6057847 0.96154946
71195216172120072006 2_R_Addition_MultipleBond_3_5_6.inp
C 1.83306808 -1.14899336 -0.10238466
C 1.14035379 -0.00450294 -0.12761873
C 1.78023501 1.29241262 -0.03498597
H 0.05537903 -0.03018391 -0.21680117
C 1.13186054 2.46648919 -0.03848139
H 0.69419563 2.82356633 2.17583659
H 0.05462265 2.51789154 -0.16080716
H 1.66921622 3.40556755 0.00421148
O 1.32767117 -2.39763449 -0.18063482
H 2.86437211 1.29180669 0.0591924
H 0.37089304 -2.35999755 -0.25471899
H 2.91387307 -1.17007087 -0.0133218
711952311741200570042_intra_H_migration_3_11.inp
C 1.805534 1.374422 0.078182
C 1.088839 0.263466 0.361137
C -0.254169 0.002923 -0.140271
H 1.535087 -0.49201 1.005044
C -1.15512 0.919258 -0.505476
H 3.311438 1.001858 1.184055
H -2.11865 0.624251 -0.903544
H -0.967993 1.983101 -0.399198
O 3.064805 1.624934 0.496572
H -0.528229 -1.04751 -0.205871
H 0.719605 2.559644 1.349738
H 1.490655 2.087326 -0.674253
71191231191111057000 2_intra_H_migration_9_6.inp
C -0.408881 1.275356 -0.154308
C 0.200139 -0.017146 0.354549
C 1.513685 0.094874 -0.333822
H 0.302067 0.004745 1.446267
C 2.391982 1.077448 0.113672
H 1.383145 2.050709 0.071777
H 3.267536 1.314558 -0.482225
H 2.52499 1.187922 1.1922
O 0.18385 2.377749 0.068327
H 1.584927 -0.292453 -1.345961
H -0.394588 -0.874766 0.044537
H -1.211576 1.241219 -0.893347
71191231191111057000 2_intra_H_migration_1_4.inp
C 0.102331 -0.059273 0.053433
C 1.596339 0.083779 0.049928
C 2.238103 1.263771 -0.199555
H 0.647609 0.088534 1.193897
C 3.719323 1.428185 -0.200984
H 4.236777 0.488737 0.005596
H 4.02698 2.162426 0.552546
H 4.067822 1.808957 -1.166583
O -0.679608 0.87815 -0.219326
H 1.620032 2.131342 -0.413752
H 2.149451 -0.828133 0.263707
H -0.239824 -1.108157 0.067196
71191242197113038000 2.inp

C 0.02420845 -0.02381223 -0.39131741
C 1.3956636 0.06802829 0.22043522
C 1.93808609 1.48677842 0.3519108
H 1.34468483 -0.43032546 1.19840808
C 3.27739103 1.54248819 0.99412384
H 1.21298101 2.08668549 0.92422305
H 3.89556545 2.42685344 0.90038283
H 3.60026988 0.76836728 1.68185804
O -0.63198604 0.9161045 -0.75380907
H 1.96659352 1.9616129 -0.63499791
H 2.06368899 -0.55954575 -0.3842896
H -0.3722006 -1.05661893 -0.49675845
20020000000000000001.inp
H -0.349377 -2.135744 3.379022
H -0.496811 -2.135119 2.649372
711952161721200720062_R_Addition_MultipleBond_2_1_9.inp
C 0.779114 0.475078 0.210285
C 1.682046 1.518006 -0.091486
C 2.976613 1.363765 0.311176
H 1.417732 2.407731 -0.669275
C 4.091016 2.287051 0.004043
H 3.770462 3.168233 -0.552665
H 4.857082 1.75402 -0.570049
H 4.576364 2.595935 0.936151
O 2.261207 -1.616253 1.271651
H 3.204947 0.464913 0.885192
H 1.442804 -1.059557 1.244426
H -0.189449 0.624561 -0.293136
711912351721200630002_intra_H_migration_2_11.inp
C -1.546183 0.139249 0.269658
C -0.101501 -0.237861 0.367861
C 0.814775 -0.057155 -0.631706
H 0.18365 -0.667788 1.325358
C 2.250233 -0.450591 -0.533082
H 2.488594 -0.866405 0.447938
H 2.905312 0.408447 -0.710632
H 2.502256 -1.199905 -1.292545
O -2.316288 0.182959 1.252697
H 0.48093 0.392093 -1.565122
H -1.2434 -1.064216 -0.061429
H -1.821217 0.599057 -0.697761
711952161721200720062_Intra_R_Add_ExoTetCyclic_F_3_1_12.inp
C 1.45377361 1.26395754 -0.1292016
C 1.75315862 -0.07446143 -0.3152601
C 0.38994375 -0.25309219 -0.83777534
H 2.12101293 -0.68012419 0.51060391
C -0.65382068 -0.97867021 -0.05086556
H -0.57651249 -0.74533997 1.01717302
H -0.5454279 -2.06908994 -0.14383611
H -1.66374122 -0.72061582 -0.38159031
O 1.50174648 1.95636125 1.0272295
H 0.17301024 0.00613442 -1.87025917
H 1.75772255 1.36274397 1.73873981
H 1.21827298 1.92929657 -0.95217419
711912462231090120002_intra_H_migration_suprafacial_1_10.inp
C -0.08236984 -0.30390077 -0.39983617
C 1.02061746 0.18465018 0.23348615
C 1.77105218 1.44635258 -0.10933433
H 1.4713316 -0.35105283 1.06401243
C 1.32771549 2.22582141 -1.33408274
H 1.66639523 1.69925842 -2.23122929
H 1.79418646 3.2098964 -1.30926543
H 0.24382021 2.34419191 -1.39696042
O 2.8913993 1.6561493 0.42044046
H 0.85472645 1.53997494 0.78241955
H -0.52050899 -1.23944525 -0.07667107
H -0.54417756 0.20401394 -1.23746069
701821991571050530041.inp
C 1.77247632 -1.18147507 -0.01917757
C 1.13716306 -0.03134926 -0.26141061

C 1.7906912 1.26261748 -0.14253496
H 0.08996641 -0.04381429 -0.56033417
C 1.20276502 2.43777223 -0.37255544
H 0.3289446 -2.3745143 -0.36943769
H 1.74491539 3.36949432 -0.26650947
H 0.16149838 2.50203943 -0.67381398
O 1.2501412 -2.42505183 -0.10322248
H 2.83696193 1.24647643 0.15760575
H 2.81431697 -1.21624108 0.28070277
70178203202105012000l.inp
C 1.770708 -1.135832 0.000006
C 1.082946 0.001403 0.000028
C 1.772166 1.32968 -0.000029
H -0.003666 0.000002 0.000087
C 0.872367 2.541629 0.000002
H 0.222154 2.522062 -0.880322
H 1.468217 3.452767 -0.00004
H 0.222243 2.522085 0.880392
O 2.978416 1.423196 -0.000095
H 1.27698 -2.100795 0.000047
H 2.855966 -1.113845 -0.000054
71195216172120072006 2_R_Addition_MultipleBond_1_9_11.inp
C 1.38509436 -0.02429153 -0.11229101
C -0.06391973 0.03565596 -0.03996746
C -0.80914854 -1.07704204 -0.02864713
H -0.5139912 1.02399179 0.00087878
C -2.29835746 -1.1199644 0.03317575
H -2.73054796 -0.11901676 0.07975966
H -2.62900699 -1.68522316 0.91029768
H -2.70308175 -1.63486514 -0.84404331
O 2.1124843 0.97240899 -0.11872671
H -0.29764713 -2.03867833 -0.07184909
H 2.58645077 1.62364442 1.22975901
H 1.83587301 -1.03217433 -0.13543136
71191235172120063000 2_R_Addition_MultipleBond_3_5_6.inp
C 2.52145 3.732531 -0.073622
C 2.360303 2.244733 -0.162662
C 1.171659 1.614045 -0.146102
H 3.274143 1.657423 -0.220484
C 1.137223 0.168473 -0.190195
H 1.285076 -0.337199 1.871987
H 0.111475 -0.202949 -0.03135
H 1.715422 -0.487514 1.236352
O 3.562991 4.237461 -0.785841
H 0.261446 2.220537 -0.119505
H 2.7532 4.009487 0.9791
H 1.580837 4.267305 -0.289846
71195216172120072006 2_beta_delta_3_2_1_9_11.inp
C 2.41979947 1.95508927 0.91697638
C 2.50759099 0.78451293 0.06006966
C 1.4992252 -0.05774726 -0.21492656
H 3.48770998 0.60816477 -0.37296514
C 0.0876355 -0.00575706 0.2753932
H -0.12364478 0.84533664 0.92135243
H -0.14629835 -0.92074562 0.8286734
H -0.5996071 0.03016612 -0.57554501
O 3.38824846 2.6877097 1.14076882
H 1.72204805 -0.89449419 -0.87380147
H 3.73733761 3.88684054 0.16081397
H 1.44497727 2.20788682 1.36099472
71195216172120072006 2_Intra_R_Add_ExoTetCyclic_F_3_1_9.inp
C 1.86866856 0.95292663 -0.03496281
C 1.44126096 0.10366282 -1.04079742
C 0.10115493 0.14658732 -0.4361887
H 1.94640971 -0.84375819 -1.21810228
C -0.46080139 -1.03337244 0.28980281
H 0.31331104 -1.54208163 0.8755345
H -0.87025137 -1.77948666 -0.40666574
H -1.26608327 -0.74477164 0.97108311
O 2.85559879 0.7281914 0.85639569

H	-0.55577693	0.98576423	-0.64713804
H	3.21942968	-0.1491856	0.70808423
H	1.48229531	1.95902729	0.08295489

711912351721200630002_r12_insertion_R_11_1_2.inp

C	1.83179009	-0.00097107	-0.00275183
C	1.9934149	1.47104379	-0.00889636
C	2.01556758	2.17292716	1.124487
H	2.12727955	1.93763905	-0.98099427
C	2.21596424	3.64818588	1.2268695
H	2.34804195	4.10669148	0.2454001
H	3.09424978	3.87487898	1.83918073
H	1.35796057	4.11720638	1.71890404
O	2.02734284	-0.70939906	-0.98003177
H	1.87677484	1.63725182	2.06432124
H	0.06556286	0.05959485	0.01174624
H	1.73199909	-0.45535617	1.00734312

711912351721200630002_intra_H_migration_9_10.inp

C	0.02511529	0.01928073	0.25512732
C	1.26498084	0.07061435	-0.58870359
C	1.85000022	1.24985637	-0.44690261
H	1.61513968	-0.74514845	-1.2150597
C	3.04787574	1.94329421	-0.96103279
H	3.72354088	2.22234538	-0.14819735
H	2.77705876	2.85439102	-1.50107191
H	3.59103889	1.28484546	-1.64865973
O	-0.02739165	1.28677835	0.88415044
H	1.01537198	1.77215783	0.38141036
H	0.07976025	-0.77509556	1.01053222
H	-0.87487071	-0.13790064	-0.35313017

421261230750120000001.inp

C	0.69110697	-0.159225	-1.5556333
C	0.07831194	-1.12305934	-0.87843563
H	0.15992849	0.73154917	-1.87967305
C	-1.36708936	-1.11465523	-0.48552362
H	-1.47540006	-1.17965557	0.6018386
H	-1.890838	-1.97731629	-0.90957416
H	-1.86875639	-0.20626638	-0.82704771
H	0.65209766	-1.99710288	-0.57492896
H	1.74310975	-0.22537849	-1.80998517

711912312361110120002.inp

C	0.00773028	-0.00407359	0.16629703
C	1.4581256	0.07194268	-0.12449005
C	2.01731445	1.48460406	-0.21913291
H	1.70091525	-0.41583649	-1.08494395
C	3.5240888	1.59339326	-0.27443339
H	3.92491652	1.43298012	0.731885
H	3.81568013	2.58555757	-0.61615686
H	3.95490663	0.82561636	-0.9222445
O	1.30618985	2.45803514	-0.2443911
H	2.0545008	-0.48825482	0.61080514
H	-0.59188717	0.89631538	0.16464652
H	-0.46244764	-0.96379116	0.3370637

711912351721200630002_intra_R_migration_9_3.inp

C	1.3591859	-0.08818258	-0.16733013
C	1.4929673	1.28576211	0.39216144
C	0.18923493	1.46071594	0.88800037
H	1.9929807	2.07967574	-0.15260923
C	-0.56209594	2.74835447	0.86512161
H	-0.11103007	3.4719574	0.18524174
H	-0.61024831	3.17460377	1.87259137
H	-1.58921449	2.55741879	0.54544448
O	-0.00398593	0.18839461	-0.51551065
H	-0.18569996	0.72088005	1.5904407
H	1.43488135	-0.90495546	0.56317336
H	1.98968198	-0.3063311	-1.03435893

711912462231090120002_intra_H_migration_2_10.inp

C	1.194988	-0.751042	0.451169
C	1.479026	-0.003499	-0.651933
C	1.451576	1.500441	-0.75172
H	1.737054	-0.475426	-1.595652

C 0.978193 2.283837 0.459197
H -0.110476 2.202699 0.530313
H 1.242381 3.331738 0.32233
H 1.408793 1.924737 1.396442
O 1.52393 2.041029 -1.884128
H 2.577669 0.957973 -0.466635
H 1.223834 -1.831539 0.393521
H 0.923636 -0.305686 1.400328
71191235172120063002_r12_insertion_R_2_1_11.inp
C 1.08579213 0.11389187 -0.06038092
C 1.05584164 2.36981531 0.12817624
C 0.8182641 2.98071582 -1.00207118
H 1.24870813 2.63011594 1.16016143
C 0.74134007 4.47589095 -1.17083788
H 0.90906082 5.00152242 -0.22943949
H -0.241226 4.75903127 -1.55857094
H 1.48920203 4.81008369 -1.89536817
O 0.8638703 -0.10491123 -1.23711138
H 0.66345787 2.37052788 -1.89147004
H 0.27792153 0.08633898 0.69399218
H 2.1172295 0.1408258 0.33700311
711952161721200720062_intra_H_migration_3_6.inp
C 1.64465766 -1.13560843 -0.60295161
C 1.10583891 -0.05972555 -0.00004507
C 1.5618329 1.3002632 -0.17187054
H 0.27688405 -0.2071243 0.68984857
C 2.77342946 1.68447776 -0.93011781
H 2.62263102 1.6315267 0.42310581
H 2.88233592 2.70405205 -1.27107527
H 3.48000539 0.93605611 -1.25947763
O 1.26357579 -2.426224 -0.40975578
H 0.84723635 2.09563943 0.00904356
H 0.54999412 -2.45930652 0.23109046
H 2.44890815 -1.07287012 -1.32499831
712193284022841360122.inp
C 0.7906098 -0.92129072 -0.72174032
C 1.22970675 0.14439173 0.23744185
C -0.26911597 0.16247689 0.60988765
H 1.92417677 -0.15333112 1.02627533
C -1.02990662 1.45179811 0.43946841
H -0.8156461 1.89696011 -0.53541868
H -0.73842329 2.16036277 1.22023581
H -2.10574867 1.27754899 0.51447365
O -0.55633881 -0.78322783 -0.46266677
H -0.48827084 -0.30848997 1.57201478
H 1.57953691 1.08040738 -0.21379687
H 1.11119005 -1.19205937 -1.72155489
711912462231090120002_r12_insertion_R_5_3_10.inp
C 1.79363923 -1.11577139 -0.03349162
C 1.0858934 0.00680415 0.02934275
C 1.72420337 1.35379159 0.0190265
H -0.00041098 0.00315421 0.04456508
C 1.87674699 1.65606018 2.11304541
H 0.83085263 1.59764622 2.38935746
H 2.32286974 2.64289174 2.12105162
H 2.50626874 0.82158222 2.399895
O 1.1008851 2.36806186 -0.31194639
H 2.83122051 1.33818994 -0.04081574
H 1.32079235 -2.09025486 -0.06756096
H 2.88027421 -1.09369375 -0.06211946
711912462231090120002_intra_H_migration_9_6.inp
C 1.76624527 -1.12447072 0.00870103
C 1.08379615 0.01272718 -0.03356666
C 1.71744952 1.36397317 -0.05322825
H -0.00415507 0.00593765 -0.06208017
C 1.20131638 2.37456554 0.9323136
H 0.84681085 2.96797268 -0.22015162
H 1.91987638 3.01542786 1.43373232
H 0.28437669 2.13639719 1.46487832
O 1.22438485 2.19378498 -1.12683639

H 2.80825929 1.28934875 -0.08637086
H 1.26710323 -2.0863491 0.03184181
H 2.85244936 -1.13143232 0.02128471
711952311741200570042_intra_R_migration_2_9.inp
C 1.6047094 -0.43453997 0.16004721
C 0.26544953 -0.25294811 0.17762552
C -0.62474004 -0.47385656 -0.97170129
H -0.21727127 -0.02324446 1.12122073
C -0.41460392 -1.37196436 -1.9315773
H 1.04238432 1.90243623 -0.87210569
H -1.10985347 -1.48402491 -2.75542787
H 0.44449025 -2.03544985 -1.91588621
O 0.66664278 1.92122895 0.01902465
H -1.51863522 0.14241469 -0.99563703
H 2.13045627 -0.66662765 -0.7609692
H 2.19492837 -0.31466002 1.0589985
711952271761240550042.inp
C 1.77795939 -1.15701189 0.07404455
C 1.14958008 0.01074395 0.16836561
C 1.83727439 1.33732707 -0.00572305
H 0.0783001 0.03246121 0.36462925
C 1.32591908 2.12377129 -1.16474975
H 2.92026695 1.16211543 -0.11946764
H 1.41810646 3.20339215 -1.18680181
H 0.9781699 1.61613011 -2.05711491
O 1.22673725 -2.39072334 0.20902774
H 1.73942205 1.93336647 0.91046109
H 0.28682447 -2.30524907 0.38395891
H 2.84162788 -1.23059294 -0.12736333
711912462231090120002_r12_insertion_R_3_2_4.inp
C 2.00604281 -0.82310754 0.19415222
C 1.11203674 0.02732418 -0.22956091
C 1.65467716 2.1111464 -0.0456361
H 0.12209012 -0.02377727 -0.66233661
C 1.28844285 2.58184258 -1.43675421
H 1.99164916 2.18454216 -2.16999372
H 1.36019769 3.67467398 -1.44774602
H 0.26638992 2.30856558 -1.70765356
O 2.8200065 2.03507104 0.33227917
H 0.84084299 2.15891904 0.70703825
H 1.82121992 -1.89568867 0.15087692
H 2.95397739 -0.48075986 0.59884507
711952442360980080002.inp
C -0.77271164 -0.03889277 -0.94790749
C 0.0182532 0.23806936 0.05396241
C 1.52796559 0.12749433 0.02417044
H -0.39951249 0.59774511 1.00146398
C 2.04247157 -0.81136967 1.10074932
H 1.73446385 -0.45860602 2.08860644
H 3.13332138 -0.84738006 1.07197351
H 1.64907272 -1.81849257 0.94705576
O 2.11506291 1.39992973 0.26979771
H 1.83283311 -0.24398966 -0.96205439
H 1.79307707 2.01331005 -0.39302878
H -1.83083061 -0.04035884 -1.16636914
561281360900380000001.inp
C 1.7978356 -1.12466901 -0.00002462
C 1.07882993 -0.00506848 -0.00001256
C 1.73910078 1.31471837 -0.00000021
H -0.00661266 -0.00871536 -0.00001121
O 1.15159666 2.36628309 0.00001121
H 2.84991501 1.28141117 -0.00000212
H 1.33659608 -2.10538055 -0.00003382
H 2.88432237 -1.09122512 -0.00002565
711952311741200570042_intra_H_migration_9_11.inp
C 1.238108 -0.019285 -0.321459
C -0.039366 0.183375 -0.599733
C -0.975721 -0.868563 -1.033154
H -0.445373 1.187547 -0.495498
C -0.66039 -1.882334 -1.835693

H 2.216834 1.763689 1.458569
H -1.394754 -2.631701 -2.107273
H 0.333561 -1.982019 -2.260101
O 2.481028 1.93653 0.545221
H -1.996692 -0.771175 -0.671189
H 1.92937 0.953594 -0.037792
H 1.777352 -0.960894 -0.308284
71191235172120063002_Intra_R_Add_Endocyclic_F_9_3.inp
C 1.36086032 -0.09608376 -0.14450157
C 1.49272884 1.27707362 0.4173766
C 0.17897227 1.46326232 0.88174583
H 2.0122694 2.06642819 -0.11561622
C -0.56096726 2.75700244 0.83986434
H -0.08669075 3.47690939 0.1719565
H -0.6317968 3.18365263 1.84581161
H -1.58096441 2.57431464 0.49363176
O 0.00867906 0.19154374 -0.5251371
H -0.2188176 0.72716574 1.575493
H 1.41239078 -0.91301301 0.58792595
H 2.00999355 -0.31996225 -0.99618457
711952311741200570042_intra_H_migration_2_6.inp
C 1.55999719 0.07684467 -0.21520143
C 0.0578256 0.03886589 -0.26828511
C -0.73840174 -1.12482504 0.12318414
H -0.38650557 0.70457124 -1.00586957
C -0.26961838 -2.31277023 0.5182111
H 0.1966856 0.83400999 0.75695156
H -0.94810828 -3.10747373 0.80457401
H 0.79091177 -2.53173038 0.57184715
O 1.50572717 0.9787431 0.88905102
H -1.81437667 -0.96933387 0.10867049
H 2.03944794 0.51852107 -1.09224162
H 2.04858492 -0.86560471 0.04444515
10000000000000000002.inp
H 0.65999 2.850779 2.649396
711912351721200630002_intra_H_migration_9_11.inp
C -1.45487971 -0.1284263 0.00008842
C -0.0610203 -0.46990987 0.18927223
C 0.91628777 -0.04866584 -0.62893096
H 0.16969254 -1.08362778 1.0572643
C 2.36961873 -0.34442294 -0.44886589
H 2.54669108 -0.96655559 0.43091714
H 2.94420688 0.58112415 -0.33686155
H 2.77298123 -0.8627232 -1.324742
O -2.44901649 -0.77964887 0.69454042
H 0.64614462 0.5576404 -1.49281228
H -2.05605189 0.37458768 0.97149073
H -1.74749046 0.34850816 -0.94012455
150390060000000000002.inp
C -1.50796536 2.13978845 1.30904562
H -1.9478067 1.17274245 1.50968066
H -1.05260522 2.33770463 0.34864416
H -1.52397572 2.90905247 2.06857256
711912462231090120002_R_Addition_MultipleBond_9_3_2.inp
C 1.86503222 -0.84584416 -0.30934463
C 1.20137254 -0.04236461 0.47551617
C 1.72827418 2.04801654 0.32173735
H 0.42711901 -0.1381885 1.22467771
C 0.34000444 2.64259938 0.22157182
H -0.10603587 2.40626966 -0.74548394
H 0.43783194 3.73071364 0.29895232
H -0.31150732 2.30454618 1.03022467
O 2.50617521 2.04206943 -0.62767985
H 2.12503448 1.92587166 1.35070928
H 1.67687276 -1.91866295 -0.29841806
H 2.6190908 -0.46317309 -0.99090857
712193323793121330062.inp
C 0.8362589 -0.99256785 -0.624384
C 1.16493023 0.15066818 0.27271557
C -0.28695403 0.22170932 0.62743881

H	2.07086063	0.50475275	0.74307886
C	-1.06329698	1.48583757	0.31135074
H	-0.85394479	1.81895527	-0.70727245
H	-0.78471038	2.28097034	1.00883332
H	-2.13563618	1.29888509	0.4083053
O	-0.56220473	-0.85178917	-0.30787958
H	-0.51025035	-0.10598876	1.65508172
H	1.2308285	-1.9728183	-0.3185783
H	1.02788818	-0.86009845	-1.69789898

S4.1.3 R3

71215357465230024000 2.inp

C	-1.12091565	-0.48362837	0.62504526
C	-0.44474605	-1.22953724	-0.42744198
C	0.1502846	0.20604491	-0.05484622
C	1.43331124	0.23391766	0.74989843
H	1.52242878	-0.62699447	1.41675998
H	1.46807758	1.14578507	1.35085527
H	2.29006151	0.24016924	0.07182849
O	-0.11086926	1.13991147	-0.88438858
H	-0.96041413	-0.75982795	1.66083215
H	-2.04878503	0.02292163	0.39694882
H	-0.88486355	-1.26109662	-1.41479359
H	0.21012497	-2.05124731	-0.16130103

ketenets.inp

C	-0.048463197	-0.224357821	0.34810971
C	1.286248489	-0.058433694	-0.287531875
C	1.852370522	1.963173041	-0.062015363
C	2.422212972	2.290291044	-1.217736905
H	2.773976342	3.311136698	-1.308228117
H	2.542488055	1.60383428	-2.036498807
O	1.487499686	2.177570041	1.046406818
H	-0.810815063	0.372752714	-0.156618434
H	-0.353327472	-1.277409133	0.277960253
H	2.153123111	-0.447093135	0.238777479
H	1.338032545	-0.120602047	-1.36937383
H	-0.025601989	0.049701013	1.403348073

Htrans.inp

C	0.702623	0.154056	0.774292
C	1.390795	0.345018	-0.559526
C	2.760899	0.960759	-0.229628
C	2.697716	1.630646	1.112328
H	1.611153	0.886067	1.476864
H	2.270075	2.634384	1.08469
H	3.590719	1.560776	1.72912
O	3.744622	0.866971	-0.913612
H	0.758168	-0.844045	1.204718
H	-0.263193	0.631549	0.919923
H	0.853956	1.052961	-1.199469
H	1.538587	-0.56953	-1.136324

712193173963061480122.inp

C	-6.6898928	2.42524405	0.10445176
C	-5.60081518	1.38949383	-0.22233961
C	-4.79768249	2.61473721	-0.57008592
C	-3.36294675	2.92974202	-0.39243061
H	-3.0700139	2.9222358	0.66997243
H	-3.13433287	3.92090655	-0.79319624
H	-2.74740584	2.19592015	-0.91813573
O	-5.75692082	3.51010565	-0.11272545
H	-7.06356144	2.43554031	1.12952983
H	-7.52066749	2.47505823	-0.60251256
H	-5.81538748	0.68793916	-1.03078803
H	-5.23849944	0.83251195	0.65052818

71191231236111012000 2_r12_insertion_R_3_2_11.inp

C	1.80490669	-0.48010516	1.10189626
C	1.10373602	0.0155416	0.0487911
C	1.07350152	2.26102145	0.04131014
C	1.83352991	2.82317222	1.20178191

H 1.39653839 2.42530199 2.1205672
H 1.81703229 3.91524503 1.20101007
H 2.85805067 2.44712333 1.1521828
O 0.49696174 2.81710077 -0.82339918
H 2.87329441 -0.65558063 1.03834855
H 1.32997817 -0.67292738 2.05769422
H 0.01895339 0.04041308 0.07036067
H 1.54874855 0.05763897 -0.94016254
7121931739630614801 22_r13_insertion_ROR_3_2_1_8.inp
C 1.78375463 1.49929024 0.22265835
C 1.43968145 0.09822036 -0.26256791
C 0.00593722 0.48834876 -0.42609668
C -1.2227612 -0.31845348 -0.58999881
H -1.48680857 -0.46498985 -1.64548628
H -2.05626973 0.20947557 -0.11856678
H -1.1161279 -1.31197394 -0.13816246
O 0.00978717 1.76840587 -0.23088768
H 2.33672547 2.16889928 -0.42402004
H 1.89645353 1.67217905 1.28728173
H 1.60158222 -0.71357333 0.45802809
H 1.97703787 -0.16462823 -1.18252739
71191231236111012 0002_Intra_R_Add_ExoTetCyclic_F_1_3_4.inp
C 0.61908593 0.42824223 0.3699815
C 1.37483934 -0.21906278 -0.69543703
C 2.02161043 1.16263699 -0.43058463
C 3.24617061 1.21313265 0.45941981
H 3.30664652 0.361818 1.14083774
H 3.24583966 2.13843117 1.03844911
H 4.13738937 1.21076961 -0.17465154
O 1.7062061 2.13112976 -1.15795124
H 0.82601452 0.21019366 1.40980991
H -0.27740315 0.98367316 0.13508286
H 0.90992227 -0.28113607 -1.67234908
H 2.00069039 -1.06481961 -0.4263539
43091073051000000 0002.inp
C 0.2806128 1.11879455 1.51197614
C 1.6190798 0.89104785 0.85597133
H 1.52779449 0.22975888 -0.01061114
H 2.29793188 0.46304891 1.59610489
H 2.02703951 1.85919287 0.55913299
O -0.77121747 0.68926294 1.20702379
ketene.inp
C 2.120499 2.436522 -0.10168
C 2.168751 2.316795 -1.402747
H 2.603975 3.109666 -1.992683
H 1.772979 1.426716 -1.868725
O 2.077907 2.542207 1.046794
ethyl.inp
C -12.084648 3.218826 0.000649
C -10.619918 3.463892 0.033105
H -12.402157 2.550998 0.806527
H -12.402198 2.783993 -0.951359
H -12.655077 4.152905 0.124468
H -10.082749 3.498827 0.973259
H -10.082695 3.742082 -0.865583
71191231236111012 0002.inp
C 0.00887905 -0.05803389 -0.23267199
C 1.42473661 0.04614766 0.18978333
C 1.95773254 1.46848457 0.29049326
C 3.31242357 1.61877693 0.94430632
H 3.19235041 1.51818009 2.0280678
H 3.72709321 2.60173218 0.72515999
H 4.00037753 0.83408933 0.6191499
O 1.33892925 2.41828225 -0.12044476
H -0.48327746 -1.02174903 -0.2506206
H -0.5221026 0.82389879 -0.56514072
H 2.09337699 -0.47987005 -0.51414637
H 1.60560013 -0.46032871 1.14944097
28076056008000000 0001.inp
C -0.38698215 -1.97245734 -1.08810989

C	-0.59261351	-0.93196532	-1.88361811
H	-0.63983915	-1.94101346	-0.03325662
H	0.04237502	-2.89707234	-1.46011172
H	-1.02197069	-0.00735032	-1.51161629
H	-0.33975652	-0.96340921	-2.93847137

S4.1.4 R4

712193323383422180162_r12_insertion_R_10_2_4.inp

C	-0.5119887	-1.07096222	-0.22851581
C	-1.15230548	0.24014098	0.14687015
C	1.14561189	0.53445195	0.07047974
C	-0.18834214	1.13769564	0.38696374
H	1.70424102	1.1015731	-0.68330043
H	-0.32154315	2.15108181	0.740846
O	0.85542437	-0.75594917	-0.44582192
H	-0.92114057	-1.51537665	-1.13937194
H	-0.61582436	-1.80112073	0.58810054
H	-1.72581372	0.82339926	-1.69360925
H	-2.20060168	0.34357987	0.39259615
H	1.77541968	0.45852936	0.96969483

711952311741200570042.inp

C	1.55728823	-0.06255581	0.00253493
C	0.06756163	0.01911483	-0.032036
C	-0.71685374	-0.60085391	-0.98887028
C	-2.09542935	-0.54491908	-1.036592
H	-2.65735242	0.01267636	-0.29486551
H	-2.65332041	-1.05440938	-1.81203476
O	2.17140411	1.22053388	-0.01540254
H	1.92257971	-0.68509871	-0.82572913
H	1.89706556	-0.52375861	0.93482417
H	1.8398352	1.69714178	-0.77854057
H	-0.40626185	0.61560838	0.74465259
H	-0.2055257	-1.17925603	-1.75774991

711952281671190640082_intra_H_migration_2_12.inp

C	0.06136476	-0.07302422	-0.03610145
C	-0.53548321	1.14115812	-0.71350911
C	1.88383468	1.20180291	-0.56201218
C	3.09126387	1.73111173	-0.62616859
H	3.21505938	2.75618352	-0.94910019
H	3.97439206	1.15547572	-0.36241464
O	1.4937492	-0.0333689	-0.25417895
H	-0.28563035	-1.02347028	-0.44223571
H	-0.11380525	-0.05904882	1.04283352
H	-1.21308834	1.7520011	-0.12284264
H	-0.86556959	1.00292762	-1.74118872
H	0.63973175	1.74777049	-0.79679336

712193434003141140082.inp

C	0.99389566	-0.96169696	-0.46165607
C	0.81808883	0.53644347	-0.72892839
C	-0.58766067	0.32853822	-0.0865889
C	-0.97912036	1.17444337	1.04935361
H	-0.50515012	1.0235118	2.01217571
H	-1.67429131	1.99493793	0.92053172
O	-0.22568417	-1.0182905	0.29473693
H	1.85736462	-1.25641148	0.14010631
H	0.94121678	-1.59335191	-1.35525519
H	0.8232704	0.86104305	-1.76882064
H	1.47163275	1.17558722	-0.13533775
H	-1.37832805	0.30948405	-0.84801955

711912461931090420002_r12_insertion_R_8_1_7.inp

C	0.00143367	0.01886778	0.00139487
C	1.52148993	-0.00938296	0.00567396
C	2.13898449	1.35605774	0.03513653
C	2.96592183	1.82725823	-0.89056478
H	3.26001047	1.22672925	-1.74641088
H	3.38000608	2.82672245	-0.82241598
O	-0.68263071	-0.88190253	-0.44526822

H -0.16734457 1.29797336 -1.29488938
H -0.46249575 0.81255296 0.62736099
H 1.86439412 -0.597241 -0.84834258
H 1.79785675 -0.56162665 0.91503948
H 1.86940687 1.98996072 0.87849891
711912461931090420002_r12_insertion_R_2_1_7.inp
C -0.0522328 0.11326009 0.12810203
C 1.41846122 0.27779896 0.39014033
C 2.20901 1.25058935 -0.36523514
C 3.42483451 1.66958255 -0.00541887
H 3.90294893 1.31151288 0.9013096
H 3.97172487 2.38919102 -0.60295614
O 0.18791098 -1.14087524 -0.51440336
H -0.47991526 0.85418018 -0.55455775
H -0.67685243 0.01199252 1.0189187
H 1.45165894 -0.87058061 -0.23250957
H 1.75137804 0.03851372 1.39882844
H 1.76166985 1.62567511 -1.28313258
701782141760940380001.inp
C -0.19537633 2.91684753 -0.70523158
C 0.63860498 2.33038204 0.14499501
C 1.2221221 -0.07716374 0.24113892
C 1.65888226 1.29302093 -0.2365512
H 1.74945013 1.24787934 -1.32818902
H 2.63892529 1.51310776 0.19280321
O 1.84380978 -0.76498896 1.00355474
H -0.17118834 2.69723983 -1.76885256
H -0.92510176 3.6444655 -0.36858769
H 0.58615603 2.57767418 1.2028992
H 0.24495754 -0.41690048 -0.16424278
711912461931090420002_intra_H_migration_7_12.inp
C 0.03703523 0.02059476 0.03831576
C 1.57167128 0.05386307 -0.03246501
C 1.79501792 1.45223265 -0.52615386
C 2.69192404 2.10371936 -1.22871229
H 3.58325524 1.59899407 -1.59966185
H 2.58313505 3.15686216 -1.46381403
O -0.34857393 1.34473534 0.33879323
H -0.33733571 -0.63624547 0.82954695
H -0.38399523 -0.30235204 -0.92226903
H 2.01586324 -0.71263896 -0.67341722
H 1.99708296 -0.04154122 0.9715152
H 0.66799173 1.9204765 -0.10831099
712193283463422100202.inp
C -0.55101695 -1.05940574 -0.16594004
C -1.24816739 0.26895542 0.1152385
C 1.10619501 0.46587434 0.03098678
C -0.0885607 1.1333043 0.6458172
H 1.97417345 0.95683991 -0.38769947
H -0.18150962 2.18489544 0.35897894
O 0.76299652 -0.69850844 -0.594316
H -1.00811259 -1.6499512 -0.95968996
H -0.4754727 -1.67271723 0.74045205
H -1.63949939 0.68806243 -0.81512227
H -2.07280737 0.16659784 0.82188981
H -0.04984527 1.10336992 1.74259847
711912291971130510002_r12_insertion_R_3_4_5.inp
C -0.032122 -0.093226 0.25748
C 1.425199 0.074912 -0.167067
C 1.008867 2.698989 -1.559333
C 1.952438 1.458704 0.028925
H 1.611134 2.028692 0.887988
H 2.969141 1.67349 -0.28788
O 0.675405 2.082875 -2.459417
H -0.690418 0.544667 -0.337669
H -0.362175 -1.126593 0.12941
H 2.055501 -0.619371 0.409595
H 1.549775 -0.22223 -1.21484
H -0.165444 0.174828 1.309614
712193434003141140082_r13_insertion_ROR_3_2_1_7.inp

C	1.74369288	1.46066226	0.16829037
C	1.77901151	-0.01263242	-0.13198073
C	-0.19492898	0.52520958	-0.13894861
C	-0.95652684	0.05973494	-1.16846665
H	-1.56467242	-0.82608962	-1.03715003
H	-0.90802168	0.52761653	-2.14414836
O	0.40698654	1.78259304	-0.22489101
H	2.4416166	2.0739169	-0.40792009
H	1.88833371	1.66790815	1.23583498
H	2.03828013	-0.72351681	0.64552098
H	2.02009589	-0.3167072	-1.14478551
H	-0.40247339	0.18397527	0.8774975

711912421971130380002_intra_H_migration_1_10.inp

C	0.6535052	3.38939813	-0.89659244
C	1.00881045	2.58021032	0.29901342
C	1.0964173	0.18940423	-0.26302199
C	1.91354932	1.38857357	0.16763732
H	2.66867946	1.57985522	-0.60669829
H	2.42936495	1.14713958	1.09862347
O	1.0809071	-0.86807028	0.3090468
H	1.13814927	3.17754114	-1.84137883
H	-0.24100933	3.99704613	-0.88838194
H	1.51213186	3.73555304	0.02581569
H	0.34045236	2.63616761	1.15084271
H	0.45640768	0.36100873	-1.15489533

711912421971130380002_r12_insertion_R_3_4_2.inp

C	2.02150121	-0.44285975	-0.86153073
C	0.86914433	0.00135208	-0.28633982
C	-0.22824032	2.51470783	0.36893909
C	0.99695733	2.19623573	-0.32823499
H	1.93813806	2.33404571	0.19149971
H	1.00185717	2.30856103	-1.40599577
O	-1.3167957	2.6063996	-0.17083409
H	2.11164382	-0.5412119	-1.9375329
H	2.90681286	-0.64589715	-0.26941976
H	-0.04985113	0.04792092	-0.86016109
H	0.75272729	-0.0506645	0.79116871
H	-0.14148054	2.63825297	1.46746622

711912421971130380002_r12_insertion_R_5_4_3.inp

C	3.17572911	2.62569351	-0.68004348
C	2.4610763	1.63867952	-1.58455805
C	1.45003286	0.11057766	0.23696173
C	1.4083886	1.36312171	-0.51858572
H	2.10250146	2.24276705	0.21951957
H	0.43605844	1.83894025	-0.63037482
O	2.34355744	-0.70741275	0.15530993
H	3.08341023	3.68993527	-0.87750973
H	4.08347399	2.29412015	-0.18428368
H	2.05685185	2.07263769	-2.4978513
H	3.04757533	0.7515268	-1.81971813
H	0.61948337	-0.03217987	0.95597569

711912461931090420002.inp

C	0.12074545	0.03028596	-0.223855
C	1.57809024	-0.06922604	0.23901737
C	3.22749004	1.69589881	-0.43562828
C	2.22637683	1.27992639	0.33101263
H	3.64918749	2.68913254	-0.32930079
H	1.8186012	1.95937882	1.07888942
O	-0.56930299	-1.13794877	-0.24836979
H	0.04524224	0.51316518	-1.21639126
H	-0.45750145	0.71314067	0.43221343
H	1.58993936	-0.56293977	1.21661669
H	2.12718489	-0.70924165	-0.457522
H	3.66608303	1.05140906	-1.19234351

711912311781240570002.inp

C	0.00348	-0.002834	-0.0198
C	1.537823	0.006236	0.053287
C	2.952521	1.911695	1.033319
C	2.102873	1.379139	0.021166
H	3.188442	1.237349	1.880223

H 1.858052 2.036915 -0.809692
O 3.416259 3.048587 1.0036
H -0.348335 0.493374 -0.927936
H -0.372889 -1.028046 -0.026491
H 1.875517 -0.5169 0.953666
H 1.932044 -0.556533 -0.803958
H -0.431464 0.515327 0.837926
71191229197113051 0002_r12_insertion_R_2_4_5.inp
C 0.065915 -0.017573 0.293918
C 1.435468 0.027801 -0.311534
C 3.130313 2.571369 -0.786775
C 1.935753 2.199862 -0.294726
H 1.081627 2.49595 -0.893677
H 1.827825 2.311263 0.778524
O 4.19356 2.597869 -1.258364
H -0.654566 0.560392 -0.294206
H -0.323197 -1.043118 0.353285
H 2.254386 -0.369661 0.281398
H 1.516762 -0.186827 -1.373379
H 0.062109 0.381541 1.313571
711952271911110530042.inp
C -0.68110323 2.32751288 0.88243279
C -0.43013251 1.47734812 -0.10830374
C 1.54188972 -0.0038597 -0.37809323
C 0.11956127 0.08822797 0.08080732
H 0.0336095 -0.19083574 1.14442532
H -0.47455781 -0.63203341 -0.48958131
O 2.44716491 0.87207545 0.13751131
H -1.04938361 3.32942854 0.69165275
H -0.54406251 2.04009235 1.92166326
H 1.98768756 1.68003404 0.39072809
H -0.57578714 1.7964017 -1.13843963
H 1.98882909 -0.95639929 -0.63363319
300540360000000000001.inp
C -1.99410726 -0.13543991 0.65996506
O -2.82582505 -0.97340653 0.86094188
H -1.98470247 0.46977248 -0.26751829
H -1.19010821 0.07885795 1.39089535
711952441951090360022.inp
C -0.00190499 0.04962251 0.00692425
C 1.52143801 0.02199161 -0.03116462
C 2.17353323 1.38090151 0.03608345
C 1.5683048 2.53818254 0.09693481
H 1.83421414 3.58330677 0.15690417
H -0.29907488 1.59174109 -1.1265137
O -0.57586798 0.67156241 -1.12406668
H -0.33792926 0.53561519 0.93345881
H -0.38157704 -0.9740104 0.0098217
H 1.85329818 -0.48070041 -0.94675482
H 1.89061463 -0.5816192 0.80676426
H 3.2697859 1.37901447 0.0231312
711912291971130510002_intra_H_migration_3_10.inp
C 2.66514 2.098501 0.749337
C 1.678206 1.363855 -0.106333
C 0.082125 0.111715 0.347619
C 1.528327 -0.148105 0.015077
H 1.696439 -0.739972 -0.884602
H 2.091935 -0.552703 0.857455
O -0.936164 -0.220088 -0.179796
H 3.689149 1.882609 0.416628
H 2.515418 3.178723 0.700269
H 0.369244 1.368953 0.635913
H 1.460815 1.774208 -1.088893
H 2.590742 1.786536 1.795094
711912421971130380002_beta_delta_1_2_4_3_7.inp
C 1.51552256 0.09763965 -0.13508376
C 1.32669965 1.40152433 0.28683673
C 3.14380531 2.59902695 1.32335689
C 1.92961526 1.81037305 1.65754938
H 1.17445702 2.42991637 2.1584661

H	2.14398908	0.96801238	2.32362875
O	3.1315287	3.09063494	0.15325493
H	0.91557756	-0.33893341	-0.92533677
H	2.32478484	-0.50597096	0.26433188
H	2.24753342	2.33207183	-0.28686627
H	0.38462627	1.87706671	0.01015177
H	4.02750991	2.69688618	1.94846872

71191246193109042002_intra_H_migration_7_5.inp

C	-0.43930648	1.39392273	0.05953212
C	-0.16134473	-0.10269646	-0.13243114
C	1.29361361	-0.37253349	0.16119355
C	2.18264974	0.59128148	0.05703254
H	1.54378699	1.68554354	-0.28962054
H	3.2583461	0.60762694	0.17781804
O	0.50655202	2.18727751	-0.59465638
H	-0.46836038	1.62174159	1.1351954
H	-1.41396413	1.64764334	-0.37365604
H	-0.37930813	-0.40389187	-1.16353386
H	-0.81238433	-0.68925649	0.52233794
H	1.58863491	-1.38451813	0.4390446

71219332383422180162_r12_insertion_R_7_1_2.inp

C	-0.28482256	-1.20588306	-0.51660952
C	-1.43293388	0.57107024	0.11751588
C	0.98781999	0.55723838	0.32157752
C	-0.2734126	1.27587813	-0.06225717
H	1.77781679	0.75635461	-0.41318806
H	-0.21674178	2.08405124	-0.78310503
O	0.69728565	-0.85317586	0.35357952
H	-0.11889507	-1.00111245	-1.57467298
H	-0.74447254	-2.15397864	-0.25675558
H	-2.34427033	0.88167376	-0.38415509
H	-1.57704122	-0.06196138	0.98711151
H	1.3728075	0.79689009	1.3158918

43091086038000000002.inp

C	0.83764064	1.53056639	1.15897594
C	0.37878597	0.64917672	2.17945111
H	-0.62048448	0.23456045	2.11220591
H	1.01246709	0.38827819	3.01921986
O	0.14972713	1.84937014	0.19354591
H	1.86202065	1.93099211	1.26521627

711952281671190640082_Intra_R_Add_Endocyclic_F_2_4.inp

C	0.18268865	-0.16025547	-0.45797263
C	-0.73337213	0.96023669	-0.08646256
C	1.70614621	1.2162023	0.60730775
C	1.08488833	2.23827867	-0.05411862
H	0.91680507	2.20550094	-1.12411308
H	1.13667424	3.23452171	0.37244495
O	1.42581618	-0.05913865	0.28873386
H	0.41125624	-0.14359648	-1.52915784
H	-0.20411937	-1.14943821	-0.20302495
H	-1.37140766	1.37862387	-0.85663949
H	-1.14425745	0.96335721	0.91801563
H	2.24999213	1.32248725	1.53891054

71219332383422180162.inp

C	-0.52254591	-1.01494107	-0.33153112
C	-1.27759143	0.3160868	-0.20611756
C	1.12150451	0.52590426	0.07097239
C	-0.18567633	1.21837434	0.25679804
H	1.84950022	1.09677146	-0.51673904
H	-0.3055869	2.22351424	0.63529443
O	0.81617737	-0.66430537	-0.64053107
H	-0.88899603	-1.66330526	-1.12781381
H	-0.56081965	-1.5652558	0.62018118
H	-1.67750378	0.63234222	-1.18082676
H	-2.1230338	0.25914124	0.48694996
H	1.59771159	0.28271787	1.03829635

711912421971130380002_Intra_R_Add_ExoTetCyclic_F_1_3_12.inp

C	-0.04484258	1.23391502	-0.76021611
C	-0.03447613	-0.14673185	-0.14102029
C	1.84632681	1.07403025	-0.52107202

C 1.37744308 -0.01490679 0.45776086
H 1.35806852 0.40353335 1.46562873
H 1.98657923 -0.92206313 0.44582363
O 2.33889677 2.16701664 -0.17398804
H -0.31292464 2.06104501 -0.10990673
H -0.28610385 1.38697943 -1.80750767
H -0.83280055 -0.32546248 0.58174682
H -0.07659429 -0.92366778 -0.90646663
H 2.06539666 0.69703941 -1.54096256
711912291971130510002.inp
C -0.006871 -0.014935 -0.013199
C 1.518664 0.004485 0.013357
C 1.737276 2.220477 1.238938
C 2.087656 1.420973 -0.003228
H 3.183698 1.399564 -0.01888
H 1.753628 1.987424 -0.881532
O 1.601707 3.384316 1.34648
H -0.391082 0.487788 -0.906017
H -0.388275 -1.038403 -0.013864
H 1.883464 -0.515554 0.904358
H 1.913452 -0.535987 -0.852017
H -0.421006 0.495024 0.862067
711912291971130510002_intra_R_migration_3_2.inp
C 0.315845 -0.152429 0.394298
C 1.644031 0.068966 -0.292362
C 2.601953 1.37701 0.657082
C 2.102917 2.038857 -0.445596
H 2.765003 2.497186 -1.187147
H 1.05143 2.284052 -0.492763
O 3.655461 1.136225 1.185991
H -0.382812 0.664602 0.1957
H -0.147343 -1.070606 0.016971
H 2.41841 -0.641099 -0.005875
H 1.558993 0.037291 -1.380585
H 0.437358 -0.241883 1.474317
280280000000000000001.inp
C 2.063871 1.411659 0.809157
O 2.159589 0.478026 1.430526
711912311911110570002.inp
C 3.888345 2.389795 -1.183835
C 2.898 1.283736 -1.118411
C 0.926047 0.086224 -0.074016
C 1.877019 1.256237 -0.042692
H 2.335732 1.260643 0.962162
H 1.253644 2.167975 -0.041817
O 0.943604 -0.79744 -0.887345
H 4.502456 2.439585 -0.272532
H 4.563835 2.279077 -2.033344
H 0.168864 0.085684 0.738756
H 2.90491 0.478273 -1.841701
H 3.397353 3.36998 -1.271247
711912291971130510002_intra_H_migration_3_8.inp
C 1.276943 1.792398 0.207643
C 1.505306 0.596742 -0.693583
C -0.81357 1.165957 -1.044421
C 0.125154 -0.020405 -1.002075
H 0.071778 -0.621624 -1.911467
H -0.214537 -0.645116 -0.166098
O -1.900801 1.283492 -1.49895
H -0.05694 2.014734 -0.356447
H 1.086652 1.5722 1.258006
H 1.973508 0.918702 -1.627911
H 2.166663 -0.149208 -0.243175
H 1.900168 2.669832 0.05781
711912291971130510002_intra_H_migration_suprafacial_7_5.inp
C -0.014827 -0.1025 -0.237591
C 1.451336 0.087709 0.147967
C 2.660768 2.147366 1.047172
C 1.916926 1.513004 0.048014
H 1.427295 1.998701 1.353637

H 1.766712 2.040403 -0.901004
O 3.318112 3.144407 1.192025
H -0.210646 0.276386 -1.244725
H -0.287849 -1.159727 -0.21828
H 1.640614 -0.284098 1.159614
H 2.086705 -0.505006 -0.520725
H -0.671786 0.431008 0.454912
711952301691190620062.inp
C 3.0047123 1.45502468 0.20188278
C 3.33664994 2.90801 0.45289119
C 1.1302007 0.05643576 0.04518754
C 1.83656771 -1.02756403 -0.19378918
H 1.64108205 -2.07048039 -0.37596566
H 2.88038703 3.54560452 -0.30718948
O 1.58925639 1.30638583 0.25134034
H 3.45712106 0.8022355 0.95898587
H 3.36253783 1.12456171 -0.78135789
H 2.9752032 3.22321062 1.43391834
H 4.41938252 3.05213493 0.4206032
H 0.04150957 0.02575486 0.09885394
290890730120000000002.inp
C -2.707677 -1.573127 -0.19947
C -1.383466 -1.907426 0.384691
H -2.617768 -1.211002 -1.227576
H -3.36975 -2.452736 -0.233602
H -1.227857 -1.888187 1.456637
H -0.586847 -2.302987 -0.233833
H -3.230086 -0.812923 0.388259
712193283463422100202_h2_elim_6_12.inp
C -0.66530842 -0.97713177 -0.11024587
C -1.28101716 0.36557852 -0.53992305
C 0.94599602 0.56344416 0.04803301
C -0.14395564 1.30405741 -0.23462278
H -0.18637145 2.38191312 -0.23831746
H 0.76751881 0.79224011 2.01220338
O 0.75873768 -0.77212454 -0.07659876
H -0.86673723 -1.80620885 -0.78679814
H -0.98060579 -1.24526316 0.90406657
H -1.53587094 0.38753378 -1.60636017
H -2.1912793 0.58897099 0.02160239
H 1.97327343 0.87456235 0.1703619
711952281671190640082_R_Addition_MultipleBond_2_1_7.inp
C 0.99780258 0.00046676 -0.02922107
C 1.64813893 -0.71233803 0.94976007
C 2.63716375 2.16989211 -0.11295738
C 3.57893931 2.76944058 0.64273225
H 4.47727757 3.16782388 0.18697662
H 3.41974771 2.91425404 1.70488258
O 1.46659084 1.8157196 0.35940813
H -0.06155922 0.1995993 0.06546586
H 1.38376098 -0.00228269 -1.04320161
H 1.19060237 -0.873229 1.91825078
H 2.67633896 -1.03018037 0.82184119
H 2.81449249 2.00784683 -1.18256725
712193323383422180162_r13_insertion_ROR_2_4_3_7.inp
C -0.43920247 1.42067989 0.19903345
C 0.13466223 -0.01022124 0.24545213
C 2.26535332 1.13515163 0.14669517
C 1.44608135 0.21438371 -0.44887335
H 2.34830344 1.19856431 1.2236667
H 1.54069951 -0.00732652 -1.5074581
O 0.62513753 2.3222082 0.38571774
H -1.17527431 1.59671247 0.99368955
H -0.93148175 1.57975325 -0.77067422
H 0.29143685 -0.3302029 1.28024284
H -0.51212291 -0.73432995 -0.25150123
H 3.04624833 1.62736404 -0.42298531
711912421971130380002.inp
C 4.17031 2.182549 -0.304289
C 3.320868 1.076908 0.210774

C 0.966596 0.104569 0.306905
C 1.865183 1.185511 -0.229481
H 1.428244 2.146467 0.075553
H 1.77746 1.167626 -1.323898
O 1.319344 -0.785905 1.033583
H -0.093662 0.174009 -0.018663
H 3.744512 3.153054 -0.535279
H 3.726089 0.106988 -0.097652
H 3.351822 1.050153 1.311491
H 5.24893 2.085937 -0.319821
712193283463422100202_Korcek_step2_4_11.inp
C 1.86174851 -0.15897985 -0.02984299
C 2.20721967 1.2858134 -0.09777404
C -0.16958865 1.33811112 -0.10258533
C 0.97321257 2.10501813 0.44975961
H 0.98896208 2.05195872 1.54340571
H 0.99784951 3.15372252 0.14801651
O -0.0696621 0.08990834 0.02276783
H 1.9945321 -0.67891083 0.91175839
H 1.95096393 -0.77450541 -0.91602635
H 2.41430299 1.5944216 -1.12485073
H 3.08455477 1.52248846 0.508381
H -0.86946049 1.7669161 -0.82905249
712193323383422180162_R_Addition_MultipleBond_4_3_5.inp
C -0.36793186 1.4427706 -0.37260048
C -0.0113997 -0.03127427 -0.11492348
C 1.86227633 1.29831626 -0.37183585
C 1.48608767 0.06196403 0.01074504
H 2.07289363 1.96377736 1.48678804
H 2.14890174 -0.71924646 0.34835264
O 0.84618501 2.07718125 -0.81369883
H -0.67707597 1.93830834 0.55422766
H -1.1237817 1.59952373 -1.14057265
H -0.49994824 -0.41208239 0.78513214
H -0.29800174 -0.68357774 -0.94863432
H 2.85335818 1.68741954 -0.55460583
711952281671190640082_intra_H_migration_2_6.inp
C -0.62065535 1.2387357 0.10734469
C 0.38015445 2.34053753 -0.12211172
C 1.06485833 -0.37287701 -0.21250614
C 2.00627341 0.43438667 0.24916652
H 3.04406961 0.21112242 0.44989651
H 1.45377037 1.70614412 0.20834909
O -0.21434937 -0.00215026 -0.47438988
H -1.59543433 1.45237802 -0.33597032
H -0.75062016 1.07808827 1.18517042
H 0.51685622 2.63250731 -1.16280524
H 0.27121059 3.18912917 0.55171671
H 1.21898036 -1.42379744 -0.44576707
711912421971130380002_r12_insertion_R_10_2_4.inp
C -0.08942349 2.96721979 -0.68832818
C 0.81902723 2.47242583 0.16839062
C 1.12512126 0.01443107 0.09680661
C 1.77327276 1.35763849 -0.18141115
H 2.00347687 1.39102092 -1.25267737
H 2.70599227 1.42045866 0.37988664
O 1.58916134 -0.83021052 0.81114331
H -0.07970599 2.69927423 -1.74054586
H -0.8301724 3.68981988 -0.36713723
H 2.19434951 3.9103704 0.16322983
H 0.73373592 2.71285919 1.22494684
H 0.15235206 -0.14360445 -0.41692496
411131060600080000002.inp
C 1.33195172 -0.63226124 -0.80512502
C 1.35923396 0.74889068 -0.81931964
C 1.7470346 1.53746635 0.2466606
H 2.06175003 1.09153477 1.18451091
H 1.74860434 2.61812367 0.17848378
H 1.01840301 -1.19982513 -1.67232044
H 1.62572086 -1.18653772 0.08027495

H 1.05375648 1.25111564 -1.73473714
7121934340031411400822_r12_insertion_R_4_3_7.inp
C -6.61719762 2.30841785 0.21826315
C -5.56923142 1.38433075 -0.38871159
C -4.65977675 2.49898333 -0.88741304
C -3.39458292 2.71103386 -0.40512063
H -2.76166349 3.48823863 -0.81639869
H -3.03489777 2.17390781 0.46583359
O -5.83557938 3.49135448 0.24596661
H -6.9574964 2.02450224 1.22012416
H -7.49151035 2.43385202 -0.43508583
H -5.92440486 0.72657311 -1.18425564
H -5.05814159 0.78527373 0.367171
H -4.93494993 2.96903433 -1.82632506
711952151711150730122.inp
C 1.57275628 0.01172109 -0.09268991
C 0.06908619 0.05053725 0.0423973
C 3.34763966 1.51992951 -0.30515522
C 4.19835197 2.30066374 0.32959135
H 5.22534087 2.37735388 -0.0014777
H 3.88461967 2.87663831 1.19886583
O 2.04609279 1.36897334 -0.17513946
H 1.88308777 -0.51956312 -0.9968652
H 2.04432443 -0.46610954 0.77215169
H -0.22557895 0.59062928 0.94428144
H -0.32038114 -0.96832908 0.10602691
H -0.38331095 0.541165 -0.82157986
711912461931090420002_R_Addition_MultipleBond_7_1_2.inp
C 1.10849685 0.03968839 0.00804452
C 1.25975853 2.02866681 0.00595838
C 0.46560884 2.48633603 1.11834537
C 0.9605461 2.69053246 2.35167116
H 2.01535835 2.54466936 2.56499062
H 0.32935619 3.01908214 3.16879103
O 1.73780172 -0.30711947 -1.00918259
H 0.00344012 0.02738211 0.00294139
H 1.56085986 -0.07501414 1.00863974
H 0.88070915 2.19963805 -0.99480042
H 2.33905616 2.09681204 0.09683417
H -0.59786689 2.63071906 0.94530007
43139151085012000002.inp
C -2.101638 -0.908264 -0.08205
C -0.746699 -0.959987 -0.787292
C -0.348725 0.331829 -1.407454
H 0.463447 0.378049 -2.123411
H -0.782544 1.266514 -1.068766
H -2.898652 -0.655651 -0.786558
H -2.347022 -1.869344 0.376489
H 0.022722 -1.273829 -0.06215
H -0.751166 -1.750041 -1.549306
H -2.100339 -0.150609 0.707115
420780560340000000001.inp
C 1.535148 1.571713 -0.440542
C 0.380684 1.459196 0.165024
H -0.08497 2.337495 0.58671
H -0.089649 0.48964 0.234429
O 2.555723 1.671172 -0.975861
712153574263300920002.inp
C -0.98276904 0.36342717 -0.52133099
C -1.15464552 -1.04096351 0.08858298
C 0.59646069 0.17113477 -0.32865206
C 0.26588906 -0.94752715 0.68736541
H 0.25961037 -0.53459019 1.69813898
H 0.8887273 -1.84275305 0.65682168
O 1.17821263 1.306129 0.03838231
H -1.33299983 1.15369897 0.14280227
H -1.30453958 0.55617368 -1.54347085
H -1.98374914 -1.16638433 0.78491133
H -1.22239359 -1.80827948 -0.68507115
H 0.99131464 -0.21672089 -1.28258091

711912291971130510002_intra_R_migration_3_1.inp

C	-0.088863	1.564411	0.871386
C	1.496453	1.345435	-0.326814
C	0.205858	-0.206972	0.257661
C	1.670066	-0.117661	-0.06298
H	1.970874	-0.74175	-0.904365
H	2.287004	-0.31744	0.813454
O	-0.684656	-0.796529	-0.261184
H	-1.153303	1.298061	0.940386
H	0.335517	1.590275	1.87641
H	1.15265	1.619911	-1.318436
H	2.158095	2.062029	0.146747
H	-0.116452	2.595538	0.483795

711912312361110120002.inp

C	0.014854	0.010269	0.09091
C	3.695687	1.221237	-0.217968
C	1.492442	-0.02305	-0.226681
C	2.219375	1.31151	-0.138364
H	1.887889	1.826985	0.775159
H	1.810875	1.934387	-0.953538
O	2.057443	-1.04511	-0.526814
H	-0.464466	-0.902276	-0.26079
H	-0.109532	0.080457	1.176578
H	4.302427	2.106395	-0.077778
H	4.167251	0.278154	-0.459816
H	-0.466872	0.887316	-0.348937

280760560080000000001.inp

C	-1.52890991	-2.43412635	-1.69315954
C	-0.72977342	-1.43764832	-1.33797048
H	-2.58733832	-2.27772133	-1.87456401
H	-1.15644917	-3.44589224	-1.81663801
H	0.328655	-1.59405333	-1.15656601
H	-1.10223418	-0.42588243	-1.21449195

711952281811130560082.inp

C	1.4547866	2.38188986	-0.60496639
C	-0.84160346	-0.60013839	-0.85728557
C	1.40474378	0.10908496	-0.0339533
C	-0.08401172	-0.03596243	0.0740634
H	1.90439882	-0.52764947	0.69952
H	-0.52662912	0.33891187	0.99379728
O	1.84802164	1.42649772	0.26582527
H	1.88125964	3.35491919	-0.40762339
H	1.13698876	2.08111007	-1.59874289
H	-0.41664751	-0.9705461	-1.78620558
H	-1.91130173	-0.71624495	-0.72527973
H	1.74958898	-0.18073196	-1.03562737

702063043023081880101.inp

C	0.22549157	0.99702586	0.21233242
C	1.14363767	0.1182064	-0.58546487
C	-0.79629548	-1.06858659	-0.16881016
C	0.56434437	-1.05294828	-0.8015729
H	-1.59604828	-1.2215546	-0.90537651
H	0.97862157	-1.89126916	-1.34609441
O	-0.93817705	0.21118709	0.43344772
H	-0.04473969	1.91464699	-0.32663844
H	0.66236212	1.2898167	1.17610281
H	2.12564896	0.42783863	-0.91815455
H	-0.89071977	-1.84989104	0.5967499

711952331891050550042.inp

C	1.47863645	0.08871778	0.02551266
C	-0.04504248	-0.0854072	-0.00562523
C	-0.66513818	0.68137066	-1.09905441
C	-1.3546624	1.78085118	-1.27066213
H	-1.67075373	2.3868281	-0.41648026
H	-1.65728542	2.1292698	-2.25445749
O	1.8634222	1.42468284	0.25768206
H	1.91067372	-0.29119725	-0.90969938
H	1.89256921	-0.49480834	0.8495783
H	-0.46303564	0.25559853	0.95383137
H	-0.28293724	-1.14986765	-0.10720937

H	1.4766182	1.97219121	-0.43022953

711912312231240120002.inp

C	0.041507	-0.23629	0.486006
C	1.175125	0.207319	-0.42552
C	1.545549	1.673968	-0.266751
C	2.609846	2.188064	-1.089938
H	2.88806	3.230133	-0.991067
H	3.134588	1.559699	-1.801139
O	0.967534	2.406242	0.530111
H	-0.863883	0.34206	0.292591
H	-0.182446	-1.293997	0.330525
H	2.078746	-0.387541	-0.244699
H	0.919656	0.038707	-1.47879
H	0.303462	-0.087798	1.535268

711912421971130380002_intra_H_migration_3_10.inp

C	2.590769	1.956348	0.925929
C	1.841472	1.46505	-0.109048
C	0.056237	0.119961	0.106706
C	1.524723	0.004432	-0.318649
H	1.613424	-0.312015	-1.357227
H	2.107555	-0.652121	0.330224
O	-0.901413	0.040245	-0.683193
H	2.977291	1.299691	1.698045
H	2.761651	3.020245	1.036931
H	0.350617	1.482887	0.372562
H	1.593967	2.120175	-0.942063
H	-0.102329	-0.032449	1.196558

711952281671190640082.inp

C	2.96679812	1.51244137	0.1533099
C	3.25347225	2.95155315	0.32576429
C	1.12265572	0.04184201	0.10406032
C	1.83473513	-1.06681134	-0.09260706
H	1.3108299	-2.00966597	-0.17091893
H	2.91288835	-1.08316148	-0.18038793
O	1.56740597	1.30756937	0.22928787
H	3.46402785	0.90885961	0.93356947
H	3.3453569	1.14402611	-0.81355375
H	2.4883877	3.61377459	0.70765834
H	4.25641667	3.32166133	0.15870157
H	0.04157173	0.0200305	0.18580268

702063003103081800141.inp

C	-1.0979964	0.42957624	-0.18884896
C	-0.71454561	-0.8994685	0.48971749
C	1.11393625	0.13964649	-0.45101169
C	0.73772067	-1.01389951	0.08850185
H	1.37665865	-1.86454066	0.26811394
O	0.13753748	1.08044439	-0.5480522
H	-1.65957172	1.11005807	0.45071405
H	-1.66004882	0.25949392	-1.11242314
H	-0.82509423	-0.85755282	1.57924975
H	-1.33046522	-1.72649359	0.1287822
H	2.08300896	0.45406095	-0.8120133

S4.1.5 *anti*-ROO1

10327943356952442214 82_HO2_Elimination_from_PeroxyRadical_4_14.inp

C	1.70586844	-0.27896671	0.37419629
C	0.30708835	-0.25391866	0.43792728
C	0.29890568	-1.27625794	-0.72306732
H	0.04196247	0.99774577	-0.09927179
H	-0.32456184	-0.2597021	1.32053302
C	-0.08643871	-2.7094263	-0.45991222
H	0.36063963	-3.07306887	0.47038225
H	0.23309151	-3.35564169	-1.28404539
H	-1.1780359	-2.78268517	-0.3728647
O	1.86168717	-1.10522742	-0.72118255
H	-0.05411306	-0.90076049	-1.68564196
O	1.60495298	2.1483136	-0.56834282
H	2.55741172	0.11581722	0.90471041
O	0.24541748	2.11188218	-0.58728565

10328342251450741618 52.inp

C	-0.05202049	0.02103138	-0.05673375
C	-0.69938997	-0.14454969	1.28101659
C	-1.36309581	1.20237165	1.16446093
H	2.18083766	1.75074382	0.51529054
H	-0.51495154	-0.82140762	2.1033181
C	-2.8769002	1.30101115	1.11247314
H	-3.29403929	0.54945492	0.43543244
H	-3.17695692	2.29429341	0.76111408
H	-3.29963003	1.14710273	2.11344197
O	-0.73194923	1.32836425	-0.2291021
H	-0.93190811	1.9756243	1.81444841
O	1.3860296	0.08877332	-0.13721137
H	-0.26808381	-0.67614014	-0.87456681
O	1.89183881	0.95103638	1.01450814

1032553213112141490832_Intra_R_Add_ExoTetCyclic_F_10_12_14.inp

C	-0.157734	-0.143053	-0.231345
C	-0.700655	-1.321105	0.04428
C	0.341419	-2.335006	0.433946
H	2.369131	1.624443	0.221978
H	-1.761625	-1.521312	0.018248
C	0.332031	-2.665941	1.924444
H	0.499505	-1.755795	2.504408
H	1.120424	-3.384724	2.15637
H	-0.630224	-3.095462	2.217078
O	1.557429	-1.809876	-0.002645
H	0.19844	-3.25055	-0.16533
O	1.198183	-0.063579	-0.200508
H	-0.656046	0.784088	-0.47616
O	1.422547	1.629618	0.413809

701782031571050570001.inp

C	1.081275	0.019786	0.001007
C	1.725709	1.341336	0.000047
C	1.083814	2.51524	0.001063
H	2.811226	1.316662	-0.001632
C	-0.389854	2.773751	0.003334
H	-0.661374	3.368871	-0.873949
H	-0.658683	3.368814	0.881484
H	-0.997351	1.870007	0.004235
O	1.701537	-1.017209	-0.000006
H	1.699571	3.412862	0.000136
H	-0.025614	-0.000302	0.002744

170170000000000000002.inp

H	1.785729	2.696169	0.065504
O	1.857232	1.872641	-0.443569

330490170000000000002.inp

H	1.27429039	1.14997439	-0.30376885
O	2.81176897	0.75521913	-1.39945436
O	2.07153664	1.64692048	-0.62102779

10328342251450741618 52_beta_delta_2_1_12_14_4.inp

C	-0.01095753	-0.14283528	-0.26123802
C	-0.2224582	-0.97768305	0.83558534
C	-0.454723	0.23166112	1.72049853

H	1.96587594	1.76212553	0.40705982
H	-0.12106178	-2.03504497	1.01811316
C	-1.7834697	0.4397574	2.4077987
H	-2.61407674	0.22398768	1.72970483
H	-1.87187007	1.47320667	2.75931734
H	-1.85798086	-0.22513032	3.27768361
O	-0.34822009	1.07303892	0.40045123
H	0.40712291	0.51005002	2.33589055
O	1.94745679	-0.03643762	-0.33932338
H	-0.06442007	-0.227661	-1.33514349
O	2.36130892	0.90552052	0.70292036

1032794335695244221482_intra_R_migration_14_10.inp

C	1.61321193	1.17664353	-0.04720195
C	0.8509677	2.05587502	-0.9949752
C	-0.53757025	2.22645827	-0.32054273
H	0.76927431	1.57847706	-1.97779504
H	1.35423271	3.02373337	-1.07068111
C	-1.1990988	3.58173591	-0.52719641
H	-0.53070152	4.3924032	-0.21779389
H	-2.12032131	3.64847333	0.06141316
H	-1.46186395	3.71816584	-1.58355393
O	-0.1131219	2.04004578	1.07485728
H	-1.19798905	1.40290685	-0.60801291
O	1.55476681	-0.12273065	-0.09780267
H	2.35455107	1.53409174	0.65802484
O	0.64610037	-0.70634299	-1.05145544

1032553213112141490832_Intra_R_Add_ExoTetCyclic_F_10_2_5.inp

C	1.566591	2.363138	0.69515
C	1.897187	1.446648	-0.287042
C	1.280121	0.10838	-0.330146
H	2.436424	4.625267	-0.768493
H	2.667273	1.677389	-1.007765
C	2.062348	-1.036819	-0.929502
H	2.603954	-0.705414	-1.818416
H	1.379699	-1.838162	-1.222572
H	2.775746	-1.437986	-0.204206
O	0.526319	0.876282	-1.204301
H	0.746585	-0.183291	0.584089
O	1.937277	3.66328	0.741467
H	0.863487	2.164821	1.494312
O	2.905444	3.958368	-0.253336

1032794335695244221482.inp

C	1.50674256	-0.03998386	0.1128307
C	-0.02398686	-0.0219402	0.04198509
C	0.12056083	-0.5476193	-1.41480864
H	-0.46973446	0.96776237	0.15761742
H	-0.50401645	-0.72763089	0.72445297
C	-0.49412784	-1.87542581	-1.78565683
H	-0.28724901	-2.63175138	-1.02084802
H	-0.09104254	-2.23105955	-2.74004045
H	-1.58182327	-1.77165323	-1.89226699
O	1.62896683	-0.67056604	-1.18667015
H	-0.05215563	0.21528407	-2.17817761
O	2.00132324	1.36836274	0.08308063
H	2.06649732	-0.54902654	0.89514523
O	3.33671267	1.45429373	0.45071998

702063003512521190061.inp

C	-0.0559155	-1.26557492	-0.28264871
C	-1.16205146	-1.54508798	0.43069077
C	-1.19819851	-0.10333188	0.93395708
H	-1.77716057	-2.41336472	0.6014835
C	-2.31605066	0.8226915	0.5182163
H	-2.52882594	0.71297844	-0.549434
H	-2.04709631	1.86527382	0.72022903
H	-3.22761236	0.58732642	1.08264592
O	0.07500418	0.11851767	0.06186355
H	-0.89396716	0.03043485	1.97559447
H	0.6530533	-1.7440832	-0.9403409

1032834075464884142042_intra_H_migration_14_4.inp

C	1.478891	0.058886	-0.481812

C	2.08643	1.494335	-0.417333
C	2.574324	1.115641	0.948603
H	1.29692	2.248187	-0.454557
H	2.858507	1.72743	-1.16337
C	3.685621	1.508542	1.832892
H	4.658638	1.117811	1.479924
H	3.528719	1.11643	2.844258
H	3.768776	2.598903	1.891682
O	2.037064	-0.17534	0.955249
H	-1.422256	0.597712	0.415906
O	0.142757	-0.134453	-0.644248
H	1.9775	-0.673654	-1.123514
O	-0.644944	1.146782	0.163096

862383323953932491131.inp

C	0.50909	-1.111436	0.611623
C	-0.781549	-1.320067	0.41014
C	-1.427913	0.031823	0.277357
H	-1.276258	-2.26957	0.290118
C	-1.883496	0.365965	-1.137237
H	-1.052099	0.255958	-1.837003
H	-2.258231	1.390511	-1.188302
H	-2.687072	-0.312372	-1.439265
O	-0.380603	0.92507	0.712258
H	-2.246903	0.17926	0.990297
O	0.862799	0.190278	0.708259
H	1.34285	-1.793217	0.697533

103251332349222134 0512.inp

C	0.06542747	-0.00844753	0.27373833
C	1.38464262	0.06022022	-0.37814489
C	2.0265582	1.48499723	-0.20943438
H	1.24624075	-0.10385314	-1.45653252
H	2.05686901	-0.69745803	0.0316218
C	3.26843816	1.6833943	-1.08960936
H	4.03678262	0.93965354	-0.84913536
H	3.68380386	2.67961776	-0.91302012
H	3.0080723	1.59221624	-2.14929743
O	2.26178881	1.77845705	1.14322154
H	1.24037331	2.21042045	-0.52432697
O	-0.93935596	0.68037164	-0.1376794
H	-0.15849539	-0.60661961	1.15223934
O	-0.72947631	1.53293552	-1.30998542

1032553252922271680662_r12_insertion_R_2_1_12.inp

C	0.415985	0.046784	0.236094
C	0.05344	1.255982	1.017368
C	-0.026275	1.319111	2.346302
H	2.612611	1.299811	-1.477741
H	-0.178575	2.126043	0.413043
C	0.258148	0.243719	3.347803
H	-0.613008	0.087571	3.991034
H	1.08103	0.554752	3.998884
H	0.530399	-0.71099	2.899002
O	-0.007461	-0.04293	-0.972828
H	-0.327994	2.273229	2.773846
O	2.053834	0.141449	-0.161225
H	0.510555	-0.883598	0.811778
O	2.395347	1.405415	-0.542508

862624746286584661681.inp

C	0.01628126	-0.44975047	-0.78482291
C	-0.34093009	-0.73321384	0.61428341
C	-1.20437969	0.541852	0.6061042
H	-0.45815962	-1.64065646	1.18801566
C	-2.70098954	0.36072835	0.69778527
H	-3.03731959	-0.47137941	0.07037996
H	-3.21069211	1.26956908	0.36213057
H	-2.99729271	0.16220554	1.73590311
O	-0.77957768	0.75797857	-0.86597937
H	-0.81881997	1.35924694	1.21690894
O	1.06737031	-0.30643103	0.25372845
H	0.24470444	-1.00284526	-1.68079129

1032553213112141490832.inp

C	-0.063053	0.046817	0.257445
C	-0.76152	0.682211	-0.672706
C	-2.255766	0.499771	-0.803054
H	2.398767	1.546288	0.159739
H	-0.271832	1.350762	-1.369516
C	-3.01047	1.839922	-0.883507
H	-2.88659	2.384216	0.055082
H	-4.072674	1.664836	-1.059169
H	-2.606268	2.450955	-1.69366
O	-2.823564	-0.336337	0.105363
H	-2.462629	-0.025384	-1.764308
O	1.273167	0.111363	0.496135
H	-0.503799	-0.64475	0.967071
O	1.910782	0.971588	-0.439654

320320000000000000003.inp

O	0.00504762	2.38280528	0.66482711
O	0.24917138	2.44164672	-0.56634111

1032834075464884142042_intra_H_migration_3_11.inp

C	-0.02614099	-0.01226899	0.19089947
C	-0.89935485	1.25837719	0.2705114
C	-0.71400863	1.26130035	-1.26091005
H	-0.4997414	2.09324501	0.8450717
H	-1.93016917	1.03253138	0.56303781
C	-1.55822792	1.83245648	-2.34771043
H	-2.59065567	1.46134526	-2.27122805
H	-1.1663163	1.54934897	-3.33005241
H	-1.57885302	2.92539729	-2.28276752
O	-0.4688933	-0.17638113	-1.25188471
H	0.59390325	1.65983469	-1.21143946
O	1.38024319	0.24918089	0.20869419
H	-0.18187308	-0.908809	0.78354346
O	1.64772373	1.55092864	-0.5314891

1032553252922271680662.inp

C	0.100238	0.03543779	-0.19561959
C	-0.50771689	-0.82484389	-1.28319356
C	0.01956392	-1.96585844	-1.75840277
H	2.17305385	1.98941138	0.23472559
H	-1.41646881	-0.41645645	-1.71345329
C	1.26769002	-2.66062909	-1.30132937
H	1.8342543	-2.0629587	-0.57755761
H	1.91815056	-2.88671749	-2.15577019
H	1.02481364	-3.61676555	-0.81746279
O	0.8377457	-0.65677238	0.75170065
H	-0.51698957	-2.44949653	-2.57570341
O	0.88218847	1.05569657	-0.91703424
H	-0.68715281	0.56698278	0.36749414
O	1.20923498	2.14596979	0.09839943

1032794335695244221482_r12_insertion_R_2_1_12.inp

C	1.42577386	-1.19467144	0.33187886
C	0.30842755	-0.24765765	0.07541044
C	0.48913241	-0.63833547	-1.42759567
H	0.52576904	0.81463164	0.29020286
H	-0.66778044	-0.49296961	0.51169717
C	-0.59033401	-1.434416	-2.11551014
H	-0.94978565	-2.24524297	-1.47325147
H	-0.21717652	-1.86803179	-3.04926838
H	-1.43701868	-0.77893427	-2.35656931
O	1.66984669	-1.54764407	-0.99811029
H	0.9069538	0.15249798	-2.05259406
O	1.58438014	2.5668818	0.34640594
H	2.07481676	-1.50653054	1.13475091
O	1.2202734	3.75295645	0.01158303

1032834225145074161852_R_Addition_MultipleBond_2_1_10.inp

C	-0.100205	0.170732	-0.137462
C	-0.804642	-0.474784	-1.158749
C	-1.813536	-1.295125	-0.387328
H	2.519101	1.097274	0.988546
H	-0.268962	-0.880661	-2.01388
C	-3.112787	-0.647984	0.090759
H	-2.914614	0.339206	0.529396

H	-3.583667	-1.278647	0.856509
H	-3.813056	-0.523698	-0.747271
O	-0.797263	-1.399315	0.729867
H	-2.003413	-2.269479	-0.856521
O	1.25377	0.396088	-0.325466
H	-0.537471	0.786092	0.639618
O	1.667154	1.510303	0.69702

88192211178128064 0191.inp

C	-0.023473	0.058852	0.130914
C	-0.857187	1.102761	0.103777
C	-2.280347	0.844453	-0.100276
H	2.627489	1.316123	0.029812
H	-0.51828	2.122168	0.224751
O	-3.131052	1.699228	-0.156408
H	-2.554809	-0.228407	-0.207495
O	1.304592	0.051174	0.30152
H	-0.354199	-0.970267	0.01926
O	1.788107	1.373334	0.50001

1032834225145074161852_r12_insertion_R_2_3_10.inp

C	-0.07670457	0.04888925	-0.18261738
C	-0.70573002	-1.29801198	-0.14618969
C	-0.87964586	-1.4593112	1.26920371
H	1.83152934	1.5194721	1.10677577
H	-1.43466552	-1.62434725	-0.8796161
C	-2.08671092	-2.08612666	1.89461272
H	-2.34253306	-1.55447118	2.81796275
H	-1.87001644	-3.12770799	2.1726978
H	-2.95471935	-2.06895545	1.2302185
O	-0.78118559	0.43395659	1.05671759
H	0.01430716	-1.44643124	1.88762291
O	1.36034755	-0.05566233	0.02197027
H	-0.27265888	0.73008388	-1.00956837
O	1.88288037	1.37107034	0.13133174

862343734213371620571.inp

C	1.137139	-0.015661	-0.017555
C	1.809519	1.314023	0.02757
C	2.196794	1.953416	-1.244373
H	2.37968	1.531958	0.927199
C	3.37312	2.882905	-1.32556
H	3.548534	3.369364	-0.364385
H	3.192179	3.654874	-2.0773
H	4.271158	2.329454	-1.611124
O	1.062413	2.392218	-0.523705
H	1.946519	1.422834	-2.163429
O	1.422109	-0.928753	0.708619
H	0.349201	-0.103637	-0.79505

1032834075464884142042.inp

C	-0.25907154	0.28111383	-0.43356469
C	-0.85051965	-0.65626843	-1.51092508
C	-0.95628369	-1.6844467	-0.39725464
H	-0.16192918	-0.88939186	-2.3281701
H	-1.79914731	-0.29699903	-1.93202774
C	-1.9830671	-2.69405726	-0.05719827
H	-2.97152313	-2.24067035	0.14424453
H	-1.69083622	-3.25302545	0.83920616
H	-2.10422582	-3.40860018	-0.87955976
O	-0.49277313	-0.75612113	0.62335882
H	2.13574794	0.81042907	0.95237071
O	1.11691484	0.51536622	-0.69809036
H	-0.76117906	1.20735854	-0.16210312
O	1.62810801	1.44767204	0.39420167

862062612811410570001.inp

C	-0.00388417	-0.32919881	-0.43884081
C	1.16066103	0.14752355	0.4097576
C	1.69304485	1.5057466	-0.03778044
H	1.97910441	-0.58007213	0.27500751
H	0.87780742	0.13065953	1.46609278
C	1.97626025	2.53103908	1.02949306
H	1.05056858	2.78723562	1.56246247
H	2.3998693	3.43247205	0.58269351

H 2.6725232 2.12713711 1.77617489
O 1.87584268 1.73543306 -1.24637597
O -0.94922712 -0.97934334 0.02203201
H 0.03982016 -0.06659062 -1.50635432
1032553252922271680662_intra_R_migration_10_2.inp
C 0.776496 -0.4244 0.170803
C 0.351584 -0.373225 2.192965
C -0.438209 -1.270377 2.725603
H 0.329012 1.566041 -0.530026
H 0.928664 0.463522 2.559776
C -1.205779 -2.352645 2.026653
H -1.114812 -2.277127 0.943782
H -2.265436 -2.280395 2.285211
H -0.857889 -3.335998 2.356241
O -0.327898 -0.241019 -0.328483
H -0.545295 -1.224295 3.813511
O 1.752419 0.533435 0.121485
H 1.274785 -1.396165 0.277229
O 1.186887 1.804966 -0.128353
712193284022841360122.inp
C 0.52588348 -1.74665692 0.93911946
C -0.51254404 -0.65613462 0.82540246
C -0.30326909 -0.78009902 -0.7179857
H -0.23956084 0.32205771 1.2433446
H -1.52166522 -0.90990773 1.17137165
C -1.42136275 -1.35393586 -1.55548236
H -1.84595467 -2.2434295 -1.0778938
H -1.05355642 -1.63661946 -2.54789594
H -2.21859651 -0.60990827 -1.68308538
O 0.8001099 -1.81122347 -0.46495441
H 0.16149817 0.09285614 -1.18343613
H 1.315599 -1.966224 1.64604756
1032794335695244221482_intra_H_migration_14_4.inp
C 1.36622194 -0.08431548 -0.30828831
C 2.03844379 1.29072608 -0.47763478
C 2.97356045 0.96862473 0.68668294
H 0.90837617 1.88859556 0.08945547
H 2.33777521 1.75519731 -1.41446843
C 4.45637891 0.8330679 0.40134672
H 4.6338403 0.24639552 -0.50579315
H 4.95186804 0.33061252 1.23881708
H 4.91111051 1.82399376 0.2774904
O 2.28443222 -0.38707375 0.80801841
H 2.78566388 1.54047221 1.60076533
O -0.01949197 0.10618882 0.01330704
H 1.38058227 -0.867461 -1.0664124
O -0.09273559 1.49366096 0.63064831
1032553213112141490832_r12_insertion_R_3_6_7.inp
C -0.157757 0.371635 0.51711
C -0.841163 1.178148 -0.293242
C -2.295416 0.94956 -0.486791
H 2.599277 1.067981 -0.133985
H -0.35891 1.986617 -0.82614
C -3.037218 2.644184 0.490593
H -2.712221 2.426888 1.500504
H -4.095928 2.533977 0.28993
H -2.521571 3.449576 -0.021747
O -2.922028 0.047368 0.088649
H -2.697558 1.37411 -1.42666
O 1.155934 0.400414 0.815458
H -0.624695 -0.445158 1.057998
O 1.792407 1.497488 0.170164
150390060000000000002.inp
C -3.268987 2.423067 -0.731633
H -2.447993 2.689214 -0.080456
H -3.880599 1.560685 -0.505539
H -3.478559 3.019455 -1.608727
1032834225145074161852_Cyclic_Ether_Formation_2_12.inp
C -0.12808015 -0.02353279 -0.11009712
C 0.19139319 -1.4686018 0.02010051

C	-0.20664749	-1.44131816	1.47799
H	2.0855228	1.95121775	0.68002218
H	0.56528911	-2.21352149	-0.66604847
C	-1.35789765	-2.33716664	1.90385706
H	-2.17978034	-2.29329407	1.18311312
H	-1.734359	-2.00387245	2.87668256
H	-1.01996222	-3.37643222	1.99923368
O	-0.66644017	0.00420892	1.27311541
H	0.6240747	-1.47318446	2.18936175
O	1.27677081	0.13592964	-0.15934806
H	-0.7077286	0.5398981	-0.83076536
O	1.65475243	1.94962734	-0.21028105

S4.1.6 *syn*-ROO1

1032794335695244221482_HO2_Elimination_from_PeroxyRadical_4_14.inp

C	1.53448287	-0.39672058	0.17865353
C	0.14725332	-0.38729877	0.32856488
C	0.16099035	-1.38211427	-0.84006869
H	-0.06613366	0.8691787	-0.17394015
H	-0.42001085	-0.44333204	1.24937845
C	-0.53160718	-1.05841573	-2.13451324
H	-0.21913232	-1.74926135	-2.92073907
H	-0.31136474	-0.0366166	-2.44762628
H	-1.61194448	-1.15698441	-1.99813106
O	1.62735319	-1.16117426	-0.91224406
H	0.01141436	-2.41796869	-0.53129459
O	1.54795783	1.79357513	-0.63523784
H	2.41554094	-0.10699555	0.73267268
O	0.28260371	1.95613178	-0.64179167

1032834335505263841702_Cyclic_Ether_Formation_6_12.inp

C	1.476425	1.238954	-0.044645
C	1.638809	-0.258015	-0.329649
C	0.105523	-0.153732	-0.241786
H	2.174114	-0.85904	0.404031
H	1.978795	-0.447682	-1.346853
C	-0.259965	-0.084505	1.199837
H	1.3354	3.253569	2.12659
H	-1.112051	0.516461	1.490858
H	0.085183	-0.847253	1.88776
O	0.201843	1.224318	-0.696667
H	-0.54188	-0.764175	-0.876214
O	1.25597	1.397234	1.339533
H	2.159162	2.00169	-0.405328
O	2.097231	2.79984	1.74887

1032834225145074161852.inp

C	-0.143478	0.015495	-0.151099
C	-0.605378	-0.205961	1.250581
C	-1.412427	1.046417	1.133984
H	1.67988	1.901021	0.125315
H	-0.232102	-0.794103	2.075285
C	-1.094601	2.204564	2.058964
H	-1.560198	3.119417	1.684681
H	-0.015926	2.352654	2.132839
H	-1.490445	1.999182	3.057437
O	-0.892042	1.230656	-0.21721
H	-2.497099	0.884001	1.086544
O	1.215404	0.17482	-0.428673
H	-0.444808	-0.707689	-0.919545
O	1.799916	1.038458	0.54059

1032834335505263841702_r12_insertion_R_1_12_14.inp

C	-0.082935	-0.098019	-0.139084
C	-0.791633	-0.407079	1.191235
C	-1.356525	1.016645	1.036836
H	-0.142204	-0.604726	2.041764
H	-1.554695	-1.178511	1.0946
C	-0.673433	2.063519	1.846229
H	1.421653	2.08719	0.603154
H	-0.685822	3.083052	1.475987

H	-0.573538	1.919994	2.91571
O	-0.924879	1.044867	-0.347717
H	-2.447129	1.106396	1.098083
O	1.2519	0.214464	-0.066779
H	-0.154063	-0.821839	-0.95777
O	1.195548	1.330878	1.159624

1032834225145074161852_Intra_R_Add_ExoTetCyclic_F_2_12_14.inp

C	-0.0507	-0.038229	-0.014405
C	0.153968	-1.495206	0.078356
C	-0.319893	-1.408269	1.497887
H	1.862307	1.765336	1.029484
H	0.528861	-2.253717	-0.590133
C	0.635803	-1.693349	2.635646
H	0.195447	-1.36147	3.578218
H	1.578479	-1.168559	2.476439
H	0.828999	-2.767444	2.702603
O	-0.60581	0.004726	1.299243
H	-1.264151	-1.952843	1.633462
O	1.309746	0.052416	-0.01847
H	-0.581267	0.564421	-0.748844
O	1.738491	1.763398	0.072032

1032834335505263841702.inp

C	-0.13950369	0.01618702	-0.15415068
C	-0.62366328	-0.3021709	1.259919
C	-1.42558713	1.01872102	1.14016089
H	0.16968077	-0.27437993	2.00392703
H	-1.21715115	-1.20917012	1.36167405
C	-1.14529137	2.08908383	2.10903269
H	1.66014685	1.85903329	0.21465263
H	-0.11861958	2.38980155	2.28368611
H	-1.93938209	2.58193329	2.65513783
O	-0.82502348	1.25752457	-0.17633196
H	-2.49978196	0.85939775	1.02028271
O	1.22699053	0.14512678	-0.39892651
H	-0.45182779	-0.6575492	-0.95837549
O	1.81125784	0.98329184	0.59118592

1032834225145074161852_r12_insertion_R_10_1_12.inp

C	0.187382	0.144608	0.043138
C	-0.331853	1.326367	0.534406
C	-0.425944	1.796873	-0.892581
H	2.682671	0.445286	-1.27951
H	-0.541838	1.740477	1.505423
C	0.393798	2.9677	-1.373883
H	0.40951	2.993619	-2.466277
H	1.414206	2.907829	-0.996801
H	-0.06886	3.892451	-1.017692
O	0.116836	0.473932	-1.28565
H	-1.455025	1.845005	-1.262499
O	2.093474	0.22063	0.467231
H	0.188516	-0.889164	0.357278
O	2.728337	0.977693	-0.473852

170170000000000000002.inp

H	2.001435	1.553842	-1.3013
O	2.311839	2.390829	-1.682836

1032553363282331340492_r12_insertion_R_10_1_2.inp

C	0.231386	0.1624	0.199368
C	-0.310642	1.24617	1.593837
C	-1.763969	1.264595	1.63623
H	0.183526	2.15767	1.268089
H	0.182041	0.767049	2.437513
C	-2.476398	0.573206	2.533856
H	1.57795	1.308591	-1.129656
H	-3.559625	0.604546	2.542411
H	-1.992124	-0.033387	3.293831
O	-0.269555	0.661513	-0.827683
H	-2.271851	1.849681	0.875947
O	1.620803	0.161309	0.349051
H	-0.11654	-0.795692	0.614942
O	2.1791	1.242719	-0.364854

1032834335505263841702_r12_insertion_R_1_2_3.inp

C	0.03989	0.057574	0.0217
C	-0.670233	-1.232759	0.292628
C	-1.056053	-0.023369	1.936392
H	-0.064744	-2.09221	0.552934
H	-1.599132	-1.428534	-0.230016
C	-0.543441	-0.465403	3.114417
H	2.08491	1.439818	0.635139
H	0.506032	-0.332514	3.346971
H	-1.166669	-1.026836	3.799002
O	-0.32261	0.862453	1.130778
H	-2.132834	0.023409	1.789988
O	1.415365	-0.208496	0.037658
H	-0.215207	0.58453	-0.901806
O	2.093418	1.010997	-0.230549
330490170000000000002.inp			
H	1.070214	0.605446	0.466478
O	2.83639	0.663094	-0.030741
O	1.861545	1.106705	0.725703
1032834225145074161852_beta_delta_2_3_10_1_12.inp			
C	-0.087831	0.23924	-0.195338
C	-0.875023	-0.302933	-1.224083
C	-1.695671	-1.279283	-0.431912
H	2.393957	0.750253	0.954509
H	-0.425435	-0.563778	-2.176322
C	-2.129989	-2.535675	-1.160691
H	-2.527323	-3.26032	-0.446807
H	-1.280181	-2.989836	-1.675745
H	-2.911781	-2.304475	-1.88974
O	-0.620798	-1.482832	0.478773
H	-2.556354	-0.816194	0.07319
O	1.186848	0.531691	-0.4504
H	-0.490957	0.725193	0.685324
O	1.721813	1.340772	0.592617
1032794335695244221482.inp			
C	1.343715	0.069622	0.136421
C	-0.166748	-0.089963	0.04254
C	0.137717	-0.731589	-1.325341
H	-0.710497	0.853955	0.004671
H	-0.602353	-0.748343	0.791774
C	-0.332405	-0.008231	-2.56218
H	-0.08767	1.055005	-2.502341
H	0.145459	-0.425673	-3.451207
H	-1.41582	-0.116884	-2.668466
O	1.565367	-0.563134	-1.089723
H	-0.094192	-1.798477	-1.376836
O	1.702852	1.47207	0.080566
H	1.91508	-0.360684	0.960595
O	2.947069	1.668725	0.418825
70206300351252119006l.inp			
C	-0.160677	-1.209467	-0.143398
C	-1.280512	-1.273483	0.577203
C	-1.260962	0.239455	0.578788
H	-1.923896	-2.039362	0.974018
C	-1.052823	0.979634	1.874339
H	-0.808317	2.027222	1.682809
H	-0.242496	0.522066	2.445635
H	-1.967911	0.942915	2.471915
O	-0.016453	0.15613	-0.210759
H	-2.027557	0.704349	-0.046686
H	0.552237	-1.867149	-0.622845
1032553323472351320382.inp			
C	0.081387	0.104526	-0.069926
C	1.589425	0.019226	0.004176
C	2.220663	1.381354	0.022597
H	1.927798	-0.547196	-0.869983
H	1.860937	-0.544098	0.900431
C	2.972161	1.84125	1.014991
H	-1.272715	0.713699	-1.271691
H	3.409312	2.832644	0.978729
H	3.183303	1.240746	1.895372

O -0.314042 0.642061 -1.269375
H 2.028092 2.005734 -0.845813
O -0.406908 -1.282388 0.049151
H -0.355946 0.629117 0.78763
O -1.700162 -1.321135 -0.072289
1032794635545263921102.inp
C -1.21521039 1.64977556 0.3313528
C -0.66285295 0.95832198 -0.97029331
C 0.03055025 0.01715176 0.02291636
H 0.01978221 1.60711566 -1.51549775
H -1.42656434 0.53878013 -1.62050467
C 1.54480039 -0.04137069 -0.0160471
H 1.80254021 1.71732124 0.73191601
H 1.89216664 -0.62600641 0.84668868
H 1.86717636 -0.55538456 -0.92569499
O -0.4205595 0.7851698 1.1574742
H -0.38846092 -0.99467281 0.04337699
O -1.00402941 2.93327406 0.41118676
H -2.29316383 1.44692918 0.48504503
O 2.12743995 1.23889201 -0.03629398
1032513323492221340512.inp
C 0.06644087 0.0163775 -0.22055486
C 1.41344762 0.06594541 0.36350959
C 2.01840618 1.4949355 0.20041175
H 2.06348932 -0.67170022 -0.10872816
H 1.31819834 -0.13793476 1.43634616
C 3.29270425 1.68498318 1.02483179
H 3.6735058 2.6963918 0.87891479
H 4.05986995 0.9740505 0.70617924
H 3.08954178 1.53120198 2.08664809
O 2.13432921 1.85662013 -1.08997297
H 1.23545662 2.18250175 0.60867271
O -0.88912039 0.66758158 0.26739402
H -0.21284987 -0.54330685 -1.1073178
O -0.64310297 1.41293004 1.37502803
1032794335695244221482_r12_insertion_R_12_1_10.inp
C 1.54506054 -0.00457165 -0.2306093
C 0.05865664 0.09528615 -0.16783168
C -0.37514791 -0.85265009 -1.29375495
H -0.22445972 1.12377793 -0.39014608
H -0.32432511 -0.17856516 0.82150659
C -1.68578554 -0.4685856 -1.96287174
H -1.89512616 -1.13594682 -2.80123303
H -1.63398968 0.55720264 -2.33363773
H -2.50656131 -0.55091986 -1.24494262
O 0.70803705 -0.69678619 -2.1667961
H -0.42898753 -1.8876926 -0.92319807
O 2.32589574 1.0077991 -0.20712601
H 2.09204195 -0.93852918 -0.18490449
O 1.79249825 2.22521054 -0.14139965
862383593282392020981.inp
C 2.488855 1.209253 1.272796
C 3.707432 1.983212 1.425671
C 1.244855 -0.046006 -0.250177
H 4.186213 2.363031 0.528206
H 3.897239 2.526208 2.347069
C 1.15768 -1.129054 0.512431
H 1.709622 -1.249907 1.435849
H 0.527099 -1.94464 0.185545
O 1.969843 1.081293 0.001243
H 0.726281 0.051487 -1.196196
O 3.698822 0.554522 1.544176
H 1.736264 1.140634 2.056958
1032834335505263841702_r13_insertion_ROR_3_2_1_10.inp
C -0.156547 0.07359 -0.250754
C -0.892528 -1.100923 -0.817724
C -0.768395 -1.449177 1.225767
H -0.320738 -1.821472 -1.389475
H -1.932697 -0.972075 -1.09303
C -0.029373 -2.531258 1.584921

H	2.086772	0.834391	0.67249
H	1.052728	-2.48544	1.593885
H	-0.517289	-3.475959	1.789288
O	-0.174856	-0.179195	1.143896
H	-1.840651	-1.432576	1.407578
O	1.155105	0.023843	-0.740787
H	-0.575468	1.065663	-0.440259
O	1.854875	1.142329	-0.213409
70182196150104060081.inp			
C	1.337397	2.192629	0.986948
C	1.926969	3.374659	1.082449
C	1.282576	-0.053615	0.351502
H	2.92832	3.538836	0.705046
H	1.399426	4.195835	1.547067
C	1.817967	-1.136657	-0.190973
H	2.816654	-1.124725	-0.608786
H	1.247744	-2.054573	-0.218831
O	1.956655	1.12651	0.411934
H	0.281905	-0.039717	0.777694
H	0.33281	2.002149	1.35803
86262474628658466 1681.inp			
C	-0.347601	-0.710058	-0.798524
C	-0.353841	-1.005654	0.62028
C	-1.393786	0.116477	0.725355
H	-0.226084	-1.904889	1.205591
C	-1.095433	1.337929	1.553484
H	-1.792143	2.141466	1.304971
H	-0.078492	1.683408	1.363019
H	-1.20446	1.103848	2.616295
O	-1.269932	0.339849	-0.71911
H	-2.389421	-0.292177	0.919695
O	0.791646	-0.422531	-0.021009
H	-0.232293	-1.25919	-1.722224
103283433550526384 1702_intra_H_migration_6_7.inp			
C	-0.02263	-0.036299	-0.012395
C	-0.842011	0.585297	1.124543
C	-1.37666	1.498364	0.000226
H	-0.29255	1.040272	1.944824
H	-1.60089	-0.103986	1.491028
C	-0.653792	2.802697	-0.156708
H	0.625845	2.375139	-0.018112
H	-0.683865	3.223327	-1.160355
H	-0.772592	3.526058	0.649081
O	-0.866758	0.56706	-0.987176
H	-2.463586	1.605537	-0.06804
O	1.307624	0.401062	-0.137271
H	0.044126	-1.117142	-0.136506
O	1.491411	1.679802	0.397436
10325532131121414 90832.inp			
C	0.07508	0.048799	-0.04175
C	-0.812625	0.790991	0.604633
C	-2.287553	0.46511	0.576155
H	2.522804	1.017076	1.142483
H	-0.499349	1.666384	1.159565
C	-3.150492	1.670889	0.157523
H	-4.209652	1.420269	0.229765
H	-2.91471	1.945934	-0.872513
H	-2.936465	2.527092	0.801007
O	-2.626164	-0.65128	-0.120879
H	-2.61053	0.203721	1.610269
O	1.420119	0.226014	-0.121142
H	-0.188473	-0.828515	-0.622204
O	1.817819	1.385721	0.599583
103283433550526384 1702_intra_H_migration_12_5.inp			
C	-0.185489	0.094301	-0.129594
C	-0.302587	-0.653233	1.192711
C	-0.815663	0.519153	2.014212
H	0.664471	-1.010694	1.543597
H	-1.008812	-1.482745	1.158421
C	-0.009761	1.25083	2.843914

H	1.911396	1.526189	0.175336
H	1.060358	1.07595	2.867252
H	-0.408703	2.067667	3.433224
O	-0.600964	1.360293	0.27112
H	-1.889691	0.643383	2.093314
O	1.091118	0.10807	-0.730537
H	-0.80394	-0.290851	-0.950046
O	2.046834	0.570855	0.215504
320320000000000000003.inp			
O	0.1257704	2.53912219	0.86130441
O	-0.7681184	2.30694881	1.62074459
1032834225145074161852_r12_insertion_R_1_10_3.inp			
C	0.043497	0.057952	0.203046
C	-0.457497	1.113058	1.133271
C	-1.521788	1.632247	0.37801
H	2.041899	1.279045	-0.708438
H	0.23145	1.750584	1.673729
C	-1.874594	3.079327	0.316224
H	-1.075015	3.71258	0.701191
H	-2.792856	3.262869	0.884008
H	-2.078703	3.355089	-0.721071
O	-0.369721	0.805295	-0.92087
H	-2.304877	0.954142	0.053008
O	1.41346	-0.229683	0.212848
H	-0.406091	-0.941168	0.252746
O	2.161052	0.980561	0.202885
1032553252922271680662.inp			
C	-0.039152	0.067202	-0.037733
C	-0.599284	-1.323713	0.146029
C	-1.649551	-1.773733	-0.529846
H	2.208876	1.409872	0.077482
H	-0.08456	-1.936115	0.879964
C	-2.236139	-3.139467	-0.375776
H	-2.216011	-3.675332	-1.329407
H	-3.284131	-3.073152	-0.067772
H	-1.694215	-3.729643	0.365494
O	-0.091093	0.746003	1.119926
H	-2.128556	-1.119711	-1.256387
O	1.274412	-0.093591	-0.549461
H	-0.643202	0.639649	-0.767509
O	1.810048	1.202485	-0.776162
1032794335695244221482_r12_insertion_R_2_1_12.inp			
C	1.30460306	-1.20288868	-0.02605897
C	0.54687505	-0.01619516	-0.05105645
C	0.00303907	-0.58358697	-1.37219917
H	1.16040819	1.01964982	-0.21937918
H	-0.07947904	0.35097005	0.7725998
C	0.21268699	0.18037031	-2.64721834
H	-0.00500863	-0.44550091	-3.51507706
H	1.23912514	0.5461068	-2.71389062
H	-0.45867404	1.04254599	-2.65931444
O	0.97276889	-1.70217861	-1.21786844
H	-0.99675404	-1.0112698	-1.28851853
O	1.3100764	2.44724522	-0.07569094
H	2.00859234	-1.71023372	0.62076517
O	0.40853585	2.77059838	0.74258687
1032553363282331340492.inp			
C	0.129869	0.544956	0.449796
C	-0.111957	-0.938362	-0.024009
C	-1.552333	-1.215909	-0.306136
H	0.278167	-1.578088	0.771151
H	0.508096	-1.065294	-0.913285
C	-2.028451	-1.476197	-1.518282
H	2.420086	1.725736	-0.794688
H	-3.080899	-1.67971	-1.680446
H	-1.379877	-1.502957	-2.389486
O	-0.455565	0.7288	1.60401
H	-2.225854	-1.192192	0.546096
O	1.534049	0.797593	0.561282
H	-0.290396	1.208072	-0.331757

O	2.058149	0.834066	-0.764361

1032834335505263841702_intra_H_migration_12_4.inp

C	-0.191343	0.097342	-0.128188
C	-0.295809	-0.67793	1.179091
C	-0.783191	0.481377	2.034182
H	0.672576	-1.053455	1.50668
H	-1.011629	-1.498851	1.137747
C	0.04405	1.186593	2.865823
H	1.923223	1.503213	0.175918
H	-0.336427	1.994163	3.479601
H	1.112567	1.000566	2.8668
O	-0.588688	1.357995	0.306278
H	-1.854409	0.614453	2.13415
O	1.076331	0.112544	-0.747786
H	-0.825525	-0.26392	-0.947488
O	2.050129	0.54603	0.193695

621181110620190000001.inp

C	-0.003805	0.010068	0.108698
H	2.324289	1.650903	0.547087
O	-0.696024	-0.855845	0.530777
O	1.29764	0.099768	0.496555
H	-0.276846	0.802899	-0.60225
O	1.91157	1.186354	-0.189726

411131060600080000002.inp

C	-0.568269	-2.636325	-0.109439
C	-1.558047	-1.695334	-0.317819
H	-0.251427	-2.910994	0.888878
H	-0.080543	-3.126861	-0.945472
C	-2.012681	-1.295976	-1.559735
H	-2.792906	-0.552336	-1.663187
H	-1.597123	-1.719671	-2.468172
H	-2.011557	-1.236322	0.55806

1032593183022431560572.inp

C	-0.210381	-0.52167	-0.061833
C	0.245603	-1.854653	-0.513979
C	-1.760537	0.294479	1.524124
H	0.184037	-2.694904	0.162737
H	0.703368	-1.954405	-1.488068
C	-2.152919	0.472104	2.777727
H	0.665687	1.805115	1.092675
H	-1.731854	-0.108724	3.588946
H	-2.922308	1.19948	2.996875
O	-0.838314	-0.649118	1.186447
H	-2.162333	0.869142	0.692798
O	0.947092	0.290289	0.018235
H	-0.897335	-0.044337	-0.77671
O	0.526013	1.647263	0.151362

1032593183022431560572_intra_R_migration_2_12.inp

C	-0.008529	0.039765	-0.106112
C	-0.404453	1.161969	-0.794598
C	0.053764	-2.270333	0.090135
H	-0.845047	1.074394	-1.778725
H	-0.18283	2.13887	-0.389178
C	-0.498585	-3.458156	-0.092173
H	2.933072	1.413872	-0.26522
H	-1.355576	-3.590031	-0.740811
H	-0.078877	-4.321444	0.405161
O	-0.433744	-1.160363	-0.55069
H	0.926111	-2.092192	0.710192
O	1.840661	-0.036013	-0.652618
H	0.273866	0.097917	0.941676
O	2.59995	0.671286	0.251451

712193284022841360122.inp

C	0.20826218	-1.87924295	0.80170635
C	-0.12864092	-0.42684801	0.64251451
C	-0.94118935	-0.94148323	-0.56585098
H	0.70330496	0.23005631	0.36243342
H	-0.69806009	0.04172009	1.44837558
C	-0.59868835	-0.40910324	-1.93315256
H	-1.07606596	-1.00914783	-2.71127796

H	0.48248582	-0.42822061	-2.0918197
H	-0.94882543	0.62291786	-2.02775263
O	-0.41444843	-2.28578423	-0.35927329
H	-2.02151243	-0.94943521	-0.39748658
H	1.10161699	-2.39781496	1.13142684

103279433569524421482_intra_H_migration_14_4.inp

C	1.55748255	-0.01709585	-0.21343474
C	2.14845234	1.38891842	-0.37354671
C	3.05825706	1.03152258	0.78422365
H	1.00540884	1.8509996	0.16872688
H	2.44702936	1.88286178	-1.29346527
C	3.03761862	1.87281933	2.04018729
H	3.59631604	1.37010472	2.83232635
H	2.01111323	2.02593407	2.37712509
H	3.50497688	2.8427409	1.84873044
O	2.36486768	-0.2337787	0.93346386
H	4.08969094	0.8511326	0.45523357
O	0.18848358	0.10294693	-0.00315205
H	1.67840181	-0.7991967	-0.96625313
O	0.01376245	1.32834506	0.64916837

1032553363282331340492_intra_H_migration_10_7.inp

C	0.085907	0.266476	-0.209301
C	1.583965	0.254428	-0.668523
C	2.27112	1.50383	-0.218948
H	1.56123	0.182113	-1.759058
H	2.063749	-0.638401	-0.262539
C	3.311672	1.51789	0.605594
H	-1.577125	0.062899	-1.362391
H	3.787173	2.447383	0.897281
H	3.730294	0.600891	1.01074
O	-0.649427	1.189382	-0.708743
H	1.866307	2.434723	-0.605842
O	-0.409444	-1.090971	-0.561581
H	0.071096	0.212671	0.902232
O	-1.510194	-0.943814	-1.337395

862624746527626062121.inp

C	0.4352	0.205381	-0.748791
C	0.678131	-1.304298	-0.880312
C	-0.846374	-1.201649	-0.660358
H	1.300376	-1.796483	-0.131964
H	0.920862	-1.606402	-1.896558
C	-0.916222	-0.628978	0.7602
H	-1.883904	-0.178111	0.98627
H	-0.627892	-1.336033	1.541855
O	-0.818188	0.038467	-1.425647
H	-1.577462	-1.935348	-0.989512
O	0.083457	0.398483	0.610496
H	1.071434	1.007916	-1.108766

1032593183022431560572_Cyclic_Ether_Formation_2_12.inp

C	-0.100105	-0.522807	-0.320934
C	0.819551	-1.478418	-0.951513
C	-1.75989	-0.311372	1.324523
H	1.219044	-2.28314	-0.350147
H	1.162465	-1.317702	-1.963043
C	-2.491343	-0.760672	2.333167
H	1.09191	1.574986	1.506242
H	-2.358661	-1.760263	2.72822
H	-3.235795	-0.115375	2.77836
O	-0.810336	-1.094396	0.729666
H	-1.850744	0.692637	0.921733
O	1.038873	0.162103	0.032423
H	-0.752495	0.057964	-0.98186
O	0.567519	1.685466	0.703996

S4.1.7 *anti*-ROO2

1032834075575353511512.inp

C	-0.57933783	-0.70651875	-1.0103483
C	-0.01753696	0.14699522	0.13875043

C	-0.77888991	-0.81406281	0.99029805
C	-0.61303339	-1.33398968	2.36166683
H	0.23045884	-2.03469336	2.43492041
H	-1.51631091	-1.86107898	2.68186839
H	-0.42903609	-0.50973702	3.05399145
O	-1.16775055	-1.62862186	-0.05333854
H	0.18017248	-1.19929179	-1.61658188
H	-1.34685005	-0.23865887	-1.62834736
H	2.53515986	1.41151556	-0.12329984
O	1.37999542	0.00949979	0.34284086
H	-0.28858443	1.20706551	0.16284715
O	2.03118528	0.79608089	-0.66568023
70178203157105057 0001.inp			
C	1.10931504	0.00471183	-0.00000428
C	1.75728036	1.32329396	-0.00003822
C	1.02944922	2.44193785	0.00003329
C	1.57089663	3.83171355	0.00000814
H	1.21548432	4.37947681	-0.87835087
H	2.66202148	3.84128129	-0.00007798
H	1.21562313	4.37945464	0.87843712
O	1.6993496	-1.0475063	-0.00006501
H	-0.00233977	0.03086467	0.00008405
H	-0.05689988	2.34662605	0.00011769
H	2.8436312	1.33940329	-0.0001237
17017000000000000002.inp			
H	1.66609011	1.61751711	-2.1980992
O	0.85449489	1.08488789	-2.2077398
10325532133727814200062_intra_R_migration_3_14.inp			
C	2.01465803	-0.90650741	0.84003468
C	1.42695734	0.11617062	-0.13689051
C	-0.00629216	-0.06667921	-0.45254039
C	-0.86456352	-1.19855515	-0.0732028
H	-1.67485556	-1.35612294	-0.78668638
H	-1.32274506	-0.92804337	0.89384441
H	-0.30726388	-2.12278538	0.08013282
O	2.06782703	-2.08459165	0.61512588
H	0.05758464	2.28640685	1.35883989
H	2.41229222	-0.45387081	1.7679717
H	-0.41616354	0.72536409	-1.07063616
O	1.59423253	1.4052131	0.30242164
H	2.00317685	0.02861192	-1.07811576
O	0.16129171	1.33061548	1.39921469
33049017000000000002.inp			
H	0.72532596	2.29254721	-0.36546402
O	2.03605143	1.70855832	0.7794252
O	1.52206861	2.66676847	0.04662282
10325532133727814200062_intra_R_migration_3_1.inp			
C	-0.88212447	0.29678294	0.17417847
C	0.76045104	0.16619789	-0.00045137
C	-0.07556227	-0.90340641	-0.50832455
C	-0.11792639	-2.24511548	0.16851939
H	0.71222085	-2.8611944	-0.18725929
H	-1.05425213	-2.75908556	-0.05579972
H	-0.03981971	-2.14127272	1.25119388
O	-1.3605517	0.15345174	1.32783908
H	2.86583058	0.84300586	-1.79665449
H	-1.28042278	1.0211571 -	0.54454841
H	-0.22457625	-0.88656285	-1.58336471
O	1.26803579	1.11279164	-0.85108252
H	1.25577314	0.06970088	0.96193034
O	1.9936593	0.45080237	-1.9154741
10325531934328013600062.inp			
C	-0.69703661	0.65836091	-1.1716299
C	0.04723676	-0.00609202	0.00268257
C	-0.31751312	-1.48407423	0.11006039
C	0.29197929	-2.14949609	1.34131401
H	-0.01037815	-3.19778573	1.39130548
H	-0.04411829	-1.65779376	2.25837066
H	1.38161796	-2.10745208	1.31378711
O	-1.86148503	0.75367459	-1.2922402

H	1.93912659	1.67217816	-1.01481999
H	-1.40860579	-1.54557627	0.14123825
H	0.00811669	-1.99300692	-0.80212228
O	1.43901438	0.07625754	-0.17268267
H	-0.24660974	0.5460525	0.90571164
O	1.8091269	1.44524946	-0.08588772

103283407557535351512_Intra_R_Add_ExoTetCyclic_F_3_1_9.inp

C	-0.70681962	0.76972765	1.11251456
C	-0.01817285	-0.0119756	0.01699798
C	-0.67376352	0.81393718	-1.03405143
C	-0.37182661	1.01831668	-2.46657921
H	-1.13084357	0.56728183	-3.11706861
H	0.60380656	0.5976041	-2.71814457
H	-0.36028089	2.09284093	-2.67345865
O	-1.57156843	1.4657087	-0.37612458
H	-0.18848191	1.63746434	1.5013172
H	-1.40734716	0.28141459	1.78125316
H	2.44270287	1.35704449	0.66362673
O	1.40808567	-0.05631408	0.01289427
H	-0.26530254	-1.08073009	0.02818915
O	1.89534529	1.28317464	-0.124823

1032794335955623191362.inp

C	0.02320657	-0.02843717	-0.02916686
C	1.54519327	0.00162275	0.02542162
C	1.48303561	-0.65404404	-1.36185941
C	2.17114541	-1.98167282	-1.55142744
H	1.86633464	-2.4303084	-2.49932555
H	1.91164923	-2.67148112	-0.74436695
H	3.25505349	-1.83957703	-1.56405168
O	0.04623031	-0.77116935	-1.25633753
H	-0.4230096	0.96367142	-0.1503387
H	-0.48132841	-0.57158114	0.7726587
H	1.75516358	0.05442936	-2.15229953
O	2.06098913	1.3410645	0.05202165
H	2.04123136	-0.57062561	0.80932167
O	3.36097666	1.35226923	-0.04486876

1032553213822780970062_intra_R_migration_1_3.inp

C	-0.826895	-1.131038	0.002918
C	-0.024102	0.062643	-0.14442
C	-1.031979	0.328495	1.033191
C	-0.545982	0.114742	2.447209
H	0.330415	-0.531397	2.495682
H	-1.353538	-0.307787	3.047804
H	-0.278097	1.088145	2.865131
O	-2.066313	0.966151	0.763618
H	-0.438557	-1.962422	0.57565
H	-1.692731	-1.272949	-0.625967
H	2.57269	1.225854	-0.087982
O	1.30724	-0.123735	0.185644
H	-0.206198	0.733942	-0.980765
O	1.843874	1.155706	0.538272

1032553213372781420062_Intra_R_Add_Endocyclic_F_3_8.inp

C	-0.47739221	-0.86531747	1.02882016
C	0.0775839	0.26859601	0.24981983
C	-0.81020838	1.27117096	0.95485621
C	-1.89256779	2.00041326	0.25559131
H	-2.66280845	2.31731002	0.96164778
H	-2.35319475	1.37898962	-0.51620921
H	-1.48908602	2.89991301	-0.22721881
O	-1.49191469	-0.40199946	1.65731893
H	2.64853907	1.07359007	-0.8032609
H	-0.12803426	-1.8894919	1.13088995
H	-0.38600691	1.70638146	1.85357444
O	1.47525762	0.40054588	0.49035783
H	-0.0919212	0.25315953	-0.83588975
O	1.94256944	1.50075489	-0.30774307

1032553213372781420062_r12_insertion_R_2_12_14.inp

C	-0.71793254	0.61054864	0.00356398
C	0.77019838	-0.00643482	0.18642821
C	0.61901348	-1.32126286	-0.4054757

C	-0.12947217	-2.41393521	0.251906
H	-0.65901475	-3.03412061	-0.47494774
H	-0.83853188	-2.03142043	0.99172952
H	0.57084094	-3.07379751	0.78395918
O	-1.65164791	0.34339523	0.68569146
H	1.83954413	1.57758616	-2.2548508
H	-0.68655657	1.33245143	-0.82930198
H	1.0712847	-1.46193943	-1.38163208
O	1.66141568	0.85084181	-0.29942163
H	0.8496104	-0.00911701	1.28614241
O	1.12198313	0.95445761	-2.10308883

702063003512521190061.inp

C	-0.95801252	-0.88765772	0.8176681
C	0.28706854	-0.42127863	0.718837
C	0.33337698	-1.08484182	-0.64006093
C	1.27102397	-2.24085092	-0.87276997
H	1.0079932	-2.76870749	-1.7926112
H	1.22599904	-2.9392749	-0.03457788
H	2.29610563	-1.87215057	-0.96794486
O	-1.04728657	-1.54275833	-0.38767184
H	-1.79323426	-0.87409231	1.50532988
H	0.31037444	-0.39200878	-1.48531262
H	0.96143955	0.16833547	1.31513331

1032834075575353511512_intra_H_migration_3_13.inp

C	-0.33709506	-1.23079923	0.4679285
C	-0.45134891	0.20376825	-0.12264481
C	-0.68996062	0.56150592	1.29505926
C	-0.72460533	1.76169997	2.12918194
H	0.30015312	1.98165497	2.46369384
H	-1.36250532	1.62484832	3.00592481
H	-1.06734647	2.61675201	1.54427679
O	-0.74057761	-0.68937975	1.76959444
H	0.69358815	-1.57420041	0.51678666
H	-1.03117584	-2.00270316	0.13706166
H	2.47121199	1.01862667	-0.24909949
O	0.55743898	0.78176015	-0.81343688
H	-1.34255944	0.33804914	-0.75429593
O	1.79576349	0.80889626	0.40145529

1022422782862701420061.inp

C	-0.322203	-0.91072	0.056807
C	-0.078786	0.552935	0.06566
C	-0.983983	1.536146	0.038784
C	-2.46845	1.403587	0.032616
H	-2.900609	2.0359	0.813679
H	-2.798465	0.375818	0.161521
H	-2.858092	1.771456	-0.92272
O	-1.375326	-1.443202	-0.187217
H	2.769957	0.119253	-0.514611
H	0.575142	-1.507527	0.313121
H	-0.57507	2.543796	0.054624
O	1.241907	0.908155	0.236718
O	1.956236	0.429796	-0.927066

1032794335955623191362_intra_H_migration_14_11.inp

C	1.56007312	-0.08607265	-1.7967004
C	1.56554957	-0.10047026	-0.25646566
C	2.20342562	1.29852178	-0.43690124
C	3.32843915	1.94676637	0.29377865
H	3.5097652	2.94731371	-0.10612762
H	4.2479181	1.36201709	0.18388589
H	3.08436443	2.0315715	1.35413974
O	2.33081758	1.14460407	-1.80946286
H	0.57710341	0.04965588	-2.24777478
H	2.119103	-0.88732053	-2.28058425
H	1.02070207	1.79488674	-0.19331807
O	0.36503738	0.04869697	0.43528227
H	2.13025581	-0.89975266	0.22428288
O	-0.16000977	1.26639361	-0.00038838

1032553213372781420062.inp

C	-0.8104308	0.52895314	-0.49424362
C	0.57832001	-0.09426303	-0.32407575

C	0.41514055	-1.55195238	-0.54555284
C	-0.0228636	-2.43030556	0.56778544
H	-0.09243624	-3.47125895	0.25089245
H	-1.00624421	-2.1248566	0.95379228
H	0.6694383	-2.37059852	1.41666331
O	-1.59294715	0.63319484	0.40974277
H	2.39347355	1.95661954	-0.86403278
H	-1.05684048	0.81508762	-1.53604649
H	0.43581224	-1.90246715	-1.57135747
O	1.46886571	0.38808118	-1.31413582
H	0.94020268	0.13029166	0.68829307
O	1.50124445	1.81072723	-1.19702255

1032834185635133391512_r12_insertion_R_2_3_8.inp

C	-0.02874436	0.01478055	-0.04160815
C	1.45632298	0.02961803	-0.06696909
C	1.51196068	-0.76779884	-1.35213635
C	2.06293284	-2.1422506	-1.41293777
H	1.64719498	-2.68379237	-2.26522869
H	1.84295947	-2.69897336	-0.49872955
H	3.15282102	-2.11183272	-1.53974365
O	-0.38232362	-0.81843259	-0.94615626
H	1.03557011	2.70262667	-0.67861819
H	-0.74270813	0.58127359	0.55312834
H	1.49645408	-0.17380076	-2.25885819
O	2.12037145	1.29641883	-0.06760072
H	1.96039889	-0.47941715	0.76475838
O	1.6094115	2.08325884	-1.14186094

29041017000000000002.inp

C	0.61771	0.33585169	2.03130768
O	-0.26325105	-0.0872144	2.67600102
H	0.53901106	1.22600971	1.3513483

1032553213822780970062_Cyclic_Ether_Formation_1_12.inp

C	-0.74914	-0.763686	-0.988954
C	-0.197681	0.086958	0.073298
C	-0.874657	-0.048453	1.452597
C	-0.687035	1.106045	2.392363
H	0.386335	1.268353	2.526676
H	-1.168268	0.901912	3.34744
H	-1.103494	2.0171	1.950364
O	-1.48355	-1.049668	1.73075
H	-0.548661	-0.536435	-2.026399
H	-1.263851	-1.672046	-0.706472
H	2.292456	-1.138072	1.420476
O	0.936558	-0.718983	-0.041156
H	0.000811	1.128923	-0.197327
O	2.049999	-0.2356	1.182332

86206272286182000000 1.inp

C	3.30755408	1.17342497	0.19685079
C	1.14510105	0.06821945	-0.21854226
C	1.81172877	1.41522588	0.01634291
C	1.19066563	2.20920167	1.15392214
H	0.13030216	2.3920186	0.96423406
H	1.6922441	3.16988278	1.2722155
H	1.28702268	1.67315815	2.1012814
O	3.94976085	1.6327793	1.09870297
H	3.7678748	0.53269127	-0.57955498
H	1.73074385	1.94863463	-0.94100238
O	1.39157785	-0.63784603	-1.1576259
H	0.41325989	-0.24283901	0.55623461

86262474628658466168 1.inp

C	-0.16823589	-1.10649622	-0.58629291
C	-0.47143033	0.27551459	-0.90884043
C	-0.50777179	0.49317415	0.60585329
C	-1.86113161	0.81484001	1.18622622
H	-1.85220398	0.66298808	2.26726033
H	-2.63173301	0.17167115	0.75381128
H	-2.11749129	1.8590154	0.9846722
O	-0.16943836	-0.9191706	0.80122624
H	-0.37514595	-2.08358106	-0.99965621
H	0.28573476	1.13480493	0.99502715

O	0.81216867	-0.29919471	-1.19333347
H	-1.02025021	0.7651143	-1.70135268

1032794335955623191362_HO2_Elimination_from_PeroxyRadical_9_14.inp

C	0.23801834	-0.32863952	0.18778491
C	1.61508834	-0.16294382	0.12539941
C	1.56070569	-0.79123281	-1.24118203
C	2.25391637	-2.12726628	-1.38982891
H	1.93522725	-2.60353257	-2.31912257
H	2.00593459	-2.78666341	-0.55527027
H	3.33743545	-1.98591614	-1.42390804
O	0.11923543	-0.94667507	-1.08966824
H	-0.03781654	0.97099135	0.0161214
H	-0.42466407	-0.63535855	0.99173342
H	1.78995334	-0.11878001	-2.07186273
O	1.56855245	1.98246047	-0.30980788
H	2.41160099	-0.00916096	0.83478802
O	0.32573855	2.16476378	-0.26408521

103255321337278142006 2_r12_insertion_R_1_2_12.inp

C	0.29100539	-1.03922422	0.40703316
C	0.17746337	0.38825379	0.01562884
C	-0.56770498	1.2517845	0.81089276
C	-0.936597	2.63372593	0.4127792
H	-1.99408793	2.83308678	0.61323497
H	-0.73229055	2.82061886	-0.64262403
H	-0.36334328	3.35986175	1.00246462
O	0.69076223	-1.90297003	-0.33472821
H	2.7543032	-0.64793619	-0.62429011
H	0.02997707	-1.26197728	1.46110302
H	-0.81157287	0.93551938	1.8232175
O	1.95125852	0.69497029	0.39605608
H	0.26560599	0.59456608	-1.04694581
O	2.67481611	0.31498976	-0.70639953

702062893452741310061.inp

C	1.00215266	-0.57308862	1.37493009
C	0.80810222	-0.61054638	-0.11850533
C	-0.37565717	-1.18440432	0.12263443
C	-1.55440109	-1.72003953	-0.59014518
H	-2.45691256	-1.18094345	-0.28982715
H	-1.69707167	-2.7765232	-0.34763635
H	-1.42103306	-1.61742982	-1.66758678
O	-0.31198156	-1.20510471	1.50430738
H	0.99911347	0.40547773	1.85919899
H	1.77408244	-1.21821523	1.79926781
H	1.37207433	-0.30947247	-0.98410091

1032553213372781420062_S42_insertion_R_13_2_1.inp

C	-0.494774	1.21856	0.790727
C	0.011919	0.115738	-0.083782
C	-0.605228	-0.450696	-1.146032
C	-2.039725	-0.327886	-1.517986
H	-2.578429	0.373633	-0.886383
H	-2.516508	-1.312947	-1.459767
H	-2.120236	-0.004494	-2.560805
O	-1.549833	1.782128	0.666972
H	2.765657	0.989941	0.398902
H	0.217418	1.488218	1.594591
H	0.012219	-1.124875	-1.734181
O	1.341824	-0.201823	0.127896
H	-0.56365	-0.986949	1.423355
O	2.102636	0.952056	-0.299682

1032553213372781420062_intra_H_migration_3_10.inp

C	-0.81589035	-0.16394377	1.27238667
C	0.10290144	0.3675045	0.20332661
C	-0.75644597	1.61329123	0.32699436
C	-1.89920541	1.82928979	-0.61039648
H	-2.58194097	2.5928252	-0.23464059
H	-2.4645385	0.90606713	-0.7648435
H	-1.52269837	2.16127348	-1.5853789
O	-1.64814974	-1.02749307	1.18409643
H	2.82842675	0.81564676	-0.47754719
H	-1.15287418	1.02274431	1.64843718

H	-0.24148098	2.47553246	0.74326051
O	1.40462512	0.51510705	0.69589234
H	0.0675918	-0.15124797	-0.75790224
O	2.09321857	1.38020171	-0.21605322
1032794335955623191362_intra_H_migration_14_9.inp			
C	2.06829063	1.36633782	-0.31689369
C	1.4810625	-0.05579953	-0.29875773
C	2.288022	-0.17005474	1.01573321
C	3.31558329	-1.26516132	1.11130601
H	3.97120411	-1.10301422	1.96909479
H	3.92879098	-1.30726274	0.20753914
H	2.81007163	-2.2261843	1.23923673
O	2.90769115	1.131976	0.74792043
H	0.91146595	1.79172392	0.11714737
H	2.47844386	1.95089594	-1.13964528
H	1.65702065	-0.09987784	1.90333662
O	0.09010432	0.03730252	-0.24211407
H	1.73690106	-0.77345261	-1.07944248
O	-0.16931909	1.18170585	0.51374625
1032834075575353511512_intra_H_migration_3_11.inp			
C	-0.91028638	0.73028527	0.90432607
C	0.1502613	0.04822999	0.01989998
C	-0.13550692	1.2482237	-0.91537476
C	-0.27845599	1.30220387	-2.39741855
H	0.64279273	0.96172958	-2.87334735
H	-0.48216572	2.32651495	-2.7186471
H	-1.10770874	0.66797622	-2.72841201
O	-1.20585177	1.68545465	-0.14895521
H	-0.51494639	1.22104448	1.79369424
H	-1.79353186	0.13297361	1.13182733
H	0.95186547	1.79207279	-0.43933775
O	1.49086209	0.09391754	0.39776165
H	-0.06797438	-0.96694052	-0.31273709
O	1.79675155	1.45186662	0.49953563
1032794335955623191362_HO2_Elimination_from_PeroxyRadical_11_14.inp			
C	1.84116117	-0.11785781	1.51334607
C	1.67264053	-0.19159318	0.02840252
C	0.30949777	-0.38613779	0.25425861
C	-0.72770691	-1.27482443	-0.3640152
H	-1.72753376	-0.98063272	-0.03915896
H	-0.56085921	-2.31554999	-0.07637836
H	-0.68007432	-1.19513515	-1.45223783
O	0.42275797	-0.3394687	1.68306496
H	2.1768501	0.82820899	1.94319608
H	2.41278557	-0.95410278	1.92900861
H	-0.00584284	0.84661521	-0.10323225
O	1.52948511	1.91424389	-0.56993304
H	2.35301755	-0.37399687	-0.78692354
O	0.28533658	2.05867312	-0.47058113
1032553213372781420062_HO2_Elimination_from_PeroxyRadical_11_14.inp			
C	-0.04221111	0.75332082	1.25676369
C	0.11404975	-0.0225356	-0.00445364
C	-0.29265386	0.44686732	-1.25312573
C	-0.44059817	1.87138391	-1.64029364
H	-1.0604721	1.97613155	-2.53228981
H	0.55663997	2.27537339	-1.86899238
H	-0.83836297	2.4839042	-0.82978622
O	0.06370963	1.95483059	1.3414469
H	2.20972368	1.86679354	0.51814984
H	-0.2139121	0.13930289	2.16132054
H	-0.38338734	-0.29926671	-2.03681333
O	1.94315922	0.06712178	0.08931592
H	0.08094769	-1.09865943	0.13980809
O	2.37896039	1.31941717	-0.26459052
1032834075575353511512_r12_insertion_R_1_2_12.inp			
C	-0.40682076	1.05417977	-1.24480457
C	-0.49218176	0.19706224	-0.00755696
C	-0.32135719	-0.87714868	-0.85179563
C	-0.21783917	-2.34636315	-0.79820943
H	-0.93090348	-2.81134392	-1.48495725

H	-0.41579362	-2.69616767	0.2151912
H	0.78588876	-2.67082693	-1.09226282
O	-0.11670305	-0.17384452	-2.00533645
H	0.43594445	1.74098224	-1.30242757
H	-1.33796768	1.49889	-1.59619468
H	2.65975094	0.01948368	-0.17614175
O	1.23229667	0.51885534	0.91595846
H	-1.0074959	0.31655036	0.93115
O	2.19920242	0.85001743	-0.00889178
1032834075575353511512_r13_insertion_ROR_3_2_1_8.inp			
C	1.6776901	1.53579253	-0.23345164
C	1.45931724	0.13280629	0.40234059
C	0.12244372	0.26991933	-0.22131867
C	-1.02753824	-0.57400453	-0.54190733
H	-1.95205344	0.00677047	-0.59017393
H	-1.12403082	-1.37454766	0.19324427
H	-0.85029422	-1.04524748	-1.52013086
O	0.26692365	1.53567716	-0.63293407
H	2.30970498	1.49253892	-1.11736356
H	1.89843327	2.38655224	0.41039403
H	2.69659087	-1.66659375	-1.70786754
O	2.24772097	-0.92484585	0.10471574
H	1.42385254	0.16096191	1.50199852
O	1.95320538	-1.06743532	-1.59911366
1032513323622861080002.inp			
C	-0.063422	0.22718434	-0.17174082
C	1.40072908	-0.14438611	0.04002629
C	1.97314788	-1.00167837	-1.07483311
C	3.37443924	-1.5219734	-0.77017816
H	3.73974207	-2.13776626	-1.59434494
H	3.37551891	-2.13706548	0.13421266
H	4.08013358	-0.70165665	-0.62363962
O	-0.89385256	-0.58492043	-0.46410395
H	1.27405055	-1.8287538	-1.22188828
H	-0.30771076	1.29736314	-0.02415074
H	1.97053583	-0.41831443	-2.00123215
O	2.18369466	1.07103563	0.11817465
H	1.48474773	-0.64073914	1.01334066
O	1.90396032	1.76072652	1.18815111
862624746847083821241.inp			
C	-0.93938665	-0.82085009	-1.44569313
C	-0.16389616	0.34754973	-0.85323833
C	-0.29023269	-0.35305745	0.41783659
C	-0.44608488	-0.05170867	1.86162866
H	0.09826587	0.8591477	2.110319
H	-0.03772189	-0.87842087	2.44844326
H	-1.50088184	0.06883324	2.11082718
O	-0.98635464	-1.4504546	-0.13705641
H	-0.41813057	-1.43341589	-2.18309941
H	-1.94767517	-0.56238064	-1.77550125
O	0.97295137	-0.24429107	-0.20162687
H	-0.11843374	1.3881276 -1.14196828	
1032553213372781420062_Cyclic_Ether_Formation_3_12.inp			
C	-0.60801145	0.59383888	-1.24618612
C	-0.13198045	0.00791876	0.0719312
C	0.13851597	-1.44309917	0.03814664
C	0.0235363	-2.30242665	1.23731338
H	0.83823401	-3.02773583	1.29297426
H	-0.91594262	-2.87099085	1.18998646
H	0.00821343	-1.70673816	2.15135209
O	-1.75290982	0.87530959	-1.46169532
H	2.44159476	1.80775333	0.1762042
H	0.19050488	0.71633928	-2.00621053
H	0.46960692	-1.8680068	-0.90335234
O	1.22979244	0.15808754	0.29006628
H	-0.76034461	0.34682906	0.90181548
O	1.49773017	1.86337987	0.36459738
1032553213372781420062_intra_H_migration_3_9.inp			
C	1.2620205	-0.02122699	-0.16571533
C	1.66326291	1.40458719	0.18638201

C	2.9035928	1.50180497	1.09160683
C	3.2747764	0.39530869	2.0281595
H	4.20770358	0.6278261 2	.5445374
H	3.38298659	-0.5622498	1.51465675
H	2.49405529	0.26006208	2.78723033
O	0.71294992	-0.74970663	0.61509521
H	3.63084423	1.53926088	-0.02682844
H	1.4905491	-0.32717464	-1.20490389
H	3.02920627	2.50998633	1.49084217
O	2.04371172	2.12586823	-0.97367323
H	0.79118702	1.91338822	0.61018211
O	3.33568348	1.64820283	-1.22609464
1032834075575353511512_Cyclic_Ether_Formation_3_12.inp			
C	-0.99753538	0.14181286	-1.25023572
C	-0.02806857	0.07021712	-0.04936348
C	-0.31664228	-1.37821119	-0.13765657
C	0.14530942	-2.62895111	0.48161606
H	0.80670175	-2.40201059	1.3171506
H	0.68616857	-3.24679945	-0.24206497
H	-0.71025835	-3.20910061	0.84500154
O	-1.066352	-1.3328749	-1.2454693
H	-0.54377172	0.50373749	-2.16960746
H	-1.98934139	0.55577544	-1.07146113
H	2.66803292	1.46680526	-0.82666573
O	1.28533042	0.0025725	-0.42158818
H	-0.22520971	0.67882827	0.83827532
O	1.75573236	1.6762077 -0.60188402	
1032834185635133391512_r12_insertion_R_2_12_14.inp			
C	0.05703794	0.10466926	-0.01113445
C	1.06633628	1.08681708	-0.44989566
C	0.53399097	1.88515762	0.77237693
C	-0.29514994	3.1106658	0.52679068
H	-0.84294441	3.39681774	1.4268916
H	-1.01152001	2.94392926	-0.28204933
H	0.36519049	3.93437072	0.24431016
O	-0.32359461	0.70655242	1.10647901
H	4.17149139	0.7453376	-0.14927728
H	-0.19483558	-0.92161146	-0.24158466
H	1.28757578	1.99442231	1.55015345
O	2.14222125	0.30629202	-0.1537741
H	1.08006324	1.56925125	-1.43174983
O	3.51421885	1.43202168	-0.30412997
862343734213371620571.inp			
C	1.13714147	-0.01567144	-0.01756113
C	1.80959484	1.31397577	0.02754532
C	2.19683121	1.95336747	-1.24440955
C	3.37320246	2.88279508	-1.32564951
H	3.19225966	3.65478416	-2.07736864
H	4.27119451	2.32929991	-1.611272
H	3.54869615	3.36923083	-0.36447743
O	1.42210346	-0.9287892	0.70858441
H	0.34915621	-0.10359512	-0.79501329
H	1.94647674	1.42281189	-2.16345931
O	1.06251524	2.39221862	-0.52367068
H	2.37981821	1.53186746	0.92714591
1032834185635133391512_r12_insertion_R_1_2_12.inp			
C	-0.14508678	-0.52791652	1.05991876
C	-0.02561838	0.17645491	-0.11044168
C	-0.4283134	1.39090131	0.70011409
C	-1.78918629	1.99417768	0.47995388
H	-2.03472794	2.68434013	1.2899078
H	-2.55685369	1.21914776	0.42727841
H	-1.78857862	2.55377466	-0.45915068
O	-0.40564517	0.50773975	1.89871021
H	2.87350266	0.521105	1.12566659
H	-0.04115104	-1.5407953	1.42329608
H	0.36276051	2.13192767	0.81198859
O	1.91472647	0.47453532	-0.47062171
H	-0.15896761	-0.0819729	-1.14784088
O	2.47879342	1.19203837	0.55588306

1032834185635133391512.inp

C	0.553101	1.112148	-0.107152
C	0.340945	-0.307141	0.290596
C	-1.14853	0.072337	0.17325
C	-1.964422	0.129774	1.437208
H	-2.919122	0.62592	1.250896
H	-1.430686	0.680237	2.215501
H	-2.163353	-0.884209	1.794048
O	-0.772216	1.401729	-0.309324
H	2.300399	-1.205295	-1.506811
H	1.333098	1.677631	-0.597819
H	-1.651776	-0.470827	-0.629017
O	0.62898	-1.315299	-0.673283
H	0.707386	-0.633363	1.267706
O	2.037071	-1.535441	-0.641085

1032513624072430760002.inp

C	-0.53781536	-0.2474259	1.37030752
C	0.11228724	-0.39061282	-0.03596001
C	1.64721902	-0.28025046	-0.03150888
C	2.20894506	-0.43057336	-1.43073016
H	1.90659227	-1.38481202	-1.86536137
H	3.29836458	-0.38245744	-1.39694463
H	1.84960827	0.38060632	-2.07039873
O	0.08268548	0.04009342	2.35067871
H	1.84114131	1.01441975	1.40117422
H	-1.62310079	-0.45426016	1.38084728
H	2.04161669	-1.07789623	0.6125772
O	-0.41540006	-1.48408247	-0.60830038
H	-0.30605673	0.50590558	-0.55192982
O	2.03207292	0.98974552	0.46001513

1032553213822780970062.inp

C	-0.87940169	0.15248322	-1.69527854
C	-0.21189093	0.25101729	-0.38050428
C	-1.11088308	-0.44962652	0.67446259
C	-0.93016139	-1.93267799	0.84664108
H	-0.87507352	-2.42800336	-0.1257536
H	-1.75337346	-2.33305418	1.4363698
H	0.01907131	-2.11845836	1.35582555
O	-1.92698689	0.20424616	1.27039308
H	-0.64623856	-0.6714503	-2.35756179
H	-1.71090213	0.80759249	-1.9143803
H	2.41237075	0.28528472	0.59378235
O	1.05216107	-0.37829505	-0.51155167
H	-0.0984148	1.29244534	-0.05516571
O	1.68983354	-0.32904855	0.76300855

1032834185635133391512_intra_H_migration_1_13.inp

C	-0.30641428	-0.87222082	-0.11528068
C	0.01290818	0.56317279	0.18587785
C	-0.77856319	0.20405296	1.49876985
C	-2.05321687	0.94021398	1.79344803
H	-2.61148218	0.43147344	2.58252782
H	-2.68200469	1.00146712	0.90232745
H	-1.81908891	1.95349093	2.12884445
O	-1.01784774	-1.15171631	0.98218485
H	2.80960104	0.6803689	-1.1067618
H	0.24203066	-1.65000749	-0.6354262
H	-0.10176854	0.12489501	2.34703266
O	1.32997763	0.76481131	0.36362643
H	-0.49397257	1.30646152	-0.44514425
O	1.99896644	0.17385783	-1.22694293

S4.1.8 *syn*-ROO2

1032794335955623191362_intra_H_migration_12_13.inp

C	2.07764012	1.40576825	-0.36621709
C	1.56965142	-0.03447703	-0.17961804
C	2.40944314	-0.04789862	1.13986975
C	1.72290977	-0.09031065	2.47980752
H	0.85945307	0.57694811	2.50654878

H	2.42764722	0.18992086	3.27013138
H	1.37590983	-1.11226487	2.67722443
O	2.94326568	1.35350811	0.76288695
H	3.27093829	-0.71913055	1.08603041
O	0.12025033	-0.01480703	-0.09187553
H	1.81449043	-0.80790851	-0.9091592
O	-0.21515004	1.36847318	0.43862611
H	0.87240259	1.87415086	0.01206065
H	2.45853064	1.90917494	-1.2510111
862102582121340940381.inp			
C	2.65571696	1.5200261 -1.0752083	
C	3.10038175	2.95785032	-0.96709483
C	1.31904038	-0.02852282	0.26200246
C	1.44456349	-1.0693643	-0.57302712
H	2.00413377	-1.03617113	-1.50069867
H	0.96581394	-2.0088396	-0.32425027
O	1.86012715	1.24419692	0.10702878
H	0.75955525	-0.0630388	1.18871923
O	3.80085138	3.50353171	-1.82638216
H	2.75896392	3.49410941	-0.06784681
H	3.52954145	0.85415913	-1.12073976
H	2.06048598	1.37368042	-1.98806446
1701700000000000000002.inp			
H	1.78924514	1.78410663	-0.63037165
O	2.59094786	2.36979537	-0.70193635
3304901700000000000002.inp			
O	1.03744247	2.72568189	0.86824418
O	-0.21250175	2.29993051	0.41504325
H	-0.21382972	1.3104226	0.47894057
1032794335955623191362.inp			
C	-0.13652528	-0.0737247	-0.02373323
C	1.38915304	0.00398199	0.09327185
C	1.48372214	-0.74274602	-1.26711421
C	2.08700417	-0.03981072	-2.46096989
H	1.67826832	0.96732761	-2.57722179
H	1.86506066	-0.61301411	-3.36742334
H	3.17629432	0.03399604	-2.35609795
O	-0.02819472	-0.80485944	-1.32992225
H	1.87662351	-1.75785137	-1.15184874
O	1.82513566	1.42236707	0.06597324
H	1.87544966	-0.46269308	0.94905102
O	3.18687131	1.54482403	0.30213967
H	-0.63209834	0.89180466	-0.14545202
H	-0.64143473	-0.6779703	0.73247131
1032834335765453171472_Cyclic_Ether_Formation_4_10.inp			
C	-0.86337321	0.09355717	-1.35626262
C	-0.16996946	-0.02880803	0.00608407
C	-0.23849381	-1.56126278	-0.15123823
C	1.20506712	-1.93885535	-0.25089338
H	2.36631231	1.9091997	0.01864774
H	1.63379021	-2.13007899	-1.22787006
H	1.74594916	-2.27207914	0.62903862
O	-0.90734255	-1.42011015	-1.48498091
H	-0.84188983	-2.15673545	0.53889112
O	1.29900314	0.13079458	-0.04043107
H	-0.57818674	0.50302453	0.86300595
O	1.38355325	1.8615039	-0.03048529
H	-0.26040132	0.54863546	-2.14184359
H	-1.87629368	0.50092484	-1.34094551
702063003512521190061.inp			
C	-0.78562312	-1.1048719	1.0080742
C	0.47671487	-0.70274462	0.77288154
C	0.45353784	-1.42655781	-0.57203651
C	0.503145	-0.64297711	-1.86179443
H	-0.15692582	0.22798722	-1.80778983
H	0.19348144	-1.27005379	-2.70500676
H	1.52701686	-0.29491491	-2.05010615
O	-1.00874379	-1.82707772	-0.20856161
H	1.03403862	-2.35237279	-0.60876871
H	1.21789748	-0.12343612	1.29869852

H	-1.55248139	-1.03958947	1.76426975
1032834335765453171472.inp			
C	-0.50368546	-1.44812335	-0.08510568
C	0.00000901	0.00001363	0.00015196
C	-0.71139189	0.1522498	1.38067869
C	0.10105479	0.2588137	2.58874714
H	2.81526506	1.38624162	-0.03626491
H	1.09325702	-0.1766305	2.62011153
H	-0.31272418	0.67914316	3.49884601
O	-1.28679111	-1.295715	1.18321476
H	-1.55979461	0.83867101	1.36645209
O	1.44446529	-0.00015525	0.00035665
H	-0.35054065	0.68846555	-0.77088634
O	1.83635548	1.48580519	0.00001064
H	0.28208256	-2.20076545	0.01654049
H	-1.15468555	-1.68063773	-0.93027401
1032553363542781120042.inp			
C	-0.51680503	1.21537921	-1.08743637
C	-0.03074105	0.09544782	-0.1512063
C	-0.5574951	-1.26322305	-0.51818138
C	-1.28455678	-2.02396558	0.3106049
H	2.93709584	1.02290789	0.35218928
H	-1.52324089	-1.7045557	1.32187387
H	-1.67775621	-2.98699744	-0.00055778
O	-1.87798847	1.5392754	-0.90346186
H	-0.33212451	-1.59973359	-1.52910048
O	1.4355928	-0.04073327	-0.28928638
H	-0.26081583	0.35040474	0.88826729
O	2.00964707	1.30484649	0.18397904
H	-0.31323836	0.98214359	-2.14455952
H	0.04414369	2.14178792	-0.86198681
1032834335765453171472_r12_insertion_R_1_2_3.inp			
C	-0.2827243	-1.2147184	-0.17522177
C	0.11063258	0.23324189	0.08670138
C	-0.86557979	-0.13875507	1.70750103
C	-0.23412722	-0.02076039	2.94630344
H	2.87587603	1.44073318	-0.72072821
H	0.4238021 -0.80427182	3.30563474	
H	-0.31696705	0.89520897	3.52231927
O	-0.97128938	-1.47285481	1.1222804
H	-1.73841575	0.49045841	1.51843858
O	1.41392974	0.52958967	0.3463794
H	-0.42551871	1.02083759	-0.4449387
O	2.10219827	0.96282865	-1.1034529
H	0.57885517	-1.8783736	-0.27160872
H	-0.97779593	-1.3557879	-1.00702892
1032794335955623191362_intra_H_migration_12_5.inp			
C	-0.19549407	-0.59069113	-1.3397867
C	-0.05402375	-0.05037904	0.093928
C	1.46595346	-0.47033959	0.02594056
C	2.46210453	0.6378112 0.01421392	
H	1.56960874	1.73591128	-0.39278804
H	3.17936316	0.61878549	-0.80590419
H	2.83755111	0.98489009	0.97753165
O	1.23559027	-1.06242703	-1.33059805
H	1.72558022	-1.25757494	0.74633464
O	-0.28634137	1.35483892	0.33778407
H	-0.65230436	-0.54028884	0.86371051
O	0.53415213	2.14045511	-0.6394805
H	-0.34872799	0.18240835	-2.09354246
H	-0.88129123	-1.42844348	-1.47716462
synQOOH24decom.inp			
C	-0.776046164	1.335950628	-1.106524354
C	0.097317583	-0.032960922	-0.014928949
C	-0.470656946	-1.311380736	-0.419474578
C	-1.694817914	-1.700830896	-0.059100467
H	2.617837791	1.459620632	-0.245340999
H	-2.327043069	-1.075379701	0.561580553
H	-2.101592017	-2.644480485	-0.401209458
O	-1.942516681	1.438505091	-0.669059764

H 0.140182399 -1.92011495 -1.079801323
O 1.431735161 0.029894512 -0.298986626
H -0.165380355 0.38316432 0.953595697
O 1.9950697 1.125733455 0.410041002
H -0.593710926 0.834912653 -2.075459293
H -0.026309372 2.104437983 -0.841958675
1032834335765453171472_r12_insertion_R_2_3_8.inp
C -0.46043979 -1.29106707 -0.69015346
C -0.0573634 0.01039852 0.00293599
C -0.63195002 -0.341322 1.38968906
C 0.15184787 -0.91344793 2.38581705
H 2.65793828 1.55110873 0.3589438
H 1.15628178 -1.26385278 2.17398902
H -0.24875072 -1.08630848 3.37876516
O -1.35242061 -1.77522399 0.39468366
H -1.55143884 0.1613224 1.67358993
O 1.38540638 0.13613136 -0.06283585
H -0.50445175 0.91524509 -0.41501234
O 1.69265987 1.51273163 0.54874645
H 0.38888054 -1.96355435 -0.84809867
H -1.02284477 -1.17004608 -1.62004171
1032794335955623191362_HO2_Elimination_from_PeroxyRadical_13_12.inp
C 0.1793455 -0.31565011 0.28266111
C 1.54070861 -0.31221315 -0.03852549
C 1.24870351 -0.97685149 -1.36769688
C 1.52967232 -0.31176171 -2.69557837
H 1.16629794 0.71770016 -2.69993305
H 1.03682998 -0.86899629 -3.49912904
H 2.60905269 -0.30214064 -2.89101555
O -0.23593986 -0.97706928 -0.97921612
H 1.57440541 -2.02595329 -1.35486742
O 1.5124784 2.04948093 -0.47414175
H 2.45369871 -0.11581408 0.5006959
O 0.22333819 2.21303763 -0.15541018
H -0.08622588 0.98520879 0.12908648
H -0.34954934 -0.56040796 1.1995939
1032834335765453171472_intra_R_migration_4_10.inp
C -0.86348206 0.09401911 -1.35628111
C -0.16986541 -0.02875247 0.00592062
C -0.23848543 -1.56116084 -0.15181194
C 1.20504201 -1.93879273 -0.25180102
H 2.36650893 1.90913391 0.01861123
H 1.63360065 -2.12976647 -1.22889864
H 1.74604884 -2.27228321 0.62795321
O -0.90754034 -1.41961065 -1.4854092
H -0.84179886 -2.15679531 0.53825002
O 1.29910723 0.13079516 -0.04078628
H -0.57792072 0.50286317 0.86305419
O 1.38373979 1.86149729 -0.03037772
H -0.26061604 0.54928679 -2.14183364
H -1.87638179 0.5014279 -1.34068898
1032834185635133391512.inp
C -0.69413406 -1.1620151 0.41789146
C -0.10526436 0.12384031 -0.03463231
C -0.82949983 0.75999911 1.20081909
C -0.0116943 1.39010262 2.29439649
H 0.85587305 0.7716631 2.53611452
H -0.62317152 1.52200374 3.19335819
H 0.34956012 2.37166707 1.96625727
O -1.30186802 -0.66739464 1.58668827
H -1.72040428 1.3316344 0.93317954
O 1.35352388 0.05467201 0.0678664
H -0.35109355 0.5290135 -1.01957796
O 1.86490673 1.4356434 -0.45217071
H -0.59760724 -2.21381617 0.20576839
H 2.79100863 1.17854387 -0.66378661
86262474678676 5682401.inp
C 0.05179 -0.940109 1.367522
C 0.397651 -0.745818 -0.114829
C -1.122997 -0.770769 -0.417035

C -1.00395 0.692732 -0.862811
H -1.564452 1.411773 -0.263607
H -1.134941 0.874647 -1.932765
O -1.430888 -0.917115 1.041397
H -1.666802 -1.492104 -1.022929
O 0.474148 0.695229 -0.515771
H 1.17688 -1.341753 -0.584747
H 0.336934 -0.118603 2.026349
H 0.323514 -1.908927 1.794853

S4.1.9 ROO3

1032834186085282960802_r12_insertion_R_3_13_14.inp
C -1.177506 1.127922 0.912733
C -0.490356 -0.135181 1.225075
C 0.069221 0.012592 -0.232103
H -1.109079 -1.005249 1.460704
H 0.340437 -0.048229 1.926878
C -0.57395 -0.859383 -1.294506
H -0.340599 -0.467437 -2.284612
H -1.65749 -0.874009 -1.154339
H -0.18261 -1.873614 -1.205808
O -0.655858 1.377516 -0.27046
H 2.483369 1.583164 0.306119
H -2.042814 1.664758 1.278517
O 1.356153 0.096125 -0.437721
O 2.012006 0.982407 0.89184
1032834186085282960802_intra_H_migration_13_4.inp
C 2.942502 1.059094 0.717283
C 1.873954 1.731376 -0.038834
C 1.16276 0.33766 -0.012668
H 1.348475 2.520462 0.5122
H 2.077972 2.059515 -1.059252
C 1.125447 -0.460657 -1.287655
H 0.927509 -1.503634 -1.047461
H 2.073241 -0.371525 -1.820502
H 0.31273 -0.084432 -1.91235
O 2.437165 -0.146287 0.815647
H -0.930212 -1.140672 1.578899
H 3.743496 1.391133 1.367781
O 0.092015 0.389165 0.730226
O -0.74836 -1.140078 0.633008
1032794336255753100462_intra_H_migration_14_7.inp
C 2.38083543 -1.7693924 -0.55416305
C 2.30539005 -0.9863013 0.76363349
C 1.53386732 0.04035758 -0.06895814
H 3.25901635 -0.62256055 1.14439783
H 1.71996744 -1.43024843 1.56741356
C 1.92572003 1.49931143 -0.20126948
H 0.65381432 1.75892574 -0.53923753
H 2.62645319 1.74827024 -0.9924789
H 2.06988431 2.0284245 0.73908227
O 1.75168884 -0.6854862 -1.26666149
H 1.77351099 -2.67680249 -0.59000858
H 3.38086808 -1.97039489 -0.94283209
O 0.18588136 0.0973084 0.34354587
O -0.37891446 1.12676926 -0.42257459
1701700000000000000002.inp
H 2.233259 0.713141 -0.388898
O 2.831771 0.768427 -1.151258
3304901700000000000002.inp
H 0.689742 1.477267 -0.597185
O 2.098773 2.172373 0.352199
O 1.454069 2.048226 -0.782823
1032553103162871500212.inp
C -2.112584 0.395928 -0.9936
C -0.634736 0.622103 -0.820257
C 0.033553 0.246919 0.268725

H -0.099431 1.114791 -1.620737
H 2.596028 0.381406 -0.949884
C -0.509784 -0.42222 1.49092
H -0.180706 -1.463289 1.533439
H -1.598432 -0.415597 1.486384
H -0.148496 0.088158 2.386641
O -2.590387 -0.868636 -0.834141
H -2.447527 0.762772 -1.980443
H -2.709016 1.025688 -0.299059
O 1.382783 0.417462 0.455878
O 1.995859 1.070007 -0.642979
1032794336255753100462_r22_cycloaddition_10_1.inp
C -0.11344275 -0.25215023 -1.55117662
C -0.002635 -0.26316098 -0.05135907
C 1.48737307 -0.53723345 -0.09576805
H -0.54832643 -1.09634403 0.40287484
H -0.27700555 0.66431868 0.44825824
C 2.41975635 -0.79132801 1.0332787
H 3.44703024 -0.68267016 0.68463569
H 2.2786646 -1.81844747 1.38718362
H 2.23235945 -0.10544198 1.86072363
O 1.75521477 -0.86146194 -1.27827015
H -0.15200113 0.68763338 -2.08249503
H -0.42210483 -1.14377313 -2.07947009
O 1.95732556 1.59663383 -0.14342312
O 1.57650492 1.9966012 -1.25761541
1032834186085282960802_r12_insertion_R_2_3_10.inp
C -1.163726 1.132891 0.923593
C -0.482206 -0.135309 1.227507
C 0.065935 0.011864 -0.234076
H -1.104654 -1.001793 1.466522
H 0.354954 -0.055362 1.922531
C -0.591826 -0.853857 -1.292655
H -0.364343 -0.461498 -2.283959
H -1.674209 -0.861795 -1.143325
H -0.206305 -1.87076 -1.209198
O -0.650643 1.381491 -0.263545
H 2.494441 1.566106 0.286482
H -2.022365 1.674586 1.297835
O 1.35158 0.087587 -0.450483
O 2.024295 0.967217 0.875091
712193173963061480122.inp
C -2.13252728 -1.28720807 -0.98827426
C -2.01769743 -0.67259255 0.41716813
C -0.62015387 -1.22854193 0.34617634
H -2.67694865 -1.07816168 1.18705751
H -2.07871586 0.42259008 0.43184749
C 0.66988818 -0.70805711 0.85025085
H 0.9278735 0.2610052 0.39313871
H 1.4799933 -1.40724203 0.62624234
H 0.62453588 -0.56983783 1.93309853
O -0.72563693 -1.6259342 -0.98116895
H -2.36133469 -0.60084714 -1.80503231
H -2.75154629 -2.18379875 -1.06171747
1032834225705693100612_intra_R_migration_2_10.inp
C -0.754421 0.108685 2.178464
C -0.752417 -0.777166 1.094488
C -0.014707 0.053513 0.097101
H -1.610704 -1.37446 0.814205
H 1.772265 1.810468 0.300284
C -0.304885 -0.1186 -1.374986
H 0.154056 0.695606 -1.935705
H -1.382075 -0.103587 -1.545407
H 0.111766 -1.06503 -1.724505
O -0.572429 1.235274 0.653046
H 0.18018 0.485908 2.572735
H -1.613668 0.191034 2.841407
O 1.377207 -0.022249 0.387926
O 2.026182 1.06464 -0.259619
702063003662761100081.inp

C 1.40114195 -1.47432141 0.1739857
C 0.5202444 -1.17625832 -1.05166518
C -0.52506342 -0.87139951 -0.00647395
H 0.2975842 -2.02931069 -1.692563
H 0.82786555 -0.32525018 -1.65928085
C -1.78819763 -0.47942515 0.03013815
H -2.30095485 -0.33848084 0.97291331
H -2.32009971 -0.29607465 -0.8934808
O 0.30167187 -1.1493204 1.05811637
H 2.24501256 -0.80105496 0.33449866
H 1.71231509 -2.51267288 0.3010916
1032794336255753100462_r12_insertion_R_6_3_13.inp
C 0.07928553 -0.82102991 -1.06716254
C 0.06047732 -0.00253634 0.23479281
C 1.54785344 0.05022649 0.00782477
H -0.28950973 -0.51387593 1.13362394
H -0.45447058 0.96224685 0.14764478
C 2.54924387 1.1074249 0.26803708
H 2.55522746 1.36824078 1.32905256
H 2.33936493 2.02299424 -0.30736943
H 3.54994298 0.76357008 -0.00725261
O 1.48980172 -0.5488451 -1.2451764
H -0.49967042 -0.42574946 -1.90305507
H -0.10129162 -1.89180311 -0.95227887
O -0.24273811 2.09787141 -2.86982069
O 0.63067321 2.75417207 -2.38406933
1032794336255753100462.inp
C 0.134127 -0.503314 -1.430435
C 0.002727 0.011108 0.008484
C 1.535279 0.022559 -0.022113
H -0.413423 -0.696945 0.723944
H -0.457714 0.989871 0.122085
C 2.348622 -0.729135 0.995356
H 3.397353 -0.741994 0.692346
H 1.986181 -1.755901 1.057026
H 2.264226 -0.256965 1.976147
O 1.576067 -0.518655 -1.314651
H -0.190071 0.191576 -2.206629
H -0.252071 -1.505999 -1.622986
O 2.113281 1.373543 -0.015342
O 1.515573 2.182867 -0.837651
1032513213602331230512.inp
C 0.02479276 0.06709733 0.27353338
C 1.50579628 0.0511163 -0.04746933
C 2.04804061 1.44013245 -0.32597321
H 1.69976465 -0.57604834 -0.92546146
H 2.06536311 -0.39607428 0.78055423
C 3.5414543 1.54791479 -0.51105049
H 3.80112607 2.53911347 -0.87955069
H 3.9064935 0.77999281 -1.19809954
H 4.03389304 1.38049246 0.45214755
O 1.31835328 2.40070666 -0.38566958
H -0.57165022 0.43830827 -0.55865971
H -0.19318884 0.65176952 1.16783681
O 0.00564218 -1.78922585 1.59580397
O -0.45807313 -1.2800683 0.49098073
320320000000000000003.inp
O 2.52399412 2.62401575 0.73781138
O 1.60679888 2.11200125 0.16660162
1032834225705693100612_intra_R_migration_2_13.inp
C -0.046138 -0.316196 1.811574
C -0.059896 -1.135086 0.560794
C 0.036391 0.062501 -0.139919
H 0.002499 -2.180379 0.31308
H 2.324026 1.381134 0.808128
C -0.333484 0.548644 -1.49009
H 0.043723 -0.142863 -2.244332
H 0.101499 1.533083 -1.664028
H -1.421184 0.613981 -1.575011
O -0.063121 0.88288 0.961984

H 0.872649 -0.346491 2.403893
H -0.935731 -0.346382 2.44498
O 1.961523 0.057558 -0.443841
O 2.588509 0.456881 0.706814
702062893452741310061.inp
C -0.753908 0.316377 1.388099
C -0.87833 -1.169015 1.170692
C -0.65602 -0.924741 -0.125318
H -1.062988 -2.032346 1.785924
C -0.53439 -1.63106 -1.417882
H -0.669546 -2.703571 -1.274021
H 0.450492 -1.451848 -1.85743
H -1.288243 -1.268104 -2.121639
O -0.521626 0.449878 -0.051373
H 0.114914 0.673503 1.944617
H -1.655231 0.86105 1.676301
1032834225705693100612_intra_H_migration_2_5.inp
C -0.811841 -0.154896 -1.676652
C -0.53861 1.079808 -0.854083
C 0.033983 0.119152 0.208457
H -1.2253 1.904657 -0.686162
H 0.725768 1.424702 -1.141764
C -0.601966 -0.097728 1.554668
H -0.129475 -0.953039 2.042206
H -1.663055 -0.31585 1.425202
H -0.483064 0.786631 2.181736
O -0.172023 -0.978953 -0.686654
H -0.311288 -0.219069 -2.647492
H -1.872253 -0.406527 -1.790213
O 1.373812 0.465928 0.408785
O 1.815782 1.007703 -0.800715
1032794336255753100462_intra_H_migration_14_4.inp
C 3.09940584 0.93400179 0.65480724
C 2.09058441 1.48456072 -0.32262677
C 1.57957341 0.03463227 -0.45208322
H 0.99525401 1.74537871 0.4073839
H 2.29358168 2.18008793 -1.13204552
C 1.70162101 -0.77478034 -1.71451748
H 1.44965102 -1.81552848 -1.50037071
H 2.73073559 -0.73493752 -2.07450979
H 1.0291606 -0.3908905 -2.48253815
O 2.50586588 -0.37246338 0.56046081
H 3.06067357 1.32803794 1.67482348
H 4.13505622 0.93608474 0.29686353
O 0.23881791 0.02544505 -0.0548003
O 0.10610601 1.06013209 0.87447859
1032834186085282960802.inp
C -1.000392 1.252529 1.048188
C -0.490162 -0.140882 1.251712
C 0.037291 0.034171 -0.19016
H -1.234578 -0.934505 1.360482
H 0.292847 -0.256052 2.001921
C -0.531387 -0.849035 -1.272145
H -0.233328 -0.475419 -2.252972
H -1.619905 -0.843273 -1.204498
H -0.167812 -1.87053 -1.14789
O -0.513306 1.378086 -0.244329
H 1.963127 1.70717 0.267041
H -1.942333 1.726967 1.296884
O 1.413229 0.043665 -0.399418
O 2.057632 0.808484 0.607505
1032834186085282960802_r12_insertion_R_6_3_10.inp
C -1.178901 1.127815 0.911211
C -0.495838 -0.137675 1.222821
C 0.070266 0.013098 -0.231531
H -1.117366 -1.007086 1.453414
H 0.332292 -0.05442 1.928224
C -0.570295 -0.854266 -1.29927
H -0.331849 -0.459882 -2.287193
H -1.654453 -0.86688 -1.163767

H -0.181595 -1.869631 -1.211952
O -0.65155 1.379739 -0.268968
H 2.486114 1.575989 0.322049
H -2.044601 1.665491 1.274822
O 1.358265 0.094283 -0.431354
O 2.010431 0.974799 0.903817
862624746847083821241.inp
C -1.414633 0.614289 1.318913
C -0.393142 -0.511871 1.240775
C -0.452409 -0.32001 -0.202315
H -0.225928 -1.364015 1.884292
C -0.360947 -1.115541 -1.450681
H -0.010059 -0.473383 -2.262509
H -1.34011 -1.515147 -1.716746
H 0.347359 -1.933058 -1.317211
O -1.380017 0.743193 -0.127798
H -1.100657 1.528513 1.825205
H -2.405851 0.292332 1.644582
O 0.715785 0.033545 0.505842
1032794336255753100462_r12_insertion_R_7_6_3.inp
C -0.30362032 -0.52598221 -1.29347412
C -0.11913933 -0.10842829 0.17628251
C 1.28178077 -0.59404314 -0.06446514
H -0.66758336 -0.73005268 0.88717916
H -0.23145422 0.94529551 0.41771809
C 2.45879357 -0.74810861 0.65588345
H 3.04444559 0.37623618 0.31309731
H 3.12794859 -1.52748623 0.30007053
H 2.35486389 -0.67213379 1.7346399
O 1.03808368 -1.10188056 -1.28100295
H -0.34647619 0.29678172 -2.00548748
H -1.04858178 -1.28925122 -1.51356827
O 3.10793399 1.53665837 -0.26866857
O 1.94171844 1.53557062 -0.7808273
1032794336255753100462_HO2_Elimination_from_PeroxyRadical_4_14.inp
C -0.58728611 -0.66276148 -1.56194388
C 0.30785577 -0.28012394 -0.38579968
C 0.8945558 0.61226148 -1.29672841
H -0.37802457 0.61910014 0.36606589
H 0.82481773 -0.94028801 0.29991587
C 2.13629235 1.3920157 -1.4501903
H 2.54698706 1.63983325 -0.47237955
H 1.93413815 2.31171992 -2.00091586
H 2.86846125 0.80109984 -2.01064903
O 0.0700312 0.39802545 -2.33506564
H -1.65712993 -0.46915209 -1.48220068
H -0.38770936 -1.62605559 -2.03055387
O -0.17741575 2.43245492 -0.21291871
O -0.79648605 1.74554589 0.66974651
1032553363992780670042.inp
C -1.252109 -1.2853 1.515456
C -0.11322 -0.775933 1.068888
C 0.03166 -0.087913 -0.297964
H 0.802651 -0.788679 1.648798
H 1.341496 2.025657 0.295279
C -0.257535 -1.003779 -1.487561
H -0.162161 -0.440626 -2.417157
H -1.271411 -1.392045 -1.398059
H 0.452533 -1.831263 -1.489994
O -0.819177 0.949875 -0.220786
H -1.292329 -1.795666 2.470954
H -2.175273 -1.208833 0.951062
O 1.386015 0.324354 -0.498209
O 1.768174 1.197483 0.549515
1032513213542841290002.inp
C -0.57212543 1.1933834 0.22070863
C 0.05139865 0.04071237 -0.60515902
C 1.50490807 -0.0069095 -0.34414276
H -0.09561995 0.27217926 -1.6657008
H -0.41465455 -0.91203844 -0.35081858

C 2.17056535 -1.09373826 0.41074166
H 1.96796204 -2.05649874 -0.06700963
H 1.7351031 -1.12714858 1.41484207
H 3.24467785 -0.92738621 0.47705604
O -0.43527201 1.06174624 1.5458939
H -1.61064216 1.39245583 -0.08856962
H 0.00260447 2.1106344 -0.0728821
O 2.2382657 0.94520185 -0.7191297
O 1.6429082 1.98455429 -1.38715943
1032794336255753100462_r12_insertion_R_1_2_3.inp
C 0.57639296 -0.35924881 -1.37139893
C 0.06587563 0.10726482 -0.03979822
C 2.2871809 -0.05955939 0.02705382
H -0.39241143 -0.60467709 0.6380218
H -0.11148681 1.16361975 0.12215554
C 2.91630672 -0.82761446 1.13488894
H 3.83789185 -1.2864022 0.76111661
H 2.24972819 -1.62539054 1.46181644
H 3.15458777 -0.1694474 1.96892141
O 1.95568583 -0.71450791 -1.09463796
H 0.55494006 0.42436521 -2.12615589
H 0.09492093 -1.26821389 -1.73489794
O 2.55900561 1.20620981 0.01037579
O 2.21307255 1.90699566 -1.08164849
1032553033062561730462.inp
C 3.59016848 -1.13355161 -0.08581118
C 4.16201228 -0.70285404 -1.38425928
C 1.56617027 0.10452236 0.26028007
H 4.14018663 -1.37248243 -2.23573471
H 4.46995549 0.32379208 -1.53480896
C 0.08070274 0.0716953 0.07484985
H -0.34347606 1.02511775 0.38491185
H -0.34324691 -0.73538391 0.67395472
H -0.15044664 -0.11493971 -0.97477949
O 2.13587168 -1.06688348 -0.09326002
H 3.96472445 -0.53504205 0.74554487
H 3.79456327 -2.18575485 0.11377577
O 2.23676393 1.28149523 -0.02057543
O 2.06614159 0.77665739 1.36508241
1032794336255753100462_intra_R_migration_14_10.inp
C -0.17989474 0.03083362 -1.40909686
C -0.02020076 -0.23137319 0.08872898
C 1.48102614 -0.15272656 0.09575171
H -0.33475714 -1.23769421 0.36661559
H -0.46622044 0.51827779 0.74476245
C 2.39721752 -1.26294211 0.44473879
H 2.09735324 -2.16063613 -0.09461351
H 2.32008227 -1.45830771 1.52085323
H 3.42706397 -1.00430482 0.20090979
O 0.94505732 -0.57564819 -1.93648816
H -0.1674004 1.11927798 -1.59939231
H -1.08523043 -0.37441848 -1.88371499
O 2.04138565 1.00927384 0.01850294
O 1.23024065 2.07564783 -0.04819806
862062612811410570001.inp
C 0.168284 0.104969 -0.341449
C 1.565077 -0.132008 0.198638
C 2.224922 1.19269 0.569989
H 2.140543 -0.717547 -0.51907
H 1.456995 -0.702712 1.128548
C 3.519713 1.523165 -0.122409
H 3.891813 2.490113 0.212268
H 3.362927 1.534009 -1.205724
H 4.259864 0.743973 0.084307
O -0.248209 -0.404271 -1.344806
H -0.45186 0.795716 0.261013
O 1.699157 1.927671 1.369161
1032834225705693100612_Cyclic_Ether_Formation_2_13.inp
C -0.009197 0.204334 1.456108
C -0.01968 0.029798 -0.02491

C 1.081205 1.022752 -0.087765
H -0.529304 -0.5834 -0.750997
H 3.706734 0.108936 0.590836
C 1.263566 2.254914 -0.912624
H 2.226136 2.700554 -0.657791
H 0.463961 2.96696 -0.706739
H 1.268763 1.992218 -1.970881
O 0.971847 1.254716 1.328689
H 0.34468 -0.644079 2.051021
H -0.949406 0.583037 1.873481
O 1.882224 -0.058895 -0.355019
O 3.561566 0.430223 -0.307285
1032834225705693100612.inp
C -1.216332 1.141518 1.172818
C -0.506281 -0.16523 1.225967
C -0.044918 0.083346 -0.180009
H -0.131229 -0.790677 2.021783
H 1.702084 1.97607 0.251812
C -0.48679 -0.844841 -1.290653
H -0.255069 -0.393466 -2.257357
H -1.56315 -1.002472 -1.219058
H 0.025538 -1.804622 -1.206952
O -0.745381 1.342542 -0.182002
H -0.846455 1.918962 1.854081
H -2.311599 1.113579 1.215613
O 1.334345 0.258791 -0.390394
O 1.865814 1.100458 0.623262
1032834335915713060802.inp
C -0.59818582 1.94906038 -0.48636098
C -0.74203703 1.23580592 0.85959549
C 0.08472152 0.10999064 0.20666041
H -1.75273829 0.94358301 1.14059453
H -0.23454281 1.71155476 1.69528163
C -0.53237813 -1.21875113 0.0229041
H 2.39676189 1.23523901 0.03597864
H 0.09082837 -2.09661587 -0.08557754
H -1.59563955 -1.2797728 -0.16692434
O 0.19035165 0.8638096 -1.02184654
H -0.03001013 2.88237632 -0.47509364
H -1.52189935 2.08104189 -1.0542691
O 1.35794548 -0.15093872 0.73986581
O 2.05696375 1.06940978 0.92449276
1032794336255753100462_intra_H_migration_10_12.inp
C 0.32724 -0.049913 -1.566338
C 0.034194 0.106459 -0.080792
C 1.51622 -0.317372 -0.008235
H -0.621743 -0.674857 0.306449
H -0.225985 1.089013 0.304539
C 2.089428 -1.224788 1.034945
H 3.120805 -1.473532 0.780246
H 1.500681 -2.140734 1.088244
H 2.07078 -0.72446 2.004399
O 1.371131 -0.934347 -1.323571
H -0.342482 -0.28811 -2.389649
H 0.99564 1.071265 -1.688608
O 2.393481 0.768172 -0.178112
O 1.817309 1.75782 -0.985287
1032834225705693100612_r12_insertion_R_1_10_3.inp
C -1.534318 -0.295616 -1.416487
C -1.051587 0.899464 -0.660071
C -0.20155 0.287656 0.293811
H -0.700575 1.776165 -1.187768
H 1.868276 0.998192 -1.020056
C -0.64149 -0.644368 1.381257
H 0.103368 -1.424675 1.532909
H -1.596492 -1.097391 1.126064
H -0.755151 -0.071174 2.305705
O -0.287182 -0.946979 -1.236516
H -1.768876 -0.083212 -2.464836
H -2.369424 -0.849206 -0.966825

O 1.008533 0.764843 0.619724
O 1.544298 1.632486 -0.363843

S4.1.10 ROO4

702023143922960840001.inp
C -2.165589 0.623474 -1.014938
C -2.089595 -0.765802 -0.290421
C -0.621923 -0.459096 0.038815
H -2.223887 -1.647723 -0.927323
H -2.725515 -0.881308 0.594861
C -0.663433 0.906005 -0.662103
H 0.022996 0.986186 -1.512894
H -0.478712 1.752482 0.009327
O 0.255804 -1.074654 0.637943
H -2.870766 1.325404 -0.563543
H -2.368817 0.558618 -2.086493
1032553213412331340512_intra_H_migration_1_7.inp
C -0.823047 0.9417 -0.093856
C 0.250993 -0.079369 0.2377
C 1.495567 0.331536 -0.572039
H -0.015586 -1.107375 -0.031106
H 0.507818 -0.078554 1.305684
C 1.384547 1.793941 -0.955233
H 0.070708 1.795146 -0.847259
O 1.847931 2.152241 -2.211431
H 1.67131 2.497183 -0.162498
O 2.412627 -0.413747 -0.927677
H -1.564521 0.663798 -0.842701
H -1.205449 1.575789 0.704254
H 3.747573 1.534943 -2.376692
O 3.425236 2.42265 -2.079635
1032834075014433582002_Intra_R_Add_ExoTetCyclic_F_3_1_11.inp
C -1.46805 -3.05128 1.21214
C -0.584657 -1.909837 1.729776
C -0.89964 -1.024719 0.554874
H 0.479096 -2.152491 1.821878
H -0.924809 -1.525546 2.701971
C -0.264309 0.209131 0.066527
H -0.67884 0.512608 -0.898774
O 1.173968 -0.122097 -0.081045
H -0.335416 1.036988 0.783462
O -1.830377 -1.688797 -0.120941
H -2.456997 -3.208175 1.626228
H -1.011077 -3.894525 0.703427
H 2.669691 0.932212 -0.807896
O 1.891927 1.253107 -0.296872
1032834224994513281772_Cyclic_Ether_Formation_2_8.inp
C 0.105479 -1.334678 1.096262
C -0.198614 -0.229447 0.126515
C 1.277325 -0.100644 -0.220136
H -0.036446 3.07914 2.363093
H -1.10168 0.03576 -0.405581
C 1.641556 1.26679 0.311888
H 2.493752 1.27134 0.993236
O 0.378213 1.52277 1.065614
H 1.75547 2.028681 -0.464806
O 1.577256 -1.212689 0.758992
H -0.274933 -2.330374 0.835979
H -0.077294 -1.123379 2.154133
H 1.649897 -0.352201 -1.216797
O 0.76602 3.033929 1.793251
1032553212962181640662_intra_R_migration_3_8.inp
C 1.119064 -0.013069 3.253571
C -0.14057 -0.296023 2.454897
C -0.277257 0.516537 1.202169
H -0.989605 -0.131601 3.140253
H -0.14061 -1.376472 2.236738

C	-0.002817	0.016301	-0.067966
H	-0.058856	-1.05428	-0.253952
O	1.884949	-0.093816	-0.144155
H	-0.162627	0.647972	-0.934924
O	2.054141	0.712968	2.885258
H	1.177401	-0.5112	4.236635
H	2.602672	1.212256	1.090763
H	-0.410154	1.589067	1.327996
O	2.511845	1.208588	0.099936
1032553252772121680812.inp			
C	-2.166408	2.897289	1.685666
C	-1.571181	1.963617	0.67211
C	-0.649877	1.034842	0.964399
H	2.75041	1.305231	-0.779476
H	-1.932182	2.084839	-0.348783
C	-0.062392	0.097139	-0.044754
H	-0.258707	-0.952849	0.210591
O	1.405447	0.107508	-0.048181
H	-0.42274	0.298151	-1.058932
O	-3.589804	2.969827	1.65369
H	-1.857905	3.944977	1.482168
H	-1.827613	2.682624	2.711308
H	-0.284775	0.919616	1.985239
O	1.79445	1.478957	-0.621025
1032834075014433582002_intra_H_migration_14_7.inp			
C	4.018703	2.548198	1.860811
C	3.418303	1.117136	1.75343
C	2.822653	1.601303	0.472107
H	2.650264	0.864317	2.49423
H	4.138329	0.294658	1.682836
C	1.935608	1.093962	-0.567131
H	1.418577	1.931866	-1.056476
O	1.051563	0.086639	-0.066497
H	2.505442	0.54097	-1.339096
O	3.326858	2.891112	0.529158
H	5.096092	2.652909	1.743179
H	3.621003	3.194081	2.641129
H	-0.415187	0.432647	1.358906
O	0.448151	0.894695	1.438414
701782141760940380001.inp			
C	-0.04832	-0.075275	0.2152
C	1.430613	0.054492	-0.097025
C	1.891875	1.495205	-0.039167
H	1.593294	-0.341837	-1.111069
H	1.986095	-0.574358	0.608616
C	2.264472	2.221831	-1.102504
H	2.275193	1.806634	-2.108194
H	2.581463	3.255702	-1.002953
O	-0.512922	-0.808502	1.096834
H	-0.722148	0.541965	-0.40584
H	1.900889	1.947574	0.952584
170170000000000000002.inp			
H	1.807023	-0.303376	-1.39183
O	2.580372	-0.733279	-0.935768
1032834224994513281772_r12_insertion_R_1_10_3.inp			
C	0.105241	0.631935	-3.510947
C	0.069393	-0.476874	-2.51477
C	-0.5056	0.185777	-1.388096
H	1.668692	1.628285	-1.057838
H	0.889698	-1.178103	-2.400681
C	0.006304	-0.021277	0.011433
H	-0.367854	0.740216	0.700849
O	1.471412	-0.01628	0.032085
H	-0.260431	-1.018293	0.387833
O	0.310309	1.627043	-2.365668
H	-0.832782	0.876751	-4.018083
H	0.940918	0.629984	-4.21327
H	-1.492885	0.634251	-1.48328
O	1.905876	1.435926	-0.102338
1032794335394813321702_intra_H_migration_14_13.inp			

C	3.609997	2.386829	-1.05708
C	2.251643	1.972706	-1.678749
C	1.997224	1.244636	-0.355751
H	2.337754	1.303616	-2.541934
H	1.56046	2.781409	-1.924029
C	1.395972	-0.12971	-0.127831
H	1.537942	-0.839801	-0.948425
O	-0.053313	0.131835	-0.057898
H	1.766083	-0.539136	0.818037
O	3.318592	1.494294	0.140781
H	3.701839	3.423337	-0.731857
H	4.517003	2.043928	-1.554938
H	0.983404	1.786178	0.387697
O	-0.115796	1.367919	0.816614
1032553213412331340512_r12_insertion_R_4_2_1.inp			
C	-1.873179	2.283029	2.504742
C	-0.79974	1.778982	1.846478
C	-0.870878	0.465858	1.156427
H	0.417773	0.907226	3.296159
H	0.086176	2.377815	1.66799
C	0.333854	-0.021602	0.360746
H	0.900388	-0.742486	0.964516
O	1.322031	0.998615	0.060734
H	-0.00355	-0.505691	-0.560146
O	-1.864885	-0.284961	1.220473
H	-2.784525	1.700766	2.606724
H	-1.847372	3.268027	2.959026
H	1.466771	2.254773	-1.426377
O	0.658892	1.877983	-1.008297
3304901700000000000002.inp			
O	2.06919	-0.525637	-2.101625
H	3.323664	0.215793	-0.838037
O	2.357212	-0.005976	-0.838305
1032834075014433582002.inp			
C	-1.064699	-1.054275	3.258327
C	-1.4044	-1.339045	1.767761
C	-0.736078	-0.012844	1.494747
H	-2.473347	-1.395968	1.53317
H	-0.904573	-2.217128	1.342998
C	-0.131293	0.682784	0.367227
H	-0.775321	0.676982	-0.517882
O	1.114171	-0.07356	0.00873
H	0.153274	1.706995	0.624898
O	-0.376394	0.239789	2.847147
H	-0.339089	-1.716492	3.729947
H	-1.902019	-0.835095	3.920469
H	2.631633	0.453825	-1.116811
O	1.675372	0.645804	-1.24827
1032553213412331340512_r12_insertion_R_3_2_4.inp			
C	1.577275	1.828938	-2.74575
C	0.783541	2.602692	-1.942438
C	-0.564166	1.10544	-0.96828
H	-0.087706	3.109965	-2.34627
H	1.143303	2.962166	-0.984127
C	0.200302	0.145137	-0.07203
H	-0.480191	-0.269151	0.681476
O	1.348863	0.739745	0.569953
H	0.634573	-0.665416	-0.669639
O	-1.611907	0.848168	-1.533575
H	1.276929	1.55958	-3.754635
H	2.516238	1.416877	-2.386449
H	1.533696	1.844007	2.165443
O	0.759999	1.750243	1.563182
862383584133292070551.inp			
C	-1.210135	-0.618898	-1.49036
C	-0.799636	-1.229234	-0.363187
C	0.004328	0.009814	0.011338
H	1.771745	1.815018	-0.78703
H	-0.967866	-2.184084	0.106462
C	1.513297	-0.03355	-0.033598

H	1.910693	-0.532315	0.854156
O	2.073334	1.311953	-0.002374
H	1.842352	-0.575874	-0.928515
O	-0.522483	0.62889	-1.317347
H	-1.835099	-0.79381	-2.352105
H	-0.372605	0.619586	0.834724

103255321341233134 0512.inp

C	-1.25024	2.402718	2.994104
C	-0.242562	1.800767	2.076585
C	-0.759279	0.642939	1.252082
H	0.656645	1.454443	2.618282
H	0.165223	2.543246	1.36654
C	0.220943	-0.126232	0.372529
H	0.451734	-1.089808	0.84601
O	1.529729	0.489249	0.232074
H	-0.219858	-0.30778	-0.612303
O	-1.940323	0.258032	1.272082
H	-2.268086	2.031522	3.013917
H	-0.97131	3.233155	3.633597
H	2.207427	1.758134	-1.088411
O	1.306139	1.689097	-0.698191

10328340750144335820 02_Intra_R_Add_ExoTetCyclic_F_3_8_14.inp

C	-1.959006	0.31924	-0.47161
C	-1.098978	0.50159	0.812427
C	0.03258	-0.027129	-0.03021
H	-1.385886	-0.140649	1.652834
H	-0.975753	1.526574	1.16717
C	1.481368	-0.160359	0.055546
H	1.863136	-0.460552	1.036724
O	1.624437	1.265156	-0.171459
H	1.920622	-0.766186	-0.741874
O	-0.724424	-0.267671	-1.165798
H	-2.259022	1.229212	-0.988794
H	-2.752285	-0.425588	-0.448589
H	3.367483	2.465221	-0.352433
O	3.430732	1.576434	0.069948

103255321341233134 0512_r12_insertion_R_3_6_7.inp

C	-1.038913	2.426151	2.86407
C	-0.159101	1.685102	2.023494
C	-0.762246	0.514299	1.249669
H	0.814479	1.431381	2.465007
H	0.262516	2.307158	1.082589
C	0.170757	-0.282241	0.336018
H	0.31403	-1.279986	0.780118
O	1.482262	0.279218	0.197111
H	-0.322374	-0.403838	-0.636758
O	-1.967252	0.230357	1.307916
H	-2.111408	2.407738	2.692924
H	-0.658876	3.046691	3.669496
H	1.878197	2.400289	-0.456562
O	0.984113	2.017155	-0.296197

10328341852243831816 62_Intra_R_Add_ExoTetCyclic_F_1_3_13.inp

C	0.687524	1.828283	-2.727006
C	-0.090675	0.555815	-2.601363
C	-0.541466	0.876694	-1.154335
H	0.521617	-0.356254	-2.627949
H	-0.907698	0.436584	-3.326003
C	-0.064021	0.084764	0.018378
H	-0.343506	0.545677	0.970436
O	1.3905	-0.122307	-0.000478
H	-0.461751	-0.942015	-0.009308
O	0.730176	2.313408	-1.477323
H	0.976806	2.419251	-3.587421
H	1.889589	1.773704	-0.163042
H	-1.498468	1.383692	-1.040328
O	2.02106	1.12978	0.588882

10327943353948133217 02_HO2_Elimination_from_PeroxyRadical_13_14.inp

C	-1.404524	-1.31268	0.890386
C	-0.544069	-0.324241	1.719391
C	0.332297	-0.19533	0.450903

H	-0.030343	-0.767413	2.575491
H	-1.054852	0.588383	2.034952
C	1.704769	-0.107243	0.27662
H	2.330403	0.39368	1.006509
O	1.637519	1.891358	-0.883323
H	2.193417	-0.589194	-0.562457
O	-0.482017	-1.18069	-0.297462
H	-2.409817	-0.975135	0.633149
H	-1.417549	-2.34672	1.240175
H	0.045206	0.952286	-0.098176
O	0.322334	2.07898	-0.776307

862062612531690570001.inp

C	-0.61242	-1.158694	-0.88173
C	-0.00007	0.105053	-0.27074
C	1.504415	0.128972	-0.302544
H	-0.315094	0.237501	0.775715
H	-0.360266	1.011798	-0.780565
C	2.224723	1.337538	0.278153
H	-0.32359	-1.271322	-1.931826
O	1.632829	2.301653	0.775114
H	3.323218	1.284538	0.220012
O	2.216842	-0.775422	-0.773461
H	-0.277722	-2.056989	-0.352716
H	-1.705078	-1.113276	-0.827397

103283407501443358 2002_intra_H_migration_14_4.inp

C	2.77934	-0.684309	-1.064196
C	2.294899	-0.033895	0.261598
C	0.921823	-0.349995	-0.232813
H	2.435842	1.055353	0.329475
H	2.623867	-0.512779	1.188972
C	-0.45022	-0.025066	0.136302
H	-1.143437	-0.824347	-0.160985
O	-0.895476	1.152131	-0.60714
H	-0.516256	0.155979	1.214962
O	1.304177	-0.935106	-1.41951
H	3.302347	-1.637207	-1.003775
H	3.209426	-0.015692	-1.806922
H	0.104359	2.989213	-0.635107
O	0.597249	2.203242	-0.313112

103255321341233134 0512_intra_H_migration_8_11.inp

C	2.423519	0.797798	0.151853
C	1.561091	-0.381367	-0.024304
C	0.066517	-0.04719	-0.206037
H	1.614461	-1.06422	0.844086
H	1.854816	-0.987104	-0.898947
C	-0.409629	1.374721	0.102646
H	-1.282899	1.59736	-0.511148
O	0.583481	2.397433	-0.178931
H	-0.701507	1.422575	1.161984
O	-0.744679	-0.922056	-0.525153
H	1.57239	1.988445	0.052592
H	3.481944	0.920887	0.350579
H	-0.045703	4.578102	-0.234047
O	-0.667584	4.16983	0.41841

702063003662761100081.inp

C	0.736972	1.525339	-0.008815
C	1.644743	0.670131	-0.934004
C	0.423909	-0.216129	-1.12665
H	2.49255	0.189159	-0.438468
H	1.996697	1.17613	-1.8372
C	0.080367	-1.32055	-1.783866
H	-0.922875	-1.725555	-1.714062
H	0.800265	-1.840566	-2.405655
O	-0.411818	0.575895	-0.271938
H	0.487045	2.52685	-0.360945
H	0.987841	1.529082	1.052568

103255321296218164 0662_intra_H_migration_3_7.inp

C	0.070709	-0.030741	-0.099198
C	1.52535	0.15887	0.207555
C	2.042596	1.513492	0.095878

H	2.170009	-0.565044	-0.320745
H	1.805827	-0.144101	1.270684
C	3.457412	1.756182	0.235233
H	3.408207	1.80985	-0.947821
O	4.28908	0.782869	0.659273
H	3.725932	2.793158	0.496448
O	-0.707578	0.897678	-0.361712
H	-0.296961	-1.068557	-0.064773
H	4.011009	-0.160492	2.620975
H	1.370827	2.343169	-0.125451
O	3.110286	0.198467	2.455237
1032553363432331190492.inp			
C	-1.611673	1.308623	3.317743
C	-0.621283	1.178488	2.426204
C	-0.797243	1.275813	0.928109
H	2.842852	1.032761	-0.136128
H	0.400705	0.987014	2.748364
C	-0.14086	0.10907	0.142221
H	-0.527181	-0.857257	0.482592
O	1.282394	-0.000608	0.411998
H	-0.311268	0.236122	-0.929808
O	-2.128033	1.435289	0.486813
H	-2.63362	1.503762	3.006431
H	-1.423948	1.225288	4.383851
H	-0.289602	2.195113	0.559028
O	1.889497	1.265885	-0.208637
862343624284022780761.inp			
C	-0.175875	1.324693	-0.072065
C	-1.458012	0.526105	0.193204
C	-0.966941	-0.916158	0.274853
H	-2.162414	0.606489	-0.645739
H	-1.989868	0.806374	1.107067
C	0.464276	-0.958669	-0.24452
H	1.16013	-1.195845	0.574503
O	0.719336	0.376009	-0.780923
H	0.592524	-1.687257	-1.048061
O	-1.606656	-1.887459	0.690932
H	0.306092	1.648278	0.860201
H	-0.298144	2.179604	-0.73604
862102742661560380021.inp			
C	0.246827	2.069783	-1.138648
C	-0.704459	1.236776	-0.258876
C	-0.104874	-0.13179	-0.001291
H	-1.681037	1.119861	-0.745752
H	-0.881203	1.730259	0.710354
C	1.42324	-0.049232	0.06824
H	1.782697	-0.136127	1.098506
O	1.739347	1.276975	-0.506117
H	1.899178	-0.798339	-0.565627
O	-0.703347	-1.20704	0.127849
H	2.175643	1.921456	0.077408
H	0.325527	1.571105	-2.127345
1032834185224383181662.inp			
C	-0.702635	-0.691	3.091835
C	0.468808	0.197196	2.739066
C	-0.331547	0.533007	1.447178
H	1.431461	-0.298224	2.571161
H	0.63608	1.059362	3.39714
C	0.15515	-0.058603	0.140167
H	0.48831	-1.093594	0.275749
O	1.360517	0.612043	-0.338808
H	-0.620326	-0.016933	-0.630692
O	-1.501287	-0.294937	1.963595
H	-1.195993	-0.961623	4.016528
H	1.653052	2.263563	-1.33227
H	-0.642341	1.571303	1.333933
O	0.888197	2.010762	-0.766965

S4.2 Total Energies

S4.2.1 R1

geometry name	L3 energy	L2 ZPE	total
MOR1.inp	-231.428	0.101953	-231.3261728
MOR1RO.inp	-231.381	0.097191	-231.2834817
MOR1rop	-231.452	0.097722	-231.3543152
711912311911110570002_r12_insertion_R_1_2_4.out	-231.41	0.095995	-231.3144402
290410170000000000002.out	-113.711	0.01321	-231.3278115
421261230750120000001.out	-117.709	0.07991	
711912311911110570002_intra_H_migration_3_4.out	-231.402	0.095739	-231.3058873
711912311781240570002.out	-231.467	0.100307	-231.3662413
711952161721200720062_intra_H_migration_3_11.out	-231.409	0.096045	-231.3133608
711952161721200720062.out	-231.461	0.099919	-231.3614096
711952311741200570042_intra_H_migration_5_11	-231.393	0.095744	-231.2976541
711952311741200570042.inp	-231.451	0.100483	-231.3507625

S4.2.2 R2

geometry name	L3 energy	L2 ZPE	total
711912462231090120002_intra_H_migration_2_10	-231.431	0.094813	-231.3358179
711912462231090120002_intra_H_migration_9_6	-231.415	0.095272	-231.3200704
711912462231090120002_intra_H_migration_9_11	-231.42	0.09501	-231.3247166
711912462231090120002_R_Addition_MultipleBond_9_3_2	-231.426	0.095151	-231.3306797
711912462231090120002_R_Addition_MultipleBond_9_3_5	-231.438	0.095679	-231.3418986
711912462231090120002_R_Addition_MultipleBond_9_3_10	-231.426	0.09161	-231.3346075
711912351721200630002_intra_H_migration_9_11	-231.421	0.095211	-231.3253702
711912351721200630002_intra_H_migration_9_10	-231.422	0.095272	-231.3269837
711912351721200630002_intra_H_migration_9_6	-231.44	0.094684	-231.345518
711912351721200630002_r12_insertion_R_2_1_11	-231.42	0.095244	-231.3249984
711912351721200630002_R_Addition_MultipleBond_9_1_11	-231.425	0.091309	-231.3332313
711912351721200630002_R_Addition_MultipleBond_3_5_6	-231.283	0.086773	-231.1962651
711912311911110570002_intra_H_migration_1_4	-231.43	0.094994	-231.3346457
711912311911110570002_intra_H_migration_9_6	-231.441	0.095992	-231.3450674
711912311911110570002_intra_H_migration_3_4	-231.44	0.095739	-231.3442175
711912311911110570002_intra_H_migration_3_6	-231.421	0.094699	-231.3264368
711912311911110570002_intra_H_migration_3_12	-231.432	0.095796	-231.3358449
711912311911110570002_Intra_R_Add_ExoTetCyclic_F_3_1_12	-231.472	0.099522	-231.3728425
711912311911110570002_r12_insertion_R_4_2_1	-231.43	0.091198	-231.3386377
711912311911110570002_R_Addition_MultipleBond_3_2_1	-231.451	0.096056	-231.3547017
711912311911110570002_R_Addition_MultipleBond_3_5_6	-231.422	0.091076	-231.3311043
711952161721200720062_intra_H_migration_3_11	-231.448	0.096045	-231.3519757
711952161721200720062_intra_H_migration_suprafacial_2_6	-231.417	0.095813	-231.3209995

711952161721200720062_Intra_R_Add_ExoTetCyclic_F_3_1_12	-231.426	0.098711	-231.3272295
711952161721200720062_r12_insertion_R_11_9_1	-231.422	0.091	-231.3309393
711952161721200720062_R_Addition_MultipleBond_3_5_6	-231.419	0.091849	-231.3268487
711952161721200720062_R_Addition_MultipleBond_2_1_9	-231.328	0.092673	-231.2356904
711952311741200570042_intra_H_migration_2_6	-231.417	0.096328	-231.3203318
711952311741200570042_intra_H_migration_3_11	-231.412	0.092263	-231.3193698
711952311741200570042_intra_H_migration_9_4	-231.413	0.091523	-231.3218769
711952311741200570042_intra_H_migration_5_11	-231.432	0.095744	-231.3360076
711952311741200570042_intra_R_migration_2_9	-231.425	0.096839	-231.3284746
711952311741200570042_r12_insertion_R_9_1_11	-231.41	0.090798	-231.3188859
711952311741200570042_r13_insertion_ROR_4_2_1_9	-231.422	0.094659	-231.3277657
,712193323793121330062	-231.455	0.100467	-231.3542784
,711912462231090120002	-231.462	0.100102	-231.3621332
,711912351721200630002	-231.458	0.099392	-231.3588813
,711912312361110120002	-231.497	0.098192	-231.3986427
,711952422191130120002	-231.468	0.098798	-231.368874
,711952442360980080002	-231.455	0.099836	-231.3555361
,711912311911110570002	-231.49	0.097714	-231.3922076
,711952161721200720062	-231.499	0.100198	-231.3992965
,711952221681140760062	-231.458	0.100403	-231.3579155
,711952311741200570042	-231.489	0.100483	-231.3889493
,711952271911110530042	-231.478	0.100885	-231.3769966
,711912311781240570002	-231.505	0.100307	-231.4042471
,711912421971130380002	-231.486	0.098085	-231.3879031
,711912291971130510002	-231.508	0.101241	-231.4063773
,712153574202750690002	-231.472	0.10031	-231.3718643
,711952271761240550042	-231.472	0.099096	-231.3729178
,712193423882780860062	-231.446	0.100296	-231.345362
,711912461931090420002	-231.458	0.099031	-231.3590396
,270630410040000000002	-77.781	0.036652	-77.74437721
,441041030570000000001	-153.65	0.055707	-153.59396
,150390060000000000002	-39.7755	0.02972	-39.74577797
,561281360900380000001	-191.674	0.061655	-191.6122869
,300540360000000000001	-114.382	0.026801	-114.35473
,411131080600060000002	-117.039	0.066114	-116.972701
,691933002841740640042	-230.202	0.077127	-230.1253513
,290410170000000000002	-113.73	0.01321	-113.716979
,421261230750120000001	-117.728	0.07991	-117.6478682
,170170000000000000002	-75.6704	0.008635	-75.661767
,541501581120600060001	-155.642	0.082157	-155.5600809
,180340020000000000001	-76.3699	0.021679	-76.34825909

,531371410970450040002	-155.065	0.071624	-154.9933029
,531371300930560080002	-155.081	0.070463	-155.0108362
,541501561120600080001	-155.755	0.021681	-155.7333666

S4.2.3 R3

geometry name	L3 energy	L2 ZPE	total
712193173963061480122.out	-231.43	0.102028	-231.3276102
712193173963061480122_r13_insertion_ROR_3_2_1_8.out	-231.382	0.097203	-231.2851365
712153574652300240002.out	-231.437	0.100074	-231.3364757
711912312361110120002_Intra_R_Add_ExoTetCyclic_F_1_3_4.out	-231.436	0.099106	-231.3369497
280760560080000000001.out	-78.4567	0.051166	-231.335078
430910730510000000002.out	-152.973	0.043372	

S4.2.4 R4

geometry name	L3 energy	L2 ZPE	total
,712193434003141140082	-231.417	0.100754	-462.6375822
,711952281671190640082	-231.42	0.098453	-462.643547
,711912461931090420002	-231.421	0.099031	-462.6721869
,712193283463422100202	-231.454	0.103743	-462.7004817
,711912421971130380002	-231.448	0.098077	-462.7188466
,711912291971130510002	-231.47	0.101119	-344.5458556
,280280000000000000001	-113.182	0.005117	-231.3568211
,431391510850120000002	-118.268	0.088382	-196.5853131
,280760560080000000001	-78.4567	0.051163	-231.3244062
,430910860380000000002	-152.962	0.042669	-267.2541203
,300540360000000000001	-114.362	0.026801	-231.3285561
,411131060600080000002	-117.059	0.066199	-348.2877314
,712193434003141140082_r12_insertion_R_4_3_7	-231.395	0.100109	-462.5859362
,712193434003141140082_r13_insertion_ROR_3_2_1_7	-231.39	0.098695	-462.5882151
,711952281671190640082_Intra_R_Add_Endocyclic_F_2_4	-231.396	0.099637	-462.612832
,712193283463422100202_Korcek_step2_4_11	-231.416	0.100051	-462.6365838
,711912291971130510002_intra_H_migration_3_8	-231.416	0.09535	-462.6675702
,711912291971130510002_r12_insertion_R_3_4_5	-231.444	0.096675	-462.653728
,711912461931090420002_R_Addition_MultipleBond_7_1_2	-231.404	0.097152	-462.5971706
,711952281671190640082_R_Addition_MultipleBond_2_1_7	-231.387	0.096384	-231.290551

S4.2.5 anti-ROO1

geometry name	L3 Energy	L2 ZPE	total
1032794335695244221482_intra_H_migration_14_4	-381.5874686	0.105722	-381.481747

1032794335695244221482_HO2_Elimination_from_PeroxyRadical_4_14	-381.5868253	0.105183	-381.481642
1032834225145074161852_Cyclic_Ether_Formation_2_12	-381.5931983	0.106486	-381.486712
1032834225145074161852_r12_insertion_R_2_3_10	-381.5900316	0.108171	-381.481861
1032834225145074161852_R_Addition_MultipleBond_2_1_10	-381.5857359	0.108213	-381.477523
1032834225145074161852_beta_delta_2_1_12_14_4	-381.5870248	0.10795	-381.479075
1032834075464884142042_intra_H_migration_14_4	-381.5883894	0.105905	-381.482484
1032834075464884142042_intra_H_migration_3_11	-381.5998782	0.106647	-381.493231
1032794335695244221482_intra_R_migration_14_10	-381.5720812	0.108181	-381.4639
,1032794335695244221482	-381.6474369	0.112117	-381.53532
,1032834225145074161852	-381.6227546	0.109542	-381.513213
,1032834075464884142042	-381.6353619	0.110431	-381.524931
,1032553252922271680662	-381.6264377	0.108297	-381.518141
,1032553213112141490832	-381.6188685	0.10793	-381.510939
,330490170000000000002	-150.7473735	0.014542	-150.732831
,702063003512521190061	-230.8508686	0.091474	-230.759395
,170170000000000000002	-75.65656473	0.00864	-75.6479247
,862624746286584661681	-305.9778594	0.096835	-305.881024
,862062612811410570001	-306.0570273	0.094054	-305.962973

S4.2.6 syn-ROO1

geometry name	L3 Energy	L2 ZPE	total
1032553363282331340492_intra_H_migration_10_7.out	-381.6251282	0.106464	-381.518664
1032553363282331340492_r12_insertion_R_10_1_2.out	-381.6273706	0.10692	-381.520451
1032593183022431560572_Cyclic_Ether_Formation_2_12.out	-381.6041063	0.104831	-381.499275
1032593183022431560572_intra_R_migration_2_12.out	-381.5961509	0.105772	-381.490379
1032794335695244221482_HO2_Elimination_from_PeroxyRadical_4_14.out	-381.5957796	0.105199	-381.490581
1032794335695244221482_intra_H_migration_14_4.out	-381.5942295	0.105752	-381.488477
1032794335695244221482_r12_insertion_R_12_1_10.out	-381.5752387	0.107845	-381.467394
1032794335695244221482_r12_insertion_R_2_1_12.out	-381.5721597	0.103	-381.46916
1032834225145074161852_beta_delta_2_3_10_1_12.out	-381.5957836	0.108163	-381.487621
1032834225145074161852_Intra_R_Add_ExoTetCyclic_F_2_12_14.out	-381.6005199	0.106492	-381.494028
1032834225145074161852_r12_insertion_R_10_1_12.out	-381.5910713	0.107748	-381.483323
1032834225145074161852_r12_insertion_R_1_10_3.out	-381.5943139	0.108186	-381.486128
1032834335505263841702_Cyclic_Ether_Formation_6_12.out	-381.59053	0.107767	-381.482763
1032834335505263841702_intra_H_migration_12_4.out	-381.6117536	0.109001	-381.502753
1032834335505263841702_intra_H_migration_12_5.out	-381.6117538	0.108886	-381.502868
1032834335505263841702_intra_H_migration_6_7.out	-381.6018469	0.106127	-381.49572
1032834335505263841702_r12_insertion_R_1_12_14.out	-381.601474	0.108928	-381.492546
1032834335505263841702_r12_insertion_R_1_2_3.out	-381.6027865	0.107084	-381.495703
1032834335505263841702_r13_insertion_ROR_3_2_1_10.out	-381.6027895	0.107072	-381.495717
1032513323492221340512.out	-381.5932297	0.108331	-381.484899
1032553213112141490832.out	-381.6233492	0.107937	-381.515412

1032553252922271680662.out	-381.6334639	0.108108	-381.525356
1032553323472351320382.out	-381.6668772	0.110873	-381.556004
1032553363282331340492.out	-381.6298951	0.108187	-381.521708
1032593183022431560572.out	-381.626987	0.10687	-381.520117
1032794335695244221482.out	-381.6547911	0.112302	-381.542489
1032794635545263921102.out	-381.7199895	0.112011	-381.607978
1032834225145074161852.out	-381.6302956	0.109509	-381.520787
1032834335505263841702.out	-381.6329118	0.109676	-381.523236
1701700000000000000002.out	-75.65709748	0.008636	-75.6484615
3203200000000000000003.out	-150.1659713	0.003904	-150.162067
3304901700000000000002.out	-150.7508277	0.014543	-150.736285
4111310606000800000002.out	-117.0594999	0.066232	-116.993268
6211811106201900000001.out	-264.5918495	0.037162	-264.554688
7018219615010406000081.out	-230.861848	0.089363	-230.772485
7020630035125211900061.out	-230.8543918	0.091491	-230.762901
7121932840228413600122.out	-231.4281258	0.101953	-231.326173
8623835932823920209 81.out	-305.993159	0.096158	-305.897001
8626247462865846616 81.out	-305.9850964	0.096878	-305.888218
8626247465276260621 21.out	-306.0062535	0.099714	-305.906539

S4.2.7 anti-ROO2

geometry name	L3 Energy	L2 ZPE	total
1022422782862701420061.out	-381.08906	0.097849	-380.991211
1032513323622861080002.out	-381.6717215	0.109647	-381.562075
1032513624072430760002.out	-381.7343661	0.109026	-381.62534
1032553193432801360062.out	-381.667679	0.109542	-381.558137
1032553213372781420062.out	-381.6497792	0.106833	-381.542946
1032553213822780970062.out	-381.656481	0.106522	-381.549959
1032794335955623191362.out	-381.643052	0.111766	-381.531286
1032834075575353511512.out	-381.6290864	0.10998	-381.519106
1032834185635133391512.out	-381.6262311	0.109841	-381.51639
1701700000000000000002.out	-75.65709748	0.008637	-75.6484605
2904101700000000000002.out	-113.7114533	0.013206	-113.698247
3304901700000000000002.out	-150.7508281	0.014544	-150.736284
7017820315710505700001.out	-230.8975666	0.089984	-230.807583
7020628934527413100061.out	-230.8581852	0.091228	-230.766957
7020630035125211900061.out	-230.8543932	0.091451	-230.762942

862062722861820000001.out	-306.0500106	0.094025	-305.955986
862343734213371620571.out	-306.020881	0.095266	-305.925615
862624746286584661681.out	-305.9852686	0.096833	-305.888436
862624746847083821241.out	-305.989476	0.096572	-305.892904
1032553213372781420062_Cyclic_Ether_Formation_3_12.out	-381.627911	0.104336	-381.523575
1032553213372781420062_HO2_Elimination_from_PeroxyRadical_11_14.out	-381.626337	0.106614	-381.519723
1032553213372781420062_intra_H_migration_3_10.out	-381.589069	0.104275	-381.484794
1032553213372781420062_intra_H_migration_3_9.out	-381.6113309	0.103633	-381.507698
1032553213372781420062_Intra_R_Add_Endocyclic_F_3_8.out	-381.5832629	0.105482	-381.477781
1032553213372781420062_intra_R_migration_3_14.out	-381.6129701	0.104288	-381.508682
1032553213372781420062_intra_R_migration_3_1.out	-381.6287791	0.107323	-381.521456
1032553213372781420062_r12_insertion_R_1_2_12.out	-381.6276696	0.106093	-381.521577
1032553213372781420062_r12_insertion_R_13_2_1.out	-381.582097	0.098987	-381.48311
1032553213372781420062_r12_insertion_R_2_12_14.out	-381.605359	0.102391	-381.502968
1032553213822780970062_intra_R_migration_1_3.out	-381.6335115	0.107053	-381.526458
1032794335955623191362_HO2_Elimination_from_PeroxyRadical_11_14.out	-381.5755699	0.104881	-381.470689
1032794335955623191362_HO2_Elimination_from_PeroxyRadical_9_14.out	-381.575777	0.105005	-381.470772
1032794335955623191362_intra_H_migration_14_11.out	-381.5981714	0.106567	-381.491604
1032794335955623191362_intra_H_migration_14_9.out	-381.5952709	0.10667	-381.488601
1032834075575353511512_Cyclic_Ether_Formation_3_12.out	-381.6107913	0.10692	-381.503871
1032834075575353511512_intra_H_migration_3_11.out	-381.5981748	0.106558	-381.491617
1032834075575353511512_intra_H_migration_3_13.out	-381.6053287	0.106677	-381.498652
1032834075575353511512_Intra_R_Add_ExoTetCyclic_F_3_1_9.out	-381.5865824	0.105952	-381.48063
1032834075575353511512_r12_insertion_R_1_2_12.out	-381.5985552	0.108096	-381.490459
1032834075575353511512_r13_insertion_ROR_3_2_1_8.out	-381.6053222	0.106698	-381.498624
1032834185635133391512_intra_H_migration_1_13.out	-381.5815891	0.106073	-381.475516
1032834185635133391512_r12_insertion_R_1_2_12.out	-381.594104	0.108296	-381.485808
1032834185635133391512_r12_insertion_R_2_12_14.out	-381.6065332	0.106804	-381.499729
1032834185635133391512_r12_insertion_R_2_3_8.out	-381.5838061	0.10592	-381.477886

S4.2.8 syn-ROO2

1032553363542781120042.out	-381.6127754	0.10789	-381.504885
1032794335955623191362_HO2_Elimination_from_PeroxyRadical_13_12.out	-381.5675601	0.105012	-381.462548
1032794335955623191362_intra_H_migration_12_13.out	-381.5876529	0.106767	-381.480886
1032794335955623191362_intra_H_migration_12_5.out	-381.5941744	0.106048	-381.488126
1032794335955623191362.out	-381.6352271	0.111952	-381.523275

103283418563513339l512.out	-381.6200349	0.109876	-381.510159
1032834335765453171472_Cyclic_Ether_Formation_4_10.out	-381.5779767	0.107482	-381.470495
1032834335765453171472_intra_R_migration_4_10.out	-381.5779767	0.107482	-381.470495
1032834335765453171472.out	-381.6060638	0.109196	-381.496868
1032834335765453171472_r12_insertion_R_1_2_3.out	-381.5812651	0.107816	-381.473449
1032834335765453171472_r12_insertion_R_2_3_8.out	-381.5881093	0.108384	-381.479725
1701700000000000000002.out	-75.65656468	0.008637	-75.6479277
3304901700000000000002.out	-150.7473736	0.014543	-150.732831
7020630035125211900061.out	-230.8508685	0.091471	-230.759397
8621025821213409403 81.out	-306.0173805	0.094672	-305.922708
8626247467867656824 01.out	-305.9747046	0.098661	-305.876044
synQOOH21CETS.out	-381.6005178	0.106494	-381.494024
synQOOH21HO2.out	-381.5916719	0.108251	-381.483421
synQOOH21RO.out	-381.5830965	0.105833	-381.477263
synQOOH24decom.out	-381.6042261	0.105627	-381.498599

S4.2.9 ROO3

1032794336255753100462_HO2_Elimination_from_PeroxyRadical_4_14.out	-381.600765	0.104953	-381.495812
1032794336255753100462_intra_H_migration_10_12.out	-381.6065016	0.106216	-381.500286
1032794336255753100462_intra_H_migration_14_4.out	-381.5965649	0.10511	-381.491455
1032794336255753100462_intra_H_migration_14_7.out	-381.5938258	0.105274	-381.488552
1032794336255753100462_intra_R_migration_14_10.out	-381.5825235	0.107515	-381.475008
1032794336255753100462_r12_insertion_R_1_2_3.out	-381.5847809	0.106768	-381.478013
1032794336255753100462_r12_insertion_R_7_6_3.out	-381.6043293	0.105629	-381.4987
1032834186085282960802_intra_H_migration_13_4.out	-381.5978776	0.10509	-381.492788
1032834186085282960802_r12_insertion_R_2_3_10.out	-381.602968	0.105805	-381.497163
1032834186085282960802_r12_insertion_R_3_13_14.out	-381.602969	0.105824	-381.497145
1032834186085282960802_r12_insertion_R_6_3_10.out	-381.6029705	0.105834	-381.497137
1032834225705693100612_Cyclic_Ether_Formation_2_13.out	-381.6052133	0.10596	-381.499253
1032834225705693100612_intra_H_migration_2_5.out	-381.5965667	0.105081	-381.491486
1032834225705693100612_intra_R_migration_2_10.out	-381.5940874	0.107788	-381.486299
1032834225705693100612_intra_R_migration_2_13.out	-381.5979846	0.107531	-381.490454
1032834225705693100612_r12_insertion_R_1_10_3.out	-381.5979537	0.108706	-381.489248
1032513213542841290002.out	-381.6014662	0.107828	-381.493638
1032513213602331230512.out	-381.6831047	0.109907	-381.573198
1032553033062561730462.out	-381.6437035	0.107493	-381.536211
1032553103162871500212.out	-381.6239819	0.106934	-381.517048
1032553363992780670042.out	-381.636132	0.107791	-381.528341
1032794336255753100462.out	-381.6584456	0.111572	-381.546874
1032834186085282960802.out	-381.6431973	0.110023	-381.533174
1032834225705693100612.out	-381.6325284	0.109017	-381.523511

10328343359157130608002.out	-381.6323669	0.109001	-381.523366
1701700000000000000002.out	-75.65709748	0.008636	-75.6484615
3203200000000000000003.out	-150.1659712	0.003904	-150.162067
3304901700000000000002.out	-150.7508278	0.014544	-150.736284
7020628934527413100061.out	-230.8581847	0.09122	-230.766965
7020630036627611000081.out	-230.8640004	0.091979	-230.772021
7121931739630614800122.out	-231.4296354	0.102047	-231.327588
8620626128114105700001.out	-306.0596499	0.094073	-305.965577
8626247468470838212411.out	-305.9894744	0.096588	-305.892886

S4.2.10 ROO4

geometry name	L3 Energy	L2 ZPE	total
1032553213412331340512_r12_insertion_R_3_2_4.out	-381.6060747	0.105458	-381.500617
1032553213412331340512_r12_insertion_R_4_2_1.out	-381.582145	0.100212	-381.481933
1032794335394813321702_HO2_Elimination_from_PeroxyRadical_13_14.out	-381.5690802	0.105855	-381.463225
1032794335394813321702_intra_H_migration_14_11.out	-381.5892046	0.107659	-381.481546
1032794335394813321702_intra_H_migration_14_13.out	-381.5842759	0.107438	-381.476838
1032794335394813321702_intra_H_migration_14_4.out	-381.5865179	0.106513	-381.480005
1032794335394813321702_r13_insertion_ROR_13_3_6_8.out	-381.5463847	0.106487	-381.439898
1032834075014433582002_intra_H_migration_14_4.out	-381.5814722	0.107823	-381.473649
1032834075014433582002_intra_H_migration_14_7.out	-381.5902875	0.107358	-381.48293
1032834075014433582002_intra_H_migration_3_13.out	-381.5842734	0.107467	-381.476806
1032834075014433582002_Intra_R_Add_ExoTetCyclic_F_3_1_11.out	-381.5716293	0.106463	-381.465166
1032834075014433582002_Intra_R_Add_ExoTetCyclic_F_3_8_14.out	-381.5994293	0.108105	-381.491324
1032834075014433582002_R_Addition_MultipleBond_3_6_8.out	-381.5807975	0.108853	-381.471945
1032834185224383181662_intra_H_migration_8_4.out	-381.5593027	0.108408	-381.450895
1032834185224383181662_Intra_R_Add_ExoTetCyclic_F_1_3_13.out	-381.5714075	0.10961	-381.461797
1032834224994513281772_Cyclic_Ether_Formation_2_8.out	-381.5728466	0.107896	-381.464951
1032834224994513281772_intra_H_migration_2_4.out	-381.5902727	0.106521	-381.483752
1032834224994513281772_intra_R_migration_2_10.out	-381.5724006	0.10949	-381.462911
1032834224994513281772_r12_insertion_R_1_10_3.out	-381.5735988	0.109639	-381.46396
1022422933072121170491.out	-381.0858477	0.098778	-380.98707
1032553212962181640662.out	-381.6475898	0.109151	-381.538439
1032553213412331340512.out	-381.6461052	0.106725	-381.53938
1032553252772121680812.out	-381.6038844	0.108354	-381.49553
1032553363432331190492.out	-381.6134814	0.108327	-381.505154
1032794335394813321702.out	-381.6309265	0.112874	-381.518053
1032794636255453100462.out	-381.7103592	0.110686	-381.599673
1032834075014433582002.out	-381.6158193	0.111964	-381.503855
1032834185224383181662.out	-381.6138887	0.110954	-381.502935
1032834224994513281772.out	-381.6059789	0.110563	-381.495416

170170000000000000002.out	-75.65656473	0.008636	-75.6479287
280760560080000000001.out	-78.45667149	0.051165	-78.4055065
330490170000000000002.out	-150.7473752	0.014542	-150.732833
702063003662761100081.out	-230.8610899	0.091987	-230.769103
751551631410490170002.out	-303.1577525	0.052527	-303.105225
851932482321350420002.out	-305.3570391	0.080448	-305.276591
862062722421580680001.out	-306.0505929	0.094411	-305.956182
862383584133292070551.out	-305.991805	0.097266	-305.894539
862624746286284361441.out	-305.9874099	0.097513	-305.889897
862624746786765682401.out	-305.974704	0.098659	-305.876045

S5. adiabatic ionization energies calculated at the CBS-QB3 level of theory at 0 K

m/z	Species	Adiabatic Ionization Energy (eV)
56	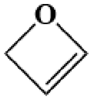	9.01
	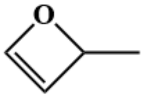	8.77
	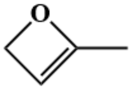	8.55
70	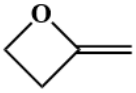	8.57
	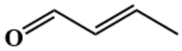	9.81
	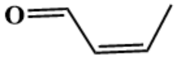	9.77
76	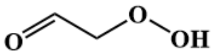	9.61
	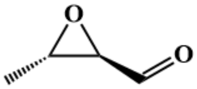	9.96
	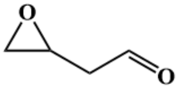	9.88
	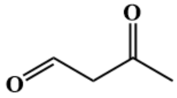	9.54
86	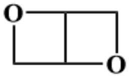	9.34
	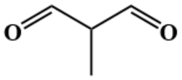	9.91
	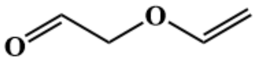	9.14

S6. potential energy surfaces

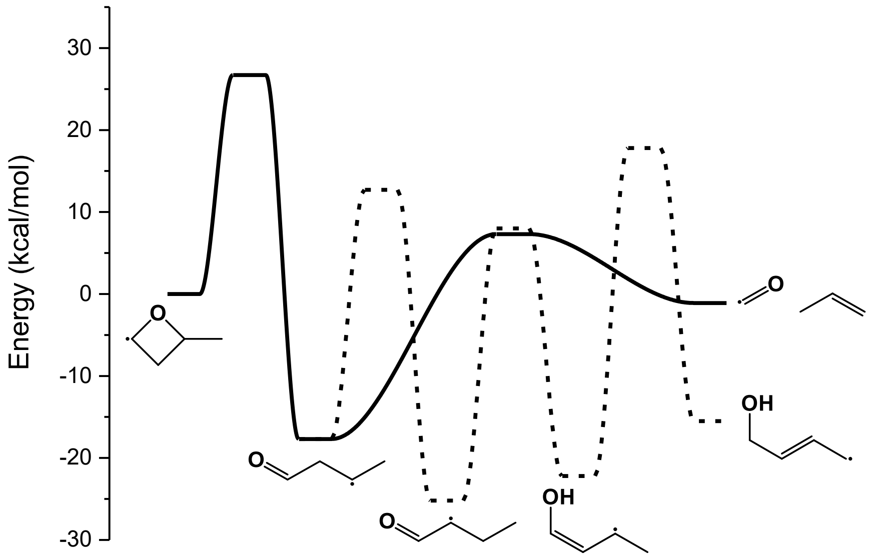

Figure S6.1 PES for R1

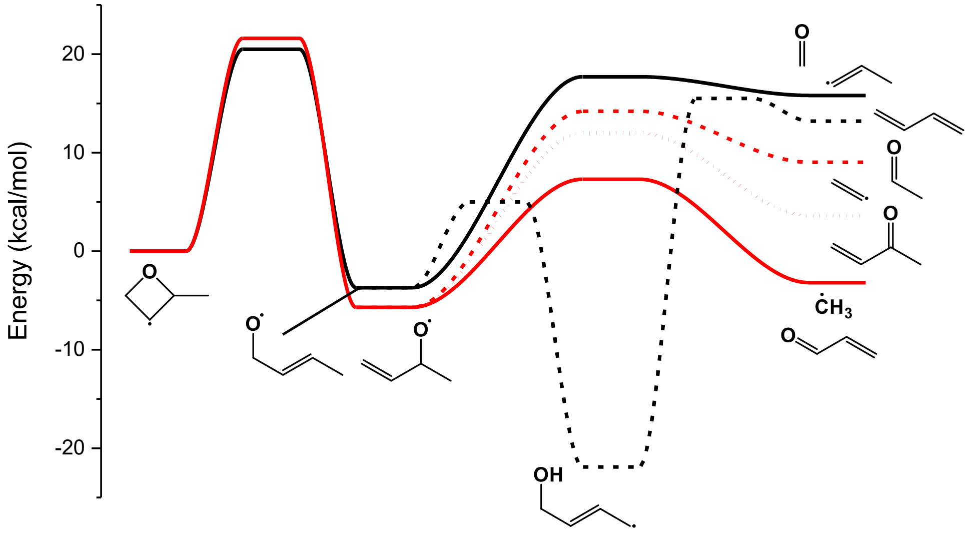

Figure S6.2 PES for R2

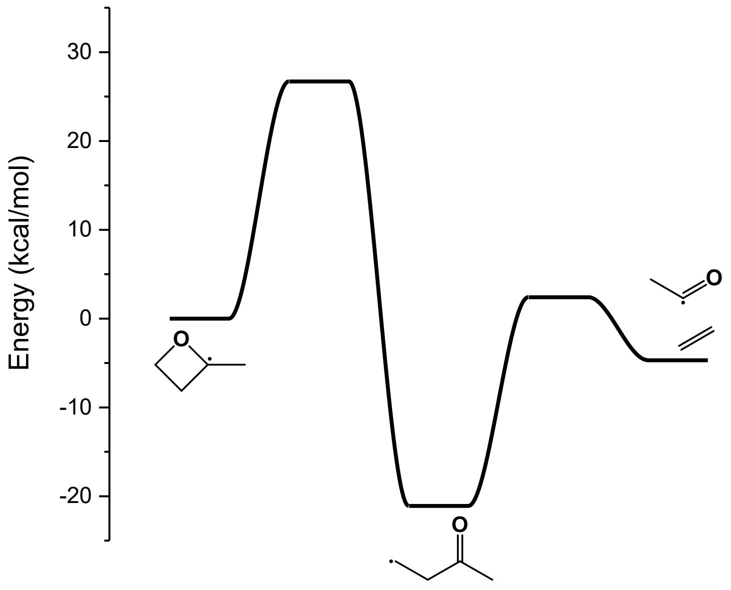

Figure S6.3 R3 PES

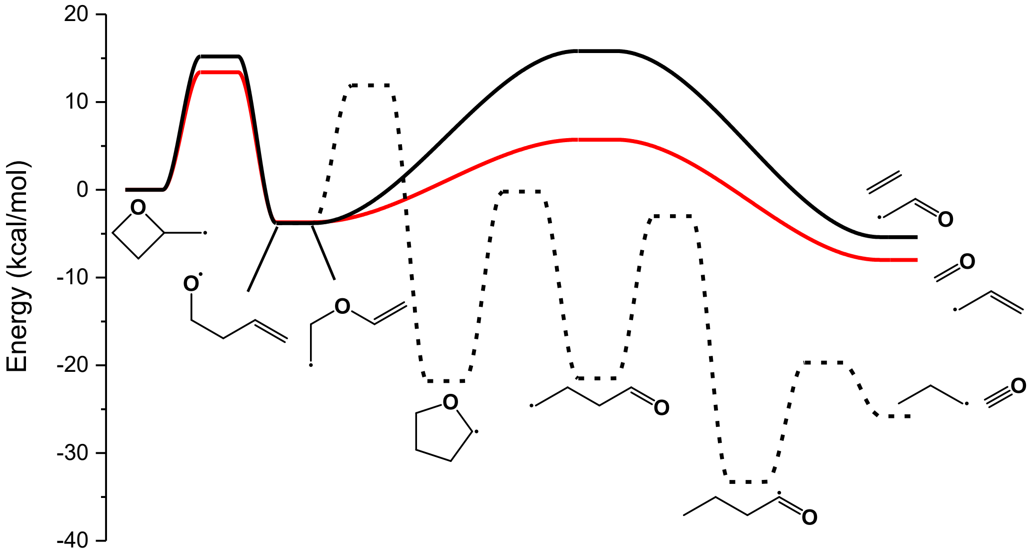

Figure S6.4 R4 PES

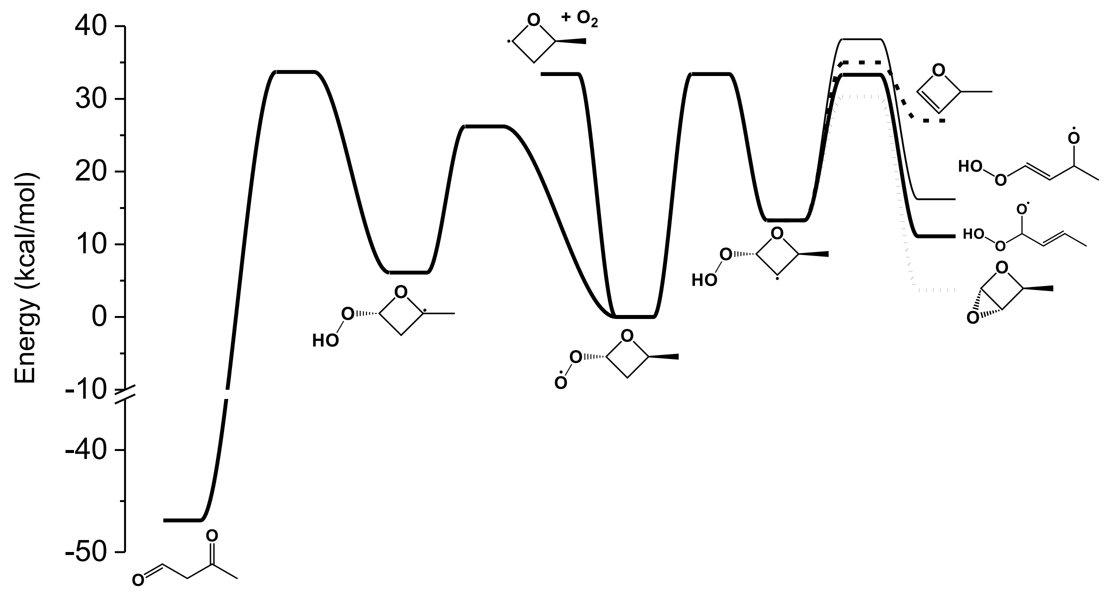

Figure S6.5 *anti*-ROO1 PES

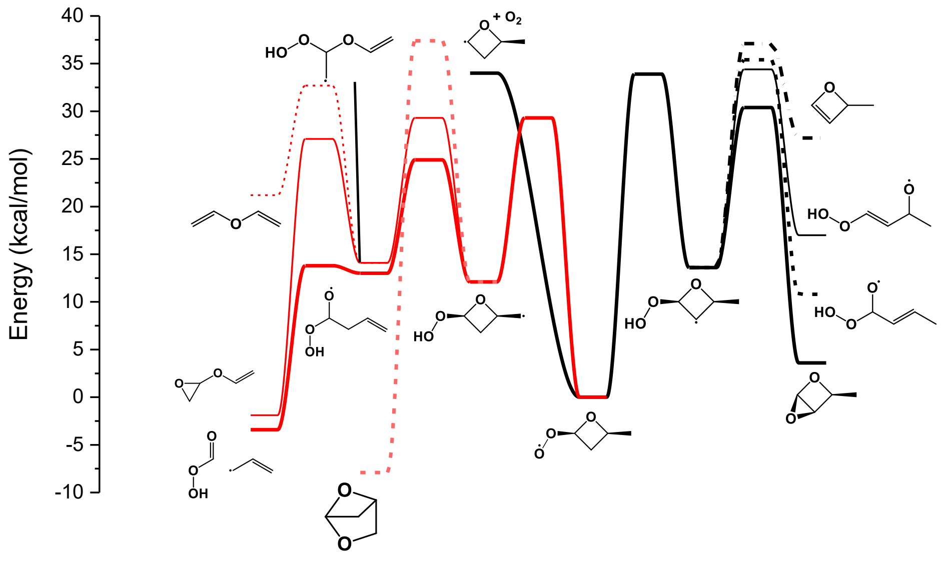

Figure S6.6 *syn*-ROO1 PES

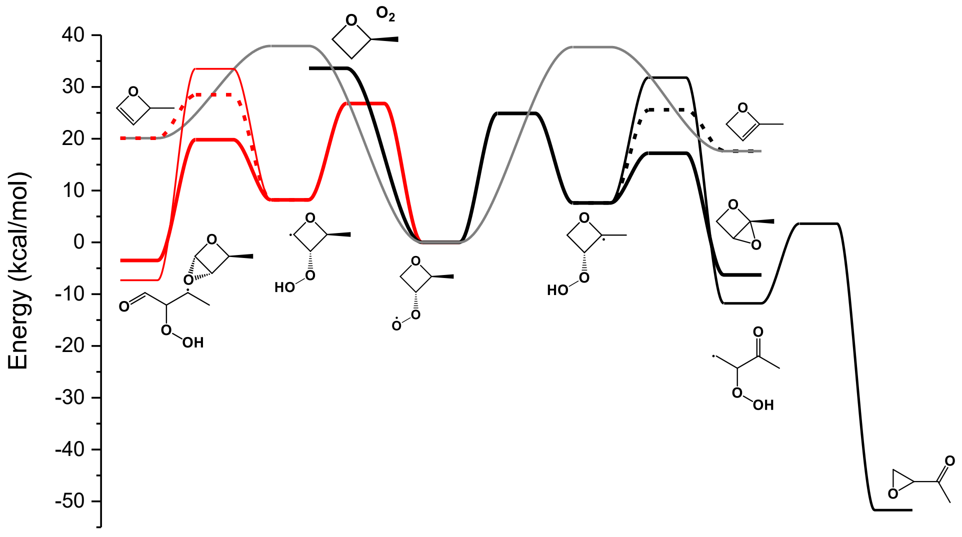

Figure S6.7 *anti*-ROO2 PES

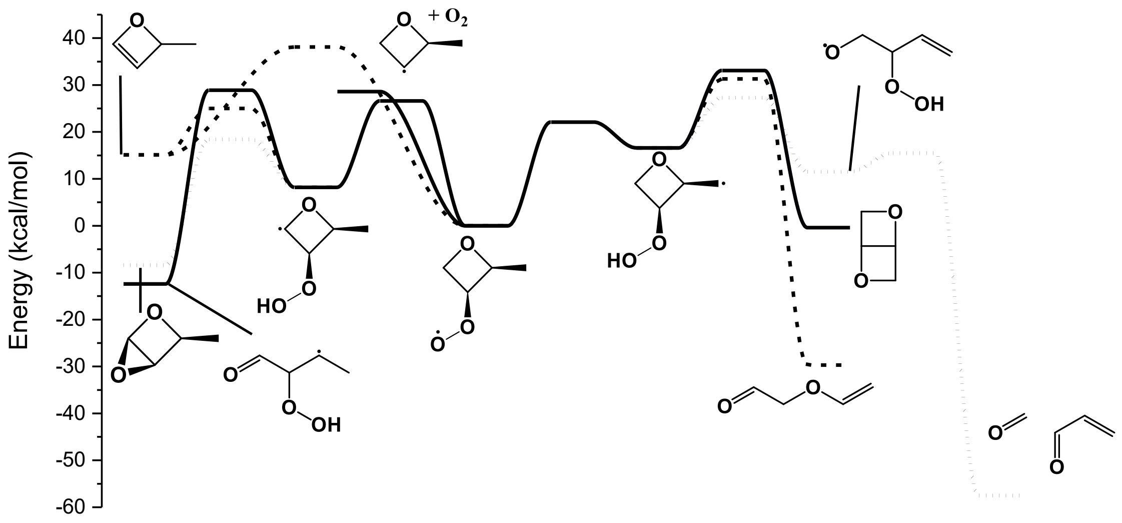

Figure S6.8 *syn*-ROO2 PES

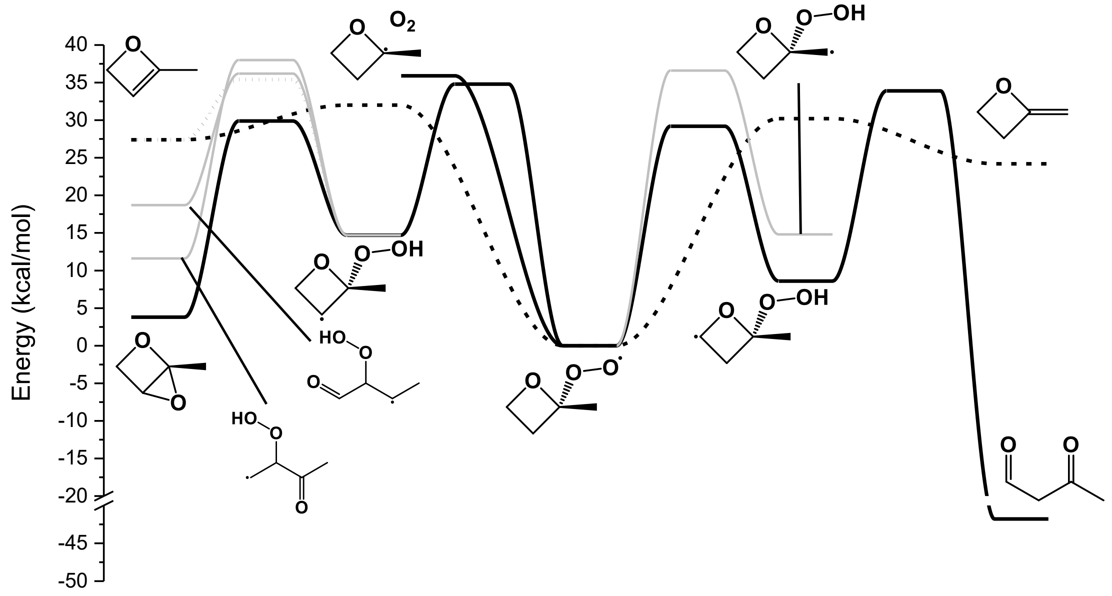

Figure S6.9 ROO3 PES

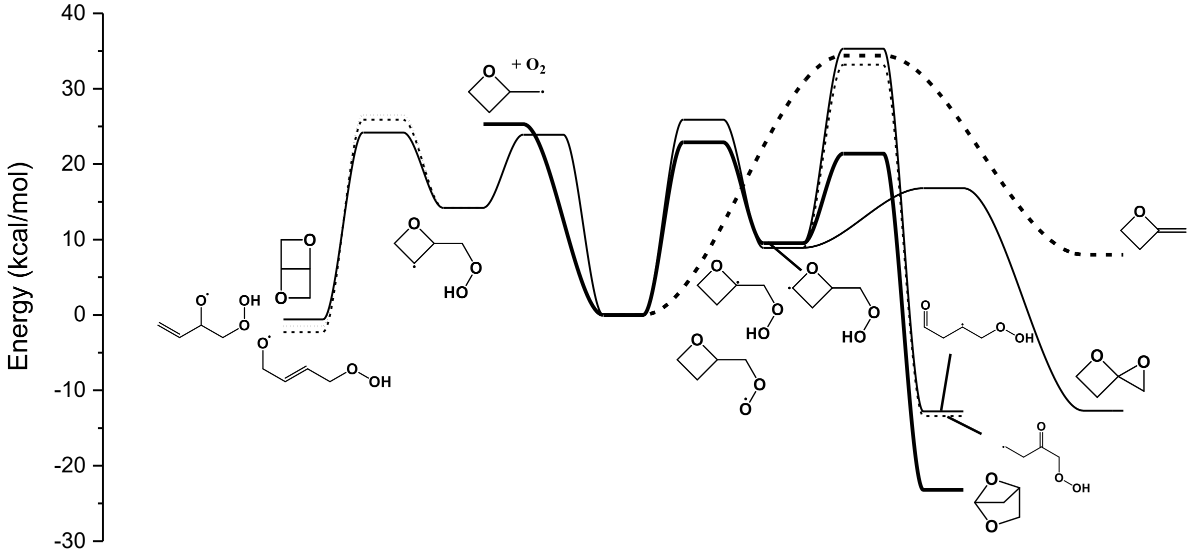

Figure S6.10 ROO4 PES

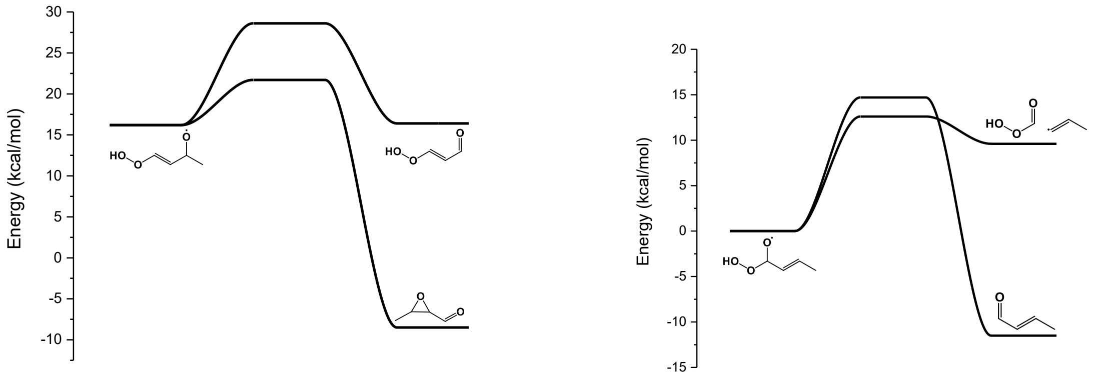

Figure S6.11 PES for ring-opened QOOH12

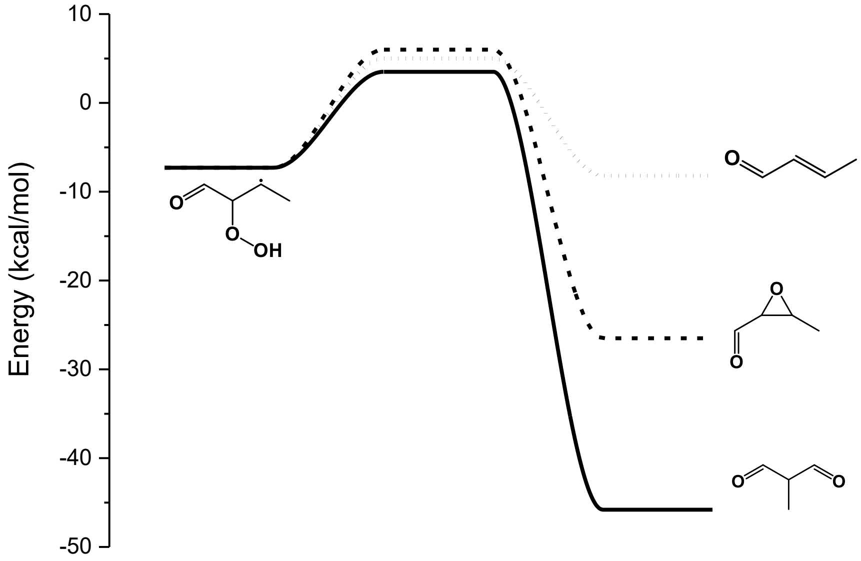

Figure S6.12 PES for ring-opened QOOH21

S7. difference mass spectrum integrated 30 ms post-photolysis at 800 K and 6 Torr over the photon energy range 8.5 – 11.0 eV

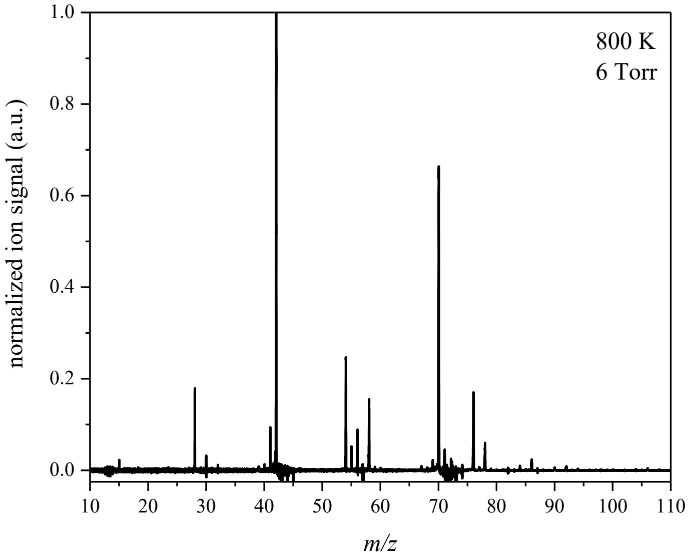

S8. methylperoxy radical (CH_3OO) absolute photoionization spectrum

To enable the quantification of methyl peroxy reaction intermediates, we measured the absolute photoionization spectrum of CH_3OO using the same multiplexed PIMS apparatus that was employed in all the work described here. CH_3OO radicals were produced as shown below, by reacting known concentrations of methyl (CH_3) with O_2 in He bath gas at total P = 10 Torr, T = 300 K. Methyl radicals were in turn generated by H-abstraction from CH_4 using F atoms, formed by laser photolysis of XeF_2 at 248 nm.

$XeF_2 + h\nu(248\ nm) \rightarrow Xe + 2\ F$	
$F + CH_4 \rightarrow HF + CH_3$	R1
$CH_3 + O_2 \rightarrow CH_3OO$	R2

Methane was delivered to the reactor from a 1% CH_4 mix in He; XeF_2 was delivered by sublimating solid XeF_2 powder (Sigma-Aldrich) in a glass bubbler under a flow of He. The XeF_2/He flow was mixed with pure O_2, CH_4/He and excess He flows on the fly. An excimer laser pulse created typically $(1 - 2)\times10^{12}$ F atoms per cm^3. Methane concentration was $\sim1\times10^{14}\ cm^{-3}$, sufficiently high to ensure a complete conversion of F atoms into HF with negligible competing losses. The depletion of methane was monitored in situ via photoionization at 13.5 eV, providing a firm determination of the initial concentration of methyl radicals.

Methyl was reacted with variable concentrations of O_2 in the $(0 - 4.4)\times10^{16}\ cm^{-3}$ range. The time-resolved decay of methyl radicals and the simultaneous production of CH_3OO were monitored as a function of $[O_2]$ via the ion peaks at *m/z* = 15 (CH_3^+) and 47 (CH_3OO^+), respectively. This experiment was repeated twice, using ionization energies of 10.65 and 11 eV. The experimental time traces of CH_3 and CH_3OO are shown. The decay of methyl and formation of methyl peroxy were fit to a chemical model, comprised of reactions R1 – R5. The self-reaction of methyl (R3) has been investigated most recently by Fockenberg and co-workers [1] and is expected to contribute to the decay of CH_3 in the absence of O_2. R4 and R5 represent the combined first-order losses of methyl and methyl peroxy, respectively, including wall reactions and reactions with impurities.

$CH_3 + CH_3 \rightarrow C_2H_6$	R3
$CH_3 \rightarrow loss$	R4
$CH_3OO \rightarrow loss$	R5

The results of the model fits are also shown below. The model used global rate coefficients $k_1 = 6.1\times10^{-11}$ cm^3/molec·s (fixed to the literature value for F + CH_4) [2], $k_2 = 6\times10^{-14}$ cm^3/molec·s, $k_3 = 7\times10^{-11}$ cm^3/molec·s, $k_4 = 20\ s^{-1}$, and $k_5 = 1\ s^{-1}$. The reaction of CH_3 with O_2 is formally a termolecular association ($CH_3 + O_2 + M \rightarrow CH_3OO + M$). Because of this, its rate coefficient depends strongly on the identities and concentrations of various collision partners in the falloff regime, which includes the temperature and pressure of this study. Our optimized value of k_2 is in general close to that of Selzer and Bayes (at similar [He] and $[O_2]$ to ours), who have performed the most comprehensive analysis of R2 in various bath gases [3]. Our optimized k_3 is somewhat higher than the result of Fockenberg ($k_3 \sim5\times10^{-11}$ cm^3/molec·s) [1]. The modeled concentrations of CH_3 and CH_3OO were converted into ion counts using the known ionization cross-sections of methyl [4] and fitted ionizations of CH_3OO at 10.65 and 11 eV – (3.5±1) and (9±2) Mb, respectively.

The modeled traces reproduce the measured ion counts due to CH_3 and CH_3OO well at nearly all energies and conditions, with the exception of $[O_2] = 0$, where the model deviates appreciably from the data. These deviations are likely due to deficiencies of the sub-mechanism, used to model these data, and to imperfect

knowledge of rate coefficients. However, we note that the deviations of model from data or small changes in k_2 or k_3 do not affect the conclusions of this analysis. At the highest $[O_2]$ used here, nearly all (>97%) CH_3 is converted into CH_3OO, such that the ratio of the ionization cross-sections can be obtained directly from the signal heights of CH_3 at $[O_2] = 0$ and CH_3OO at the highest $[O_2]$. When analyzed that way, the ionization cross-sections are identical to those obtained from the model.

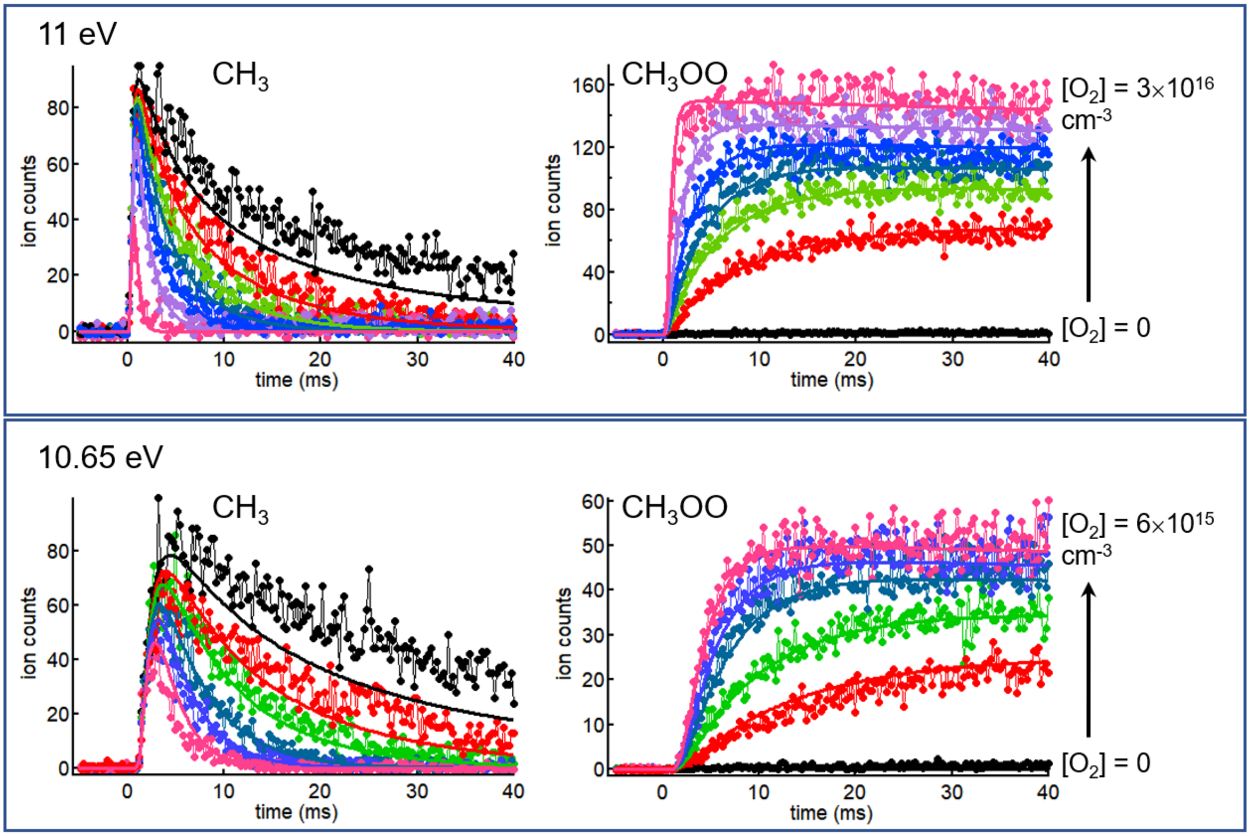

Ion counts at *m/z* = 15 (CH_3^+) and 47 (CH3OO$^+$) acquired using photoionization energies 11 eV (top) and 10.65 eV (bottom) during the reaction of methyl with O_2. Experimental data are shown as symbols, and model fits (as described in the text) as solid lines. The range of $[O_2]$ employed in this study is indicated on the right.

The ionization spectrum of CH_3OO was obtained by scanning the VUV ionization energy from 10 to 11.8 eV, using the reaction of CH_3 with O_2 at an oxygen concentration of 3×10^{16} cm^{-3}. The spectrum was then scaled to match the measured absolute ionization cross-sections of CH_3OO at 10.65 and 11 eV, as described above. This procedure enables placing the photoinization spectrum of methyl peroxy on an absolute basis.

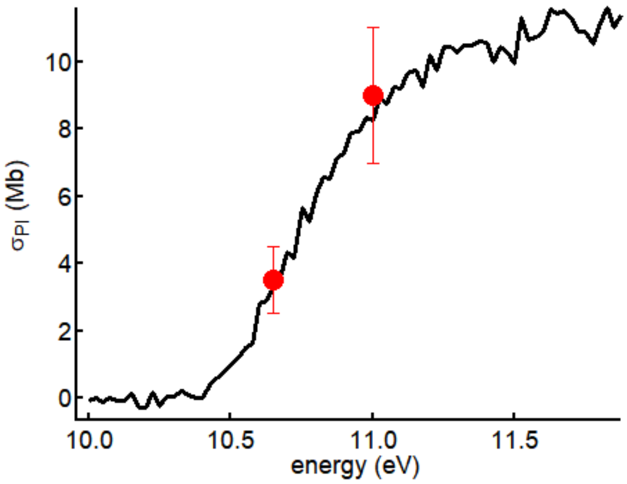

The photoionization spectrum of CH_3OO (black line), scaled to match the absolute PI cross-sections at 10.65 and 11 eV (red symbols)

References

[1] B. Wang, H. Hou, L.M. Yoder, J.T. Muckerman, C. Fockenberg, Experimental and theoretical investigations on the methyl-methyl recombination reaction, J. Phys. Chem. A 107 (2003) 11414-11426.
[2] R. Atkinson, D. Baulch, R. Cox, J. Crowley, R. Hampson Jr., J. Kerr, M. Rossi, J. Troe, Summary of evaluated kinetic and photochemical data for atmospheric chemistry, IUPAC subcommittee on gas kinetic data evaluation for atmospheric chemistry, 20 (2001).
[3] E.A. Selzer, K.D. Bayes, Pressure dependence of the rate of reaction of methyl radicals with oxygen, J. Phys. Chem. 87 (1983) 392-394.
[4] J.D. Savee, S. Soorkia, O. Welz, T.M. Shelby, C.A. Taatjes, D.L. Osborn, Absolute photoionization cross-section of the propargyl radical, J. Chem. Phys. 136 (2012) 134307.

S9. photoionization spectra and fittings for each mass quantified in Cl-initiated 2-methyloxetane oxidation at 650 K and 800 K

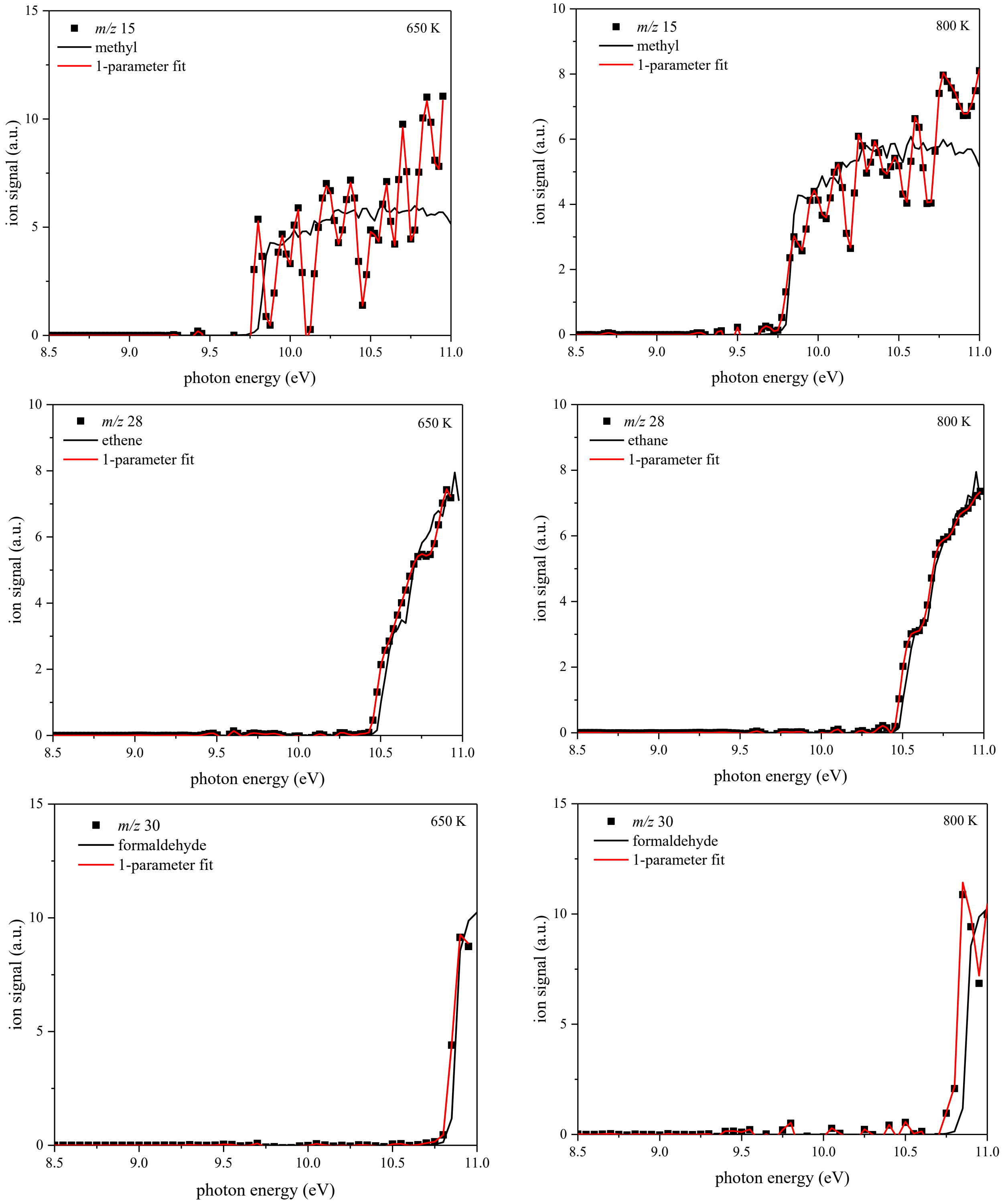

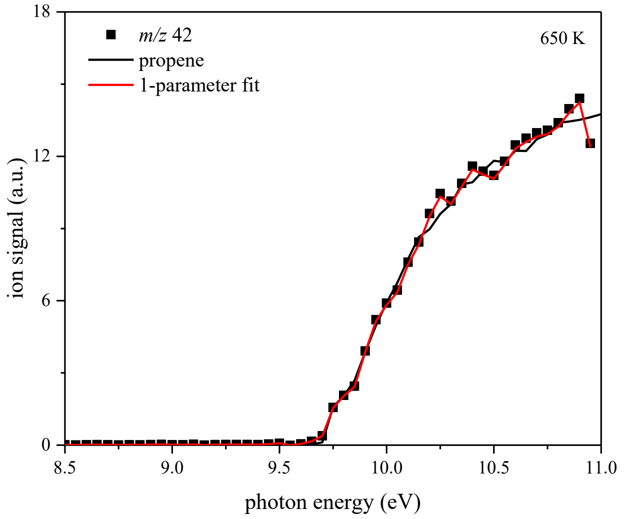

m/z 42
propene
1-parameter fit
650 K
ion signal (a.u.)
photon energy (eV)
0
6
12
18
8.5
9.0
9.5
10.0
10.5
11.0

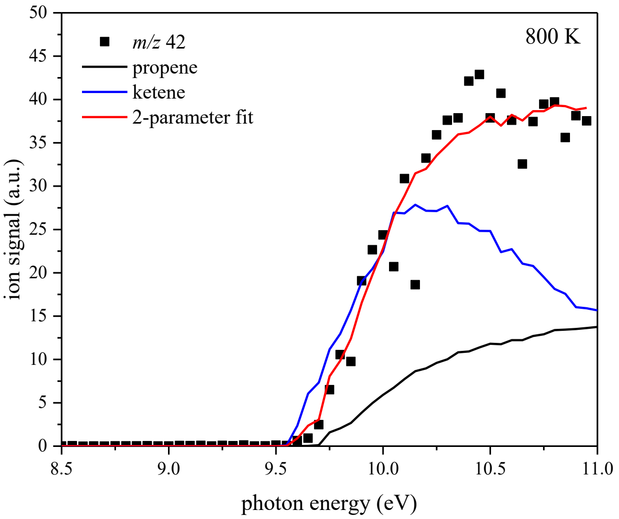

m/z 42
propene
ketene
2-parameter fit
800 K
ion signal (a.u.)
photon energy (eV)
0
5
10
15
20
25
30
35
40
45
50
8.5
9.0
9.5
10.0
10.5
11.0

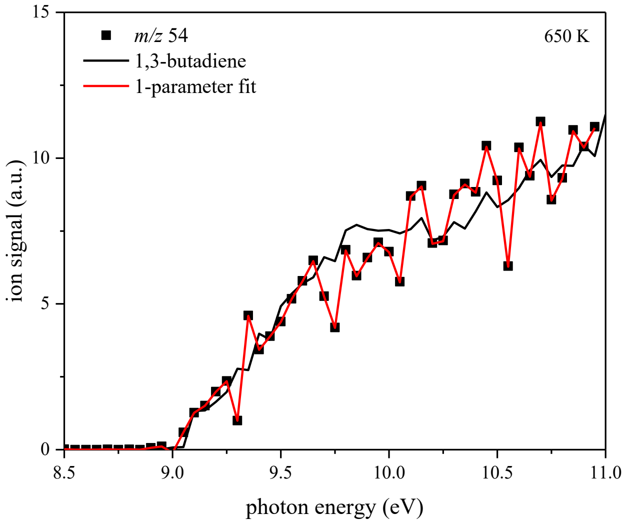

m/z 54
1,3-butadiene
1-parameter fit
650 K
ion signal (a.u.)
photon energy (eV)
0
5
10
15
8.5
9.0
9.5
10.0
10.5
11.0

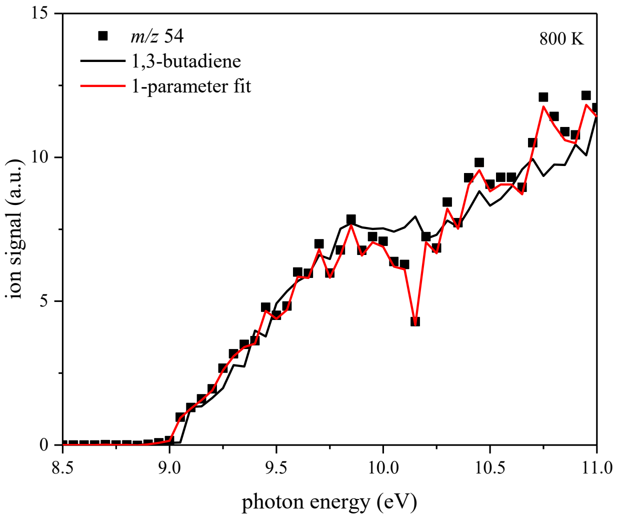

m/z 54
1,3-butadiene
1-parameter fit
800 K
ion signal (a.u.)
photon energy (eV)
0
5
10
15
8.5
9.0
9.5
10.0
10.5
11.0

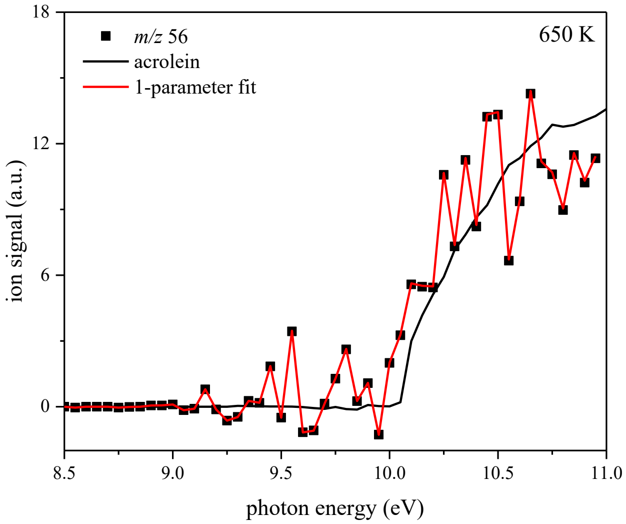

m/z 56
acrolein
1-parameter fit
650 K
ion signal (a.u.)
photon energy (eV)
0
6
12
18
8.5
9.0
9.5
10.0
10.5
11.0

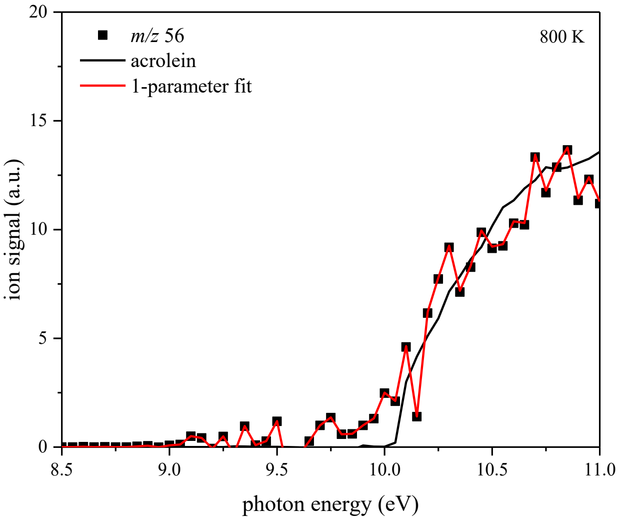

m/z 56
acrolein
1-parameter fit
800 K
ion signal (a.u.)
photon energy (eV)
0
5
10
15
20
8.5
9.0
9.5
10.0
10.5
11.0

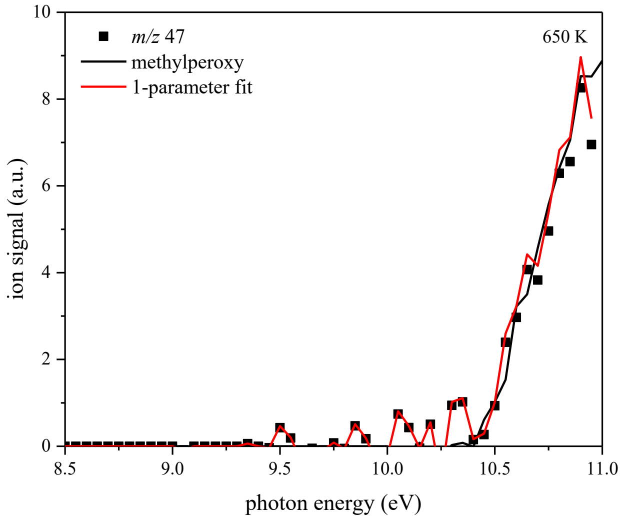
m/z 47
methylperoxy
1-parameter fit
650 K
ion signal (a.u.)
photon energy (eV)
0
2
4
6
8
10
8.5
9.0
9.5
10.0
10.5
11.0

S10. connecting products of MPIMS experiments to R and/or QOOH

m/z	species	R	QOOH
15	methyl	R1, R2, R3	*anti*/*syn*-QOOH12
28	ethene	R1, R2, R3, R4	
30	formaldehyde	R2, R4	*syn*-QOOH24
42	propene	R1, R2, R3	
	ketene	R3 (+ O_2), R4 (+ O_2)	
47	methylperoxy	produced from methyl + O_2	
54	1,3-butadiene	R1, R2, R3	
56	acrolein	R1, R2, R3	*syn*-QOOH24

S11. temperature dependence of ion signal at *m/z* 70 and *m/z* 86

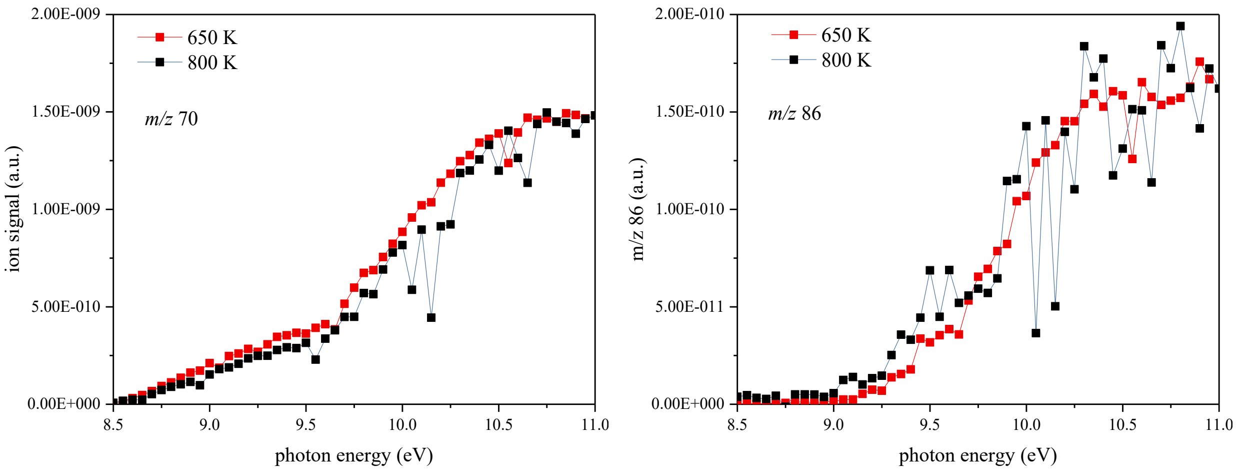

S12. cyclic ether isomers (*m/z* 86) derived from 2-methyloxetane oxidation

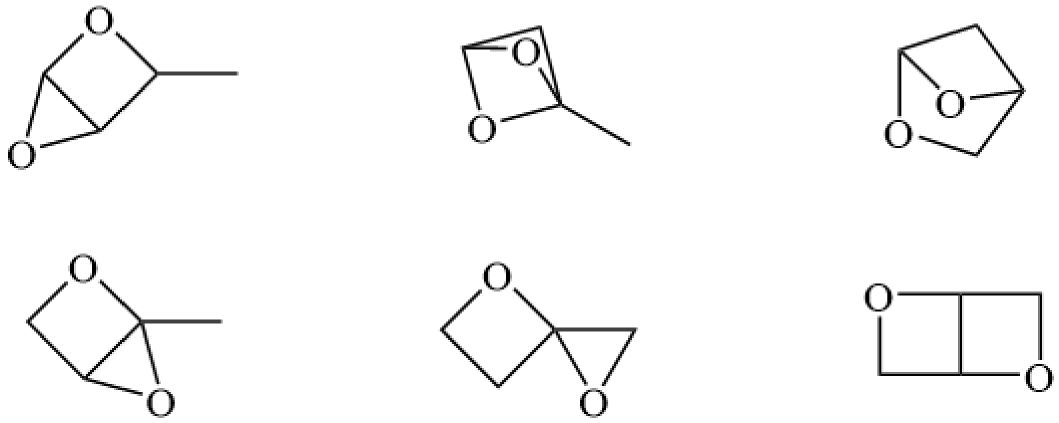

S13. ion signal at *m/z* 62 and 76

Species resulting from chain-chlorination (*i.e.*, Ṙ + Cl → RCl) were detected in the present experiments at *m/z* 106 and 108 in a ratio of 3.28:1, which corresponds to the isotopic abundance of ^{35}Cl and ^{37}Cl. Other peaks with a similar ratio were detected at *m/z* 62 and 64 (^{35}Cl/^{37}Cl = 3.16), as well as *m/z* 76 and 78 (^{35}Cl/^{37}Cl = 3.03). These masses correspond to vinyl chloride and allyl chloride, respectively. Both species are fragments resulting from the photoionization of RCl, as shown in **Figure S13a**.

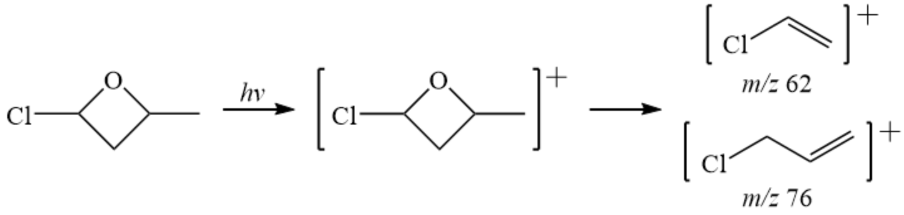

Figure S13a. Dissociation of RCl (*m/z* 106) cations into fragment ions of vinyl chloride (*m/z* 62) and allyl chloride (*m/z* 76).

However, *m/z* 62 and 76 also corresponds to performic acid and hydroperoxy acetaldehyde, which are small ketohydroperoxides that can form during the oxidation of 2-methyloxetane. These species were found to be energetically accessible in the potential energy surfaces constructed in the present work. Reaction mechanisms to performic acid (*m/z* 62) and hydroperoxy acetaldehyde (*m/z* 76) are shown in **Figure S13b**.

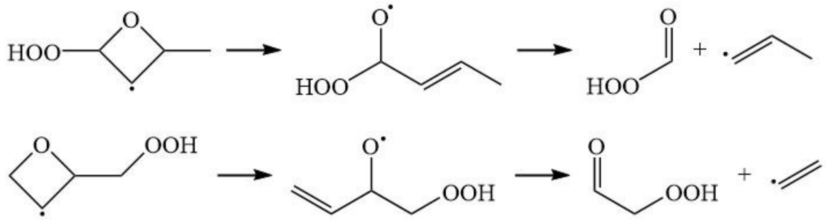

Figure S13b. Reaction mechanisms producing performic acid (top) and hydroperoxy acetaldehyde (bottom) from 2-methyloxetanylperoxy radicals.

The ion signal at *m/z* 62 and 76 at 650 K is shown in **Figure S13c**. Due to low signal-to-noise, the onset energy at *m/z* 62 appears to be in the range of ~ 10.0-10.2 eV. This aligns with the measured onset energy of vinyl chloride, at 9.98 eV, [1] and does not preclude the contribution of performic acid to the ion signal, which has an adiabatic ionization energy of 10.87 (± 0.05) eV, calculated at the ~QCISD(T)/CBS//M06-2X/aug-cc-pVTZ level of theory [2]. Meanwhile, the onset energy at *m/z* 76 is 10.0 eV, which agrees with the measured onset energy of allyl chloride, 10.04 (± 0.01) eV [3]. We employed the CBS-QB3 level of theory to calculate an adiabatic ionization energy of 9.61 eV for hydroperoxy acetaldehyde. Rousso et al. [4] performed a conformer search at the ~CCSD(T)/CBS//M06-2X/cc-pVTZ level of theory and found that the adiabatic ionization energy of hydroperoxy acetaldehyde varies from 9.60 to 9.89 eV, with the lowest-energy conformer at 9.76 eV. They also reported an uncertainty of ± 0.05 eV on their calculations. As a result, and in combination with the observation of ^{35}Cl and ^{37}Cl at *m/z* 62 and 64, as well as at *m/z* 76 and 78, we can conclude with certainty that vinyl chloride and allyl chloride contribute significantly to the ion signal at *m/z* 62 and 76 (**Figure S13c**). However, the contribution of performic acid and hydroperoxy acetaldehyde is less clear.

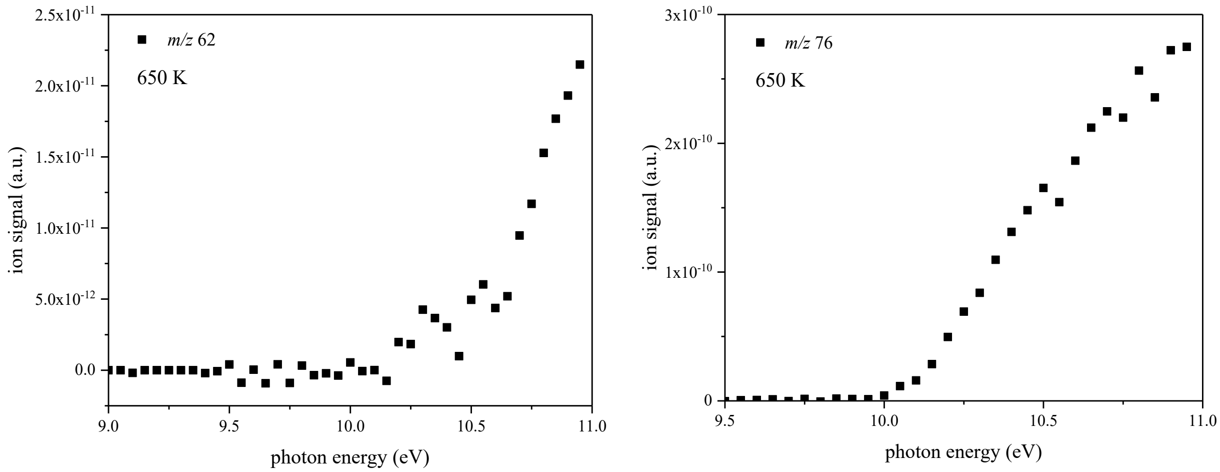

Figure S13c. Raw *m/z* 62 and 76 photoionization spectra at 30 ms post-photolysis, 650 K, and 6 Torr.

We employed several strategies in an attempt to decouple the contribution of vinyl chloride and allyl chloride with the possible contributions of performic acid and hydroperoxy acetaldehyde to the ion signals at *m/z* 62 and 76, respectively. First, we calculated the exact mass of the chlorinated species and ketohydroperoxides and found negligible differences. For example, the mass of performic acid is 62.00039 amu, while that of vinyl chloride is 61.99233 amu. This difference is too small to be resolved by the mass spectrometer utilized in the present experiments ($\Delta m/m$ ~1500). Next, we compared the time traces of *m/z* 62 and 76 against that of *m/z* 106 (**Figure S13d**) to determine if there is a narrow time window after photolysis over which there is significant contribution from ketohydroperoxides to the total ion signal. **Figure S13d** shows that the shape of the *m/z* 62 ion signal from 20-50 ms is consistent with the shape of the *m/z* 106 ion signal. However, **Figure S13d** also shows that, for *m/z* 76, there is a sharp rise in signal from ~20-22 ms, while that of *m/z* 106 is more gradual. Both plots in **Figure S13d** indicate that any contribution from the ketohydroperoxides is most likely to occur in the first 1-3 ms after photolysis.

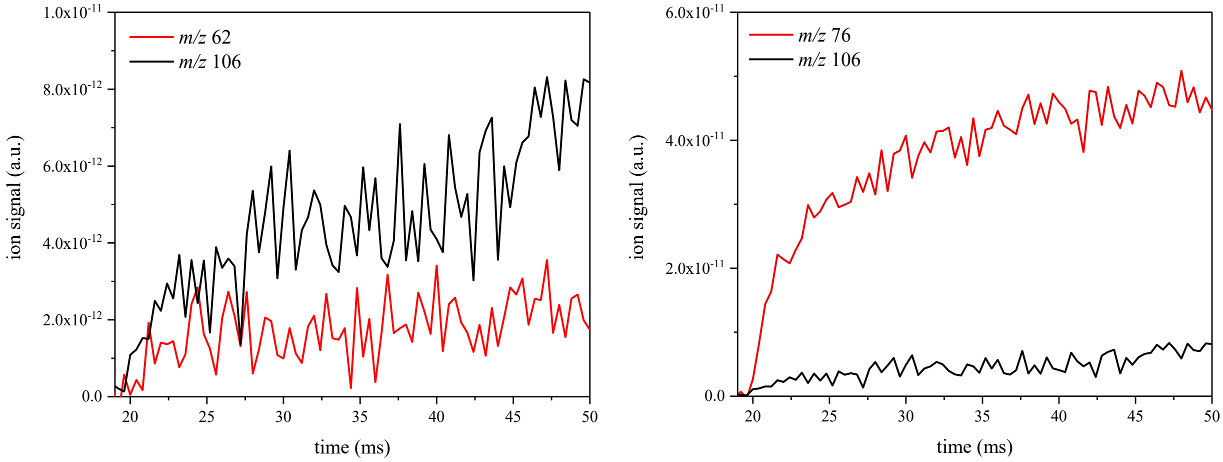

Figure S13d. Time profiles for (a) *m/z* 62 and (b) *m/z* 76 against *m/z* 106, measured at 650 K and 6 Torr, and integrated over the photon energy range 9.0-11.0 eV.

We probed this further by plotting the photoionization spectra of *m/z* 62 and 76 in the first 2 ms after photolysis and comparing to the photoionization spectra at both masses integrated over the entire kinetic window (20-50 ms), where, presumably, a majority of the signal arises from chlorinated photofragments of RCl. This is shown in **Figure S13e**, where there is no significant difference between the overall ion signal and that from 20-22 ms at *m/z* 62. At *m/z* 76, there may be a difference in the two spectra above ~10.3 eV, but the low signal-to-noise prevents a definitive conclusion. As a result, due to limitations in our experimental setup (*i.e.,* chain-chlorination), we are unable to prove, or disprove, that performic acid and hydroperoxy acetaldehyde contribute the ion signal at *m/z* 62 and 76.

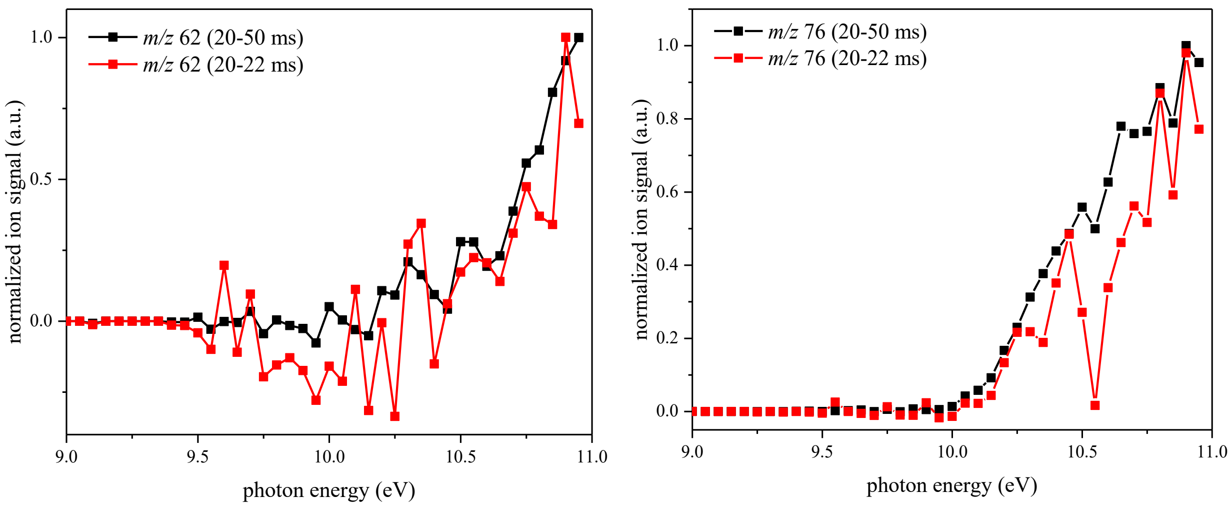

Figure S13e. Photoionization spectra at (a) *m/z* 62 and (b) *m/z* 76 measured at 650 K and 6 Torr, and integrated (black) from 20-50 post-photolysis and (red) 20-22 ms post-photolysis. The ion signal over 20-22 ms for *m/z* 62 and 76 was scaled by a factor of ~25 and ~30, respectively, to compare to the overall signal.

References

[1] L. Sheng, F. Qi, L. Tao, Y. Zhang, S. Yu, C.-K. Wong, W.-K. Li, Experimental and theoretical studies of the photoionization and dissociative photoionizations of vinyl chloride, Int. J. Mass Spectrom. Ion Process. 148 (1995) 179-189.
[2] K. Moshammer, A.W. Jasper, D.M. Popolan-Vaida, A. Lucassen, P. Dievart, H. Selim, A.J. Eskola, C.A. Taatjes, S.R. Leone, S.M. Sarathy, Y. Ju, P. Dagaut, K. Kohse-Hoinghaus, N. Hansen, Detection and identification of the keto-hydroperoxide ($HOOCH_2OCHO$) and other intermediates during low-temperature oxidation of dimethyl ether, J. Phys. Chem. A 119 (2015) 7361-7374.
[3] R.D. Buff, A.C. Parr, A.J. Jason, The photoionization of allyl chloride from onset to 20 eV, Int. J. Mass Spectrom. Ion Phys. 40 (1981) 31-34.
[4] A.C. Rousso, N. Hansen, A.W. Jasper, Y. Ju, Low-temperature oxidation of ethylene by ozone in a jet-stirred reactor, J. Phys. Chem. A 122 (2018) 8674-8685.

S14. prescribed consumption reactions of 2-methyloxetane in chemical kinetics mechanisms

Significant effort has focused on calculating high-fidelity rate coefficients for cyclic ether formation via Q̇OOH → cyclic ether + ȮH [1-8]. However, consumption reactions of cyclic ethers have traditionally been neglected in chemical kinetics mechanisms, such as 2-methyloxetane in mechanisms of *n*-butane [9-14]. This uncertainty in the fate of cyclic ethers results in a variety of different consumption reactions that are prescribed in mechanisms. For example, **Figure S14a** shows the consumption reactions assigned in the mechanisms of Cord et al. [9] and Biet et al. [10]. While the mechanism of Biet et al. was recently updated by Duan et al. [11], the reactions for 2-methyloxetane remained unchanged. As shown in **Figure S14a**, H-abstraction and subsequent O_2 addition are included in the mechanisms, including the formation and decomposition of ketohydroperoxides. However, each isomer is lumped into one representative species. For example, R represents all four 2-methyloxetanyl radicals.

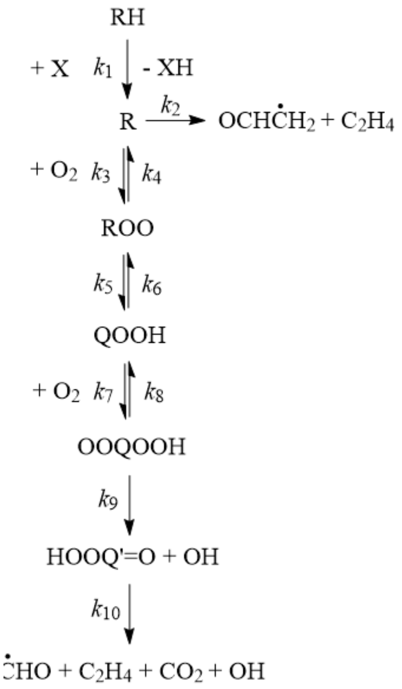

reaction	A (cm, mol, s)	n	E_a (kcal mol^{-1})
k_1	$2.70 \cdot 10^{6}$	2.00	0.45
k_2	$5.00 \cdot 10^{13}$	0.00	24.8
k_3	$3.00 \cdot 10^{19}$	-2.50	0.00
k_4	$5.00 \cdot 10^{22}$	-2.50	40.0
k_5	$8.00 \cdot 10^{13}$	0.00	25.5
k_6	$4.80 \cdot 10^{12}$	0.00	19.0
k_7	$3.00 \cdot 10^{19}$	-2.50	0.00
k_8	$5.00 \cdot 10^{22}$	-2.50	40.0
k_9	$1.00 \cdot 10^{9}$	0.00	7.50
k_{10}	$3.75 \cdot 10^{15}$	0.00	43.0

RH = 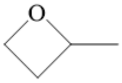

X = H, OH, HO_2, CH_3, CH_3OO, C_2H_5

Figure S14a. Reaction scheme prescribed for 2-methyloxetane in the mechanisms of Cord et al. [9] and Biet et al. [10]. Arrhenius parameters for each reaction are labeled in the scheme and listed on the right. The Arrhenius parameter for k_1 is for abstraction by OH.

On the other hand, **Figure S14b** shows the consumption reactions assigned in the mechanisms of Healy et al. [12], Bugler et al. [13], and NUIGMech1.1 [14,15]. These mechanisms do not consider 2-methyloxetanyl radicals, which is in contrast to those described above. Here, H-abstraction and subsequent ring-opening is lumped into one step. In addition, the Arrhenius parameters assigned in these mechanisms are arbitrary because the reactions span multiple transition states.

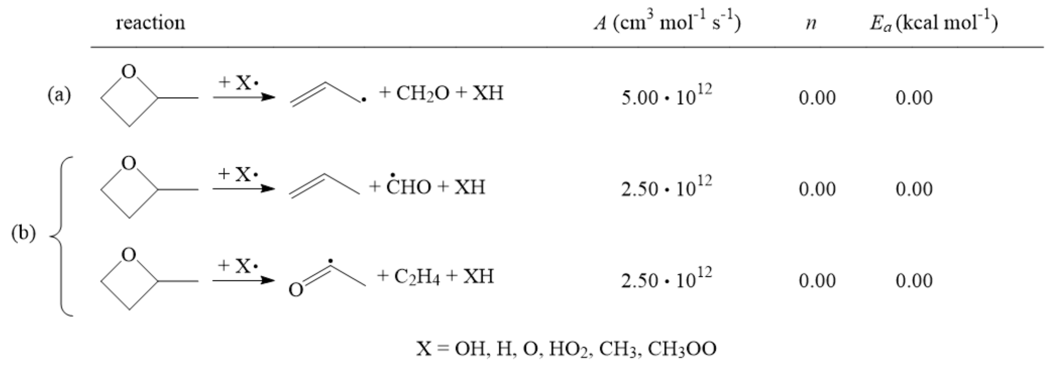

Figure S14b. Reaction type and Arrhenius parameters assigned for 2-methyloxetane in the mechanisms of (a) Healy et al. [12] and Bugler et al. [13], and (b) NUIGMech1.1 [14,15]. Arrhenius parameters listed are for abstraction by OH.

References

[1] W.-T. Chan, H.O. Pritchard, I.P. Hamilton, Dissociative ring-closure in aliphatic hydroperoxyl radicals, Phys. Chem. Chem. Phys. 16 (1999) 3715-3719.
[2] C.D. Wijaya, R. Sumathi, W.H. Green, Thermodynamic properties and kinetic parameters for cyclic ether formation from hydroperoxyalkyl radicals, J. Phys. Chem. A 107 (2003) 4908-4920.
[3] H. Sun, J.W. Bozzelli, Thermochemical and kinetic analysis on the reactions of neopentyl and hydroperoxy-neopentyl radicals with oxygen: part i. OH and initial stable HC product formation, J. Phys. Chem. A 108 (2004) 1694-1711.
[4] A. Miyoshi, Systematic computational study on the unimolecular reactions of alkylperoxy (RO_2), hydroperoxyalkyl (QOOH), and hydroperoxyalkylperoxy (O_2QOOH) radicals, J. Phys. Chem. A 115 (2011) 3301-3325.
[5] S.M. Villano, L.K. Huynh, H.-H. Carstensen, A.M. Dean, High-pressure rate rules for alkyl + O_2 reactions. the isomerization, cyclic ether formation, and *β*-scission reactions of hydroperoxy alkyl radicals, J. Phys. Chem. A 116 (2012) 5068-5089.
[6] M. Cord, B. Sirjean, R. Fournet, A. Tomlin, M. Ruiz-Lopez, F. Battin-Leclerc, Improvement of the modeling of the low-temperature oxidation of *n*-butane: study of the primary reactions, J. Phys. Chem. A 116 (2012) 6142-6158.
[7] C.F. Goldsmith, W.H. Green, S.J. Klippenstein, Role of O_2 + QOOH in low-temperature ignition of propane. 1. temperature and pressure dependent rate coefficients, J. Phys. Chem. A 116 (2012) 3325-3346.
[8] J. Bugler, J. Power, H.J. Curran, A theoretical study of cyclic ether formation reactions, Proc. Combust. Inst. 36 (2017) 161-167.
[9] M. Cord, B. Sirjean, R. Fournet, A. Tomlin, M. Ruiz-Lopez, F. Battin-Leclerc, Improvement of the modeling of the low-temperature oxidation of *n*-butane: Study of the primary reactions, J. Phys. Chem. A 116 (2012) 6142-6158.
[10] J. Biet, M.H. Hakka, V. Warth, P.-A. Glaude, F. Battin-Leclerc, Experimental and modeling study of the low-temperature oxidation of large alkanes, Energy Fuels 22 (2008) 2258-2269.
[11] J. Duan, J. Ji, L. Ye, Y. Zhai, L. Zhang, A theoretical kinetics study on low-temperature oxidation of n-C_4H_9 radicals, Proc. Combust. Inst. 38 (2021) 681-689.
[12] D. Healy, N.S. Donato, C.J. Aul, E.L. Petersen, C.M. Zinner, G. Bourque, H.J. Curran, *n*-Butane: Ignition delay measurements at high pressure and detailed chemical kinetic simulations, Combust. Flame 157 (2010) 1526-1539.
[13] J. Bugler, A. Rodriguez, O. Herbinet, F. Battin-Leclerc, C. Togbe, G. Dayma, P. Dagaut, H.J. Curran, An experimental and modelling study of *n*-pentane oxidation in two jet-stirred reactors: The

importance of pressure-dependent kinetics and new reaction pathways, Proc. Combust. Inst. 36 (2017) 441-448.
[14] A.A.E.-S. Mohamed, S. Panigrahy, A.B. Sahu, G. Borque, H.J. Curran, An experimental and kinetic modeling study of the auto-ignition of natural gas blends containing C1-C7 alkanes, Proc. Combust. Inst. 38 (2021) 365-373.
[15] Y. Wu, S. Panigrahy, A.B. Sahu, C. Bariki, J. Beeckmann, J. Liang, A.A. Mohamed, S. Dong, C. Tang, H. Pitsch, Understanding the antagonistic effect of methanol as a component in surrogate fuel models: A case study of methanol/n-heptane mixtures, Combust. Flame 226 (2021) 229-242.
